# Towards predicting the geographical origin of ancient samples with metagenomic data

**DOI:** 10.1038/s41598-023-40246-x

**Published:** 2024-09-18

**Authors:** Davide Bozzi, Samuel Neuenschwander, Diana Ivette Cruz Dávalos, Bárbara Sousa da Mota, Hannes Schroeder, J. Víctor Moreno-Mayar, Morten E. Allentoft, Anna-Sapfo Malaspinas

**Affiliations:** 1 Department of Computational Biology, University of Lausanne, 1015 Lausanne, Switzerland; 2 Swiss Institute of Bioinformatics, 1015 Lausanne, Switzerland; 3 Vital-IT, SIB Swiss Institute of Bioinformatics, 1015 Lausanne, Switzerland; 4 Globe Institute, Faculty of Health and Medical Sciences, University of Copenhagen, Copenhagen, Denmark; 5 Lundbeck Foundation GeoGenetics Centre, Globe Institute, University of Copenhagen, Copenhagen, Denmark; 6 Trace and Environmental DNA (TrEnD) Laboratory, School of Molecular and Life Sciences, Curtin University, Perth, WA Australia

**Keywords:** Computational biology and bioinformatics, Microbiology

## Abstract

Reconstructing the history—such as the place of birth and death—of an individual sample is a fundamental goal in ancient DNA (aDNA) studies. However, knowing the place of death can be particularly challenging when samples come from museum collections with incomplete or erroneous archives. While analyses of human DNA and isotope data can inform us about the ancestry of an individual and provide clues about where the person lived, they cannot specifically trace the place of death. Moreover, while ancient human DNA can be retrieved, a large fraction of the sequenced molecules in ancient DNA studies derive from exogenous DNA. This DNA—which is usually discarded in aDNA analyses—is constituted mostly by microbial DNA from soil-dwelling microorganisms that have colonized the buried remains post-mortem. In this study, we hypothesize that remains of individuals buried in the same or close geographic areas, exposed to similar microbial communities, could harbor more similar metagenomes. We propose to use metagenomic data from ancient samples' shotgun sequencing to locate the place of death of a given individual which can also help to solve cases of sample mislabeling. We used a k-mer-based approach to compute similarity scores between metagenomic samples from different locations and propose a method based on dimensionality reduction and logistic regression to assign a geographical origin to target samples. We apply our method to several public datasets and observe that individual samples from closer geographic locations tend to show higher similarities in their metagenomes compared to those of different origin, allowing good geographical predictions of test samples. Moreover, we observe that the genus *Streptomyces* commonly infiltrates ancient remains and represents a valuable biomarker to trace the samples' geographic origin. Our results provide a proof of concept and show how metagenomic data can also be used to shed light on the place of origin of ancient samples.

## Introduction

The extraction and sequencing of DNA from ancient samples using shotgun sequencing techniques is becoming more and more common thanks to the advancements in ancient DNA (aDNA) laboratory protocols and the drop in sequencing costs^1,2^. Shotgun sequencing of ancient bones and teeth not only allows the study of ancient animals but also permits the recovery of aDNA from host-associated microorganisms. This has fuelled an entirely new field of research that focuses on the study of ancient microbes: paleomicrobiology^3,4^.

However, DNA degradation tends to reduce the amount of endogenous DNA that can be obtained from ancient samples^5^. Furthermore, microbes colonize the remains post-mortem, and their DNA is usually sequenced alongside the endogenous DNA, resulting in most aDNA sequencing experiments containing a lot of data from exogenous microorganisms^6^. This DNA is usually discarded in aDNA studies interested in the host or host-associated microbes as it mostly reflects the community of microorganisms that live in the surrounding soil, including those that actively colonize the bones (hereafter the bone necrobiome)^7–9^.

To the exclusion of recent excavations, the exact sampling location of ancient human remains might be unknown. Numerous samples were excavated decades or centuries ago and, since then, a lot may have happened, including change of ownership, international shipments and change of storage site. Thus, for some old museum collections, information regarding the sampling location might be missing or misleading. This poses major challenges to the aDNA field which heavily relies on such collections.

To reconstruct the human past and study human migrations, it is fundamental to correctly trace the place of birth and death of an individual. Discrepancies between the two locations can provide information about human mobility patterns in the past. The analysis of human DNA can give clues about the ancestors of a given person identifying the putative place of origin of an individual or of its ancestors, however, it does not allow us to identify the place where the individual died. Consequently, ancestry analysis on human DNA alone cannot distinguish genuine patterns of human migration from cases of samples mislabeling or remains moved after death, and it cannot make use of samples with unknown sampling location. Isotope data (e.g., strontium ^87^Sr/^86^Sr ratios) can help identify the place where an individual was born and lived^10,11^ and have proved useful to identify migrants^12–16^. However, their application is limited to few geographical areas where reference data are available and can be deceived if different locations harbor similar isotope values. Moreover, it has been demonstrated that human agricultural practices can bias the common strontium isotope analyses by affecting the accuracy of strontium reference maps^17^.

Besides human genetics and isotopes, another promising lead to trace human migrations is the study of host-associated microorganisms. Seminal studies have shown that microorganisms exhibiting patterns of co-evolution with the human host^18,19^, or pathogens that are environmentally acquired during life^20^, hold potential to elucidate human migrations. However, while useful to trace human movements, these data have not been used to assess the individuals' place of death.

Methods that can specifically trace the location of death of an individual would allow us to identify the geographical origin of displaced remains with unknown sampling location and identify mislabeled ones, assisting the analysis of ancient human remains in archaeogenetic studies and/or helping with the repatriation processes^21,22^. In addition, such tools could hold potential to serve other disciplines, such as forensic analyses.

Based on the observation that most of the non-human DNA in ancient bone metagenomes is of exogenous origin, we hypothesize that remains of individuals buried in the same or close geographic areas, exposed to similar microbial communities, could harbor more similar metagenomes. Here we propose to use metagenomic data from shotgun sequencing to locate the place of death of a given individual which can also help to solve cases of sample mislabeling.

To infer the geographic origin of a given sample, we rely on published reference data that includes individuals with known geographical locations. The approach we then propose relies on two steps; first, the computation of a distance matrix to measure how dissimilar the sample of interest is compared to a reference set. Second—based on this computed matrix—and using a logistic regression model trained on the reference set, the individual is classified into one of the represented geographical locations.

Inter-sample similarities can be computed in several ways. Often, reads classification steps are involved to generate taxa relative abundance tables, which are in turn used for the computation of inter-samples similarity scores. One of the disadvantages of this method is that some of the reads remain unclassified, as they are not represented in the reference database used for the classification, or because they cannot be traced to a specific group of microorganisms. The data loss caused by this procedure is related to the amount of unknown microorganisms present in the studied metagenome and, therefore, more strongly affects poorly characterized environments like the soil^23^. In the last decade, several alignment-free approaches relying on k-mers have been developed to compute metagenome similarity scores in a fast and scalable way without the need for a sequence classification step^24–26^, and different studies have demonstrated their accuracy and usefulness^27–29^. Hence, to take advantage of all the metagenomic reads from the ancient human samples, we opted for a k-mer-based approach to compute similarity scores.

We analyzed previously published datasets and trained a model capable of discriminating individuals from two different geographic locations, allowing us to determine the place of death of each given individual when excluded from model training. We then revisit a potential case of museum mislabeling in which two individuals were identified as non-locals based on human DNA ancestry analysis^30^. Furthermore, we considered the potential impact of batch effects that would result from the way the experiments were set up. And finally, we evaluated how well our method would perform when applied to a single, soil-dwelling, microbial genus, an approach that allows us to mostly bypass batch effects.

## Results

To determine whether ancient metagenomic data can be used to inform on the place of death of ancient humans, we selected published datasets from five different studies, each including individuals from distinct geographic locations (see method section for more details). We used these data to compile two binary dataset each one including individuals from two distinct geographical locations. In one dataset (hereafter called Brazil-Polynesia dataset) we included ancient individuals from different areas in Brazil^31^ and ancient individuals from Polynesia^32^. The second dataset (hereafter Denmark-England dataset) included ancient individuals from Denmark and England^33^. Both datasets comprise bone and tooth dentine/cementum samples. For the Brazil-Polynesian dataset two different extraction protocols were used^34,35^. These differences were taken into consideration in the analysis of the Brazil-Polynesia dataset to inspect the potential impact of batch effects on the analysis. We note that the extraction protocol and sample type variables correlate partially, but not perfectly, with the geographic origin variable. For the Denmark-England dataset all the data were generated in the same study and samples were subjected to the same laboratory procedure, avoiding the risk of a laboratory-induced batch effect, enabling us to test our method in an ideal scenario. For both datasets, the Illumina shotgun data (raw FASTQ files) were preprocessed to remove human reads and possible lab contaminants prior to any analysis (see methods). In other words, we filtered the data to analyze only the samples' microbial metagenome (i.e. the exogenous component)—(Supplementary Fig. 1).

### The ancient bone metagenome is enriched in soil-derived microorganisms

As a first step to our analysis, we inspected the bacterial composition of the used datasets. We wanted to better understand which microbiome sources contributed the most to the ancient bones and teeth metagenome.

**Figure 1:**
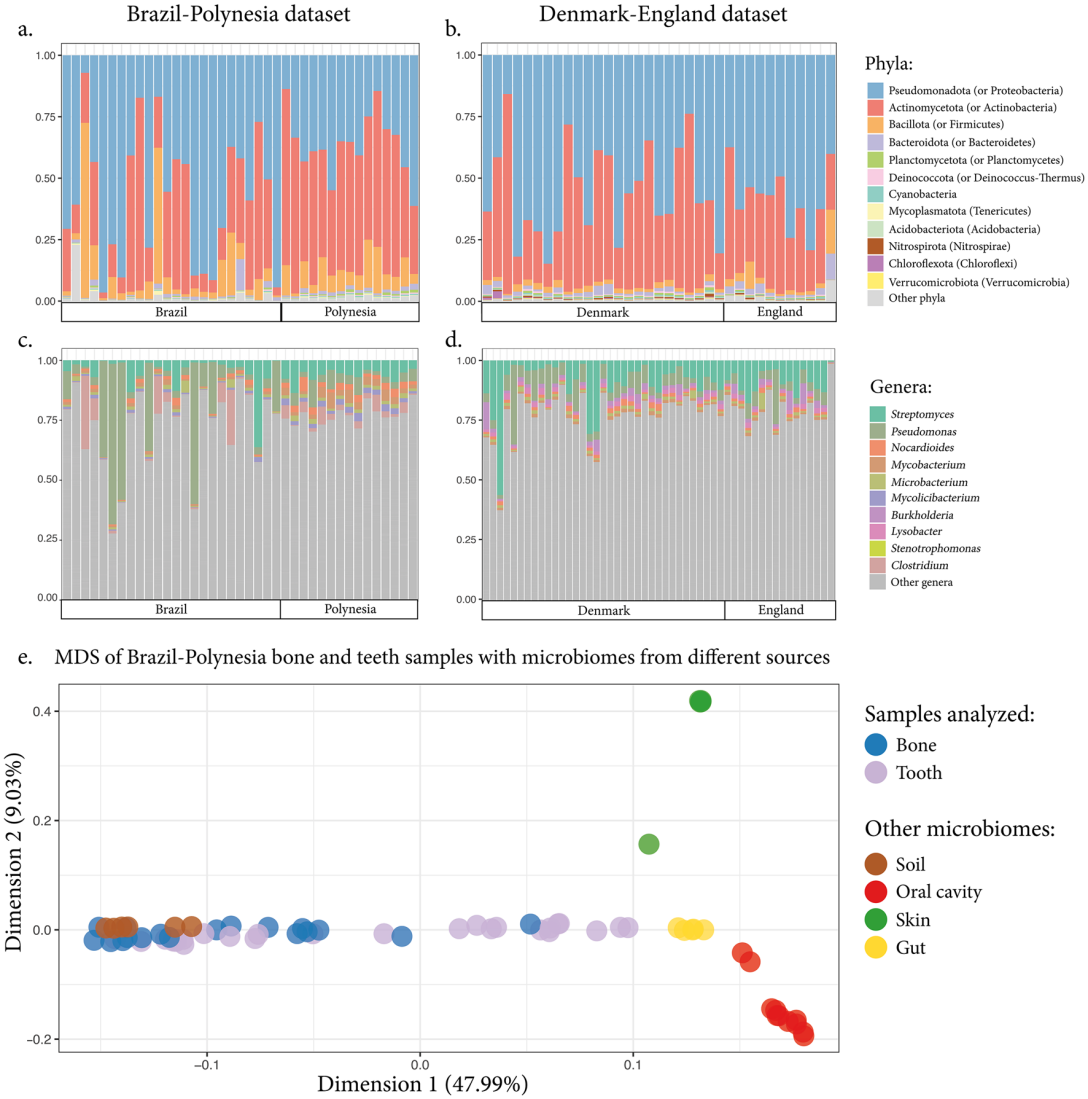
Compositional analysis of ancient samples. The microbial composition of the ancient samples at the phylum (panels **a** and **b**) and genus (panels **c** and **d**) level for the Brazil-Polynesia dataset (left) and the Denmark-England dataset (right). We observe an overall enrichment of soil-dwelling bacteria genera. MDS plot (**e**) including bones and teeth samples from the Brazil-Polynesia dataset with microbiomes from publicly available data from other sources: soil^37^, human oral cavity^38^, skin^39^, and gut^40^. The MDS based on the Jaccard distance matrix shows that ancient bones and teeth cluster with modern soil samples and not with other sources, indicating that the metagenomic composition of the buried remains is more similar to the one found in the soil.

Compositional analysis of the metagenomes revealed Pseudomonadota (previously Proteobacteria) to be the most common phylum (highest median value across the samples) in both investigated datasets, followed by Actinomycetota (previously Actinobacteria), Bacillota (previously Firmicutes) and Bacteroidota (previously Bacteroidetes) (Fig. 1, panels a and b). At the genus level, the most represented clade (highest median value across the samples) was *Streptomyces* (~ 5.7% of the total number of classified reads in the two datasets), a group of soil-dwelling microorganisms known to be involved in organic matter degradation^36^, followed by *Pseudomonas* and other soil/water-dwelling or ubiquitous genera like *Nocardioides*, *Mycobacterium*, *Microbacterium*, *Burkholderia*, *Lysobacter,* etc. (Fig. 1, panels c and d). This suggests that the soil microbiome plays a crucial role in the ancient remains' microbial colonization, likely representing a major source for the bone necrobiome composition, and the long-term storage in the museum is not erasing this signal.

We then assessed where these ancient samples would fall on a classical multidimensional scaling (MDS) plot that included microbiome communities from different environments that could have contributed to the bone metagenome. As reference data, we used previously published data from different sources, including soil^37^, human oral cavity^38^, skin^39^, and gut^40^. We then taxonomically classified the reads and clustered them into taxa at the species and genus levels. We used the taxa table to compute inter-sample Jaccard distances and then performed dimensionality reduction. In this analysis, teeth and bone samples clustered with previously published soil samples to the exclusion of the other environments (Fig. 1e). Specifically, the first MDS dimension explained 47.99% of the variance and perfectly separated soil (reference microbial data) together with bone and teeth samples (from the ancient individuals analyzed) from the other sources (oral, skin, and gut microbiomes). Such a pattern is consistent with those reported in the literature^41^ and suggests a similar microbial colonization of the two sample types from environmental bacteria of the surrounding soil. This is likely related to the organic composition of bone and tooth dentine/cementum; both tissues present a very similar composition and are mostly constituted of Type I collagen^42,43^. Some teeth samples separated less well from the host associated environments (human oral cavity, skin, and gut) on dimension 1 (Fig. 1e), presumably because they also experienced contamination from oral microbiome species. To explore this further, we performed microbial source tracking with SourceTracker2^44^ to infer the role of different potential sources of microorganisms. In the modeling we included the same samples used in the MDS plot and inferred the contribution of the following potential sources: soil^37^, human oral cavity^38^, skin^39^, and gut^40^. We also included ancient bone samples as a proxy for bone-specific taxa (i.e., taxa better modeled as part of the ancient bones rather than any other modeled source)^45,46^. Our results showed that, among the classified reads, soil and bones are the most common sources for our datasets with very little contribution of the other sources (Supplementary Fig. 2). An exception were some teeth samples where the oral cavity seems to contribute with a relevant number of taxa, suggesting that oral microbiome species DNA can leach into the sample dentine/cementum, in agreement with the previous MDS results. We also note that most of the taxa cannot be traced to any modeled source. This is likely a consequence of the high soil microbial biodiversity and suggests that the reference microbiome samples used in the modeling are not sufficient to give a complete description of all the possible soil communities. Together these observations support the hypothesis that the bone/tooth necrobiome is enriched in soil-derived species suggesting its potential use for geographical tracing of the samples.

The invasion of bone and teeth samples from soil-dwelling microorganisms is likely a continuous process that starts soon after death and continues until the samples is eventually unearthed. Therefore, we should expect the DNA recovered from these samples to be a mixture of DNA from different periods. To determine the antiquity of the DNA constituting these metagenomes, we assessed the presence of typical aDNA damage patterns with MetaDamage^47^. We detected a positive increase of cytosine deaminations (C to T transitions) at the 5' end of the reads compared to the rest of the sequences in almost all the considered samples (Supplementary Fig. 3) suggesting the presence of aDNA damage in the analyzed reads, in agreement with the fact that all the samples were unearthed long ago and subsequently stored in a museum. We also note that filtering out reads longer than 80 bp does not lead to a large increase in damage at the last base at the 5’ end (only + 4.5% as median value), suggesting that there was relatively little modern DNA contamination or that the initial reads collapsing step, in which all the long reads that cannot be collapsed were discarded, already removes most of the modern contaminants (Supplementary Fig. 3).

### K-mer-based similarities reveals geographic-dependent samples clustering patterns

**Figure 2:**
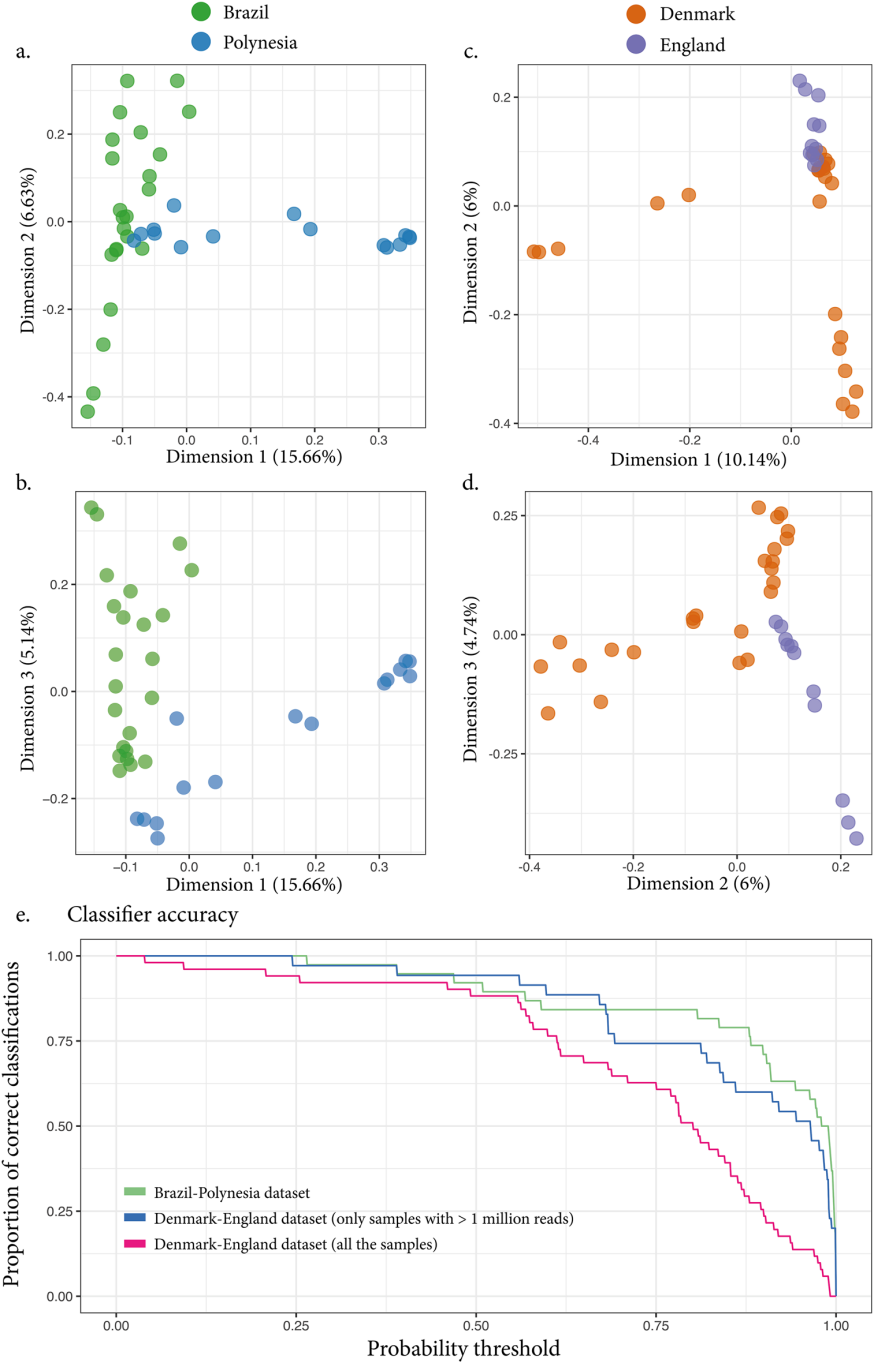
MDS based on the kmer data and accuracy assessment of the logistic regression approach. Some dimensions in the MDS plot visually separate the samples according to geography. Panels (**a**) and (**b**) show the first 3 dimensions for the Brazil-Polynesia dataset MDS. Panels (**c**) and (**d**) show the first 3 dimensions for the Denmark-England dataset MDS. Logistic regression. A logistic regression model was trained for both test datasets (Brazil-Polynesia and Denmark-England) using MDS dimensions as input. After training, we assessed the model accuracy with leave-one-out cross-validation. The accuracy curves (panel **e**) show the decrease in the number of individual samples correctly classified at increasing thresholds of classification probability. At higher thresholds, more individual samples are correctly classified for the Brazil-Polynesia dataset (green line). For the Denmark-England dataset better classifications are obtained when including only the samples with more than a million reads (blue line) compared to including all the available samples in the dataset (magenta line), indicating that the geographical origin of low-depth samples cannot be predicted with good confidence.

For each dataset we computed k-mer-based metagenome similarities scores (k-mer size = 21 bp). Dimensionality reduction is a powerful approach that transforms high dimensional data to a lower dimensional space while keeping relevant properties of the original data. In MDS, similarity scores between multiple samples are projected into the Euclidean space where each dimension constitutes an independent variable (i.e., the dimensions are not correlated between each other). When dimensionality reduction is performed on the k-mer-based similarity matrix, we observed that, for all datasets, there are dimensions that separate the samples according to their geographic origin (see Fig. 2, panels a,b,c,d for some examples). This suggests that the metagenomic data harbor a location-specific signature and that the MDS dimensions can be utilized for classification purposes. While a perfect separation of the groups using only two dimensions could be achieved for both datasets (Fig. 2, panels b and d), we wanted a quantitative way of predicting the origin of a test sample given a reference panel with individuals whose origins are known. One way to do this is to train logistic regression (logit) models on reference datasets to perform classification of test samples. Logit models can be trained on one or multiple independent variables to perform binary or multinomial classification tasks. The trained model can then be used to predict the class of a test sample. For each test sample, the model returns its probability of being classified to one of the tested groups. In our case, we trained the model to predict a sample geographic origin using the MDS dimensions as input to the model. To solve the problem of feature selection (i.e., which dimension to include in the model training) and to avoid overfitting problems due to the low sample size, we trained a penalized logistic regression model with lasso regularization while providing as input all the MDS dimensions. By design of this approach, informative features (MDS dimensions) are selected by the model as input for the training step while uninformative dimensions are discarded. The model can then be used to predict the origin of target samples. Specifically, the output of the model prediction is the probability of a sample to be part of one of the two groups used for the model training.

### Accuracy assessment of sample geographic origin prediction

The method classification accuracy was assessed with leave-one-out cross validation (jackknifing). At each iteration, one sample was left out of the training and used for testing. We first assessed the classification accuracy when the model was trained to predict the sample geographic origin and evaluated how confident the classifier was. To do this we calculated how many samples were correctly classified at increasing thresholds of probability. In the Brazil-Polynesia dataset, ~ 92% of all the samples were correctly classified (Fig. 2e) (i.e., assigned to the correct class with probability $\ge$ 0.5), indicating that only very few samples were misclassified by the model (very few false positives). However, we want a model that not only correctly classifies a sample to its group, but that it does so at high probability. For this dataset ~ 60% of the samples were classified to the correct class with probability $\ge$ 0.95 (Fig. 2e). For the Denmark-England dataset, the accuracy at a probability threshold of 0.5 was ~ 94% but fewer samples (~ 51%) were correctly classified with probability $\ge$ 0.95 (Fig. 2e). Interestingly, for this dataset, when samples with less than 1 million reads were included, the confidence of the method dropped (Fig. 2e, magenta line), indicating that low-depth samples cannot be classified with good confidence. In this case, only ~ 14% of the samples were correctly classified with probability $\ge$ 0.95, and 12% of the samples were mis-classified.

**Figure 3:**
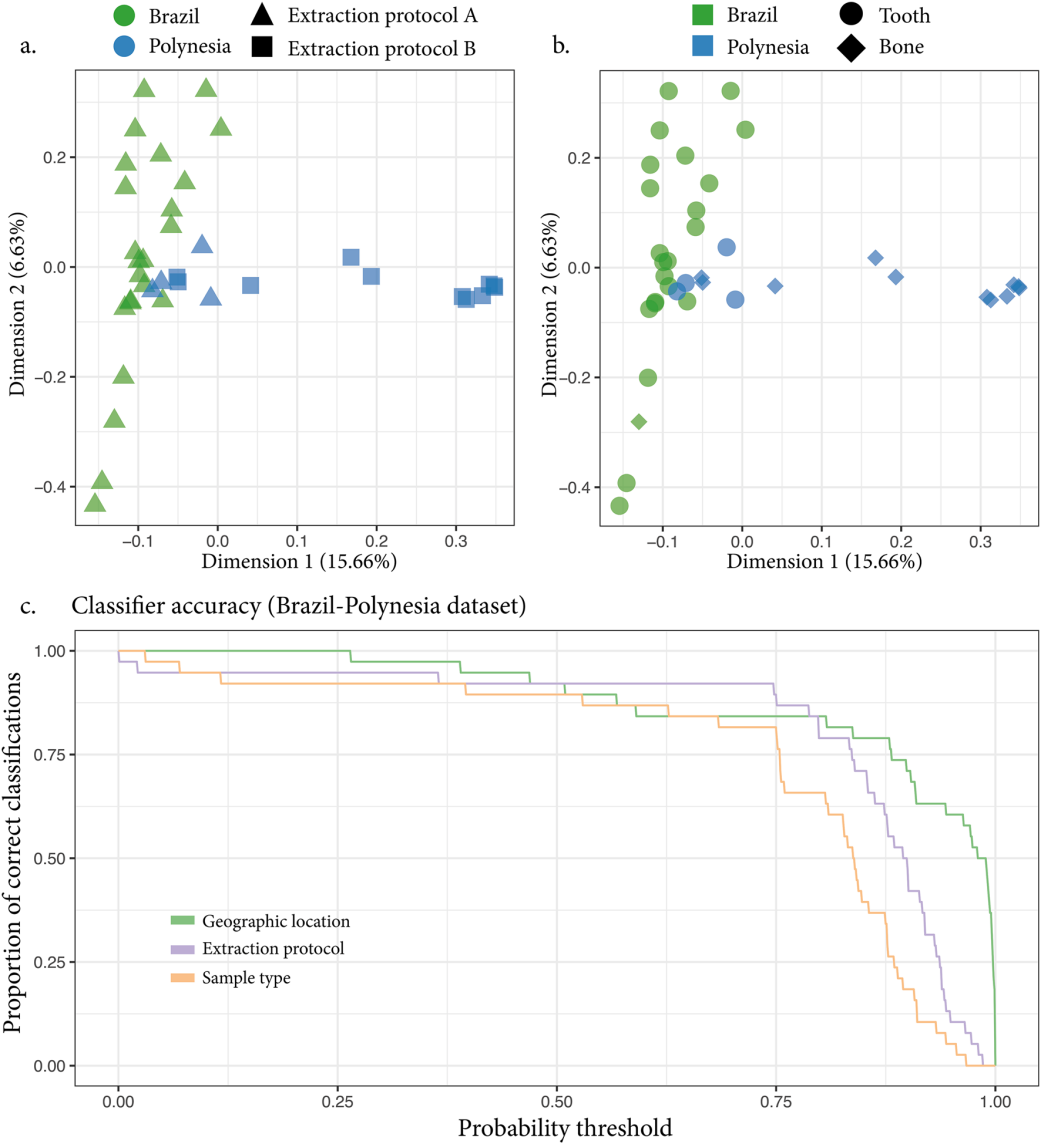
Model prediction of other variables in the Brazil-Polynesia dataset. We evaluated the possibility of training a model on the Brazil-Polynesia dataset to also predict the extraction protocol (**a**) and the sample type (**b**). Data for the Brazil-Polynesia dataset were generated using different extraction protocols and by extracting the DNA from two different sample types: bones and teeth. To test the potential effect exerted by such variables we assessed to which degree of accuracy they could be predicted. MDS was used to extract dimensions with which to train the model (panel **a** and **b**, in green individual samples from Brazil, in blue individual samples from Polynesia) and the classification accuracies were evaluated with jackknifing and compared to those obtained for the geographic origin classification (**c**). We note that both extraction protocol and sample type can be predicted with good accuracy warning for potential batch effects. Panel (**a**) and (**b**) show that the three variables partially correlate, indicating that the prediction of irrelevant variables might be driven by the relevant ones. We note that model predictions for the variable “geographic origin” outperforms the predictions for the other variables in terms of number of samples correctly classified with high probability (> 0.8) (**c**).

For the Brazil-Polynesia dataset we also evaluated to which degree our method could be trained to predict other variables such as the extraction protocol and the type of sample from which the DNA was extracted (i.e., bone vs tooth) (Fig. 3). We trained the model to predict the extraction protocol and estimated its accuracy at 0.5 probability threshold to ~ 92%, the same value obtained for geography. However only ~ 10% of the samples were correctly classified with probability $\ge$ 0.95 (Fig. 3c). Note that the extraction protocol in the Brazil-Polynesia dataset partially correlated with geography (Fig. 3a). When the model was trained to predict the sample type (bone vs tooth), 90% of the samples were correctly classified at 0.5 probability threshold and only ~ 5% of the samples were classified to the correct sample type with probability $\ge$ 0.95 (Fig. 3c), indicating that a lower classification accuracy could be achieved for the sample type compared to the other examined variables. This is in line with a similar bacterial colonization of bones and teeth dentine/cementum and suggests a more limited contribution of sample type compared to geographic location or the extraction protocol to the metagenome composition.

As a negative control, we trained the model with a mock variable after permuting the samples from different locations in two groups with equal proportions. In this case, informative dimensions cannot be found and the model cannot be trained to classify the samples according to the mock variable. This suggests that our results were likely driven by a biological signal and not by overfitting.

While the results presented above were obtained by selecting a k-mer size of 21 bp, we assessed the effect of k-mer size choice on the classifier performance by testing four other k-mer sizes: 11 bp, 15 bp, 25 bp and 31 bp (Supplementary Fig. 4). We note that, while for k-mer sizes of 15 bp, 21 bp, and 25 bp the results are qualitatively similar, the choice of k-mer size is relevant and, at the same time, not obvious, with different k-mer sizes performing differently on different datasets. This was especially true for the geographic origin prediction, while smaller differences were observed for the other two variables, namely the extraction protocol and the sample type (Supplementary Fig. 4). In general, based on our results, we discourage using very short k-mers (e.g. 11 bp), likely because of reduced specificity in the sequence information, or very long k-mers (e.g. 31 bp), as this reduces the similarity scores between samples as it increases the chance of two samples not sharing any k-mer. In general, assessment of k-mer size on model performance is advised to identify the optimal value.

As an alternative to logistic regression, we also trained and tested in the same way a random forest classifier and compared the performance of the two methods. ROC curves show how the two approaches performed similarly in many cases (Supplementary Fig. 5). However, logistic regression usually outperformed random forest classifiers for samples’ geographic origin prediction, except in the case of the Denmark-England dataset when including low depth samples. In contrast, random forest performed better on the other tested variables for the Brazil-Polynesia dataset (Supplementary Fig. 5).

Overall, our results demonstrate that at least in some cases (here two distinct datasets from several published studies), sample geographic origin can be predicted with accuracy using metagenomic data and k-mer-based similarities scores. We also show that other variables such as the extraction protocol and the sample type, which for our datasets partially correlate with geography, can be predicted, warning of potential biases that they might induce. However, we note that predictions for the variable "geographic origin" outperforms the predictions for the other two tested variables in terms of number of samples correctly classified with high probability (> 0.8).

### Assessing the effect of shared long-term museum storage

We also investigated the possible effect of long-term storage in the same museum by classifying, with the model trained on the Brazil-Polynesia dataset, three Fuego-Patagonian individuals^48^ that have been stored in the same museum (Musée de l´Homme—Paris, France) as the Polynesian individuals that were used to train the model. For all three Fuego-Patagonian samples, the Polynesian classification probability was $\le$ 20%, thus providing little support for a potential storage-induced bias. This suggests that, for the data we analyzed, the museum-derived contamination was either small or controlled by the laboratory and bioinformatic procedures.

### Geographic origin prediction of individuals with Polynesian ancestry found in Brazil

After training the model and assessing its accuracy we tested it on a real case scenario, using two mysterious samples of Polynesian ancestry allegedly found in Brazil and stored at the National Museum of Rio de Janeiro. Genomic analysis of these two individuals previously revealed that they were fully Polynesian in their ancestry^30^. We sought to test whether metagenomic similarities would place them with people from Brazil, suggesting that the individuals died and were buried there, or with people from Polynesia, suggesting that the remains were brought to Brazil only afterwards and that they were mislabeled. To do so, we classified them using the logistic regression classifier trained on all the data available for the Brazil-Polynesia reference dataset. Both samples were placed by the classifier with the Brazilian samples. In one case (Bot17) the classification was at high probability (> 99%), while for the other (Bot15) the probability was ~ 80%. Therefore, for both Polynesians, our results suggest that a scenario in which the individuals were buried in Brazil cannot be rejected (but see also below).

### *Streptomyces* genus is a potential biomarker for sample geographic origin prediction

Global metagenome similarities seem a promising avenue for detecting an ancient sample origin, however, as with all compositional studies they are susceptible to contamination or batch effect driven biases. Analyses restricted to specific soil-dwelling genera could in principle provide unbiased, less noisy, and potentially more powerful results, allowing for a more generalizable tool for sample geographic origin detection.

In our compositional analysis we identified some specific genera of soil-dwelling bacteria that are consistently found at high relative abundance in all ancient bone and teeth dentine/cementum samples offering potential for sample geographic origin prediction (Fig. 1). Among these genera we highlight the *Streptomyces* genus, previously reported in Neanderthal bones^49^ and found in all our samples with the highest relative abundance median value (~ 5.7% of the total number of classified reads in the two datasets after processing). *Streptomyces* are soil-dwelling filamentous bacteria belonging to the phylum Actinomycetota (previously Actinobacteria), a group of gram-positive bacteria known to be involved in organic matter decomposition^36^. They are also known to be producers of a wide range of antibiotics and to play important roles in soil ecology^36^. This microorganism seems, according to our data and previous analyses^49^, to be a typical member of the bone necrobiome. We report its presence in all the analyzed individual samples. Previous studies have reported a latitude-dependent diversity gradient^50^ for the *Streptomyces* genus, further suggesting its potential use for sample geographical tracing. Another advantage of this group of bacteria is that it does not include any known laboratory reagents contaminant^51,52^ and, as such, it should be robust to any laboratory contamination derived bias. Moreover, C to T transitions are increased a the 5’ end of the sequenced reads, a sign of cytosine deamination and a pattern that is typical of ancient DNA (Supplementary Fig. 6). Overall, all these characteristics, namely its soil-derived origin, its ubiquity and prevalence in ancient remains together with the certainty of it not being a laboratory contaminant, made this genus an ideal candidate to be further investigated for its potential use as a biomarker for sample geographic origin prediction.

**Figure 4:**
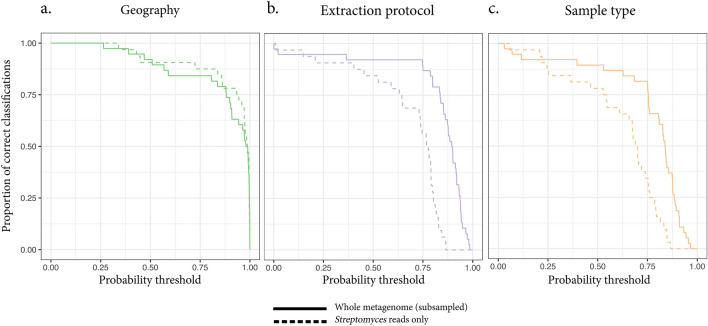
Comparison of the method prediction accuracy when using whole metagenomes or *Streptomyces* reads only. Accuracy assessment for geographic origin prediction in the Brazil-Polynesia dataset when using whole metagenomes (continuous lines) and when using *Streptomyces* reads only (dashed lines) for three different variables: geographic origin (**a**), extraction protocol (**b**) and sample type (**c**). When the method is applied to *Streptomyces* reads only, good sample geographic origin predictions are achieved while better controlling for batch effects (i.e., lower classification accuracy for the extraction protocol and the sample type).

We repeated the same analysis we ran for the whole metagenomes on the reads assigned to the *Streptomyces* genus. We restricted this analysis to the individual samples that contained a high number of *Streptomyces* reads (> 100.000). This limited the analysis to the Brazil-Polynesia dataset which contains samples that were sequenced at a higher depth. In this dataset, seven samples (all from Brazil) were discarded after this filtering step, leaving ~ 82% of the individual samples for the analysis. Moreover, the reads classified as *Streptomyces* in the Brazil-Polynesia dataset cumulatively account only for 0.7% of the whole metagenomic data. As before, we first computed k-mer-based inter-sample similarity scores and computed an MDS from the similarity matrix (Supplementary Fig. 7) and used its dimension as input for the logistic regression model. We then assessed the accuracy of the method in the same way we did for whole metagenomes data and evaluated how well the two approaches performed in predicting different variables such as geographic origin of the individual sample, extraction protocol used, and sample type (bone vs tooth) analyzed. Interestingly, we noted that the predictive power of our method for sample geographic origin was similar when using all the metagenomic data or when using only *Streptomyces* reads. Specifically, when using only *Streptomyces* data, the method was performing slightly better (Fig. 4a and Supplementary Fig. 8a). As expected, the extraction protocol could not be as well predicted on the *Streptomyces* genus compared to the whole metagenomes, suggesting this approach is more robust to experimental batch effects: no sample extraction protocol was correctly classified with a probability > 0.9 in this case, while, with the same probability, ~ 47% of the samples were correctly classified when using whole metagenomes (Fig. 4b and Supplementary Fig. 8a). We also noted that limiting the data to the *Streptomyces* genus affected the sample type predictions. Also in this case, no sample was correctly classified with probability > 0.9 compared to the ~ 18% when using whole metagenomes (Fig. 4c and Supplementary Fig. 8a). These results were expected since, in this case, all the DNA derived from oral microbiome species is being discarded leaving no information to differentiate the sample type. We demonstrated that the change in the model performances was not due to the removal of some samples (Supplementary Fig. 9) nor that a random subsampling of the reads to the median number of *Streptomyces* reads would lead to similar results (Supplementary Fig. 10), indicating that the selection of *Streptomyces-*specific reads was the reason behind the observed differences in prediction performances.

When comparing the performance of logistic regression with that of random forest classifiers in the case where only *Streptomyces* data were used, we observed that logistic regression led to better performances for samples' geographic origin prediction (Supplementary Fig. 8). Interestingly, for both the methodologies, the use of *Streptomyces* data mitigated the bias introduced by the extraction protocols and sample type as the prediction accuracy of these two variables were reduced (Supplementary Fig. 8).

When trying to classify the two Brazilians with Polynesian ancestry using the model trained on *Streptomyces* reads only, we retrieved a less confident classification as Brazilians compared to the model trained on whole metagenomes data. Specifically, one of the two individuals (Bot17) had ~ 67% probability of being Brazilian while the other (Bot15) had only ~ 51% probability of being a Brazilian, indicating that, for the latter, the two tested locations had similar probability.

## Discussion

Most of the DNA sequenced from ancient remains is very often of microbial origin^6^. These data are usually discarded by ancient human DNA studies by isolating the reads that map to the human reference genome. In recent years, the study of these ancient metagenomes has allowed the recovery of DNA from human-associated microorganisms (such as human-infecting pathogens^4^ or human associated microbial communities^53^) and the identification of DNA from plants and animals that were part of the diet of ancient humans^54,55^. Depending on the type of sample targeted for aDNA extraction and sequencing, different metagenomes can be obtained. For example, dental calculus has been shown to contain precious information regarding the human oral microbiome and the human diet as its progressive calcification throughout the life of the individuals traps and preserves DNA from such sources^55–57^. Highly vascularized tissues, such as the tooth pulp chamber, are optimal sources to access DNA from blood-borne pathogens^58^. Other samples, such as the petrous bone and tooth dentine or cementum, tend to harbor less human-associated microorganisms' DNA^57,59^. In these samples, most of the microbial DNA is likely derived from the surrounding soil. Here, we showed, in agreement with previous observations^41^, that the metagenome extracted from ancient bones and teeth dentine/cementum is enriched in soil-derived microorganisms that have colonized the remains after the death of the individual (Fig. 1). Furthermore, we show that the metagenomic reads successfully blasted by MetaDamage display the classical aDNA damage patterns in line with the fact that the samples were unearthed a long time ago and, afterwards, stored in a museum (Supplementary Fig. 3). However, contamination of the sample from soil-dwelling microorganism can occur at any time point during the period the sample was buried. As such, the presence of aDNA damage patterns might not be observed in metagenomes from recently excavated remains where present-day microbes can also infiltrate the remains^49^.

We hypothesized that these metagenomic data contain information about the place of death of ancient individuals. We then proposed to use this information to locate burials and solve cases of sample mislabeling.

To test our hypothesis, we presented a simple comparative framework that takes advantage of reference panels of ancient metagenomic samples from different locations to identify the origin of target samples. The method we propose uses a k-mer-based approach to compute between-sample metagenome similarities and then perform dimensionality reduction to generate independent input features to train models such as logistic regression or random forest for binary classification.

We trained and tested the method with samples from four different geographic locations that were assembled into two binary datasets (i.e., each one including samples form two geographic locations). We then trained a model to predict the samples' geographic origin and assessed the accuracy of the method with a jackknife approach. One dataset included samples from Denmark and England^33^. These individual samples were processed together with the same laboratory protocols, allowing us to test the model on an ideal setting, minimizing the risk of batch effects (i.e. samples processed together and/or with the same protocols being more similar). This is however an unlikely scenario, as most dataset generated in different studies are not processed using the exact same protocols. Therefore, meta-analyses of metagenomic data are always prone to laboratory-induced biases. To assess the effect of different extraction protocols on our method we incorporated another dataset that included deeply sequenced samples from Brazil and Polynesia in which samples were subjected to different extraction and library preparation protocols. The extraction protocol variable was partially, but not perfectly, correlated with the sample geographic origin. This allowed us to assess how the model would perform in predicting the two variables.

We demonstrated that, for both the test dataset, the model can predict with good accuracy the geographic origin of a sample excluded from the model training (Fig. 2e). This suggests that geographic-dependent similarities in the soil microbiome translate into similarities in the ancient bone/teeth metagenomes that can be detected even after long-term storage in a museum. Furthermore, we show that k-mer-based approaches are appropriate; they can reveal these similarities and be employed to devise models for ancient sample geographic identification when reference samples for a given location are available (Fig. 2e).

Interestingly, we find that individual samples from different locations but stored in the same museum are not assigned to the same location by the model. This indicates that the long-term museum storage experienced by these samples did not play a major role in determining the composition of the sequenced metagenome or that the methodological procedures readily controlled for such a bias. However, it is worth noting that we examined a limited number of individuals and a single museum. Further analyses will probably be needed to thoroughly exclude the possibility of a museum storage bias.

While sample metagenomes harbor a wealth of information, they are prone to batch effects due to laboratory procedures^60^ and/or contamination biases^51^. This is a major caveat for analysis interested in reusing and merging data from multiple studies. In our study we demonstrate that the model could predict the extraction protocol used for the Brazil-Polynesia dataset (Fig. 3c). The extraction protocol and geographic location variables are partially correlated in the Brazil-Polynesia dataset (Fig. 3a). Interestingly, we note that the geographic location is more accurately classified than the extraction protocol, suggesting that the extraction protocol predictions could be partially driven by the correlated variable “geographic origin”. However, in this case, an extraction protocol bias cannot be fully excluded. We argue that the use of reads from specific microbial genera, not listed among the usual laboratory contaminants, could provide an unbiased source of information. While different extraction protocols can lead to different extraction efficiencies between major groups of bacteria (e.g. shifts in gram positive vs gram negative relative abundance^61^), we expect little compositional bias within the single genera.

While not much research has been conducted to study the biogeography of single soil-dwelling bacterial genera, some studies have demonstrated the existence of phylogeographic patterns and isolation by distance processes within some terrestrial bacteria genera and species^50,62, 63^. This raises the possibility of using sequencing data coming from specific ubiquitous and abundant genera as an alternative to whole metagenomes.

Here we report the presence of *Streptomyces* genus in all our samples. The data we analyzed come from four different studies with various protocols and storage conditions, so the prevalence of *Streptomyces* bacteria is likely common in the bone necrobiome community. For instance, the Brazil-Polynesia dataset ~ 82% of the samples (32 out of 39) had > 100.000 reads classified to the *Streptomyces* genus. We used these samples to train a model to predict sample location with even higher accuracy than when using whole metagenomes, showing that the same approach we developed for whole metagenomes can be devised on a specific subset of reads (Fig. 4 and Supplementary Fig. 10). Interestingly, we also show that using only *Streptomyces*-classified reads reduces the risk of extraction protocol biases compared to the whole metagenomes analysis as we register a reduction in its classification accuracy (Fig. 4 and Supplementary Fig. 10), indicating that the *Streptomyces* genus can be a valuable biomarker for ancient samples geographical tracing in meta-analyses.

Another highly represented genus in our datasets was *Pseudomonas*. However, this genus contains known laboratory reagents contaminant species^51^ that could lead to a contamination-dependent bias when comparing samples from different batches or from different studies. Hence, we refrained from applying our logit model on this specific genus.

We then applied our method to a real case scenario and predicted the geographic origin of two Brazilian individuals with Polynesian ancestry^30^ to ponder the likeness of a museum mis-labeling. When using whole metagenomes, our method found no evidence for a Polynesian origin of the samples as they are classified as Brazilian with higher probability. However, the model trained on *Streptomyces* data only, provides a less confident classification compared to the whole metagenome analysis. The probability of being Brazilian is reduced from > 0.99 to ~ 0.67 for one individual (Bot17), and from ~ 0.80 to only 0.52 for the other (Bot15), indicating that the latter has almost the same probability of being Brazilian as it has of being Polynesian, cautioning the interpretation of the results based on whole metagenomes.

As the method relies on reference panels for the classification, it is essential that the individual samples used to train the model exemplify the general expected necrobiome composition for a given location. In the real case scenario, it is unclear where in Brazil or Polynesia one should look for as the samples have been assigned a Polynesian origin in terms of genetic ancestry and a very general sampling location in Brazil (Minas Gerais state). Therefore, we cannot exclude the existence of other locations that would serve as better geographical proxies for the origin of the two investigated individuals.

Furthermore, we observe that it is useful to have a large number of metagenomic reads to be able to have a good classification accuracy. We observed a reduction in the logit classification performance when samples with a low number of metagenomic reads (< 1 million)—after preprocessing—are included (Fig. 2e). These samples apparently do not contain enough information to be correctly classified. Therefore, we discourage applying this method on too shallow datasets. Having enough data (samples in the reference panel and sequencing data) is therefore of importance to apply our method.

For the Brazil-Polynesia datasets we also show that our method can predict other variables. The sample type (bone vs tooth) is among the variables that can be predicted, even though it provides less confident classifications compared to geography. This could be explained by a colonization/contamination from oral microbiome species in teeth samples as opposed to bone samples. When using *Streptomyces* only reads the sample type classification accuracy is strongly reduced. This indicates that our method, while suited for both kinds of samples tested, could be subject to sample type biases due to oral microbiome contamination when applied to dataset including ancient teeth whole metagenomes. Approaches that could identify and remove reads (or k-mers) from other non-soil environments (e.g., DNA from oral microbiome species) could help to control for sample type induced biases.

Current methods of ancient sample geographic prediction based on metagenomics data will face challenges due to the low number of dataset available, as most of the metagenomic data generated during aDNA studies are not made readily available on public databases. This was a limitation in this study as well. We were indeed not able to conduct analysis using large reference datasets. We believe that sharing *all* the raw sequencing data is a really important step that should become the norm in ancient DNA research. Currently, most studies only share the subset of reads that map to the human genome, rather than the entire metagenomes (raw FASTQ files). This approach not only introduces biases in the human data—as they are affected by the mapping algorithm used—but also prevents the sharing of metagenomic data, which we have shown to contain valuable information, for instance about the necrobiome community. In our specific case, if more datasets from different locations were made available, this would allow for a better description of these communities and their variability in space and across different environments. This could potentially lead to the development of geographic origin prediction methods with increased power and versatility.

A major challenge for such meta-analysis will be to account and control for procedures-derived biases. Sequencing of extraction and library negative controls is a standard procedure in modern DNA microbiome studies and, if adopted also for aDNA sequencing experiment, could help to better control for the presence of laboratory contaminants. Moreover, a better understanding of the bone necrobiome community composition could allow us to identify other microbial biomarkers that can be used for geographical tracing of bone samples.

In this study, we evaluated the method using 90 samples from four distinct geographical locations. In some ways, this is a small dataset, and we cannot make general statements about which classification methods (e.g. logit or random forest) or parameters (e.g. k-mer length) is most appropriate for a new dataset, nor can we assess the degree of generalizability of our results or the behavior of our method when samples from more or less distant locations are included. Further studies will be needed to ultimately assess the general applicability and robustness of the proposed method at different locations and geographic scales. Another limitation of the present study is the absence of environmental metadata, such as information regarding the climate and the soil type in which the remains were buried. This prevented us from assessing the potential impact of such external factors on the inter-sample similarities and the classifiers performance. Another interesting aspect that we could not probe with the analyzed datasets is the effect of temporal variation on such metagenomes. Temporally stratified data and data from specific climates or soil types could help to elucidate the effect of time and environment on the microbial composition and on the classification methods performance.

To conclude, our study provides a proof of concept that shows how metagenomes from ancient samples can be used to trace the sample geographic origin when reference panels are available. This offers potential to solve major challenges in the archaeogenetics field, such as identifying the place of death of an individual, pinpointing museum mislabel cases and hence providing valuable information for repatriation decisions.

## Methods

### Description of the datasets used in this study

We tested our methodology using previously generated datasets, including samples from five different locations: Brazil, Polynesia, Denmark, England, and Argentina (Table 1).

**Table 1:** Samples included in the study.

#n of samples	Location	Samples age range	Median number of reads
24	Brazil	18th–19th century	3.2 × 10^8^
15	Polynesia	19th century	1.8 × 10^9^
2	Brazil (?)	17th century	3.9 × 10^9^
3	Argentina	18th century	1.3 × 10^7^
29	Denmark	11th–19th century	4 × 10^6^
22	England	11th–19th century	4 × 10^6^

We used these data to compile two binary datasets to be used for model training and testing: The first dataset (referred to as “Brazil-Polynesia dataset”) included individual samples from Brazil (n = 24)^31^ with age ranging from the 1st to the  19th centuries CE, but with most of the radiocarbon dated samples (n = 22) being from the 18th and 19th  n centuries (n = 19), and from Polynesia (n = 15) dated to the 19th century.^32^ All the individual samples in this dataset were deeply sequenced with a median number of ~ 5.55 × 10^8^ reads per sample. The second dataset (referred to as “Denmark-England dataset”) included samples from Denmark (n = 29) and samples from England (n = 22)^33^ dating from the 11th to the 19th century. This dataset was subjected to a shallower sequencing. The median number of reads was 4 × 10^6^ per sample. The two datasets included both bone and tooth samples. For the tooth samples, DNA was extracted either from the dentine or from cementum. For the Brazil-Polynesia dataset two different extraction protocols were used^34,35^. For further information about the two datasets used for the model training and testing in this study see Supplementary file 2 and 3. To inspect the effect of long-term museum storage we used the model trained on the Brazil-Polynesia dataset to classify three Fuego-Patagonians individuals (Argentina) from the 18th century whose remains were stored in the same Museum as the Polynesians in the reference dataset (Musée de l’Homme—Paris, France)^48^.

Finally, we applied one of our models to a case of potential museum mislabeling. We used the model trained on the Brazil-Polynesia dataset to classify two 17th century individual samples from Brazil (according to the museum label) that were found to be of Polynesian ancestry^30^.

### Removal of human DNA and lab contaminants

All the samples were preprocessed through the custom pipeline implemented in snakemake^64^.

The first steps of the analysis included multiple mappings against the human and the bacteriophage Phi X 174 (used as control for Illumina sequencers) reference genomes to filter out reads of potentially human origin and artificial DNA fragments introduced at the sequencing step. FASTQ files were initially processed using Mapache pipeline (version 0.3.0)^65^ to map against the human reference GRCh38 assembly using default parameters and bwa aln (v0.7.17)^66^ as mapping tool with seed disabled (-l 2024).

When possible, reads were collapsed with AdapterRemoval ver. 2.3.2^67^. The collapsing step is expected to reduce the risk of modern DNA contamination by removing longer—and hence likely modern—molecules. Unmapped reads were saved in a separate bam file which was reconverted into a FASTQ with samtools^68^ (v1.9) “fastq” command and low complexity regions were filtered out with komplexity^69^ (v0.3.6) using a threshold value of 0.7. The complexity filtered reads were then mapped a second time to the human reference with bwa mem^66^ (default parameters) to filter out as many human reads as possible. Unmapped reads were extracted from the bam file using samtools^68^ view -b -f 4 command and converted to FASTQ as described above. Reads were then mapped against the Escherichia phage Phi X 174 reference genome (NC_001422.1), commonly used as control in Illumina sequencing experiments^70^, with bwa mem^66^ and all the unmapped reads were recovered and converted into FASTQ with samtools^68^ as described above. FastQC^71^ v0.11.9 was run at each filtering step.

To reduce the risk of modern DNA contamination biases, reads longer than 80 bp were discarded from the subsequent analysis using seqkit^72^ seq v2.2.0. Reads shorter than 30 bp were also removed as short reads are hard to confidently classify or map. aDNA damage patterns were inspected both before and after filtering for fragment length with MetaDamage^47^, a newly developed tool that specifically inspect deamination patterns in metagenomic samples, after subsampling each sample to 100.000 reads (for speed purposes) with seqtk-1.3 “sample” command (r106) (https://github.com/lh3/seqtk). As the end of the reads are usually affected by aDNA damage patterns, we trimmed 4 bp from each end of the read using the trimfq command from seqtk-1.3 (r106). For the Denmark-England dataset we repeated the analysis twice to test the effect of shallow depth sequencing: once including all the samples and once discarding the samples with less than one million reads. Sample MN0008 from Brazil-Polynesia dataset was discarded from the subsequent analysis because the data contained adapters even after adapter removal and no clear sign of aDNA damage.

### Reads classification and compositional analysis

We compared the microbial composition of the ancient bones and teeth samples with the microbiome found in other environments to understand the likely sources of the bone necrobiome. To do so, we downloaded previously published shotgun metagenomics data from four different terrestrial and host-associated environments which could serve as sources of microorganisms, including soil^37^ (encompassing two different soil biomes: grassland and forest), human oral cavity (plaques and tongue swab samples)^38^, human skin^39^ (palm swab samples), human gut^40^ (feces samples), together with other ancient bones^45,46^ (Supplementary file 4). To identify the dataset to be used for this analysis we took advantage of previously published resources such as AncientMetagenomeDir^73^ and TerrestrialMetagenomeDB^74^. These resources provide metadata-curated collections of metagenomes from ancient samples and terrestrial environments respectively.

Samples from these five sources were processed through the same preprocessing pipeline described above.

For all the preprocessed metagenomes, the remaining non-human reads were classified with Centrifuge^75^ against the bacterial RefSeq genome database plus the human reference. A kraken-style report was then generated and converted into a biom-table with kraken-biom^76^ specifying the maximum and minimum operational taxonomic unit (OTU) level to genus and species: –max G –min S. This table was imported in R (version 4.1.1)^77^ for the compositional analysis. We filtered out *Homo sapiens* (taxonID = 9606) and all the low abundant taxa (< 10 reads in all analyzed samples). We collected full taxonomic information for each taxonID using taxonomizr (version 0.9.3)^78^. The genome size information for all available bacteria was downloaded from NCBI while filtering for completeness and including only complete genome assemblies (https://www.ncbi.nlm.nih.gov/genome/browse#!/prokaryotes/, accession date: 05/04/2022). We computed species and genus level average genome sizes and used this information to normalize each taxon to its average genome size. Taxa lacking genome size information or with poor taxonomic characterization were removed. Samples were then rarefied to the lowest sample depth using GUniFrac (version 1.6)^79^. Distance matrices were computed with vegan (version 2.6-2)^80^ vegdist function using Jaccard distance metric and dimensionality reduction was performed using prcomp R function and plotted using autoplot function with ggplot2 (version 3.3.6)^81^ and ggfortify (version 0.4.14)^82^ libraries. Compositional stacked barplot for the Brazil-Polynesia dataset and the Denmark-England dataset were created with reshape2 (version 1.4.4)^83^, ggplot2^81^ and RColorBrewer (version 1.1-3)^84^ and the aid of other R packages, including dplyr (version 1.0.9)^85^, tidyr (version 1.2.0)^86^ and tidyverse (version 1.3.2)^87^.

### Microbial source tracking with SourceTracker2

We then predicted the potential sources for the microorganisms present in the ancient bones and teeth metagenomes with SourceTracker2^44^ using the same samples described above as sources and our two datasets (see above) as sinks (Supplementary file 5). In addition, bone samples from Campana et al.^46^ and Willmann et al.^45^ were used to model the bone necrobiome specific composition. SourceTracker2 was then run with the biom-table previously computed by kraken-biom^76^ and results were plotted in R^77^ using ggplot2 (version 3.3.6)^81^ and reshape (version 0.8.9)^83^ packages.

### K-mer based similarity score computation

We aimed at developing a method to discriminate samples from two different geographic locations that took into account inter-sample metagenome similarities. In this study we relied on a fast and powerful k-mer-based approach. We analyzed the metagenomics reads left from the preprocessing pipeline with sourmash^26^. Sourmash computes k-mers sketches (hashed versions of the k-mers) and inter-samples k-mer-based similarity scores. We used the following command to compute k-mer sketches (also called signatures):

sourmash sketch dna -p k = 21,abund "$filename" --output "$filename".sig

Where the parameter k defines the desired kmer length (here 21 bp) and "abund" parameter allows us to compute abundance-weighted sketches. While most of the results presented in this study were obtained using a k-mer length of 21 bp as a compromise between specificity and sensitivity, we have assessed the effect of k-mer size choice on the results by repeating the analysis for five different k-mers sizes: 11 bp, 15 bp, 21 bp, 25 bp, 31 bp. Longer k-mers increase the specificity but reduce the sensitivity as fewer shared k-mers are detected. Conversely, short k-mers increase the sensitivity but lose specificity by losing taxonomic information.

A matrix of samples angular similarities was then computed and saved into a csv file with the following command:

sourmash compare *.sig --csv matrix.csv

The similarity matrix produced by sourmash was imported in R (version 4.1.1)^77^.

### Model to identify the sample geographic origin

We took the k-mer-based similarity matrix as input to train a model that performed sample geographic origin identification. Logistic regression (logit) is a powerful statistical approach for binary classification problems that is robust to deviation from normality and homoscedasticity of the input data. The result of a binary logistic regression classification is a probability of being assigned to one or the other class. As logit requires independent input features, we trained a binary logit to predict the variable "geography" using the principal coordinates obtained with dimensionality reduction.

Dimensionality reduction was performed using the “prcomp” R function. Samples coordinates for each dimension were extracted and used to train a logistic regression for binary classification. Given the low number of samples available (a common feature in aDNA studies) we decided to use a penalized logistic regression with lasso regularization with “glmnet” R package (version 4.1-4)^88^ to reduce overfitting problems. glmnet alpha value was set to 1 to use lasso regularization and the best lamba value was selected using cross-validation. Lasso regularization allows for the automatic selection of the relevant coordinates taken in input by the model. As an alternative option to logistic regression, we trained a random forest model using randomForest^89^ R package.

We independently tested the method on the two datasets described above: the Brazil-Polynesia dataset and the Denmark-England dataset. Target individuals (Brazilians with Polynesian ancestry and Fuego-Patagonians) were excluded from the model training and testing. We assessed the accuracy of the classifiers in predicting the variable “geography” using leave-one-out cross validation (jackknifing). After dimensionality reduction we trained the model n times (with n equal to the number of samples in the dataset), each time one sample was excluded from the model training and used for model testing. We compared the models’ predictions to the geographic origin reported in the studies to get classification accuracy scores. We evaluated the models’ performance by computing the proportion of samples misclassified and by assessing how confident the classifier was (i.e., with which probability each sample was assigned to its correct class). We then plotted accuracy curves with the proportion of samples correctly classified above any given probability using ggplot2 (version 3.3.6)^81^.

For the Brazil-Polynesia dataset we also trained and tested the model classification accuracy for other variables including the sample type (bone vs tooth) and the extraction protocol used.

We compared the performance of the logistic regression models with the random forest models using ROC curves.

Next, we assessed the effect of long-tem museum storage and classified three Fuego-Patagonians individuals whose remains were stored in the same Museum as the Polynesians (Musée de l’Homme—Paris, France)^48^. We then applied the model to a real case scenario and analyzed two 17th century individual samples from Brazil with Polynesian ancestry (see above). Classification of these individuals of interest was achieved by performing a MDS of each target individual together with the reference samples (Brazil-Polynesia dataset). The logit model was trained on the coordinates of all the available reference individuals to the exclusion of the target sample. The trained model was then used for predicting the geographic origin of the individual of interest using the “predict” R function. We repeated this procedure for each one of the target samples separately to obtain their classification probabilities.

### Model to identify the sample geographic origin using *Streptomyces* reads

To test the possibility of using *Streptomyces* as a biomarker to trace sample geographic origin we trained another logit model after isolating reads from the *Streptomyces* genus. All the reads classified as part of the *Streptomyces* genus by KrakenUniq^90^ against the RefSeq database of prokaryotic genomes (plus *Homo sapiens*), were isolated using KrakenTools^91^ (cloned from github: https://github.com/jenniferlu717/KrakenTools/#extract_kraken_readspy on date 10/01/2023). aDNA damage patterns for *Streptomyces* classified reads were inspected with MetaDamge^47^ after subsampling each sample to 10.000 reads with seqtk-1.3 “sample” command (r106) (https://github.com/lh3/seqtk). K-mer sketches and a similarity matrix were computed on the *Streptomyces* reads with sourmash^26^ as described above. The similarity matrix was then imported into R^77^. Samples with less than 100.000 reads classified as *Streptomyces* were removed. All the subsequent steps, including logit model training, accuracy assessment, target sample predictions and data plotting were done as described above with the same R packages.

To test the effect of sample removal we repeated the same analysis for the whole metagenomes while removing the same samples removed here (i.e., those with less than 100.000 reads classified as *Streptomyces*). Then, to test the effect of subsampling we repeated the analysis after randomly subsampling each sample to the median number of *Streptomyces* reads in the dataset (661.375 reads) with seqtk-1.3 (r106) (https://github.com/lh3/seqtk) "sample" command.

## Supplementary Information

Supplementary Information 1.

Supplementary Information 2.

Supplementary Information 3.

Supplementary Information 4.

Supplementary Information 5.

Supplementary Information 6.

Supplementary Information 7.

Supplementary Information 8.

Supplementary Information 9.

## Data Availability

The raw data for the datasets from Denmark and England have been previously published^33^ and were downloaded from NCBI (Accession number: PRJEB18722). NCBI accessions for the individual samples are provided in Supplementary file 6. The Brazilian dataset was generated as part of another study^31^ (see the related study for details regarding the data). The related non-human metagenomic data are made available as part of the current study following the Fort Lauderdale Agreement principles^92^ (Accession number: PRJEB67998). We ask users to observe these principles for this dataset, which entitles the data producers to make the first presentation and publish the first analysis of specific microbial genomes/genes present in that dataset (see the related bioRxiv reference for some results: Cruz Dávalos, D. I. et al.^31^. Data from the Polynesian dataset was generated as part of this study^32^. As detailed in the related publication^32^, “following consultation with the Comisión Asesora de Monumentos Nacionales (CAMN) in Rapa Nui, [the human and non-human Ancient Polynesian] sequencing data will not be deposited in a public repository and will be made available upon request to J.V.M.-M. and A.-S.M. Access requests will be managed jointly with CAMN representatives. Ancient [Polynesian] sequencing data are available for population history research and not for public posting, medical research or commercial purposes.”. The code for the preprocessing pipeline in snakemake is provided in Supplementary file 7. The R code used for the analysis is provided as an R Markdown^93^ report (Supplementary file 8).
