# Supplementary material for: Towards predicting the geographical origin of ancient samples with metagenomic data
Source: Sci Rep. 2024 Sep 18;14:21794. doi: 10.1038/s41598-023-40246-x (PMC11411106; doi:10.1038/s41598-023-40246-x)
Supplement: Supplementary file 1 — Supplementary Information 1. [file 41598_2023_40246_MOESM1_ESM.docx]

**Supplementary file 1 - Supplementary figures:**


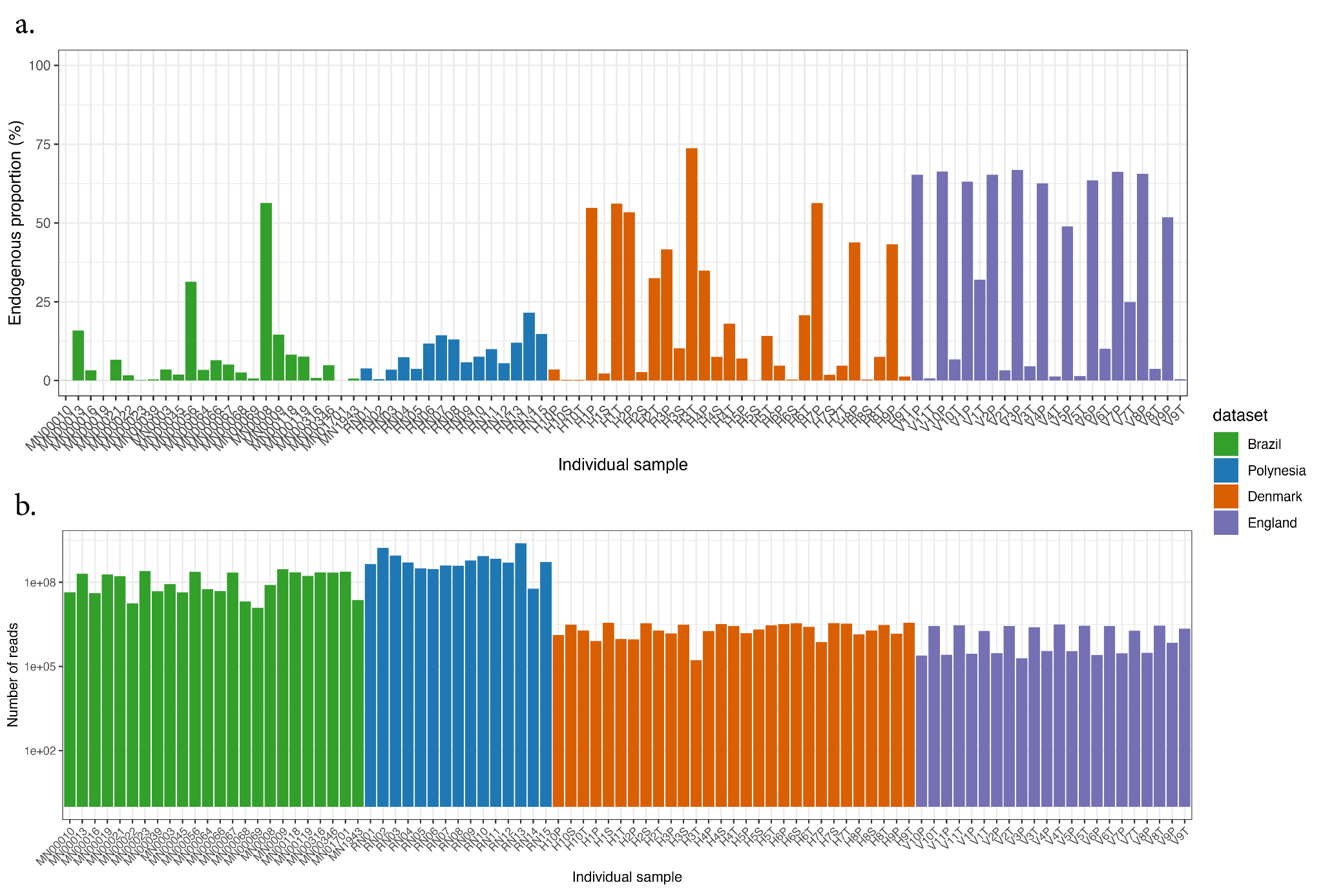


**Supplementary Figure 1. Human DNA endogenous proportion in the samples and number of reads used in this study.** (a) Human DNA endogenous proportions in the samples analyzed in this study and (b) number of reads kept after pre-processing (see methods for details). Note the logarithmic scale on the y axis for panels b.


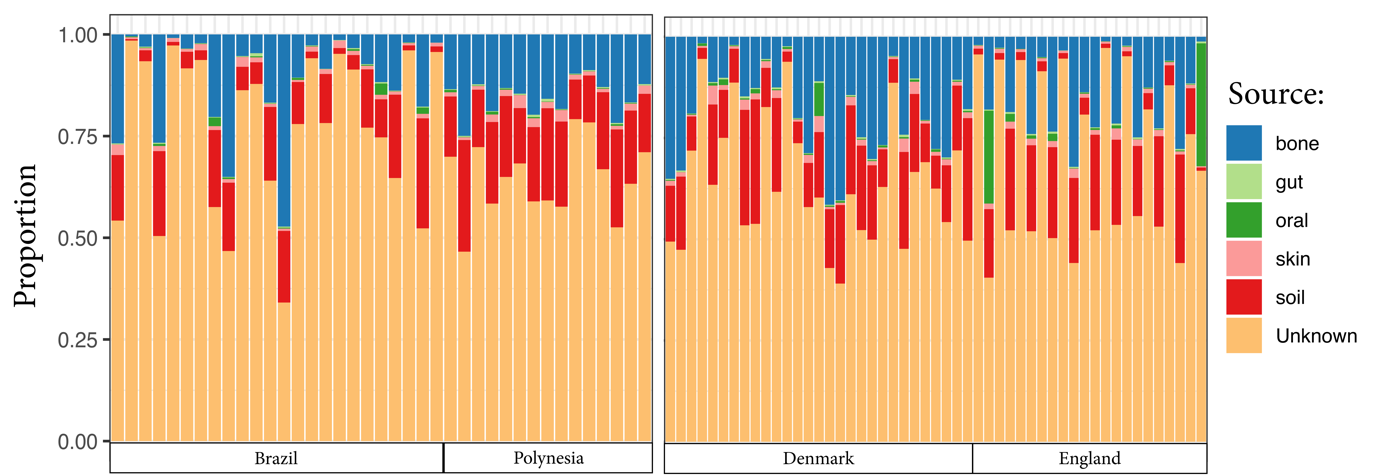


**Supplementary figure 2. Contribution of different sources to the ancient samples’ metagenome composition.** For each sample included in the analysis, SourceTracker2 was used to infer the contribution of different sources to the samples’ taxonomic composition. In most cases, a source could not be determined (orange). This is probably due to the scarcity of the reference panel. However, and as expected, among known sources, soil, and bone were the most common sources. Brazil-Polynesia dataset (left) and the Denmark-England dataset (right).


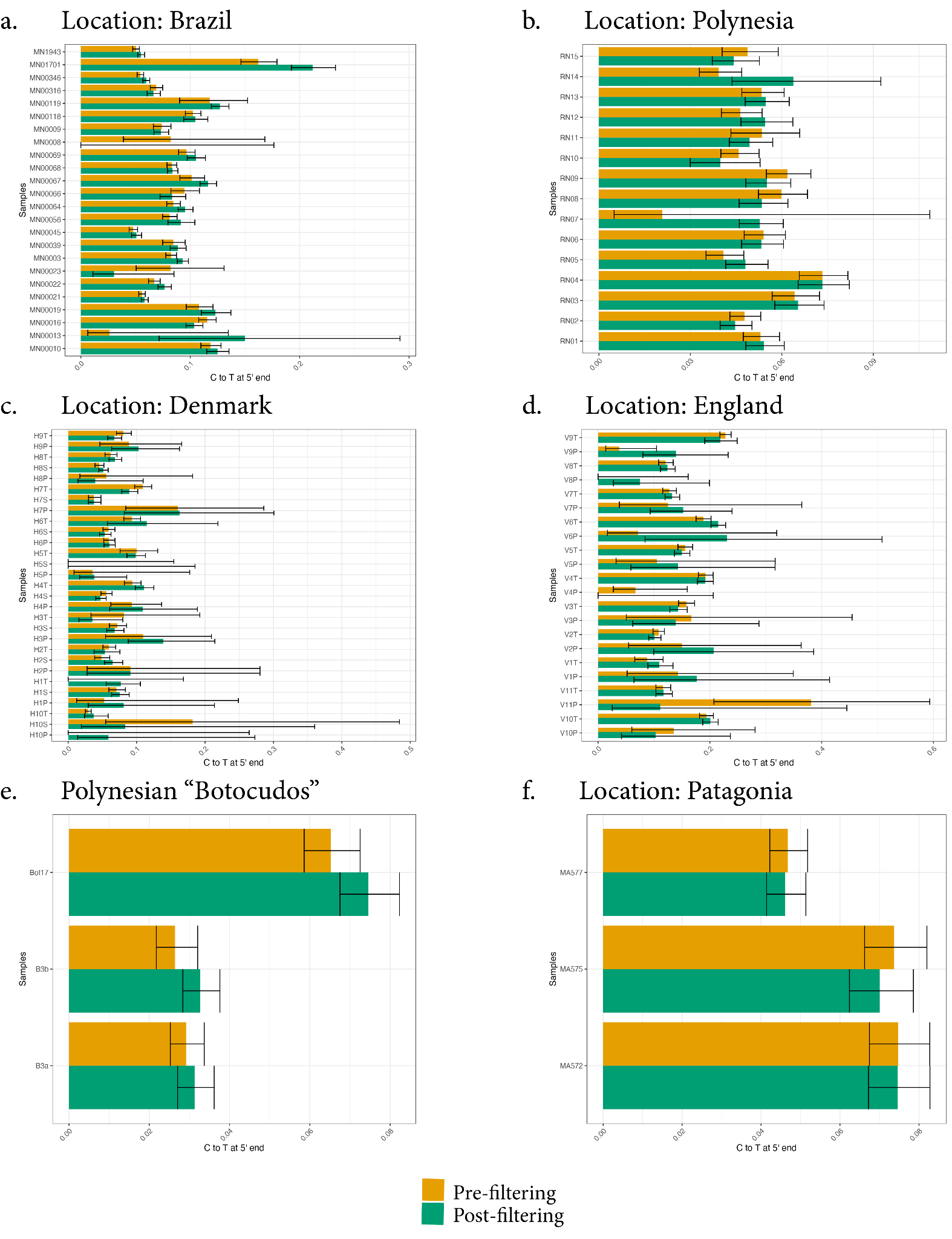


**Supplementary Figure 3. aDNA damage assessment.** The barplots show the amount of damage (computed as C to T transition at the last base at the 5') with confidence intervals for the individual samples used in this study and coming from different locations. The damage was computed from sub-sampled metagenomic data (100.000 reads per sample) with MetaDamage. Overall we observe a clear damage signal for almost all of the analyzed samples. The removal of reads longer than 80bp does not significantly increase the amount of damage detected.


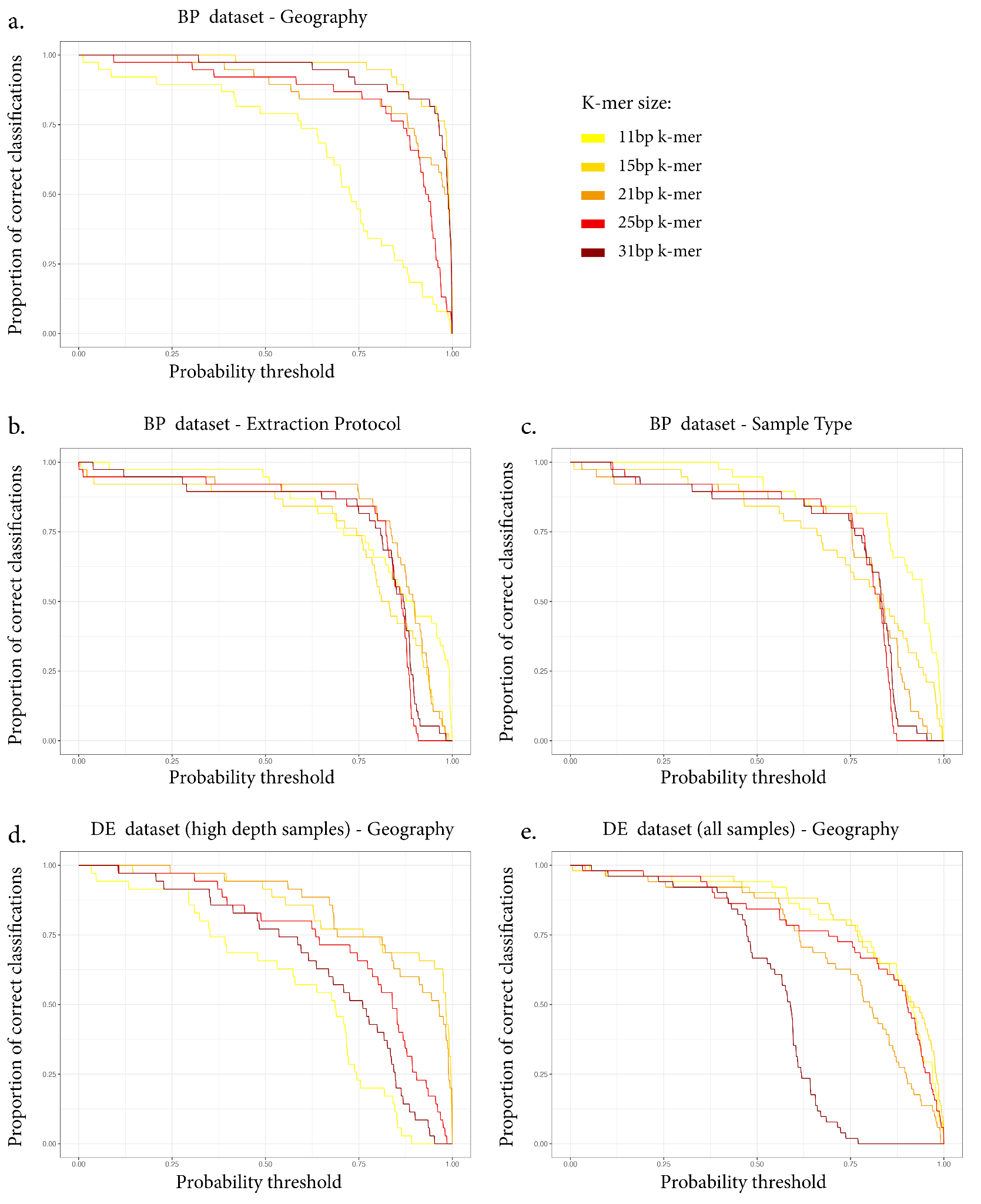


**Supplementary Figure 4. Logistic Regression prediction performances with different k-mer sizes.** Curves are shown for the Brazil-Polynesia (BP) dataset and the variables "geography" (a), "extraction protocol" (b) and "sample type" (c), and for the variables "geography" in the Denmark-England (DE) dataset when using only high depth (> 1 million reads) samples (d) or when using all the available samples in the dataset (e). The highest variability is observed in the prediction of the variable "geography" with either the shortest (11bp) or longest (31bp) tested k-mer being the worst performing one.


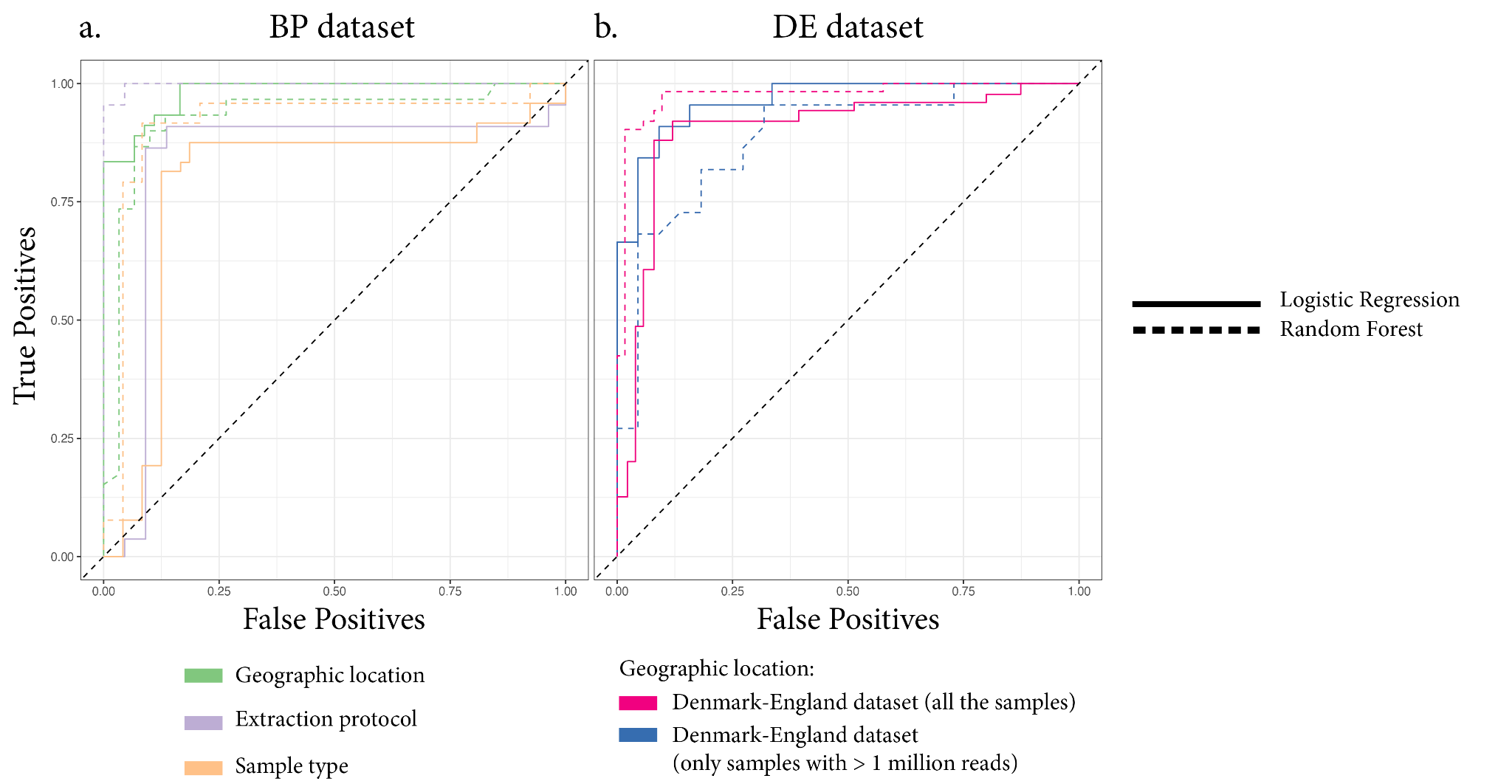


**Supplementary Figure 5. Logistic regression and random forest classifiers performances comparison.** ROC curves comparing the performances of logistic regression classifiers (continuous lines) and the performances of random forest classifiers (dashed lines) for different variables (see color code) when using whole metagenomic data for the Brazil-Polynesia (BP) dataset (a) and the Denmark-England (DE) dataset (b). The two methods perform similarly. Logistic regression usually outperforms random forest classifiers for samples' geographic origin prediction, except in the case of the Denmark-England dataset when also low depth samples were included.


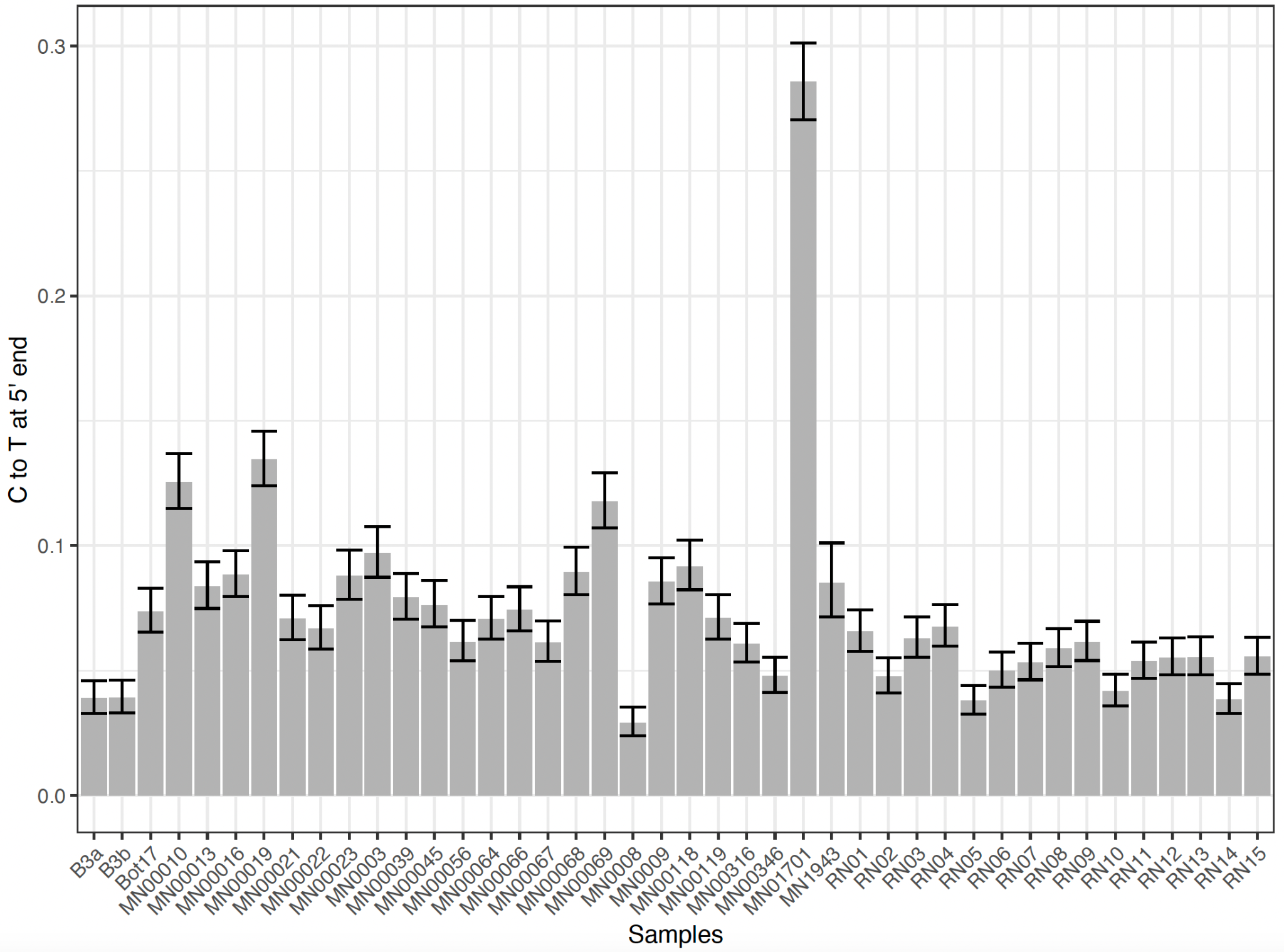


**Supplementary Figure 6. aDNA damage assessment for the Streptomyces-classified reads.** The barplots show the amount of damage (computed as C to T transition at the last base at the 5') with confidence intervals. The damage was computed from sub-sampled data (10.000 reads per sample) with MetaDamage. Streptomyces data show clear damage patterns in all the samples. Interestingly MN01701 shows much more damage than the other samples. This sample is also much older (~2000 years BP) than all the others included in this study that are mostly from the 18th-19th centuries.

**
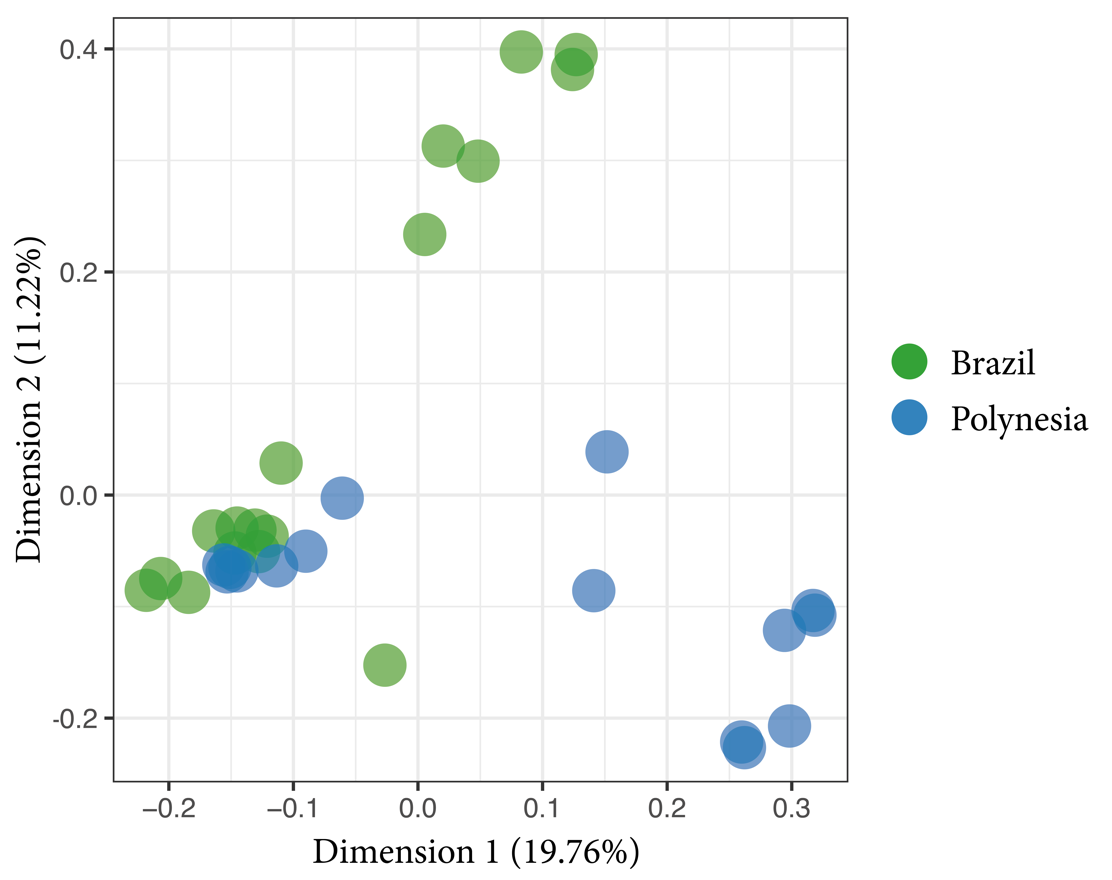
**

**Supplementary Figure 7. MDS plot on the similarity matrix computed using only reads classified as *Streptomyces*.** When using only reads from the genus *Streptomyces,* we still observe patterns of sample separation according to geography on the MDS plot. Here we show dimension 1 and 2 for the Brazil-Polynesia dataset where a good separation of the samples according to the geographic location is achieved.


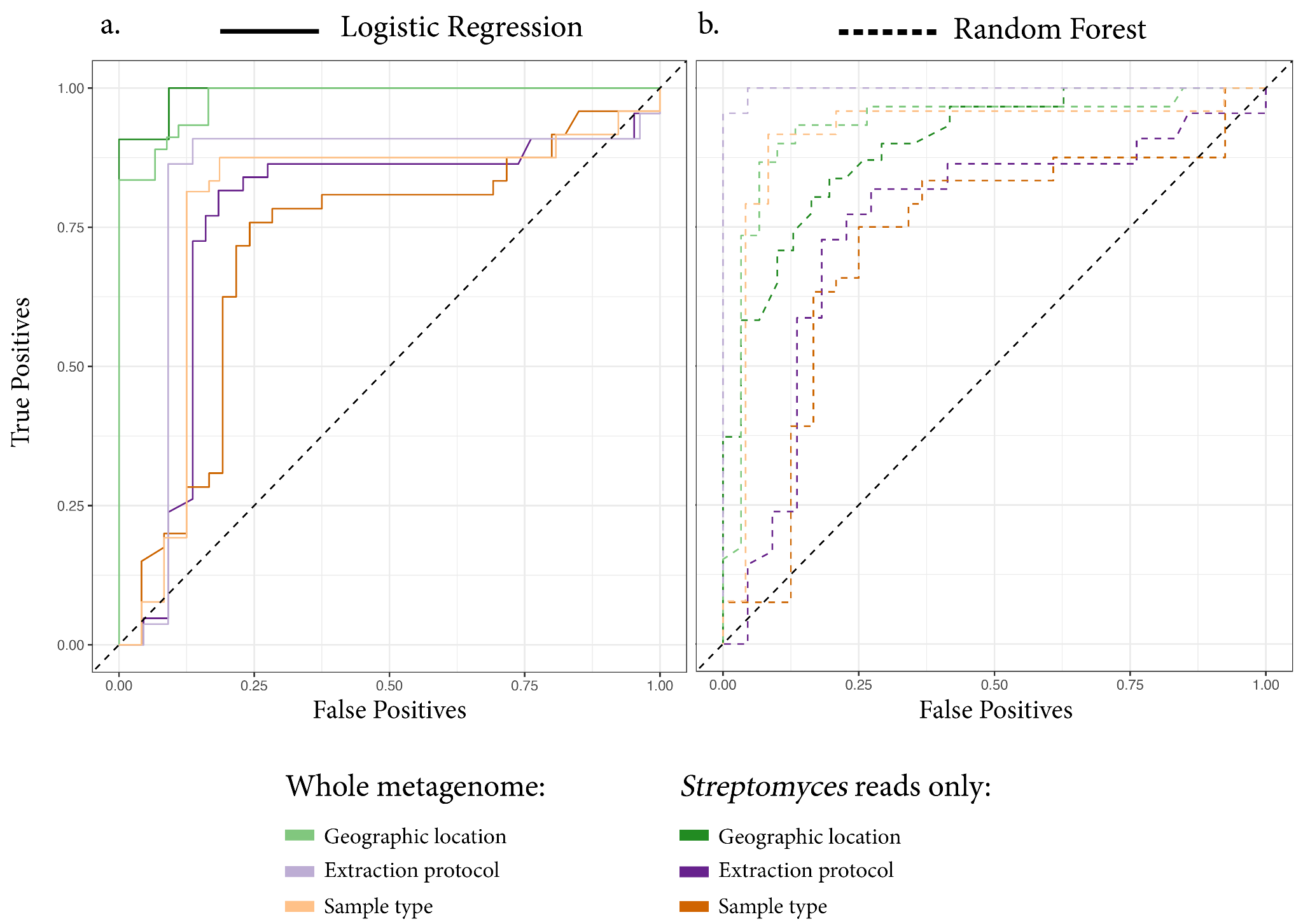


**Supplementary Figure 8. Performance of the classifiers when using whole metagenomic data or Streptomyces reads only.** (a) ROC curve comparing the performances of logistic regression classifiers when trained on whole metagenomic data (light hue) or Streptomyces reads only (dark hue). We observe similar performances in the two cases for the variable "geography" with Streptomyces data leading to slightly better performances. On the contrary, a logistic regression classifier trained on Streptomyces data leads to worse performances in the prediction of other variables such as the extraction protocol and the sample type. (b) ROC curve comparing the performances of random forest classifiers when trained on whole metagenomic data (light hue) or Streptomyces reads only (dark hue). The usage of Streptomyces data only reduces the performance of the random forest classifier for all the tested variables but affecting mostly the prediction for the extraction protocol variable. When using Streptomyces data, logistic regression led to better performances than random forest for the geography origin prediction.


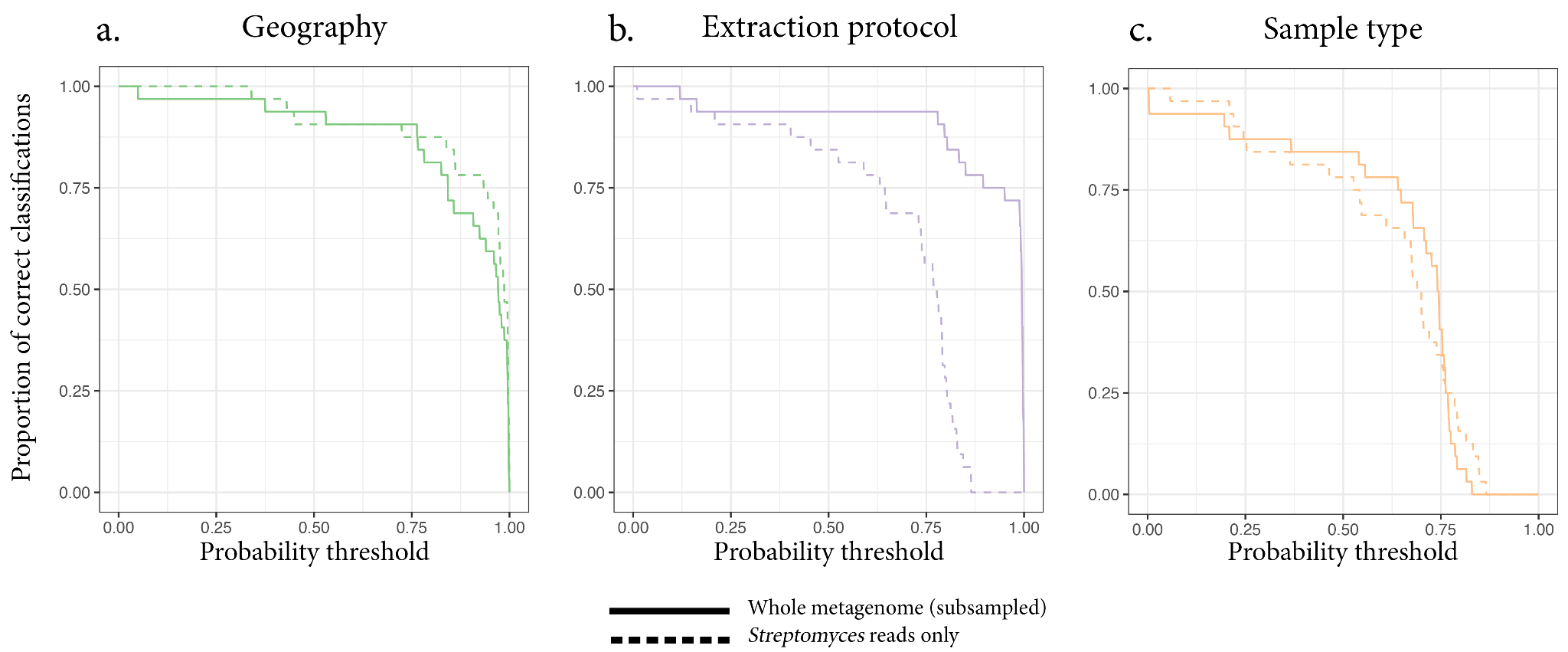


**Supplementary Figure 9. Comparison of the method prediction accuracy when using whole metagenomes or *Streptomyces* reads only and the exact same samples as input.** Accuracy assessment for geographic origin prediction in the Brazil-Polynesia dataset when using whole metagenomes (continuous lines) and when using *Streptomyces* reads only (dashed lines) for three different variables: geographic origin (a), extraction protocol (b) and sample type (c). In this case the two models were trained on the same samples, meaning that the same samples that were excluded from the *Streptomyces* analysis were also removed from the whole-metagenome analysis. Results are similar to those observed in Figure 4 when all the available samples were included for the whole-metagenome analysis, indicating that the change in the model performance was not due to the removal of some samples. This confirms that when the method is applied to *Streptomyces* reads only, good sample geographic origin predictions are achieved while better controlling for batch effects (i.e., lower classification accuracy for the extraction protocol and the sample type).


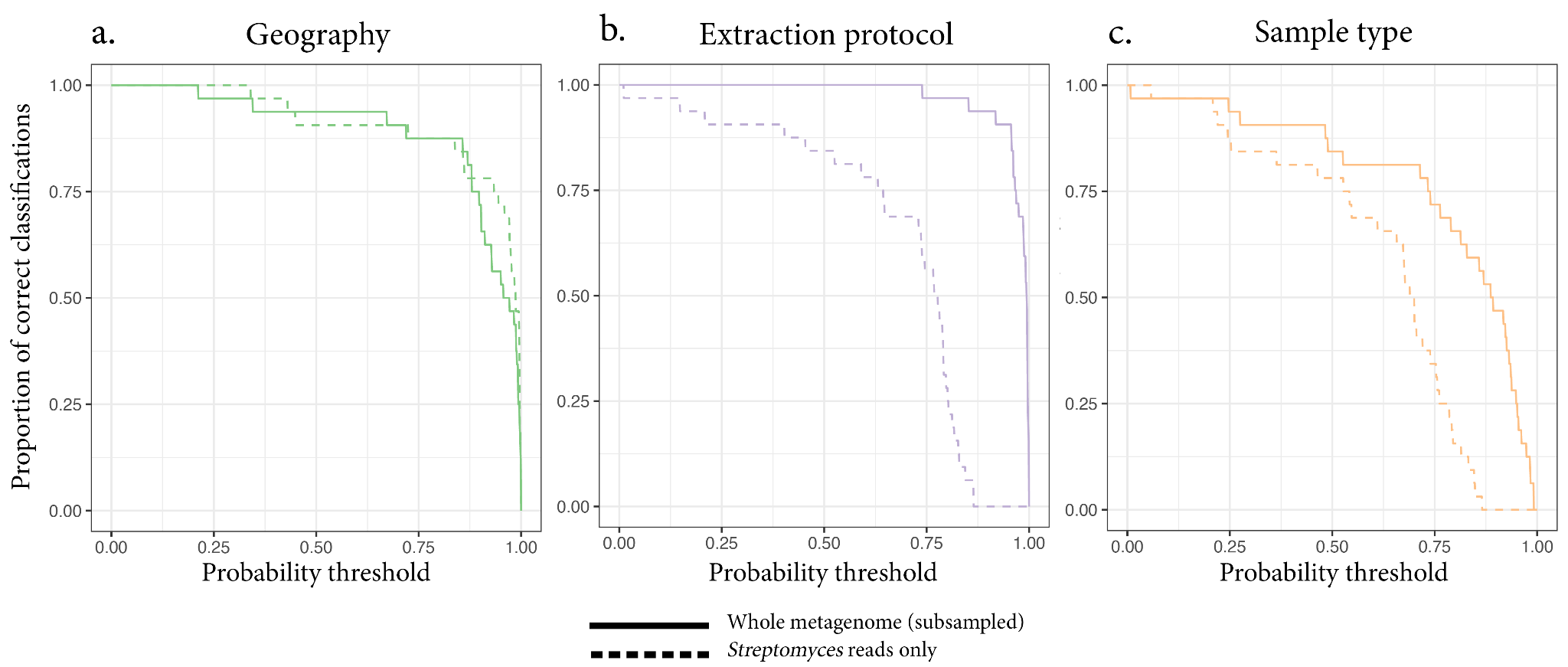


**Supplementary Figure 10. Comparison of the method prediction accuracy when using randomly subsampled whole metagenomes or *Streptomyces* reads only.** To show the validity of *Streptomyces* genus as a biomarker to trace the geographic origin of ancient samples we compared the model performance when using *Streptomyces* reads only (dashed lines) versus the performance when using a random subsample of reads from the whole metagenome (continuous lines). Metagenomes in the Brazil-Polynesia dataset were randomly subsampled to the median number of *Streptomyces* reads (661375). The samples prediction accuracy curves demonstrate that randomly subsampling the whole metagenome, while keeping the same accuracy in predicting the geography variable (a), does not reduce the extraction protocol (b) and sample type (c) biases, here correctly classified with higher probabilities then when using only reads from the *Streptomyces* genus.
