# Supplementary material for: Towards predicting the geographical origin of ancient samples with metagenomic data
Source: Sci Rep. 2024 Sep 18;14:21794. doi: 10.1038/s41598-023-40246-x (PMC11411106; doi:10.1038/s41598-023-40246-x)
Supplement: Supplementary file 6 — Supplementary Information 6. [file 41598_2023_40246_MOESM6_ESM.docx]

List of samples ncbi accessions used for model training and testing:

Denmark-England dataset:

ERR1777519, ERR1777520, ERR1777521, ERR1777522, ERR1777523, ERR1777524, ERR1777525, ERR1777526, ERR1777527, ERR1777528, ERR1777529, ERR1777530, ERR1777531, ERR1777532, ERR1777533, ERR1777534, ERR1777535, ERR1777536, ERR1777537, ERR1777538, ERR1777539, ERR1777540, ERR1777541, ERR1777542, ERR1777543, ERR1777544, ERR1777545, ERR1777546, ERR1777547, ERR1777548, ERR1777549, ERR1777550, ERR1777551, ERR1777552, ERR1777553, ERR1777554, ERR1777555, ERR1777556, ERR1777557, ERR1777558, ERR1777559, ERR1777560, ERR1777561, ERR1777562, ERR1777563, ERR1777564, ERR1777565, ERR1777566, ERR1777567, ERR1777568, ERR1777569, ERR664833,  ERR668419,  ERR668422, ERR966421, ERR966422, ERR966423.

List of samples used for the comparative compositional analysis and SourceTracker2: SRR5581849, SRR5581852, SRR5581857, SRR866660, SRR866662, SRR11562254, SRR11562255, SRR11562279, SRR11562268, SRR11562258, SRR11562259, SRR061192, SRR061320, SRR061365, SRR061562, SRR062083, SRR1761698, SRR1761705, SRR1761710, SRR1761718, SRR1761721, SRR1631060, SRR1631061, SRR1631063, SRR1631064, SRR1633008, SRR1026855, SRR1026854, SRR1026853, SRR1026852, SRR8500449, SRR8500450, SRR8500454, SRR8500455.
