# Supplementary material for: Towards predicting the geographical origin of ancient samples with metagenomic data
Source: Sci Rep. 2024 Sep 18;14:21794. doi: 10.1038/s41598-023-40246-x (PMC11411106; doi:10.1038/s41598-023-40246-x)
Supplement: Supplementary file 9 — Supplementary Information 9. [file 41598_2023_40246_MOESM9_ESM.html]

R\_code\_Bozzi\_et\_al\_2023


# R\_code\_Bozzi\_et\_al\_2023

Load libraries:

```
library(dplyr)
```

```
## Warning: package 'dplyr' was built under R version 4.1.2
```

```
## 
## Attaching package: 'dplyr'
```

```
## The following objects are masked from 'package:stats':
## 
##     filter, lag
```

```
## The following objects are masked from 'package:base':
## 
##     intersect, setdiff, setequal, union
```

```
library(ggplot2)
#options(scipen=999)
```

Read endogenous proportions file:

```
endo <- read.csv(file = "other_plots/endogenous_proportion_datasets.csv", header = T)
```

Plot endogenous proportions:

```
endo %>%
  ggplot(aes(x = factor(sample_id, level = sample_id), y = endo_prop, fill = dataset)) +
  geom_bar(stat = "identity") +
  scale_fill_manual(values=c("#33a02c", "#d95f02","#7570b3", "#1f78b4")) +
  theme_bw() +
  ylab("Endogenous proportion (%)") +
  xlab("Individual sample") +
  theme(axis.text.x = element_text(angle = 45, vjust = 1, hjust=1)) +
  coord_cartesian(ylim = c(0, 100))
```

Read count info file:

```
BP_rcount <- read.csv(file = "other_plots/BP_read_count.csv", header = T)
DE_rcount <- read.csv(file = "other_plots/DE_read_count.csv", header = T)
```

Plot BP read count:

```
BP_rcount %>%
  ggplot(aes(x = sample, y = unmapped_filtered_reads, fill = dataset)) +
  geom_bar(stat = "identity") +
  scale_fill_manual(values=c("#33a02c", "#1f78b4")) +
  theme_bw() +
  ylab("Number of reads") +
  xlab("Individual sample") +
  theme(axis.text.x = element_text(angle = 45, vjust = 1, hjust=1))
```

Plot DE read count:

```
DE_rcount %>%
  ggplot(aes(x = sample, y = unmapped_filtered_reads, fill = dataset)) +
  geom_bar(stat = "identity") +
  scale_fill_manual(values=c("#d95f02", "#7570b3")) +
  theme_bw() +
  ylab("Number of reads") +
  xlab("Individual sample") +
  theme(axis.text.x = element_text(angle = 45, vjust = 1, hjust=1))
```

Plot all the datasets together (log scale):

```
all_rcount <- rbind(BP_rcount, DE_rcount)

all_rcount %>%
  ggplot(aes(x = factor(sample, level = sample), y = unmapped_filtered_reads, fill = dataset)) +
  geom_bar(stat = "identity") +
  scale_y_continuous(trans = "log10") +
  scale_fill_manual(values=c("#33a02c", "#d95f02","#7570b3", "#1f78b4")) +
  theme_bw() +
  ylab("Number of reads") +
  xlab("Individual sample") +
  theme(axis.text.x = element_text(angle = 45, vjust = 1, hjust=1))
```

# Investigating samples aDNA damage patterns (metadamge output)

Read data BP dataset:

```
# brazilians:
brazil_nofilter <- read.csv(file = "100k_damage/brazil_damage_nofilter.csv", header = T)
brazil_nofilter$type <- c("nofilter")
brazil_filtered <- read.csv(file = "100k_damage/brazil_damage_filtered.csv", header = T)
brazil_filtered$type <- c("filtered")
## merge data:
brazil_merged <- rbind(brazil_nofilter, brazil_filtered)

# polynesians:
poly_nofilter <- read.csv(file = "100k_damage/poly_damage_nofilter.csv", header = T)
poly_nofilter$type <- c("nofilter")
poly_filtered <- read.csv(file = "100k_damage/poly_damage_filtered.csv", header = T)
poly_filtered$type <- c("filtered")
## merge data:
poly_merged <- rbind(poly_nofilter, poly_filtered)

# poly boto:
pb_nofilter <- read.csv(file = "100k_damage/poly_boto_damage_nofilter.csv", header = T)
```

```
## Warning in read.table(file = file, header = header, sep = sep, quote =
## quote, : incomplete final line found by readTableHeader on '100k_damage/
## poly_boto_damage_nofilter.csv'
```

```
pb_nofilter$type <- c("nofilter")
pb_filtered <- read.csv(file = "100k_damage/poly_boto_damage_filtered.csv", header = T)
```

```
## Warning in read.table(file = file, header = header, sep = sep, quote =
## quote, : incomplete final line found by readTableHeader on '100k_damage/
## poly_boto_damage_filtered.csv'
```

```
pb_filtered$type <- c("filtered")
## merge data:
pb_merged <- rbind(pb_nofilter, pb_filtered)

#fuegans:
fu_nofilter <- read.csv(file = "100k_damage/fuegans_damage_nofilter.csv", header = T)
```

```
## Warning in read.table(file = file, header = header, sep = sep, quote =
## quote, : incomplete final line found by readTableHeader on '100k_damage/
## fuegans_damage_nofilter.csv'
```

```
fu_nofilter$type <- c("nofilter")
fu_filtered <- read.csv(file = "100k_damage/fuegans_damage_filtered.csv", header = T)
```

```
## Warning in read.table(file = file, header = header, sep = sep, quote =
## quote, : incomplete final line found by readTableHeader on '100k_damage/
## fuegans_damage_filtered.csv'
```

```
fu_filtered$type <- c("filtered")
## merge data:
fu_merged <- rbind(fu_nofilter, fu_filtered)
```

Read data DE dataset:

```
# england:
en_nofilter <- read.csv(file = "100k_damage/england_damage_nofilter.csv", header = T)
en_nofilter$type <- c("nofilter")
en_filtered <- read.csv(file = "100k_damage/england_damage_filtered.csv", header = T)
en_filtered$type <- c("filtered")
## merge data:
en_merged <- rbind(en_nofilter, en_filtered)

# denmark:
dk_nofilter <- read.csv(file = "100k_damage/denmark_damage_nofilter.csv", header = T)
dk_nofilter$type <- c("nofilter")
dk_filtered <- read.csv(file = "100k_damage/denmark_damage_filtered.csv", header = T)
dk_filtered$type <- c("filtered")
## merge data:
dk_merged <- rbind(dk_nofilter, dk_filtered)
```

Read Streptomyces data (BP dataset)

```
strepto_nofilter <- read.csv(file = "10k_damage/BP_Streptomyces_damage_nofilter.csv", header = T)
strepto_nofilter$type <- c("nofilter")
strepto_filtered <- read.csv(file = "10k_damage/BP_Streptomyces_damage_filtered.csv", header = T)
strepto_filtered$type <- c("filtered")
## merge data:
strepto_merged <- rbind(strepto_nofilter, strepto_filtered)
```

Plot Brazilians damage

```
brazil_merged %>%
  ggplot(aes(x = Input, y = C..T.at.5..end, fill = type, ymax = 0.3))+
  geom_bar(stat = "identity", position = position_dodge(width = 0.9)) +
  geom_errorbar(aes(ymin=C..T.95..CI.lower, ymax=C..T.95..CI.upper), position = position_dodge(width = 0.9)) +
  scale_fill_manual(values=c("#009E73", "#E69F00")) +
  #geom_text(aes(label=blasted_reads), y=0.01, position=position_dodge(width=0.9)) +
  xlab("Individual samples") +
  ylab("C to T at 5' end") +
  theme_bw() +
  theme(axis.text.x = element_text(angle = 45, vjust = 1, hjust=1)) + 
  coord_flip()
```

Plot Polynesians damage:

```
poly_merged %>%
  ggplot(aes(x = Input, y = C..T.at.5..end, fill = type, ymax = 0.1))+
  geom_bar(stat = "identity", position = position_dodge(width = 0.9)) +
  geom_errorbar(aes(ymin=C..T.95..CI.lower, ymax=C..T.95..CI.upper), position = position_dodge(width = 0.9)) +
  scale_fill_manual(values=c("#009E73", "#E69F00")) +
  #geom_text(aes(label=blasted_reads), y=0.01, position=position_dodge(width=0.9)) +
  xlab("Individual samples") +
  ylab("C to T at 5' end") +
  theme_bw() +
  theme(axis.text.x = element_text(angle = 45, vjust = 1, hjust=1)) + 
  coord_flip()
```

Plot Polynesan Botocudos damage:

```
pb_merged %>%
  ggplot(aes(x = Input, y = C..T.at.5..end, fill = type, ymax = 0.1))+
  geom_bar(stat = "identity", position = position_dodge(width = 0.9)) +
  geom_errorbar(aes(ymin=C..T.95..CI.lower, ymax=C..T.95..CI.upper), position = position_dodge(width = 0.9)) +
  scale_fill_manual(values=c("#009E73", "#E69F00")) +
  #geom_text(aes(label=blasted_reads), y=0.01, position=position_dodge(width=0.9)) +
  xlab("Individual samples") +
  ylab("C to T at 5' end") +
  theme_bw() +
  theme(axis.text.x = element_text(angle = 45, vjust = 1, hjust=1)) + 
  coord_flip()
```

Plot Fuego-Patagonians damage:

```
fu_merged %>%
  ggplot(aes(x = Input, y = C..T.at.5..end, fill = type, ymax = 0.1))+
  geom_bar(stat = "identity", position = position_dodge(width = 0.9)) +
  geom_errorbar(aes(ymin=C..T.95..CI.lower, ymax=C..T.95..CI.upper), position = position_dodge(width = 0.9)) +
  scale_fill_manual(values=c("#009E73", "#E69F00")) +
  #geom_text(aes(label=blasted_reads), y=0.01, position=position_dodge(width=0.9)) +
  xlab("Individual samples") +
  ylab("C to T at 5' end") +
  theme_bw() +
  theme(axis.text.x = element_text(angle = 45, vjust = 1, hjust=1)) + 
  coord_flip()
```

Plot Denmark damage:

```
dk_merged %>%
  ggplot(aes(x = Input, y = C..T.at.5..end, fill = type, ymax = 0.5))+
  geom_bar(stat = "identity", position = position_dodge(width = 0.9)) +
  geom_errorbar(aes(ymin=C..T.95..CI.lower, ymax=C..T.95..CI.upper), position = position_dodge(width = 0.9)) +
  scale_fill_manual(values=c("#009E73", "#E69F00")) +
  #geom_text(aes(label=blasted_reads), y=0.01, position=position_dodge(width=0.9)) +
  xlab("Individual samples") +
  ylab("C to T at 5' end") +
  theme_bw() +
  theme(axis.text.x = element_text(angle = 45, vjust = 1, hjust=1)) + 
  coord_flip()
```

Plot England damage:

```
en_merged %>%
  ggplot(aes(x = Input, y = C..T.at.5..end, fill = type, ymax = 0.6))+
  geom_bar(stat = "identity", position = position_dodge(width = 0.6)) +
  geom_errorbar(aes(ymin=C..T.95..CI.lower, ymax=C..T.95..CI.upper), position = position_dodge(width = 0.9)) +
  scale_fill_manual(values=c("#009E73", "#E69F00")) +
  #geom_text(aes(label=blasted_reads), y=0.01, position=position_dodge(width=0.9)) +
  xlab("Individual samples") +
  ylab("C to T at 5' end") +
  theme_bw() +
  theme(axis.text.x = element_text(angle = 45, vjust = 1, hjust=1)) + 
  coord_flip()
```

Plot damage for Streptomyces reads (BP dataset - pre-filtering):

```
strepto_nofilter %>%
  ggplot(aes(x = Input, y = C..T.at.5..end)) +
  geom_bar(stat = "identity", fill = "grey70") +
  geom_errorbar(aes(ymin=C..T.95..CI.lower, ymax=C..T.95..CI.upper)) +
  scale_fill_manual(values=c("#009E73", "#E69F00")) +
  xlab("Individual samples") +
  ylab("C to T at 5' end") +
  theme_bw() +
  theme(axis.text.x = element_text(angle = 45, vjust = 1, hjust=1))
```

Plot damage for Streptomyces reads (BP dataset - pre-filtering vs post-filtering):

```
strepto_merged %>%
  ggplot(aes(x = Input, y = C..T.at.5..end, fill = type, ymax = 0.3))+
  geom_bar(stat = "identity", position = position_dodge(width = 0.9)) +
  geom_errorbar(aes(ymin=C..T.95..CI.lower, ymax=C..T.95..CI.upper), position = position_dodge(width = 0.9)) +
  scale_fill_manual(values=c("#009E73", "#E69F00")) +
  #geom_text(aes(label=blasted_reads), y=0.01, position=position_dodge(width=0.9)) +
  xlab("Individual samples") +
  ylab("C to T at 5' end") +
  theme_bw() +
  theme(axis.text.x = element_text(angle = 45, vjust = 1, hjust=1)) + 
  coord_flip()
```

# Compositional analysis

Load dplyr:

```
library(dplyr)
```

Import genome size info file downloaded from ncbi (including only bacteria with complete assemblies)

```
ncbi_gen_size <- read.csv("external_data/NCBI_gen_size_bacteria_complete_assembly.csv", header = TRUE)
```

Taxonomy assignment with taxonomizr: Script to generate the taxonomizr database (otuput: accessionTaxa.sql file of ~70GB): note: to be run only once (uncomment the code to run this chunk)

```
#library(taxonomizr)
#prepareDatabase('accessionTaxa.sql')
```

Add taxID information with taxonomizr:

```
library(taxonomizr)
ncbi_gen_size$taxID <- getId(ncbi_gen_size$Organism.Name,'taxonomizr_data/accessionTaxa.sql')
# add other taxonomical information with taxonomizr:
full_taxonomy <- getTaxonomy(ncbi_gen_size$taxID, 'taxonomizr_data/accessionTaxa.sql')
full_taxonomy <- as.data.frame(full_taxonomy)
ncbi_gen_size <- cbind(ncbi_gen_size, full_taxonomy)
rm(full_taxonomy)
# add the taxID of the species:
taxaNames<-read.names('taxonomizr_data/names.dmp')
```

```
## Warning: taxonomizr is moving from data.table to SQLite databases to improve
## performance. This will require changing nodes and names processing. Please see ?
## read.names.sql or ?taxonomizrSwitch
```

```
ncbi_gen_size$species_taxID <- getId((ncbi_gen_size$species), taxaNames)
```

```
## Warning: taxonomizr is moving from data.table to SQLite databases to improve
## performance. This will require changing nodes and names processing. Please see ?
## getId or ?taxonomizrSwitch
```

```
# compute the species and genus mean genome size:
species_gs <- aggregate(Size.Mb. ~ species_taxID, ncbi_gen_size, mean)
genus_gs <- aggregate(Size.Mb. ~ genus, ncbi_gen_size, mean)
```

### B-P dataset:

Improt taxa table (biom format - output of kraken-biom):

```
BP_taxa_table <- read.csv("compositional_analysis/BP_taxatable.csv", header = TRUE, row.names = 1)
# Merge the two libraries for Bot15 (B3a + B3b):
BP_taxa_table$Bot15 <- BP_taxa_table$B3a+BP_taxa_table$B3b
BP_taxa_table$B3a <- NULL
BP_taxa_table$B3b <- NULL
```

Preprocessing: filter low abundant species and add other taxonomical information with taxonomizr

```
# BP_expand BP_taxa_table with taxonomical info:
BP_exp <- getTaxonomy(rownames(BP_taxa_table), 'taxonomizr_data/accessionTaxa.sql')
BP_exp <- as.data.frame(BP_exp)
BP_exp$taxID <- rownames(BP_taxa_table)
BP_taxa_table$taxID <- rownames(BP_taxa_table)
BP_taxa_table_exp = merge(x=BP_taxa_table, y=BP_exp, by="taxID", all.x=TRUE)
# filter hits with less than 10 reads and human:
BP_filtered_taxa <- BP_taxa_table_exp[1,]
for (row in 2:nrow(BP_taxa_table_exp)) {
  if (max(BP_taxa_table_exp[row,2:42]) > 10 & BP_taxa_table_exp[row,1] != "9606" ){
     BP_filtered_taxa <- rbind(BP_filtered_taxa, BP_taxa_table_exp[row,])
  }
}
rm(BP_exp, BP_taxa_table_exp)
# merge BP_taxa_table with species_gs:
BP_taxa_t <- merge(x= BP_filtered_taxa, y=species_gs, by.x = "taxID", by.y = "species_taxID", all.x = TRUE)
rm(BP_filtered_taxa)
# merge BP_taxa_table with genus_gs:
BP_taxa_t <- merge(x= BP_taxa_t, y=genus_gs, by = "genus", all.x = TRUE)
# coalesce genus and species information:
BP_taxa_t <- BP_taxa_t %>% relocate(genus, .before = species)
BP_taxa_t <- BP_taxa_t %>% mutate(Size.Mb..x = coalesce(Size.Mb..x,Size.Mb..y))
```

Remove taxa without genome size information:

```
library(tidyr)
```

```
## Warning: package 'tidyr' was built under R version 4.1.2
```

```
# remove NA - (or manual search?)
BP_taxa_t <- BP_taxa_t %>% drop_na(Size.Mb..x)
BP_taxa_t <- BP_taxa_t %>% dplyr::rename(genome_size = Size.Mb..x)
BP_taxa_t$Size.Mb..y <- NULL
```

Genome size normalization and rarefaction:

```
library(GUniFrac)
```

```
## Warning: package 'GUniFrac' was built under R version 4.1.2
```

```
BP_t_tab <- BP_taxa_t[,2:45]
row.names(BP_t_tab) <- BP_taxa_t$taxID
BP_t_tab <- as.data.frame(t(BP_t_tab))
# genome size normalization
for (i in 1:ncol(BP_t_tab)){
  BP_t_tab[,i] <- BP_t_tab[,i]/BP_taxa_t$genome_size[i]
}
# round to integer
BP_rounded <- round(BP_t_tab)
# rarefy
BP_raretab <- Rarefy(BP_rounded, depth = min(rowSums(BP_rounded)))$otu.tab.rff
rm(BP_rounded)
```

### D-E dataset:

Import taxa table (biom format - output of kraken-biom):

```
DE_taxa_table <- read.csv("compositional_analysis/DE_taxatable.csv", header = TRUE, row.names = 1)
```

Preprocessing: filter low abundant species and add other taxonomical information with taxonomizr

```
# DE_expand DE_taxa_table with taxonomical info:
DE_exp <- getTaxonomy(rownames(DE_taxa_table), 'taxonomizr_data/accessionTaxa.sql')
DE_exp <- as.data.frame(DE_exp)
DE_exp$taxID <- rownames(DE_taxa_table)
DE_taxa_table$taxID <- rownames(DE_taxa_table)
DE_taxa_table_exp = merge(x=DE_taxa_table, y=DE_exp, by="taxID", all.x=TRUE)
# filter hits with less than 10 reads and human:
DE_filtered_taxa <- DE_taxa_table_exp[1,]
for (row in 2:nrow(DE_taxa_table_exp)) {
  if (max(DE_taxa_table_exp[row,2:52]) > 10 & DE_taxa_table_exp[row,1] != "9606" ){
     DE_filtered_taxa <- rbind(DE_filtered_taxa, DE_taxa_table_exp[row,])
  }
}
rm(DE_exp, DE_taxa_table_exp)
# merge DE_taxa_table with species_gs:
DE_taxa_t <- merge(x= DE_filtered_taxa, y=species_gs, by.x = "taxID", by.y = "species_taxID", all.x = TRUE)
rm(DE_filtered_taxa)
# merge DE_taxa_table with genus_gs:
DE_taxa_t <- merge(x= DE_taxa_t, y=genus_gs, by = "genus", all.x = TRUE)
# coalesce genus and species information:
DE_taxa_t <- DE_taxa_t %>% relocate(genus, .before = species)
DE_taxa_t <- DE_taxa_t %>% mutate(Size.Mb..x = coalesce(Size.Mb..x,Size.Mb..y))
```

Remove taxa without genome size information:

```
library(tidyr)
# remove NA - (or manual search?)
DE_taxa_t <- DE_taxa_t %>% drop_na(Size.Mb..x)
DE_taxa_t <- DE_taxa_t %>% dplyr::rename(genome_size = Size.Mb..x)
DE_taxa_t$Size.Mb..y <- NULL
```

Genome size normalization and rarefaction: (differences in the barplot are due to the randomness of the rarefaction procedure)

```
library(GUniFrac)
DE_t_tab <- DE_taxa_t[,2:52]
row.names(DE_t_tab) <- DE_taxa_t$taxID
DE_t_tab <- as.data.frame(t(DE_t_tab))
# genome size normalization
for (i in 1:ncol(DE_t_tab)){
  DE_t_tab[,i] <- DE_t_tab[,i]/DE_taxa_t$genome_size[i]
}
# round to integer
DE_rounded <- round(DE_t_tab)
# rarefy
DE_raretab <- Rarefy(DE_rounded, depth = min(rowSums(DE_rounded)))$otu.tab.rff
rm(DE_rounded)
```

# Compositional barplots:

Stacked barplot - load libraries:

```
library(dplyr)
library(plyr)
```

```
## Warning: package 'plyr' was built under R version 4.1.2
```

```
## ------------------------------------------------------------------------------
```

```
## You have loaded plyr after dplyr - this is likely to cause problems.
## If you need functions from both plyr and dplyr, please load plyr first, then dplyr:
## library(plyr); library(dplyr)
```

```
## ------------------------------------------------------------------------------
```

```
## 
## Attaching package: 'plyr'
```

```
## The following objects are masked from 'package:dplyr':
## 
##     arrange, count, desc, failwith, id, mutate, rename, summarise,
##     summarize
```

```
library(tidyverse)
```

```
## Warning: package 'tidyverse' was built under R version 4.1.2
```

```
## Registered S3 method overwritten by 'httr':
##   method         from  
##   print.response rmutil
```

```
## ── Attaching packages ─────────────────────────────────────── tidyverse 1.3.2 ──
## ✔ tibble  3.2.1     ✔ stringr 1.4.0
## ✔ readr   2.1.2     ✔ forcats 0.5.1
## ✔ purrr   1.0.1
```

```
## Warning: package 'tibble' was built under R version 4.1.2
```

```
## Warning: package 'readr' was built under R version 4.1.2
```

```
## Warning: package 'purrr' was built under R version 4.1.2
```

```
## ── Conflicts ────────────────────────────────────────── tidyverse_conflicts() ──
## ✖ plyr::arrange()   masks dplyr::arrange()
## ✖ purrr::compact()  masks plyr::compact()
## ✖ plyr::count()     masks dplyr::count()
## ✖ plyr::desc()      masks dplyr::desc()
## ✖ plyr::failwith()  masks dplyr::failwith()
## ✖ dplyr::filter()   masks stats::filter()
## ✖ plyr::id()        masks dplyr::id()
## ✖ dplyr::lag()      masks stats::lag()
## ✖ plyr::mutate()    masks dplyr::mutate()
## ✖ plyr::rename()    masks dplyr::rename()
## ✖ plyr::summarise() masks dplyr::summarise()
## ✖ plyr::summarize() masks dplyr::summarize()
```

```
library(RColorBrewer)
library(ggplot2)
library(reshape2)
```

```
## 
## Attaching package: 'reshape2'
## 
## The following object is masked from 'package:tidyr':
## 
##     smiths
```

Plot stacked barplot at the phylum level:

```
BP_phyla <- as.data.frame(BP_raretab)
BP_phyla <- as.data.frame(t(BP_phyla))
BP_phyla <- scale(BP_phyla, center = FALSE, 
               scale = colSums(BP_phyla))
BP_phyla <- as.data.frame(BP_phyla)
BP_phyla <- cbind(BP_phyla, BP_taxa_t$phylum)
names(BP_phyla)[names(BP_phyla) == "BP_taxa_t$phylum"] <- 'Phyla'
# remove target samples
BP_phyla <- BP_phyla[,5:45]
BP_phyla$Bot15 <- NULL
BP_phyla_agg <- ddply(BP_phyla,.(Phyla),numcolwise(sum))
BP_phyla_long <- melt(BP_phyla_agg, id.vars = "Phyla", variable.name = "Sample")
BP_phyla_long <- BP_phyla_long[order(BP_phyla_long$Phyla),]
# convert Phylum to a character vector from a factor because R
BP_phyla_long$Phyla <- as.character(BP_phyla_long$Phyla)
# group dataframe by Phylum, calculate median rel. abundance
medians <- ddply(BP_phyla_long, ~Phyla, function(x) c(median=median(x$value)))
# find Phyla whose rel. abund. is less than 1%
Other <- medians[medians$median <= 0.001,]$Phyla
# change their name to "Other Phyla"
BP_phyla_long[BP_phyla_long$Phyla %in% Other,]$Phyla <- 'Other Phyla'
BP_phyla_palette <- c("#80b1d3", "#fb8072", "#fdb462", "#bebada", "#b3de69", "#fccde5", "#8dd3c7", "#ffffb3", "#d9d9d9")
ggplot(BP_phyla_long, aes(fill=fct_reorder(Phyla, value, .desc = TRUE), y=value, x=Sample)) + 
  geom_bar(stat="identity") +
  theme_bw() +
  theme(axis.text.x = element_text(angle = 45, hjust=1)) + 
  scale_fill_manual(values = BP_phyla_palette)
```

Plot stacked barplot at the genus level:

```
BP_genus <- as.data.frame(BP_raretab)
BP_genus <- as.data.frame(t(BP_genus))
BP_genus <- scale(BP_genus, center = FALSE, 
               scale = colSums(BP_genus))
BP_genus <- as.data.frame(BP_genus)
BP_genus <- cbind(BP_genus, BP_taxa_t$genus)
names(BP_genus)[names(BP_genus) == "BP_taxa_t$genus"] <- 'group'
BP_genus <- BP_genus[,5:45]
BP_genus$Bot15 <- NULL
BP_genus_agg <- ddply(BP_genus,.(group),numcolwise(sum))
BP_genus_long <- melt(BP_genus_agg, id.vars = "group", variable.name = "Sample")
BP_genus_long <- BP_genus_long[order(BP_genus_long$group),]
# convert Phylum to a character vector from a factor because R
BP_genus_long$group <- as.character(BP_genus_long$group)
# group dataframe by Phylum, calculate median rel. abundance
medians <- ddply(BP_genus_long, ~group, function(x) c(median=median(x$value)))
# find group whose rel. abund. is less than 1%
Other <- medians[medians$median <= 0.01,]$group
# change their name to "Other Prokaryotes"
BP_genus_long[BP_genus_long$group %in% Other,]$group <- 'Other Genera'
# create a palette
BP_genus_palette <- c("#66C2A5", "#9DAE8C", "#F08F6D", "#D49A73", "#BABF77", "#9F9BC9", "#D2A29F", "#C79693", "#C193C6", "#E28BC3", "#AAD852", "#CBD844", "#ECD836", "#FAD53E", "#F1CD64", "#E7C689", "#D7BF9C", "#C5B9A7", "#B3B3B3")
ggplot(BP_genus_long, aes(fill=fct_reorder(group, value, .desc = TRUE), y=value, x=Sample)) + 
  geom_bar(stat="identity") +
  theme_bw() +
  theme(axis.text.x = element_text(angle = 45, hjust=1)) +
  scale_fill_manual(values = BP_genus_palette)
```

Plot stacked barplot at the phylum level:

```
DE_phyla <- as.data.frame(DE_raretab)
DE_phyla <- as.data.frame(t(DE_phyla))
DE_phyla <- scale(DE_phyla, center = FALSE, 
               scale = colSums(DE_phyla))
DE_phyla <- as.data.frame(DE_phyla)
DE_phyla <- cbind(DE_phyla, DE_taxa_t$phylum)
names(DE_phyla)[names(DE_phyla) == "DE_taxa_t$phylum"] <- 'Phyla'
DE_phyla_agg <- ddply(DE_phyla,.(Phyla),numcolwise(sum))
DE_phyla_long <- melt(DE_phyla_agg, id.vars = "Phyla", variable.name = "Sample")
DE_phyla_long <- DE_phyla_long[order(DE_phyla_long$Phyla),]
# convert Phylum to a character vector from a factor because R
DE_phyla_long$Phyla <- as.character(DE_phyla_long$Phyla)
# group dataframe by Phylum, calculate median rel. abundance
medians <- ddply(DE_phyla_long, ~Phyla, function(x) c(median=median(x$value)))
# find Phyla whose rel. abund. is less than 1%
Other <- medians[medians$median <= 0.001,]$Phyla
# change their name to "Other Phyla"
DE_phyla_long[DE_phyla_long$Phyla %in% Other,]$Phyla <- 'Other Phyla'
DE_phyla_palette <- c("#80b1d3", "#fb8072", "#fdb462", "#bebada", "#fccde5","#b3de69",  "#8dd3c7", "#ccebc5", "#b15928", "#ffed6f", "#6a3d9a", "#CBD844", "#d9d9d9", "white")
ggplot(DE_phyla_long, aes(fill=fct_reorder(Phyla, value, .desc = TRUE), y=value, x=Sample)) + 
  geom_bar(stat="identity") +
  theme_bw() +
  theme(axis.text.x = element_text(angle = 45, hjust=1)) + 
  scale_fill_manual(values = DE_phyla_palette)
```

Plot stacked barplot at the genus level:

```
DE_genus <- as.data.frame(DE_raretab)
DE_genus <- as.data.frame(t(DE_genus))
DE_genus <- scale(DE_genus, center = FALSE, 
               scale = colSums(DE_genus))
DE_genus <- as.data.frame(DE_genus)
DE_genus <- cbind(DE_genus, DE_taxa_t$genus)
names(DE_genus)[names(DE_genus) == "DE_taxa_t$genus"] <- 'group'
DE_genus_agg <- ddply(DE_genus,.(group),numcolwise(sum))
DE_genus_long <- melt(DE_genus_agg, id.vars = "group", variable.name = "Sample")
DE_genus_long <- DE_genus_long[order(DE_genus_long$group),]
# convert Phylum to a character vector from a factor because R
DE_genus_long$group <- as.character(DE_genus_long$group)
# group dataframe by Phylum, calculate median rel. abundance
medians <- ddply(DE_genus_long, ~group, function(x) c(median=median(x$value)))
# find group whose rel. abund. is less than 1%
Other <- medians[medians$median <= 0.01,]$group
# change their name to "Other Prokaryotes"
DE_genus_long[DE_genus_long$group %in% Other,]$group <- 'Other Genera'
# create a palette
DE_genus_palette <- c("#66C2A5", "#9DAE8C", "#C193C6", "#E28BC3","#F08F6D", "#BABF77", "#D49A73", "#C79693", "white")
ggplot(DE_genus_long, aes(fill=fct_reorder(group, value, .desc = TRUE), y=value, x=Sample)) + 
  geom_bar(stat="identity") +
  theme_bw() +
  theme(axis.text.x = element_text(angle = 45, hjust=1)) +
  scale_fill_manual(values = DE_genus_palette)
```

Get Streptomyces median abundance:

```
BP_strep <- BP_genus_long[BP_genus_long$group == 'Streptomyces',] 
DE_strep <- DE_genus_long[DE_genus_long$group == 'Streptomyces',]
strep <- rbind(BP_strep, DE_strep)
median(strep$value)
```

```
## [1] 0.05745968
```

# Dimensionality reduction

Import the table with all the dataset of interest

```
PCA_taxa_table <- read.csv("compositional_analysis/PCA_taxa_table.csv", header = T, row.names = 1)
```

Preprocessing: filter low abundant species and add other taxonomical information with taxonomizr

```
# PCA_expand PCA_taxa_table with taxonomical info:
PCA_exp <- getTaxonomy(rownames(PCA_taxa_table), 'taxonomizr_data/accessionTaxa.sql')
PCA_exp <- as.data.frame(PCA_exp)
PCA_exp$taxID <- rownames(PCA_taxa_table)
PCA_taxa_table$taxID <- rownames(PCA_taxa_table)
PCA_taxa_table_exp = merge(x=PCA_taxa_table, y=PCA_exp, by="taxID", all.x=TRUE)
# filter hits with less than 10 reads and human:
PCA_filtered_taxa <- PCA_taxa_table_exp[1,]
for (row in 2:nrow(PCA_taxa_table_exp)) {
  if (max(PCA_taxa_table_exp[row,2:72]) > 10 & PCA_taxa_table_exp[row,1] != "9606" ){
     PCA_filtered_taxa <- rbind(PCA_filtered_taxa, PCA_taxa_table_exp[row,])
  }
}
rm(PCA_exp, PCA_taxa_table_exp)
# merge PCA_taxa_table with species_gs:
PCA_taxa_t <- merge(x= PCA_filtered_taxa, y=species_gs, by.x = "taxID", by.y = "species_taxID", all.x = TRUE)
rm(PCA_filtered_taxa)
# merge PCA_taxa_table with genus_gs:
PCA_taxa_t <- merge(x= PCA_taxa_t, y=genus_gs, by = "genus", all.x = TRUE)
# coalesce genus and species information:
PCA_taxa_t <- PCA_taxa_t %>% relocate(genus, .before = species)
PCA_taxa_t <- PCA_taxa_t %>% mutate(Size.Mb..x = coalesce(Size.Mb..x,Size.Mb..y))
```

Remove taxa without genome size information:

```
library(tidyr)
# remove NA - (or manual search?)
PCA_taxa_t <- PCA_taxa_t %>% drop_na(Size.Mb..x)
PCA_taxa_t <- PCA_taxa_t %>% dplyr::rename(genome_size = Size.Mb..x)
PCA_taxa_t$Size.Mb..y <- NULL
```

Genome size normalization and rarefaction: (differencies in the barplot are due to the randomness of the rarefaction procedure)

```
library(GUniFrac)
PCA_t_tab <- PCA_taxa_t[,2:72]
row.names(PCA_t_tab) <- PCA_taxa_t$taxID
PCA_t_tab <- as.data.frame(t(PCA_t_tab))
# genome size normalization
for (i in 1:ncol(PCA_t_tab)){
  PCA_t_tab[,i] <- PCA_t_tab[,i]/PCA_taxa_t$genome_size[i]
}
# round to integer
PCA_rounded <- round(PCA_t_tab)
# rarefy
PCA_raretab <- Rarefy(PCA_rounded, depth = min(rowSums(PCA_rounded)))$otu.tab.rff
rm(PCA_rounded)
```

Plot the MDS

```
library(vegan)
```

```
## Warning: package 'vegan' was built under R version 4.1.2
```

```
## Loading required package: permute
```

```
## Warning: package 'permute' was built under R version 4.1.2
```

```
## Loading required package: lattice
```

```
## This is vegan 2.6-2
```

```
library(ggfortify)
```

```
## Warning: package 'ggfortify' was built under R version 4.1.2
```

```
dist_tab <- vegdist(PCA_raretab, method = "jaccard")
#raretab <- as.data.frame(t(raretab))
pca <- prcomp(dist_tab)
col_vec <- c("#1f78b4", "#1f78b4",  "#1f78b4",  "#1f78b4",  "#1f78b4",  "#ffd92f",  "#ffd92f",  "#ffd92f",  "#ffd92f",  "#ffd92f",  "#cab2d6",  "#cab2d6",  "#cab2d6",  "#cab2d6",  "#cab2d6",  "#cab2d6",  "#cab2d6",  "#cab2d6",  "#cab2d6",  "#cab2d6",  "#cab2d6",  "#cab2d6",  "#cab2d6",  "#cab2d6",  "#cab2d6",  "#cab2d6",  "#1f78b4",  "#cab2d6",  "#cab2d6",  "#1f78b4",  "#cab2d6",  "#cab2d6",  "#cab2d6",  "#cab2d6",  "#e31a1c",  "#e31a1c",  "#e31a1c",  "#e31a1c",  "#e31a1c",  "#e31a1c",  "#e31a1c",  "#e31a1c",  "#e31a1c",  "#e31a1c",  "#e31a1c",  "#cab2d6",  "#cab2d6",  "#cab2d6",  "#cab2d6",  "#1f78b4",  "#1f78b4",  "#1f78b4",  "#1f78b4",  "#1f78b4",  "#1f78b4",  "#1f78b4",  "#1f78b4",  "#1f78b4",  "#1f78b4",  "#1f78b4",  "#33a02c",  "#33a02c",  "#33a02c",  "#33a02c",  "#33a02c",  "#b15928",  "#b15928",  "#b15928",  "#b15928",  "#b15928",  "#b15928")

autoplot(pca, x = 1, y = 2, pch = 16, size = 6, alpha =0.8, label = F, label.size = 3, label.colour = NULL, label.hjust=0.0001, label.vjust=-0.5,
    col = col_vec) +
    theme_bw()
```

# SourceTracker2

B-P dataset:

```
library(ggplot2)
library(reshape2)
# import SourceTracker2 output for plotting
BP_st <- read.csv("sourcetracker2/BP_mixing_proportions.csv", row.names = 1)

BP_st["sample_name"] <- rownames(BP_st)
BP_st.molten <- melt(BP_st, id.vars="sample_name")

ggplot(data=BP_st.molten, aes(x=sample_name, y=value, fill=variable)) +
  geom_bar(stat="identity") + 
  scale_fill_manual(values=c("#1f78b4", "#b2df8a", "#33a02c", "#fb9a99", "#e31a1c", "#fdbf6f")) +
  theme_bw() +
  theme(axis.text.x = element_text(angle = 45, hjust=1))
```

D-E dataset (all samples - including low-depth ones):

```
# import SourceTracker2 output for plotting
DE_st <- read.csv("sourcetracker2/DE_mixing_proportions.csv", row.names = 1)

DE_st["sample_name"] <- rownames(DE_st)
DE_st.molten <- melt(DE_st, id.vars="sample_name")

ggplot(data=DE_st.molten, aes(x=sample_name, y=value, fill=variable)) +
  geom_bar(stat="identity") + 
  scale_fill_manual(values=c("#1f78b4", "#b2df8a", "#33a02c", "#fb9a99", "#e31a1c", "#fdbf6f")) +
  theme_bw() +
  theme(axis.text.x = element_text(angle = 45, hjust=1))
```

# k-mers-based similarities

Read BP data into R:

```
BP_mash <- read.csv("kmers_similarity_matrices/BP_similarity_matrix.csv")
# Add row names
rownames(BP_mash) <- names(BP_mash)
# Transform to matrix object for plotting
BP_mash_mat <- as.matrix(BP_mash)
# Keep only reference samples
BP_mash_mat <- BP_mash_mat[6:43, 6:43]
```

Read DE data into R: (only samples with >1 million reads)

```
# import data
DE_mash <- read.csv("kmers_similarity_matrices/DE_similarity_matrix.csv")
# Add row names
rownames(DE_mash) <- names(DE_mash)
# Transform to matrix object for plotting
DE_mash_mat <- as.matrix(DE_mash)
```

Read data into R: (all the samples)

```
# import data
DE_all_mash <- read.csv("kmers_similarity_matrices/DE_all_similarity_matrix.csv") 
# Add row names
rownames(DE_all_mash) <- names(DE_all_mash)
# Transform to matrix object for plotting
DE_all_mash_mat <- as.matrix(DE_all_mash)
```

BP dataset MDS

```
library(ggfortify)
BP_pca_mat <- prcomp(BP_mash_mat, scale. = TRUE)
```

Plot: (color according to geography) green = Brazil blue = Polynesia (RapaNui)

```
BP_col_vec <- c("#33a02c", "#33a02c", "#33a02c", "#33a02c", "#33a02c", "#33a02c", "#33a02c", "#33a02c", "#33a02c", "#33a02c", "#33a02c", "#33a02c", "#33a02c", "#33a02c", "#33a02c", "#33a02c", "#33a02c", "#33a02c", "#33a02c", "#33a02c", "#33a02c", "#33a02c", "#33a02c", "#1f78b4", "#1f78b4", "#1f78b4", "#1f78b4", "#1f78b4", "#1f78b4", "#1f78b4", "#1f78b4", "#1f78b4", "#1f78b4", "#1f78b4", "#1f78b4", "#1f78b4", "#1f78b4", "#1f78b4")

autoplot(BP_pca_mat, x = 1, y = 2, pch = 16, label = F, size = 4, label.size = 3, alpha = 0.7, label.colour = NULL, label.hjust=0.0001, label.vjust=-0.5,
          colour = BP_col_vec) +
     theme_bw()
```

```
autoplot(BP_pca_mat, x = 1, y = 3, pch = 16, label = F, size = 4, label.size = 3, alpha = 0.7, label.colour = NULL, label.hjust=0.0001, label.vjust=-0.5,
          colour = BP_col_vec) +
     theme_bw()
```

Plot: (shape according to extraction protocol) triangle = Allentoft square = Damgaard

```
BP_shape_vec_ex <- c(17, 17, 17, 17, 17, 17, 17, 17, 17, 17, 17, 17, 17, 17, 17, 17, 17, 17, 17, 17, 17, 17, 17, 17, 17, 17, 17, 15, 15, 15, 15, 15, 15, 15, 15, 15, 15, 15)

autoplot(BP_pca_mat, x = 1, y = 2, pch = 16, label = F, size = 4, label.size = 3, alpha = 0.7, label.colour = NULL, label.hjust=0.0001, label.vjust=-0.5, colour = BP_col_vec, shape = BP_shape_vec_ex) +
     theme_bw()
```

```
autoplot(BP_pca_mat, x = 1, y = 3, pch = 16, label = F, size = 4, label.size = 3, alpha = 0.7, label.colour = NULL, label.hjust=0.0001, label.vjust=-0.5, colour = BP_col_vec, shape = BP_shape_vec_ex) +
     theme_bw()
```

Plot: (shape according to sample type) diamond = Bone circle = Tooth

```
BP_shape_vec_sm <- c(19, 19, 19, 18, 19, 19, 19, 19, 19, 19, 19, 19, 19, 19, 19, 19, 19, 19, 19, 19, 19, 19, 19, 19, 19, 19, 19, 18, 18, 18, 18, 18, 18, 18, 18, 18, 18, 18)

autoplot(BP_pca_mat, x = 1, y = 2, pch = 16, label = F, size = 4, label.size = 3, alpha = 0.7, label.colour = NULL, label.hjust=0.0001, label.vjust=-0.5, colour = BP_col_vec, shape = BP_shape_vec_sm) +
     theme_bw()
```

```
autoplot(BP_pca_mat, x = 1, y = 3, pch = 16, label = F, size = 4, label.size = 3, alpha = 0.7, label.colour = NULL, label.hjust=0.0001, label.vjust=-0.5, colour = BP_col_vec, shape = BP_shape_vec_sm) +
     theme_bw()
```

Plot dimensions contribution:

```
library(factoextra)
```

```
## Welcome! Want to learn more? See two factoextra-related books at https://goo.gl/ve3WBa
```

```
fviz_eig(BP_pca_mat, ncp = 43)
```

```
summary(BP_pca_mat)
```

```
## Importance of components:
##                           PC1     PC2    PC3     PC4     PC5     PC6    PC7
## Standard deviation     2.4392 1.58678 1.3976 1.25298 1.20890 1.14913 1.1164
## Proportion of Variance 0.1566 0.06626 0.0514 0.04131 0.03846 0.03475 0.0328
## Cumulative Proportion  0.1566 0.22283 0.2742 0.31554 0.35400 0.38875 0.4215
##                            PC8     PC9    PC10    PC11    PC12    PC13   PC14
## Standard deviation     1.05743 1.04105 1.03186 1.02475 1.01553 1.01116 1.0015
## Proportion of Variance 0.02943 0.02852 0.02802 0.02763 0.02714 0.02691 0.0264
## Cumulative Proportion  0.45098 0.47950 0.50752 0.53515 0.56229 0.58920 0.6156
##                           PC15    PC16    PC17    PC18    PC19    PC20    PC21
## Standard deviation     0.98808 0.98608 0.97680 0.97218 0.96408 0.95519 0.93126
## Proportion of Variance 0.02569 0.02559 0.02511 0.02487 0.02446 0.02401 0.02282
## Cumulative Proportion  0.64128 0.66687 0.69198 0.71685 0.74131 0.76532 0.78814
##                           PC22    PC23    PC24    PC25    PC26    PC27    PC28
## Standard deviation     0.91236 0.90517 0.88985 0.87492 0.86069 0.83562 0.78964
## Proportion of Variance 0.02191 0.02156 0.02084 0.02014 0.01949 0.01838 0.01641
## Cumulative Proportion  0.81005 0.83161 0.85245 0.87259 0.89209 0.91046 0.92687
##                           PC29    PC30   PC31    PC32    PC33    PC34   PC35
## Standard deviation     0.77549 0.75675 0.6920 0.65413 0.46258 0.40579 0.3594
## Proportion of Variance 0.01583 0.01507 0.0126 0.01126 0.00563 0.00433 0.0034
## Cumulative Proportion  0.94270 0.95777 0.9704 0.98163 0.98726 0.99159 0.9950
##                           PC36    PC37      PC38
## Standard deviation     0.33127 0.28384 9.503e-17
## Proportion of Variance 0.00289 0.00212 0.000e+00
## Cumulative Proportion  0.99788 1.00000 1.000e+00
```

DE dataset Plot MDS (color according to geography) - only high-depth samples orange = Denmark purple = England

```
library(ggfortify)
DE_pca_mat <- prcomp(DE_mash_mat, scale. = TRUE)
DE_col_vec <- c("#d95f02", "#d95f02", "#d95f02", "#d95f02", "#d95f02", "#d95f02", "#d95f02", "#d95f02", "#d95f02", "#d95f02", "#d95f02", "#d95f02", "#d95f02", "#d95f02", "#d95f02", "#d95f02", "#d95f02", "#d95f02", "#d95f02", "#d95f02", "#d95f02", "#d95f02", "#d95f02", "#d95f02", "#7570b3", "#7570b3", "#7570b3", "#7570b3", "#7570b3", "#7570b3", "#7570b3", "#7570b3", "#7570b3", "#7570b3", "#7570b3")

autoplot(DE_pca_mat, x = 1, y = 2, pch = 16, label.size = 2, size = 4, alpha = 0.7, label = F, label.colour = NULL, label.hjust=0.0001, label.vjust=-0.5,
         colour = DE_col_vec) +
  theme_bw()
```

```
autoplot(DE_pca_mat, x = 2, y = 3, pch = 16, label.size = 2, size = 4, alpha = 0.7, label = F, label.colour = NULL, label.hjust=0.0001, label.vjust=-0.5,
         colour = DE_col_vec) +
  theme_bw()
```

Plot PCs contribution:

```
library(factoextra)
fviz_eig(DE_pca_mat, ncp = 43)
```

```
summary(DE_pca_mat)
```

```
## Importance of components:
##                           PC1     PC2     PC3     PC4     PC5     PC6     PC7
## Standard deviation     1.8835 1.44856 1.28775 1.19886 1.15445 1.11914 1.08359
## Proportion of Variance 0.1014 0.05995 0.04738 0.04106 0.03808 0.03579 0.03355
## Cumulative Proportion  0.1014 0.16131 0.20869 0.24975 0.28783 0.32362 0.35717
##                            PC8     PC9    PC10   PC11    PC12    PC13    PC14
## Standard deviation     1.06521 1.05315 1.03298 1.0281 1.02386 1.01334 1.01047
## Proportion of Variance 0.03242 0.03169 0.03049 0.0302 0.02995 0.02934 0.02917
## Cumulative Proportion  0.38959 0.42127 0.45176 0.4820 0.51191 0.54125 0.57043
##                           PC15    PC16    PC17    PC18   PC19    PC20    PC21
## Standard deviation     1.00451 0.99107 0.98750 0.98408 0.9721 0.96697 0.96136
## Proportion of Variance 0.02883 0.02806 0.02786 0.02767 0.0270 0.02672 0.02641
## Cumulative Proportion  0.59926 0.62732 0.65518 0.68285 0.7098 0.73656 0.76297
##                           PC22    PC23    PC24    PC25    PC26   PC27    PC28
## Standard deviation     0.94432 0.91889 0.91586 0.91013 0.89013 0.8815 0.86532
## Proportion of Variance 0.02548 0.02412 0.02397 0.02367 0.02264 0.0222 0.02139
## Cumulative Proportion  0.78845 0.81257 0.83654 0.86021 0.88284 0.9050 0.92644
##                           PC29   PC30    PC31   PC32    PC33    PC34      PC35
## Standard deviation     0.85364 0.8262 0.77035 0.6772 0.30656 0.13159 8.054e-17
## Proportion of Variance 0.02082 0.0195 0.01696 0.0131 0.00269 0.00049 0.000e+00
## Cumulative Proportion  0.94726 0.9668 0.98372 0.9968 0.99951 1.00000 1.000e+00
```

DE dataset Plot MDS (color according to geography) - also low-depth samples orange = Denmark purple = England

```
library(ggfortify)
DE_all_pca_mat <- prcomp(DE_all_mash_mat, scale. = TRUE)
DE_all_col_vec <- c("#d95f02", "#d95f02", "#d95f02", "#d95f02", "#d95f02", "#d95f02", "#d95f02", "#d95f02", "#d95f02", "#d95f02", "#d95f02", "#d95f02", "#d95f02", "#d95f02", "#d95f02", "#d95f02", "#d95f02", "#d95f02", "#d95f02", "#d95f02", "#d95f02", "#d95f02", "#d95f02", "#d95f02", "#d95f02", "#d95f02", "#d95f02", "#d95f02", "#d95f02", "#7570b3", "#7570b3", "#7570b3", "#7570b3", "#7570b3", "#7570b3", "#7570b3", "#7570b3", "#7570b3", "#7570b3", "#7570b3", "#7570b3", "#7570b3", "#7570b3", "#7570b3", "#7570b3", "#7570b3", "#7570b3", "#7570b3", "#7570b3", "#7570b3", "#7570b3")

autoplot(DE_all_pca_mat, x = 1, y = 3, pch = 16, label.size = 2, size = 6, alpha = 0.7, label = FALSE, label.colour = NULL, label.hjust=0.0001, label.vjust=-0.5,
        colour = DE_all_col_vec) +
        theme_bw()
```

# Model training and accuracy evalutation B-P dataset:

Select type of regularization (0 = ridge, 1 = lasso, 0<a<1 = elastic net):

```
library(glmnet)
```

```
## Warning: package 'glmnet' was built under R version 4.1.2
```

```
## Loading required package: Matrix
```

```
## Warning: package 'Matrix' was built under R version 4.1.2
```

```
## 
## Attaching package: 'Matrix'
```

```
## The following objects are masked from 'package:tidyr':
## 
##     expand, pack, unpack
```

```
## Loaded glmnet 4.1-4
```

```
a = 1
```

Get training dataset:

```
# get the PCs dataframe
BP_train <- as.data.frame(BP_pca_mat$x)
# remove the last PC which explain 0% of the variance
BP_train <- BP_train[,1:37]
```

Geography variable:

```
groups<- as.factor(c("Brazil", "Brazil", "Brazil", "Brazil", "Brazil", "Brazil", "Brazil", "Brazil", "Brazil", "Brazil", "Brazil", "Brazil", "Brazil", "Brazil", "Brazil", "Brazil", "Brazil", "Brazil", "Brazil", "Brazil", "Brazil", "Brazil", "Brazil", "RapaNui", "RapaNui", "RapaNui", "RapaNui", "RapaNui", "RapaNui", "RapaNui", "RapaNui", "RapaNui", "RapaNui", "RapaNui", "RapaNui", "RapaNui", "RapaNui", "RapaNui"))

n <- nrow(BP_train)
# placeholder for storing the i-th prediction
preds <- rep(NA, n)
for(i in 1:n) {
    dataf.BP_train <- BP_train[-i, ]
    dataf.test <- BP_train[i, ]
    groups_tmp <- groups[-i]
    set.seed(123) 
    cv.lasso <- cv.glmnet(as.matrix(dataf.BP_train), groups_tmp, alpha = a, family = "binomial")
    glmnet.fit <- glmnet(as.matrix(dataf.BP_train), groups_tmp, alpha = a, family = "binomial", lambda = cv.lasso$lambda.min)
    preds[i] <- predict(glmnet.fit, as.matrix(dataf.test), type="response")
}

# define the number of elements in class 1
threshold <- seq(0, 1, length.out = 1000)
accuracy <- c()
for (t in threshold){
  tot <- 0
  for (i in 1:(length(preds))){
    if ((groups[i] == "Brazil" && preds[i] < (1 - t)) || (groups[i] == "RapaNui" && preds[i] > (t))){
      tot <- tot + 1
    }
  }
  prop <- tot/length(preds)
  accuracy <- c(accuracy, prop)
}  
BP_geo <- accuracy
```

Extraction protocol variable:

```
groups<- as.factor(c("Allentoft", "Allentoft", "Allentoft", "Allentoft", "Allentoft", "Allentoft", "Allentoft", "Allentoft", "Allentoft", "Allentoft", "Allentoft", "Allentoft", "Allentoft", "Allentoft", "Allentoft", "Allentoft", "Allentoft", "Allentoft", "Allentoft", "Allentoft", "Allentoft", "Allentoft", "Allentoft", "Allentoft", "Allentoft", "Allentoft", "Allentoft", "Damgaard", "Damgaard", "Damgaard", "Damgaard", "Damgaard", "Damgaard", "Damgaard", "Damgaard", "Damgaard", "Damgaard", "Damgaard"))

n <- nrow(BP_train)
# placeholder for storing the i-th prediction
preds <- rep(NA, n)
for(i in 1:n) {
    dataf.BP_train <- BP_train[-i, ]
    dataf.test <- BP_train[i, ]
    groups_tmp <- groups[-i]
    set.seed(123) 
    cv.lasso <- cv.glmnet(as.matrix(dataf.BP_train), groups_tmp, alpha = a, family = "binomial")
    glmnet.fit <- glmnet(as.matrix(dataf.BP_train), groups_tmp, alpha = a, family = "binomial", lambda = cv.lasso$lambda.min)
    preds[i] <- predict(glmnet.fit, as.matrix(dataf.test), type="response")
}
```

```
## Warning in lognet(xd, is.sparse, ix, jx, y, weights, offset, alpha, nobs, : one
## multinomial or binomial class has fewer than 8 observations; dangerous ground

## Warning in lognet(xd, is.sparse, ix, jx, y, weights, offset, alpha, nobs, : one
## multinomial or binomial class has fewer than 8 observations; dangerous ground

## Warning in lognet(xd, is.sparse, ix, jx, y, weights, offset, alpha, nobs, : one
## multinomial or binomial class has fewer than 8 observations; dangerous ground

## Warning in lognet(xd, is.sparse, ix, jx, y, weights, offset, alpha, nobs, : one
## multinomial or binomial class has fewer than 8 observations; dangerous ground

## Warning in lognet(xd, is.sparse, ix, jx, y, weights, offset, alpha, nobs, : one
## multinomial or binomial class has fewer than 8 observations; dangerous ground

## Warning in lognet(xd, is.sparse, ix, jx, y, weights, offset, alpha, nobs, : one
## multinomial or binomial class has fewer than 8 observations; dangerous ground

## Warning in lognet(xd, is.sparse, ix, jx, y, weights, offset, alpha, nobs, : one
## multinomial or binomial class has fewer than 8 observations; dangerous ground

## Warning in lognet(xd, is.sparse, ix, jx, y, weights, offset, alpha, nobs, : one
## multinomial or binomial class has fewer than 8 observations; dangerous ground

## Warning in lognet(xd, is.sparse, ix, jx, y, weights, offset, alpha, nobs, : one
## multinomial or binomial class has fewer than 8 observations; dangerous ground

## Warning in lognet(xd, is.sparse, ix, jx, y, weights, offset, alpha, nobs, : one
## multinomial or binomial class has fewer than 8 observations; dangerous ground

## Warning in lognet(xd, is.sparse, ix, jx, y, weights, offset, alpha, nobs, : one
## multinomial or binomial class has fewer than 8 observations; dangerous ground
```

```
# define the number of elements in class 1
threshold <- seq(0, 1, length.out = 1000)
accuracy <- c()
for (t in threshold){
  tot <- 0
  for (i in 1:(length(preds))){
    if ((groups[i] == "Allentoft" && preds[i] < (1 - t)) || (groups[i] == "Damgaard" && preds[i] > (t))){
      tot <- tot + 1
    }
  }
  prop <- tot/length(preds)
  accuracy <- c(accuracy, prop)
}  
BP_extraction_protocol <- accuracy
```

Sample type variable:

```
groups<- as.factor(c("Tooth", "Tooth", "Tooth", "Bone", "Tooth", "Tooth", "Tooth", "Tooth", "Tooth", "Tooth", "Tooth", "Tooth", "Tooth", "Tooth", "Tooth", "Tooth", "Tooth", "Tooth", "Tooth", "Tooth", "Tooth", "Tooth", "Tooth", "Tooth", "Tooth", "Tooth", "Tooth", "Bone", "Bone", "Bone", "Bone", "Bone", "Bone", "Bone", "Bone", "Bone", "Bone", "Bone"))

n <- nrow(BP_train)
# placeholder for storing the i-th prediction
preds <- rep(NA, n)
for(i in 1:n) {
    dataf.BP_train <- BP_train[-i, ]
    dataf.test <- BP_train[i, ]
    groups_tmp <- groups[-i]
    set.seed(123) 
    cv.lasso <- cv.glmnet(as.matrix(dataf.BP_train), groups_tmp, alpha = a, family = "binomial")
    glmnet.fit <- glmnet(as.matrix(dataf.BP_train), groups_tmp, alpha = a, family = "binomial", lambda = cv.lasso$lambda.min)
    preds[i] <- predict(glmnet.fit, as.matrix(dataf.test), type="response")
}

# define the number of elements in class 1
threshold <- seq(0, 1, length.out = 1000)
accuracy <- c()
for (t in threshold){
  tot <- 0
  for (i in 1:(length(preds))){
    if ((groups[i] == "Bone" && preds[i] < (1 - t)) || (groups[i] == "Tooth" && preds[i] > (t))){
      tot <- tot + 1
    }
  }
  prop <- tot/length(preds)
  accuracy <- c(accuracy, prop)
}  
BP_sample_type <- accuracy
```

Negative control (mock variable):

```
groups<- as.factor(c("group1", "group2", "group1", "group2", "group1", "group2", "group1", "group2", "group1", "group2", "group1", "group2", "group1", "group2", "group1", "group2", "group1", "group2", "group1", "group2", "group1", "group2", "group1", "group2", "group1", "group2", "group1", "group2", "group1", "group2", "group1", "group2", "group1", "group2", "group1", "group2", "group1", "group2"))

n <- nrow(BP_train)
# placeholder for storing the i-th prediction
preds <- rep(NA, n)
for(i in 1:n) {
    dataf.BP_train <- BP_train[-i, ]
    dataf.test <- BP_train[i, ]
    groups_tmp <- groups[-i]
    set.seed(123) 
    cv.lasso <- cv.glmnet(as.matrix(dataf.BP_train), groups_tmp, alpha = a, family = "binomial")
    glmnet.fit <- glmnet(as.matrix(dataf.BP_train), groups_tmp, alpha = a, family = "binomial", lambda = cv.lasso$lambda.min)
    preds[i] <- predict(glmnet.fit, as.matrix(dataf.test), type="response")
}

# define the number of elements in class 1
threshold <- seq(0, 1, length.out = 1000)
accuracy <- c()
for (t in threshold){
  tot <- 0
  for (i in 1:(length(preds))){
    if ((groups[i] == "group1" && preds[i] < (1 - t)) || (groups[i] == "group2" && preds[i] > (t))){
      tot <- tot + 1
    }
  }
  prop <- tot/length(preds)
  accuracy <- c(accuracy, prop)
}  
BP_neg_ctrl <- accuracy
```

Plot accuracy curve for the B-P dataset (three variables):

```
ggplot() + 
  geom_line(aes(threshold, BP_geo), color = "#7fc97f") +
  geom_line(aes(threshold, BP_extraction_protocol), color = "#beaed4") +
  geom_line(aes(threshold, BP_sample_type), color = "#fdc086") +
  theme_bw() +
  ylim(0, 1)
```

Plot accuracy curve for the B-P dataset (three variables and random romk variable):

```
ggplot() + 
  geom_line(aes(threshold, BP_geo), color = "#7fc97f") +
  geom_line(aes(threshold, BP_extraction_protocol), color = "#beaed4") +
  geom_line(aes(threshold, BP_sample_type), color = "#fdc086") +
  geom_line(aes(threshold, BP_neg_ctrl), color = "#fb9a99") +
  theme_bw() +
  ylim(0, 1)
```

## Model training and accuracy evalutation D-E dataset:

D-E dataset (high-depth samples) geography variable:

```
DE_train <- as.data.frame(DE_pca_mat$x)
# extraction protocol:
groups<- as.factor(c("Denmark", "Denmark", "Denmark", "Denmark", "Denmark", "Denmark", "Denmark", "Denmark", "Denmark", "Denmark", "Denmark", "Denmark", "Denmark", "Denmark", "Denmark", "Denmark", "Denmark", "Denmark", "Denmark", "Denmark", "Denmark", "Denmark", "Denmark", "Denmark", "England", "England", "England", "England", "England", "England", "England", "England", "England", "England", "England"))

n <- nrow(DE_train)
# placeholder for storing the i-th prediction
preds <- rep(NA, n)
for(i in 1:n) {
    dataf.train <- DE_train[-i, ]
    dataf.test <- DE_train[i, ]
    groups_tmp <- groups[-i]
    set.seed(123) 
    cv.lasso <- cv.glmnet(as.matrix(dataf.train), groups_tmp, alpha = a, family = "binomial")
    glmnet.fit <- glmnet(as.matrix(dataf.train), groups_tmp, alpha = a, family = "binomial", lambda = cv.lasso$lambda.min)
    preds[i] <- predict(glmnet.fit, as.matrix(dataf.test), type="response")
}
```

```
## Warning in lognet(xd, is.sparse, ix, jx, y, weights, offset, alpha, nobs, : one
## multinomial or binomial class has fewer than 8 observations; dangerous ground

## Warning in lognet(xd, is.sparse, ix, jx, y, weights, offset, alpha, nobs, : one
## multinomial or binomial class has fewer than 8 observations; dangerous ground

## Warning in lognet(xd, is.sparse, ix, jx, y, weights, offset, alpha, nobs, : one
## multinomial or binomial class has fewer than 8 observations; dangerous ground

## Warning in lognet(xd, is.sparse, ix, jx, y, weights, offset, alpha, nobs, : one
## multinomial or binomial class has fewer than 8 observations; dangerous ground

## Warning in lognet(xd, is.sparse, ix, jx, y, weights, offset, alpha, nobs, : one
## multinomial or binomial class has fewer than 8 observations; dangerous ground

## Warning in lognet(xd, is.sparse, ix, jx, y, weights, offset, alpha, nobs, : one
## multinomial or binomial class has fewer than 8 observations; dangerous ground

## Warning in lognet(xd, is.sparse, ix, jx, y, weights, offset, alpha, nobs, : one
## multinomial or binomial class has fewer than 8 observations; dangerous ground

## Warning in lognet(xd, is.sparse, ix, jx, y, weights, offset, alpha, nobs, : one
## multinomial or binomial class has fewer than 8 observations; dangerous ground

## Warning in lognet(xd, is.sparse, ix, jx, y, weights, offset, alpha, nobs, : one
## multinomial or binomial class has fewer than 8 observations; dangerous ground

## Warning in lognet(xd, is.sparse, ix, jx, y, weights, offset, alpha, nobs, : one
## multinomial or binomial class has fewer than 8 observations; dangerous ground

## Warning in lognet(xd, is.sparse, ix, jx, y, weights, offset, alpha, nobs, : one
## multinomial or binomial class has fewer than 8 observations; dangerous ground
```

```
# define the number of elements in class 1
threshold <- seq(0, 1, length.out = 1000)
accuracy <- c()
for (t in threshold){
  tot <- 0
  for (i in 1:(length(preds))){
    if ((groups[i] == "Denmark" && preds[i] < (1 - t)) || (groups[i] == "England" && preds[i] > (t))){
      tot <- tot + 1
    }
  }
  prop <- tot/length(preds)
  accuracy <- c(accuracy, prop)
}  
DE_geo <- accuracy
```

D-E dataset (all samples) geography variable:

```
DE_all_train <- as.data.frame(DE_all_pca_mat$x)

# geo:
groups <- factor(c("Denmark", "Denmark", "Denmark", "Denmark", "Denmark", "Denmark", "Denmark", "Denmark", "Denmark", "Denmark", "Denmark", "Denmark", "Denmark", "Denmark", "Denmark", "Denmark", "Denmark", "Denmark", "Denmark", "Denmark", "Denmark", "Denmark", "Denmark", "Denmark", "Denmark", "Denmark", "Denmark", "Denmark", "Denmark", "England", "England",  "England", "England", "England", "England",  "England", "England", "England", "England",  "England", "England", "England", "England",  "England", "England", "England", "England",  "England", "England", "England", "England"))

n <- nrow(DE_all_train)
# placeholder for storing the i-th prediction
preds <- rep(NA, n)
for(i in 1:n) {
    dataf.train <- DE_all_train[-i, ]
    dataf.test <- DE_all_train[i, ]
    groups_tmp <- groups[-i]
    set.seed(123) 
    cv.lasso <- cv.glmnet(as.matrix(dataf.train), groups_tmp, alpha = a, family = "binomial")
    glmnet.fit <- glmnet(as.matrix(dataf.train), groups_tmp, alpha = a, family = "binomial", lambda = cv.lasso$lambda.min)
    preds[i] <- predict(glmnet.fit, as.matrix(dataf.test), type="response")
}

# define the number of elements in class 1
threshold <- seq(0, 1, length.out = 1000)
accuracy <- c()
for (t in threshold){
  tot <- 0
  for (i in 1:(length(preds))){
    if ((groups[i] == "Denmark" && preds[i] < (1 - t)) || (groups[i] == "England" && preds[i] > (t))){
      tot <- tot + 1
    }
  }
  prop <- tot/length(preds)
  accuracy <- c(accuracy, prop)
}  
DE_all_geo <- accuracy
```

Plot accuracy (comparison B-P dataset vs D-E dataset):

```
ggplot() + 
  geom_line(aes(threshold, BP_geo), color = "#7fc97f") +
  geom_line(aes(threshold, DE_geo), color = "#386cb0") +
  geom_line(aes(threshold, DE_all_geo), color = "#f0027f") +
  theme_bw() +
  ylim(0, 1)
```

# Streptomyces reads analysis:

Note: Keep only the samples with more then 100.000 reads

Read data into R:

```
strepto_mash <- read.csv("kmers_similarity_matrices/streptomyces_matrix.csv")
# Add row names
rownames(strepto_mash) <- names(strepto_mash)
```

Read data into R - number of reads assigned to streptomyces genus:

```
strepto_num_reads <- read.csv("kmers_similarity_matrices/Number_streptomyces_reads.csv", row.names = 1, header = T)
```

Filter out samples with less then 100.000 reads:

```
# initialize the filtered dataframes
strepto_filtered <- strepto_mash
strepto_num_reads_filtered <- strepto_num_reads
# select sample to remove
remove <- which(strepto_num_reads$Number_streptomyces_reads < 100000)
k <- remove[1]
count <- 0
for (i in remove){
  k <- i - count
  strepto_filtered <- strepto_filtered[-k,-k]
  strepto_num_reads_filtered <- strepto_num_reads_filtered[-k,]
  count <- count + 1
}
```

Keep only reference samples:

```
strepto_mash_mat <- strepto_filtered[4:35, 4:35]
strepto_num_reads_filtered <- strepto_num_reads_filtered[4:35,]
```

Create group info file:

```
groups <- strepto_num_reads_filtered$Location

strepto_col_list <- c()
strepto_col_list <- append(strepto_col_list, rep("#33a02c", length(which(strepto_num_reads_filtered$Location == "Brazil"))))
strepto_col_list <- append(strepto_col_list, rep("#1f78b4", length(which(strepto_num_reads_filtered$Location == "RapaNui"))))
```

MDS plot:

```
library(ggfortify)
strepto_pca_mat <- prcomp(strepto_mash_mat, scale. = TRUE)

autoplot(strepto_pca_mat, x = 1, y = 2, pch = 16, label = F, size = 6, label.size = 3, alpha = 0.7, label.colour = NULL, label.hjust=0.0001, label.vjust=-0.5,
          colour = strepto_col_list) +
     theme_bw()
```

Get training dataset:

```
# get the PCs dataframe
strepto_train <- as.data.frame(strepto_pca_mat$x)
# remove the last PC which explain 0% of the variance
strepto_train <- strepto_train[,1:31]
```

BP dataset, streptomyces reads (geography variable):

```
groups <- strepto_num_reads_filtered$Location

n <- nrow(strepto_train)
# placeholder for storing the i-th prediction
preds <- rep(NA, n)
for(i in 1:n) {
    dataf.strepto_train <- strepto_train[-i, ]
    dataf.test <- strepto_train[i, ]
    groups_tmp <- groups[-i]
    set.seed(123) 
    cv.lasso <- cv.glmnet(as.matrix(dataf.strepto_train), groups_tmp, alpha = a, family = "binomial")
    glmnet.fit <- glmnet(as.matrix(dataf.strepto_train), groups_tmp, alpha = a, family = "binomial", lambda = cv.lasso$lambda.min)
    preds[i] <- predict(glmnet.fit, as.matrix(dataf.test), type="response")
}

# define the number of elements in class 1
threshold <- seq(0, 1, length.out = 1000)
accuracy <- c()
for (t in threshold){
  tot <- 0
  for (i in 1:(length(preds))){
    if ((groups[i] == "Brazil" && preds[i] < (1 - t)) || (groups[i] == "RapaNui" && preds[i] > (t))){
      tot <- tot + 1
    }
  }
  prop <- tot/length(preds)
  accuracy <- c(accuracy, prop)
}  
st_geo <- accuracy
```

BP dataset, streptomyces reads (extraction protocol variable):

```
groups <- strepto_num_reads_filtered$extraction_protocol

n <- nrow(strepto_train)
# placeholder for storing the i-th prediction
preds <- rep(NA, n)
for(i in 1:n) {
    dataf.strepto_train <- strepto_train[-i, ]
    dataf.test <- strepto_train[i, ]
    groups_tmp <- groups[-i]
    set.seed(123) 
    cv.lasso <- cv.glmnet(as.matrix(dataf.strepto_train), groups_tmp, alpha = a, family = "binomial")
    glmnet.fit <- glmnet(as.matrix(dataf.strepto_train), groups_tmp, alpha = a, family = "binomial", lambda = cv.lasso$lambda.min)
    preds[i] <- predict(glmnet.fit, as.matrix(dataf.test), type="response")
}

# define the number of elements in class 1
threshold <- seq(0, 1, length.out = 1000)
accuracy <- c()
for (t in threshold){
  tot <- 0
  for (i in 1:(length(preds))){
    if ((groups[i] == "Allentoft" && preds[i] < (1 - t)) || (groups[i] == "Damgaard" && preds[i] > (t))){
      tot <- tot + 1
    }
  }
  prop <- tot/length(preds)
  accuracy <- c(accuracy, prop)
}  
st_extraction_protocol <- accuracy
```

BP dataset, streptomyces reads (sample type variable):

```
groups <- strepto_num_reads_filtered$sample_type

n <- nrow(strepto_train)
# placeholder for storing the i-th prediction
preds <- rep(NA, n)
for(i in 1:n) {
    dataf.strepto_train <- strepto_train[-i, ]
    dataf.test <- strepto_train[i, ]
    groups_tmp <- groups[-i]
    set.seed(123) 
    cv.lasso <- cv.glmnet(as.matrix(dataf.strepto_train), groups_tmp, alpha = a, family = "binomial")
    glmnet.fit <- glmnet(as.matrix(dataf.strepto_train), groups_tmp, alpha = a, family = "binomial", lambda = cv.lasso$lambda.min)
    preds[i] <- predict(glmnet.fit, as.matrix(dataf.test), type="response")
}

# define the number of elements in class 1
threshold <- seq(0, 1, length.out = 1000)
accuracy <- c()
for (t in threshold){
  tot <- 0
  for (i in 1:(length(preds))){
    if ((groups[i] == "Bone" && preds[i] < (1 - t)) || (groups[i] == "Tooth" && preds[i] > (t))){
      tot <- tot + 1
    }
  }
  prop <- tot/length(preds)
  accuracy <- c(accuracy, prop)
}  
st_sample_type <- accuracy
```

Comparing only geography (whole metagenome vs strempomyces reads):

```
ggplot() + 
  geom_line(aes(threshold, BP_geo), color = "#7fc97f") +
  geom_line(aes(threshold, st_geo), color = "#7fc97f", linetype = 2) +
  theme_bw() +
  ylim(0, 1)
```

Comparing only extraction protocol (whole metagenome vs strempomyces reads):

```
ggplot() + 
  geom_line(aes(threshold, BP_extraction_protocol), color = "#beaed4") +
  geom_line(aes(threshold, st_extraction_protocol), color = "#beaed4", linetype = 2) +
  theme_bw() +
  ylim(0, 1)
```

Comparing only sample type (whole metagenome vs strempomyces reads):

```
ggplot() + 
  geom_line(aes(threshold, BP_sample_type), color = "#fdc086") +
  geom_line(aes(threshold, st_sample_type), color = "#fdc086", linetype = 2) +
  theme_bw() +
  ylim(0, 1)
```

Plot accuracy curve for both metagenome-wide ans streptomyces-only anlyses (all variables together):

```
ggplot() + 
  geom_line(aes(threshold, BP_geo), color = "#7fc97f") +
  geom_line(aes(threshold, BP_extraction_protocol), color = "#beaed4") +
  geom_line(aes(threshold, BP_sample_type), color = "#fdc086") +
  geom_line(aes(threshold, st_geo), color = "#7fc97f", linetype = 2) +
  geom_line(aes(threshold, st_extraction_protocol), color = "#beaed4", linetype = 2) +
  geom_line(aes(threshold, st_sample_type), color = "#fdc086", linetype = 2) +
  theme_bw() +
  ylim(0, 1)
```

# Predicting target samples (with the model trained on B-P dataset whole metagenomes):

Define factor with the variable geography

```
groups<- as.factor(c("Brazil", "Brazil", "Brazil", "Brazil", "Brazil", "Brazil", "Brazil", "Brazil", "Brazil", "Brazil", "Brazil", "Brazil", "Brazil", "Brazil", "Brazil", "Brazil", "Brazil", "Brazil", "Brazil", "Brazil", "Brazil", "Brazil", "Brazil", "RapaNui", "RapaNui", "RapaNui", "RapaNui", "RapaNui", "RapaNui", "RapaNui", "RapaNui", "RapaNui", "RapaNui", "RapaNui", "RapaNui", "RapaNui", "RapaNui", "RapaNui"))
```

Predict Bot15 (B3b library) - probability of being Polynesian (RapaNui)

```
pred_mash <- read.csv("kmers_similarity_matrices/BP_similarity_matrix.csv")
# Add row names
rownames(pred_mash) <- names(pred_mash)
pred_mash$Bot17 <- NULL
pred_mash$MA572 <- NULL
pred_mash$MA575 <- NULL
pred_mash$MA577 <- NULL
pred_mash <- as.data.frame(t(pred_mash))
pred_mash$Bot17 <- NULL
pred_mash$MA572 <- NULL
pred_mash$MA575 <- NULL
pred_mash$MA577 <- NULL
# Transform to matrix object for plotting
pred_mash_mat <- as.matrix(pred_mash)
pred_pca_mat <- prcomp(pred_mash_mat, scale. = TRUE)
# get the PCs dataframe
pred <- as.data.frame(pred_pca_mat$x)
pred <- pred[,1:38]
# remove the last PC which explain 0% of the variance
train <- pred[2:39,1:38]
set.seed(123) 
cv.lasso <- cv.glmnet(as.matrix(train), groups, alpha = a, family = "binomial")
# Fit the final model on the training data
glmnet_model <- glmnet(as.matrix(train), groups, alpha = a, family = "binomial",
                lambda = cv.lasso$lambda.min)
# predict:
predict(glmnet_model, as.matrix(pred[1,1:38]), type="response")
```

```
##            s0
## B3b 0.1958771
```

Plot MDS with sample to predict:

```
autoplot(pred_pca_mat, x = 1, y = 3, pch = 16, label = F, size = 4, label.size = 3, alpha = 0.7, label.colour = NULL, label.hjust=0.0001, label.vjust=-0.5,
          colour = c("red", "#33a02c", "#33a02c", "#33a02c", "#33a02c", "#33a02c", "#33a02c", "#33a02c", "#33a02c", "#33a02c", "#33a02c", "#33a02c", "#33a02c", 
                     "#33a02c", "#33a02c", "#33a02c", "#33a02c", "#33a02c", "#33a02c", "#33a02c", "#33a02c", "#33a02c", "#33a02c", "#33a02c",
                     "#1f78b4", "#1f78b4", "#1f78b4", "#1f78b4", "#1f78b4", "#1f78b4", "#1f78b4", "#1f78b4", "#1f78b4", "#1f78b4", "#1f78b4", "#1f78b4", "#1f78b4", "#1f78b4", "#1f78b4")) +
     theme_bw()
```

Predict Bot17 - probability of being Polynesian (RapaNui)

```
pred_mash <- read.csv("kmers_similarity_matrices/BP_similarity_matrix.csv")
# Add row names
rownames(pred_mash) <- names(pred_mash)
pred_mash$B3b <- NULL
pred_mash$MA572 <- NULL
pred_mash$MA575 <- NULL
pred_mash$MA577 <- NULL
pred_mash <- as.data.frame(t(pred_mash))
pred_mash$B3b <- NULL
pred_mash$MA572 <- NULL
pred_mash$MA575 <- NULL
pred_mash$MA577 <- NULL
# Transform to matrix object for plotting
pred_mash_mat <- as.matrix(pred_mash)
pred_pca_mat <- prcomp(pred_mash_mat, scale. = TRUE)
# get the PCs dataframe
pred <- as.data.frame(pred_pca_mat$x)
pred <- pred[,1:38]
# remove the last PC which explain 0% of the variance
train <- pred[2:39,1:38]
set.seed(123) 
cv.lasso <- cv.glmnet(as.matrix(train), groups, alpha = a, family = "binomial")
# Fit the final model on the training data
glmnet_model <- glmnet(as.matrix(train), groups, alpha = a, family = "binomial",
                lambda = cv.lasso$lambda.min)
# predict:
predict(glmnet_model, as.matrix(pred[1,1:38]), type="response")
```

```
##                s0
## Bot17 0.007780429
```

Plot MDS with sample to predict:

```
autoplot(pred_pca_mat, x = 1, y = 3, pch = 16, label = F, size = 4, label.size = 3, alpha = 0.7, label.colour = NULL, label.hjust=0.0001, label.vjust=-0.5,
          colour = c("red", "#33a02c", "#33a02c", "#33a02c", "#33a02c", "#33a02c", "#33a02c", "#33a02c", "#33a02c", "#33a02c", "#33a02c", "#33a02c", "#33a02c", 
                     "#33a02c", "#33a02c", "#33a02c", "#33a02c", "#33a02c", "#33a02c", "#33a02c", "#33a02c", "#33a02c", "#33a02c", "#33a02c",
                     "#1f78b4", "#1f78b4", "#1f78b4", "#1f78b4", "#1f78b4", "#1f78b4", "#1f78b4", "#1f78b4", "#1f78b4", "#1f78b4", "#1f78b4", "#1f78b4", "#1f78b4", "#1f78b4", "#1f78b4")) +
     theme_bw()
```

Predict MA572 - probability of being Polynesian (RapaNui)

```
pred_mash <- read.csv("kmers_similarity_matrices/BP_similarity_matrix.csv")
# Add row names
rownames(pred_mash) <- names(pred_mash)
pred_mash$B3b <- NULL
pred_mash$Bot17 <- NULL
pred_mash$MA575 <- NULL
pred_mash$MA577 <- NULL
pred_mash <- as.data.frame(t(pred_mash))
pred_mash$B3b <- NULL
pred_mash$Bot17 <- NULL
pred_mash$MA575 <- NULL
pred_mash$MA577 <- NULL
# Transform to matrix object for plotting
pred_mash_mat <- as.matrix(pred_mash)
pred_pca_mat <- prcomp(pred_mash_mat, scale. = TRUE)
# get the PCs dataframe
pred <- as.data.frame(pred_pca_mat$x)
pred <- pred[,1:38]
# remove the last PC which explain 0% of the variance
train <- pred[2:39,1:38]
set.seed(123) 
cv.lasso <- cv.glmnet(as.matrix(train), groups, alpha = a, family = "binomial")
# Fit the final model on the training data
glmnet_model <- glmnet(as.matrix(train), groups, alpha = a, family = "binomial",
                lambda = cv.lasso$lambda.min)
# predict:
predict(glmnet_model, as.matrix(pred[1,1:38]), type="response")
```

```
##              s0
## MA572 0.1284461
```

Plot MDS with sample to predict:

```
autoplot(pred_pca_mat, x = 1, y = 3, pch = 16, label = F, size = 4, label.size = 3, alpha = 0.7, label.colour = NULL, label.hjust=0.0001, label.vjust=-0.5,
          colour = c("red", "#33a02c", "#33a02c", "#33a02c", "#33a02c", "#33a02c", "#33a02c", "#33a02c", "#33a02c", "#33a02c", "#33a02c", "#33a02c", "#33a02c", 
                     "#33a02c", "#33a02c", "#33a02c", "#33a02c", "#33a02c", "#33a02c", "#33a02c", "#33a02c", "#33a02c", "#33a02c", "#33a02c",
                     "#1f78b4", "#1f78b4", "#1f78b4", "#1f78b4", "#1f78b4", "#1f78b4", "#1f78b4", "#1f78b4", "#1f78b4", "#1f78b4", "#1f78b4", "#1f78b4", "#1f78b4", "#1f78b4", "#1f78b4")) +
     theme_bw()
```

Predict MA575 - probability of being Polynesian (RapaNui)

```
pred_mash <- read.csv("kmers_similarity_matrices/BP_similarity_matrix.csv")
# Add row names
rownames(pred_mash) <- names(pred_mash)
pred_mash$B3b <- NULL
pred_mash$Bot17 <- NULL
pred_mash$MA572 <- NULL
pred_mash$MA577 <- NULL
pred_mash <- as.data.frame(t(pred_mash))
pred_mash$B3b <- NULL
pred_mash$Bot17 <- NULL
pred_mash$MA572 <- NULL
pred_mash$MA577 <- NULL
# Transform to matrix object for plotting
pred_mash_mat <- as.matrix(pred_mash)
pred_pca_mat <- prcomp(pred_mash_mat, scale. = TRUE)
# get the PCs dataframe
pred <- as.data.frame(pred_pca_mat$x)
pred <- pred[,1:38]
# remove the last PC which explain 0% of the variance
train <- pred[2:39,1:38]
set.seed(123) 
cv.lasso <- cv.glmnet(as.matrix(train), groups, alpha = a, family = "binomial")
# Fit the final model on the training data
glmnet_model <- glmnet(as.matrix(train), groups, alpha = a, family = "binomial",
                lambda = cv.lasso$lambda.min)
# predict:
predict(glmnet_model, as.matrix(pred[1,1:38]), type="response")
```

```
##             s0
## MA575 0.202075
```

Plot MDS with sample to predict:

```
autoplot(pred_pca_mat, x = 1, y = 3, pch = 16, label = F, size = 4, label.size = 3, alpha = 0.7, label.colour = NULL, label.hjust=0.0001, label.vjust=-0.5,
          colour = c("red", "#33a02c", "#33a02c", "#33a02c", "#33a02c", "#33a02c", "#33a02c", "#33a02c", "#33a02c", "#33a02c", "#33a02c", "#33a02c", "#33a02c", 
                     "#33a02c", "#33a02c", "#33a02c", "#33a02c", "#33a02c", "#33a02c", "#33a02c", "#33a02c", "#33a02c", "#33a02c", "#33a02c",
                     "#1f78b4", "#1f78b4", "#1f78b4", "#1f78b4", "#1f78b4", "#1f78b4", "#1f78b4", "#1f78b4", "#1f78b4", "#1f78b4", "#1f78b4", "#1f78b4", "#1f78b4", "#1f78b4", "#1f78b4")) +
     theme_bw()
```

Predict MA577 - probability of being Polynesian (RapaNui)

```
pred_mash <- read.csv("kmers_similarity_matrices/BP_similarity_matrix.csv")
# Add row names
rownames(pred_mash) <- names(pred_mash)
pred_mash$B3b <- NULL
pred_mash$Bot17 <- NULL
pred_mash$MA572 <- NULL
pred_mash$MA575 <- NULL
pred_mash <- as.data.frame(t(pred_mash))
pred_mash$B3b <- NULL
pred_mash$Bot17 <- NULL
pred_mash$MA572 <- NULL
pred_mash$MA575 <- NULL
# Transform to matrix object for plotting
pred_mash_mat <- as.matrix(pred_mash)
pred_pca_mat <- prcomp(pred_mash_mat, scale. = TRUE)
# get the PCs dataframe
pred <- as.data.frame(pred_pca_mat$x)
pred <- pred[,1:38]
# remove the last PC which explain 0% of the variance
train <- pred[2:39,1:38]
set.seed(123) 
cv.lasso <- cv.glmnet(as.matrix(train), groups, alpha = a, family = "binomial")
# Fit the final model on the training data
glmnet_model <- glmnet(as.matrix(train), groups, alpha = a, family = "binomial",
                lambda = cv.lasso$lambda.min)
# predict:
predict(glmnet_model, as.matrix(pred[1,1:38]), type="response")
```

```
##              s0
## MA577 0.1832414
```

Plot MDS with sample to predict:

```
autoplot(pred_pca_mat, x = 1, y = 3, pch = 16, label = F, size = 4, label.size = 3, alpha = 0.7, label.colour = NULL, label.hjust=0.0001, label.vjust=-0.5,
          colour = c("red", "#33a02c", "#33a02c", "#33a02c", "#33a02c", "#33a02c", "#33a02c", "#33a02c", "#33a02c", "#33a02c", "#33a02c", "#33a02c", "#33a02c", 
                     "#33a02c", "#33a02c", "#33a02c", "#33a02c", "#33a02c", "#33a02c", "#33a02c", "#33a02c", "#33a02c", "#33a02c", "#33a02c",
                     "#1f78b4", "#1f78b4", "#1f78b4", "#1f78b4", "#1f78b4", "#1f78b4", "#1f78b4", "#1f78b4", "#1f78b4", "#1f78b4", "#1f78b4", "#1f78b4", "#1f78b4", "#1f78b4", "#1f78b4")) +
     theme_bw()
```

# Predicting target samples (with the model trained on B-P dataset Streptomyces reads):

Define vector with the variable geography:

```
groups <- strepto_num_reads_filtered$Location
```

Predict Bot15 (B3b library) - probability of being Polynesian (RapaNui)

```
pred_strepto_mash <- strepto_filtered
# Add row names
rownames(pred_strepto_mash) <- names(pred_strepto_mash)
pred_strepto_mash$B3a <- NULL
pred_strepto_mash$Bot17 <- NULL
pred_strepto_mash <- as.data.frame(t(pred_strepto_mash))
pred_strepto_mash$B3a <- NULL
pred_strepto_mash$Bot17 <- NULL
# Transform to matrix object for plotting
pred_strepto_mash_mat <- as.matrix(pred_strepto_mash)
pred_strepto_pca_mat <- prcomp(pred_strepto_mash_mat, scale. = TRUE)
# get the PCs dataframe
pred_strepto <- as.data.frame(pred_strepto_pca_mat$x)
pred_strepto <- pred_strepto[,1:32]
# remove the last PC which explain 0% of the variance
train <- pred_strepto[2:33,1:32]
set.seed(123) 
cv.lasso <- cv.glmnet(as.matrix(train), groups, alpha = a, family = "binomial")
# Fit the final model on the training data
glmnet_model <- glmnet(as.matrix(train), groups, alpha = a, family = "binomial",
                lambda = cv.lasso$lambda.min)
# predict:
predict(glmnet_model, as.matrix(pred_strepto[1,1:32]), type="response")
```

```
##            s0
## B3b 0.4855316
```

MDS plot with sample to predict:

```
strepto_pred_col_list <- c("red")
strepto_pred_col_list <- append(strepto_pred_col_list, rep("#33a02c", length(which(strepto_num_reads_filtered$Location == "Brazil"))))
strepto_pred_col_list <- append(strepto_pred_col_list, rep("#1f78b4", length(which(strepto_num_reads_filtered$Location == "RapaNui"))))

autoplot(pred_strepto_pca_mat, x = 1, y = 3, pch = 16, label = F, size = 4, label.size = 3, alpha = 0.7, label.colour = NULL, label.hjust=0.0001, label.vjust=-0.5,
          colour = strepto_pred_col_list) +
     theme_bw()
```

Predict Bot17 - probability of being Polynesian (RapaNui)

```
pred_strepto_mash <- strepto_filtered
# Add row names
rownames(pred_strepto_mash) <- names(pred_strepto_mash)
pred_strepto_mash$B3a <- NULL
pred_strepto_mash$B3b <- NULL
pred_strepto_mash <- as.data.frame(t(pred_strepto_mash))
pred_strepto_mash$B3a <- NULL
pred_strepto_mash$B3b <- NULL
# Transform to matrix object for plotting
pred_strepto_mash_mat <- as.matrix(pred_strepto_mash)
pred_strepto_pca_mat <- prcomp(pred_strepto_mash_mat, scale. = TRUE)
# get the PCs dataframe
pred_strepto <- as.data.frame(pred_strepto_pca_mat$x)
pred_strepto <- pred_strepto[,1:32]
# remove the last PC which explain 0% of the variance
train <- pred_strepto[2:33,1:32]
set.seed(123) 
cv.lasso <- cv.glmnet(as.matrix(train), groups, alpha = a, family = "binomial")
# Fit the final model on the training data
glmnet_model <- glmnet(as.matrix(train), groups, alpha = a, family = "binomial",
                lambda = cv.lasso$lambda.min)
# predict:
predict(glmnet_model, as.matrix(pred_strepto[1,1:32]), type="response")
```

```
##             s0
## Bot17 0.327659
```

MDS plot with sample to predict:

```
strepto_pred_col_list <- c("red")
strepto_pred_col_list <- append(strepto_pred_col_list, rep("#33a02c", length(which(strepto_num_reads_filtered$Location == "Brazil"))))
strepto_pred_col_list <- append(strepto_pred_col_list, rep("#1f78b4", length(which(strepto_num_reads_filtered$Location == "RapaNui"))))

autoplot(pred_strepto_pca_mat, x = 1, y = 3, pch = 16, label = F, size = 4, label.size = 3, alpha = 0.7, label.colour = NULL, label.hjust=0.0001, label.vjust=-0.5,
          colour = strepto_pred_col_list) +
     theme_bw()
```

Test the effect of sample removal oon the whole metagenome analysis (BP\_dataset)

Read BP data into R:

```
few_BP_mash <- read.csv("kmers_similarity_matrices/BP_few_samples.csv")
# Add row names
rownames(few_BP_mash) <- names(few_BP_mash)
few_BP_mash_mat <- as.matrix(few_BP_mash)
```

MDS plot:

```
library(ggfortify)
few_BP_pca_mat <- prcomp(few_BP_mash_mat, scale. = TRUE)

few_BD_col_vec <- c("#33a02c",  "#33a02c",  "#33a02c",  "#33a02c",  "#33a02c",  "#33a02c",  "#33a02c",  "#33a02c",  "#33a02c",  "#33a02c",  "#33a02c",  "#33a02c",  "#33a02c",  "#33a02c",  "#33a02c",  "#33a02c",  "#33a02c",  "#1f78b4",  "#1f78b4",  "#1f78b4",  "#1f78b4",  "#1f78b4",  "#1f78b4",  "#1f78b4",  "#1f78b4",  "#1f78b4",  "#1f78b4",  "#1f78b4",  "#1f78b4",  "#1f78b4",  "#1f78b4",  "#1f78b4")

autoplot(few_BP_pca_mat, x = 1, y = 2, pch = 16, label = F, size = 6, label.size = 3, alpha = 0.7, label.colour = NULL, label.hjust=0.0001, label.vjust=-0.5,
          colour = few_BD_col_vec) +
     theme_bw()
```

Get training dataset:

```
# get the PCs dataframe
few_BP_train <- as.data.frame(few_BP_pca_mat$x)
# remove the last PC which explain 0% of the variance
few_BP_train <- few_BP_train[,1:31]
```

Select type of regularization (0 = ridge, 1 = lasso, 0<a<1 = elastic net):

```
library(glmnet)
a = 1
```

Geography variable:

```
groups<- as.factor(c("Brazil",  "Brazil",   "Brazil",   "Brazil",   "Brazil",   "Brazil",   "Brazil",   "Brazil",   "Brazil",   "Brazil",   "Brazil",   "Brazil",   "Brazil",   "Brazil",   "Brazil",   "Brazil",   "Brazil",   "RapaNui",  "RapaNui",  "RapaNui",  "RapaNui",  "RapaNui",  "RapaNui",  "RapaNui",  "RapaNui",  "RapaNui",  "RapaNui",  "RapaNui",  "RapaNui",  "RapaNui",  "RapaNui",  "RapaNui"))

n <- nrow(few_BP_train)
# placeholder for storing the i-th prediction
preds <- rep(NA, n)
for(i in 1:n) {
    dataf.few_BP_train <- few_BP_train[-i, ]
    dataf.test <- few_BP_train[i, ]
    groups_tmp <- groups[-i]
    set.seed(123) 
    cv.lasso <- cv.glmnet(as.matrix(dataf.few_BP_train), groups_tmp, alpha = a, family = "binomial")
    glmnet.fit <- glmnet(as.matrix(dataf.few_BP_train), groups_tmp, alpha = a, family = "binomial", lambda = cv.lasso$lambda.min)
    preds[i] <- predict(glmnet.fit, as.matrix(dataf.test), type="response")
}

# define the number of elements in class 1
threshold <- seq(0, 1, length.out = 1000)
accuracy <- c()
for (t in threshold){
  tot <- 0
  for (i in 1:(length(preds))){
    if ((groups[i] == "Brazil" && preds[i] < (1 - t)) || (groups[i] == "RapaNui" && preds[i] > (t))){
      tot <- tot + 1
    }
  }
  prop <- tot/length(preds)
  accuracy <- c(accuracy, prop)
}  
few_BP_geo <- accuracy
```

Extraction protocol variable:

```
groups<- as.factor(c("Allentoft",   "Allentoft",    "Allentoft",    "Allentoft",    "Allentoft",    "Allentoft",    "Allentoft",    "Allentoft",    "Allentoft",    "Allentoft",    "Allentoft",    "Allentoft",    "Allentoft",    "Allentoft",    "Allentoft",    "Allentoft",    "Allentoft",    "Allentoft",    "Allentoft",    "Allentoft",    "Allentoft",    "Damgaard", "Damgaard", "Damgaard", "Damgaard", "Damgaard", "Damgaard", "Damgaard", "Damgaard", "Damgaard", "Damgaard", "Damgaard"))

n <- nrow(few_BP_train)
# placeholder for storing the i-th prediction
preds <- rep(NA, n)
for(i in 1:n) {
    dataf.few_BP_train <- few_BP_train[-i, ]
    dataf.test <- few_BP_train[i, ]
    groups_tmp <- groups[-i]
    set.seed(123) 
    cv.lasso <- cv.glmnet(as.matrix(dataf.few_BP_train), groups_tmp, alpha = a, family = "binomial")
    glmnet.fit <- glmnet(as.matrix(dataf.few_BP_train), groups_tmp, alpha = a, family = "binomial", lambda = cv.lasso$lambda.min)
    preds[i] <- predict(glmnet.fit, as.matrix(dataf.test), type="response")
}

# define the number of elements in class 1
threshold <- seq(0, 1, length.out = 1000)
accuracy <- c()
for (t in threshold){
  tot <- 0
  for (i in 1:(length(preds))){
    if ((groups[i] == "Allentoft" && preds[i] < (1 - t)) || (groups[i] == "Damgaard" && preds[i] > (t))){
      tot <- tot + 1
    }
  }
  prop <- tot/length(preds)
  accuracy <- c(accuracy, prop)
}  
few_BP_extraction_protocol <- accuracy
```

Sample type variable:

```
groups<- as.factor(c("Tooth", "Tooth", "Bone", "Tooth", "Tooth", "Tooth", "Tooth", "Tooth", "Tooth", "Tooth", "Tooth", "Tooth", "Tooth", "Tooth", "Tooth", "Tooth", "Tooth", "Tooth", "Tooth", "Tooth", "Tooth", "Bone", "Bone", "Bone", "Bone", "Bone", "Bone", "Bone", "Bone", "Bone", "Bone", "Bone"))

n <- nrow(few_BP_train)
# placeholder for storing the i-th prediction
preds <- rep(NA, n)
for(i in 1:n) {
    dataf.few_BP_train <- few_BP_train[-i, ]
    dataf.test <- few_BP_train[i, ]
    groups_tmp <- groups[-i]
    set.seed(123) 
    cv.lasso <- cv.glmnet(as.matrix(dataf.few_BP_train), groups_tmp, alpha = a, family = "binomial")
    glmnet.fit <- glmnet(as.matrix(dataf.few_BP_train), groups_tmp, alpha = a, family = "binomial", lambda = cv.lasso$lambda.min)
    preds[i] <- predict(glmnet.fit, as.matrix(dataf.test), type="response")
}

# define the number of elements in class 1
threshold <- seq(0, 1, length.out = 1000)
accuracy <- c()
for (t in threshold){
  tot <- 0
  for (i in 1:(length(preds))){
    if ((groups[i] == "Bone" && preds[i] < (1 - t)) || (groups[i] == "Tooth" && preds[i] > (t))){
      tot <- tot + 1
    }
  }
  prop <- tot/length(preds)
  accuracy <- c(accuracy, prop)
}  
few_BP_sample_type <- accuracy
```

Plot accuracy curve for the subsampled dataset:

```
ggplot() + 
  geom_line(aes(threshold, few_BP_geo), color = "#7fc97f") +
  geom_line(aes(threshold, few_BP_extraction_protocol), color = "#beaed4") +
  geom_line(aes(threshold, few_BP_sample_type), color = "#fdc086") +
  theme_bw() +
  ylim(0, 1)
```

Plot accuracy curve for the subsampled dataset (with negative control):

```
ggplot() + 
  geom_line(aes(threshold, few_BP_geo), color = "#7fc97f") +
  geom_line(aes(threshold, few_BP_extraction_protocol), color = "#beaed4") +
  geom_line(aes(threshold, few_BP_sample_type), color = "#fdc086") +
  geom_line(aes(threshold, BP_neg_ctrl), color = "#fb9a99") +
  theme_bw() +
  ylim(0, 1)
```

Comparing only geography (whole metagenome (all samples) vs whole metagenome (only samples used for streptomyces analysis):

```
ggplot() + 
  geom_line(aes(threshold, few_BP_geo), color = "#7fc97f") +
  geom_line(aes(threshold, st_geo), color = "#7fc97f", linetype = 2) +
  theme_bw() +
  ylim(0, 1)
```

Comparing only extraction protocol (whole metagenome (all samples) vs whole metagenome (only samples used for streptomyces analysis):

```
ggplot() + 
  geom_line(aes(threshold, few_BP_extraction_protocol), color = "#beaed4") +
  geom_line(aes(threshold, st_extraction_protocol), color = "#beaed4", linetype = 2) +
  theme_bw() +
  ylim(0, 1)
```

Comparing only sample type (whole metagenome (all samples) vs whole metagenome (only samples used for streptomyces analysis):

```
ggplot() + 
  geom_line(aes(threshold, few_BP_sample_type), color = "#fdc086") +
  geom_line(aes(threshold, st_sample_type), color = "#fdc086", linetype = 2) +
  theme_bw() +
  ylim(0, 1)
```

BP\_dataset subsampled to ~661k reads (661375 reads)

Read BP (661k version) data into R:

```
# import data
BP661k_mash <- read.csv("kmers_similarity_matrices/sub661k_few.csv")
# Add row names
rownames(BP661k_mash) <- names(BP661k_mash)
# Transform to matrix object for plotting
BP661k_mash_mat <- as.matrix(BP661k_mash)
```

Remove target samples

```
BP661k_mash_mat <- BP661k_mash_mat[4:35, 4:35]
```

BP (661k version) dataset MDS

```
library(ggfortify)
BP661k_pca_mat <- prcomp(BP661k_mash_mat, scale. = TRUE)
```

Plot: (color according to geography) green = Brazil blue = Polynesia (RapaNui)

```
BP661k_col_vec <- c("#33a02c",  "#33a02c",  "#33a02c",  "#33a02c",  "#33a02c",  "#33a02c",  "#33a02c",  "#33a02c",  "#33a02c",  "#33a02c",  "#33a02c",  "#33a02c",  "#33a02c",  "#33a02c",  "#33a02c",  "#33a02c",  "#33a02c",  "#1f78b4",  "#1f78b4",  "#1f78b4",  "#1f78b4",  "#1f78b4",  "#1f78b4",  "#1f78b4",  "#1f78b4",  "#1f78b4",  "#1f78b4",  "#1f78b4",  "#1f78b4",  "#1f78b4",  "#1f78b4",  "#1f78b4")

autoplot(BP661k_pca_mat, x = 1, y = 2, pch = 16, label = T, size = 4, label.size = 3, alpha = 0.7, label.colour = NULL, label.hjust=0.0001, label.vjust=-0.5,
          colour = BP661k_col_vec) +
     theme_bw()
```

Get training dataset

```
# get the PCs dataframe
BP661k_train <- as.data.frame(BP661k_pca_mat$x)
# remove the last PC which explain 0% of the variance
BP661k_train <- BP661k_train[,1:31]
```

Select type of regularization (0 = ridge, 1 = lasso, 0<a<1 = elastic net)

```
library(glmnet)
a = 1
```

Geography variable:

```
groups<- as.factor(c("Brazil",  "Brazil",   "Brazil",   "Brazil",   "Brazil",   "Brazil",   "Brazil",   "Brazil",   "Brazil",   "Brazil",   "Brazil",   "Brazil",   "Brazil",   "Brazil",   "Brazil",   "Brazil",   "Brazil",   "RapaNui",  "RapaNui",  "RapaNui",  "RapaNui",  "RapaNui",  "RapaNui",  "RapaNui",  "RapaNui",  "RapaNui",  "RapaNui",  "RapaNui",  "RapaNui",  "RapaNui",  "RapaNui",  "RapaNui"))

n <- nrow(BP661k_train)
# placeholder for storing the i-th prediction
preds <- rep(NA, n)
for(i in 1:n) {
    dataf.BP661k_train <- BP661k_train[-i, ]
    dataf.test <- BP661k_train[i, ]
    groups_tmp <- groups[-i]
    set.seed(123) 
    cv.lasso <- cv.glmnet(as.matrix(dataf.BP661k_train), groups_tmp, alpha = a, family = "binomial")
    glmnet.fit <- glmnet(as.matrix(dataf.BP661k_train), groups_tmp, alpha = a, family = "binomial", lambda = cv.lasso$lambda.min)
    preds[i] <- predict(glmnet.fit, as.matrix(dataf.test), type="response")
}

# define the number of elements in class 1
threshold <- seq(0, 1, length.out = 1000)
accuracy <- c()
for (t in threshold){
  tot <- 0
  for (i in 1:(length(preds))){
    if ((groups[i] == "Brazil" && preds[i] < (1 - t)) || (groups[i] == "RapaNui" && preds[i] > (t))){
      tot <- tot + 1
    }
  }
  prop <- tot/length(preds)
  accuracy <- c(accuracy, prop)
}  
BP661k_geo <- accuracy
```

Extraction protocol variable:

```
groups<- as.factor(c("Allentoft",   "Allentoft",    "Allentoft",    "Allentoft",    "Allentoft",    "Allentoft",    "Allentoft",    "Allentoft",    "Allentoft",    "Allentoft",    "Allentoft",    "Allentoft",    "Allentoft",    "Allentoft",    "Allentoft",    "Allentoft",    "Allentoft",    "Allentoft",    "Allentoft",    "Allentoft",    "Damgaard", "Damgaard", "Damgaard", "Damgaard", "Damgaard", "Damgaard", "Damgaard", "Damgaard", "Damgaard", "Damgaard", "Damgaard", "Damgaard"))

n <- nrow(BP661k_train)
# placeholder for storing the i-th prediction
preds <- rep(NA, n)
for(i in 1:n) {
    dataf.BP661k_train <- BP661k_train[-i, ]
    dataf.test <- BP661k_train[i, ]
    groups_tmp <- groups[-i]
    set.seed(123) 
    cv.lasso <- cv.glmnet(as.matrix(dataf.BP661k_train), groups_tmp, alpha = a, family = "binomial")
    glmnet.fit <- glmnet(as.matrix(dataf.BP661k_train), groups_tmp, alpha = a, family = "binomial", lambda = cv.lasso$lambda.min)
    preds[i] <- predict(glmnet.fit, as.matrix(dataf.test), type="response")
}

# define the number of elements in class 1
threshold <- seq(0, 1, length.out = 1000)
accuracy <- c()
for (t in threshold){
  tot <- 0
  for (i in 1:(length(preds))){
    if ((groups[i] == "Allentoft" && preds[i] < (1 - t)) || (groups[i] == "Damgaard" && preds[i] > (t))){
      tot <- tot + 1
    }
  }
  prop <- tot/length(preds)
  accuracy <- c(accuracy, prop)
}  
BP661k_extraction_protocol <- accuracy
```

Sample type variable:

```
groups<- as.factor(c("Tooth", "Tooth", "Bone", "Tooth", "Tooth", "Tooth", "Tooth", "Tooth", "Tooth", "Tooth", "Tooth", "Tooth", "Tooth", "Tooth", "Tooth", "Tooth", "Tooth", "Tooth", "Tooth", "Tooth", "Tooth", "Bone", "Bone", "Bone", "Bone", "Bone", "Bone", "Bone", "Bone", "Bone", "Bone", "Bone"))

n <- nrow(BP661k_train)
# placeholder for storing the i-th prediction
preds <- rep(NA, n)
for(i in 1:n) {
    dataf.BP661k_train <- BP661k_train[-i, ]
    dataf.test <- BP661k_train[i, ]
    groups_tmp <- groups[-i]
    set.seed(123) 
    cv.lasso <- cv.glmnet(as.matrix(dataf.BP661k_train), groups_tmp, alpha = a, family = "binomial")
    glmnet.fit <- glmnet(as.matrix(dataf.BP661k_train), groups_tmp, alpha = a, family = "binomial", lambda = cv.lasso$lambda.min)
    preds[i] <- predict(glmnet.fit, as.matrix(dataf.test), type="response")
}

# define the number of elements in class 1
threshold <- seq(0, 1, length.out = 1000)
accuracy <- c()
for (t in threshold){
  tot <- 0
  for (i in 1:(length(preds))){
    if ((groups[i] == "Bone" && preds[i] < (1 - t)) || (groups[i] == "Tooth" && preds[i] > (t))){
      tot <- tot + 1
    }
  }
  prop <- tot/length(preds)
  accuracy <- c(accuracy, prop)
}  
BP661k_sample_type <- accuracy
```

Comparing only geography (whole metagenome (all samples) vs whole metagenome (subsample to ~661k reads):

```
ggplot() + 
  geom_line(aes(threshold, BP661k_geo), color = "#7fc97f") +
  geom_line(aes(threshold, st_geo), color = "#7fc97f", linetype = 2) +
  theme_bw() +
  ylim(0, 1)
```

Comparing only extraction protocol (whole metagenome (all samples) vs whole metagenome (subsample to ~661k reads):

```
ggplot() + 
  geom_line(aes(threshold, BP661k_extraction_protocol), color = "#beaed4") +
  geom_line(aes(threshold, st_extraction_protocol), color = "#beaed4", linetype = 2) +
  theme_bw() +
  ylim(0, 1)
```

Comparing only sample type (whole metagenome vs whole metagenome (subsample to ~661k reads):

```
ggplot() + 
  geom_line(aes(threshold, BP661k_sample_type), color = "#fdc086") +
  geom_line(aes(threshold, st_sample_type), color = "#fdc086", linetype = 2) +
  theme_bw() +
  ylim(0, 1)
```

# Comparing the logit performancens using different k-mer sizes:

# 11bp

Read BP data into R:

```
BP11_mash <- read.csv("kmer_size_comparison/BP_11bp.csv")
# Add row names
rownames(BP11_mash) <- names(BP11_mash)
# Transform to matrix object for plotting
BP11_mash_mat <- as.matrix(BP11_mash)
```

BP dataset MDS

```
library(ggfortify)
BP11_pca_mat <- prcomp(BP11_mash_mat, scale. = TRUE)
```

Plot: (color according to geography) green = Brazil blue = Polynesia (RapaNui)

```
BP11_col_vec <- c("#33a02c", "#33a02c", "#33a02c", "#33a02c", "#33a02c", "#33a02c", "#33a02c", "#33a02c", "#33a02c", "#33a02c", "#33a02c", "#33a02c", "#33a02c", "#33a02c", "#33a02c", "#33a02c", "#33a02c", "#33a02c", "#33a02c", "#33a02c", "#33a02c", "#33a02c", "#33a02c", "#1f78b4", "#1f78b4", "#1f78b4", "#1f78b4", "#1f78b4", "#1f78b4", "#1f78b4", "#1f78b4", "#1f78b4", "#1f78b4", "#1f78b4", "#1f78b4", "#1f78b4", "#1f78b4", "#1f78b4")

autoplot(BP11_pca_mat, x = 1, y = 2, pch = 16, label = F, size = 4, label.size = 3, alpha = 0.7, label.colour = NULL, label.hjust=0.0001, label.vjust=-0.5,
          colour = BP11_col_vec) +
     theme_bw()
```

```
autoplot(BP11_pca_mat, x = 1, y = 3, pch = 16, label = F, size = 4, label.size = 3, alpha = 0.7, label.colour = NULL, label.hjust=0.0001, label.vjust=-0.5,
          colour = BP11_col_vec) +
     theme_bw()
```

# Model training and accuracy evalutation B-P dataset:

Select type of regularization (0 = ridge, 1 = lasso, 0<a<1 = elastic net):

```
library(glmnet)
a = 1
```

Get training dataset:

```
# get the PCs dataframe
BP11_train <- as.data.frame(BP11_pca_mat$x)
# remove the last PC which explain 0% of the variance
BP11_train <- BP11_train[,1:37]
```

Geography variable:

```
groups<- as.factor(c("Brazil", "Brazil", "Brazil", "Brazil", "Brazil", "Brazil", "Brazil", "Brazil", "Brazil", "Brazil", "Brazil", "Brazil", "Brazil", "Brazil", "Brazil", "Brazil", "Brazil", "Brazil", "Brazil", "Brazil", "Brazil", "Brazil", "Brazil", "RapaNui", "RapaNui", "RapaNui", "RapaNui", "RapaNui", "RapaNui", "RapaNui", "RapaNui", "RapaNui", "RapaNui", "RapaNui", "RapaNui", "RapaNui", "RapaNui", "RapaNui"))

n <- nrow(BP11_train)
# placeholder for storing the i-th prediction
preds <- rep(NA, n)
for(i in 1:n) {
    dataf.BP11_train <- BP11_train[-i, ]
    dataf.test <- BP11_train[i, ]
    groups_tmp <- groups[-i]
    set.seed(123) 
    cv.lasso <- cv.glmnet(as.matrix(dataf.BP11_train), groups_tmp, alpha = a, family = "binomial")
    glmnet.fit <- glmnet(as.matrix(dataf.BP11_train), groups_tmp, alpha = a, family = "binomial", lambda = cv.lasso$lambda.min)
    preds[i] <- predict(glmnet.fit, as.matrix(dataf.test), type="response")
}

# define the number of elements in class 1
threshold <- seq(0, 1, length.out = 1000)
accuracy <- c()
for (t in threshold){
  tot <- 0
  for (i in 1:(length(preds))){
    if ((groups[i] == "Brazil" && preds[i] < (1 - t)) || (groups[i] == "RapaNui" && preds[i] > (t))){
      tot <- tot + 1
    }
  }
  prop <- tot/length(preds)
  accuracy <- c(accuracy, prop)
}  
BP11_geo <- accuracy
```

Extraction protocol variable:

```
groups<- as.factor(c("Allentoft", "Allentoft", "Allentoft", "Allentoft", "Allentoft", "Allentoft", "Allentoft", "Allentoft", "Allentoft", "Allentoft", "Allentoft", "Allentoft", "Allentoft", "Allentoft", "Allentoft", "Allentoft", "Allentoft", "Allentoft", "Allentoft", "Allentoft", "Allentoft", "Allentoft", "Allentoft", "Allentoft", "Allentoft", "Allentoft", "Allentoft", "Damgaard", "Damgaard", "Damgaard", "Damgaard", "Damgaard", "Damgaard", "Damgaard", "Damgaard", "Damgaard", "Damgaard", "Damgaard"))

n <- nrow(BP11_train)
# placeholder for storing the i-th prediction
preds <- rep(NA, n)
for(i in 1:n) {
    dataf.BP11_train <- BP11_train[-i, ]
    dataf.test <- BP11_train[i, ]
    groups_tmp <- groups[-i]
    set.seed(123) 
    cv.lasso <- cv.glmnet(as.matrix(dataf.BP11_train), groups_tmp, alpha = a, family = "binomial")
    glmnet.fit <- glmnet(as.matrix(dataf.BP11_train), groups_tmp, alpha = a, family = "binomial", lambda = cv.lasso$lambda.min)
    preds[i] <- predict(glmnet.fit, as.matrix(dataf.test), type="response")
}
```

```
## Warning in lognet(xd, is.sparse, ix, jx, y, weights, offset, alpha, nobs, : one
## multinomial or binomial class has fewer than 8 observations; dangerous ground

## Warning in lognet(xd, is.sparse, ix, jx, y, weights, offset, alpha, nobs, : one
## multinomial or binomial class has fewer than 8 observations; dangerous ground

## Warning in lognet(xd, is.sparse, ix, jx, y, weights, offset, alpha, nobs, : one
## multinomial or binomial class has fewer than 8 observations; dangerous ground

## Warning in lognet(xd, is.sparse, ix, jx, y, weights, offset, alpha, nobs, : one
## multinomial or binomial class has fewer than 8 observations; dangerous ground

## Warning in lognet(xd, is.sparse, ix, jx, y, weights, offset, alpha, nobs, : one
## multinomial or binomial class has fewer than 8 observations; dangerous ground

## Warning in lognet(xd, is.sparse, ix, jx, y, weights, offset, alpha, nobs, : one
## multinomial or binomial class has fewer than 8 observations; dangerous ground

## Warning in lognet(xd, is.sparse, ix, jx, y, weights, offset, alpha, nobs, : one
## multinomial or binomial class has fewer than 8 observations; dangerous ground

## Warning in lognet(xd, is.sparse, ix, jx, y, weights, offset, alpha, nobs, : one
## multinomial or binomial class has fewer than 8 observations; dangerous ground

## Warning in lognet(xd, is.sparse, ix, jx, y, weights, offset, alpha, nobs, : one
## multinomial or binomial class has fewer than 8 observations; dangerous ground

## Warning in lognet(xd, is.sparse, ix, jx, y, weights, offset, alpha, nobs, : one
## multinomial or binomial class has fewer than 8 observations; dangerous ground

## Warning in lognet(xd, is.sparse, ix, jx, y, weights, offset, alpha, nobs, : one
## multinomial or binomial class has fewer than 8 observations; dangerous ground
```

```
# define the number of elements in class 1
threshold <- seq(0, 1, length.out = 1000)
accuracy <- c()
for (t in threshold){
  tot <- 0
  for (i in 1:(length(preds))){
    if ((groups[i] == "Allentoft" && preds[i] < (1 - t)) || (groups[i] == "Damgaard" && preds[i] > (t))){
      tot <- tot + 1
    }
  }
  prop <- tot/length(preds)
  accuracy <- c(accuracy, prop)
}  
BP11_extraction_protocol <- accuracy
```

Sample type variable:

```
groups<- as.factor(c("Tooth", "Tooth", "Tooth", "Bone", "Tooth", "Tooth", "Tooth", "Tooth", "Tooth", "Tooth", "Tooth", "Tooth", "Tooth", "Tooth", "Tooth", "Tooth", "Tooth", "Tooth", "Tooth", "Tooth", "Tooth", "Tooth", "Tooth", "Tooth", "Tooth", "Tooth", "Tooth", "Bone", "Bone", "Bone", "Bone", "Bone", "Bone", "Bone", "Bone", "Bone", "Bone", "Bone"))

n <- nrow(BP11_train)
# placeholder for storing the i-th prediction
preds <- rep(NA, n)
for(i in 1:n) {
    dataf.BP11_train <- BP11_train[-i, ]
    dataf.test <- BP11_train[i, ]
    groups_tmp <- groups[-i]
    set.seed(123) 
    cv.lasso <- cv.glmnet(as.matrix(dataf.BP11_train), groups_tmp, alpha = a, family = "binomial")
    glmnet.fit <- glmnet(as.matrix(dataf.BP11_train), groups_tmp, alpha = a, family = "binomial", lambda = cv.lasso$lambda.min)
    preds[i] <- predict(glmnet.fit, as.matrix(dataf.test), type="response")
}

# define the number of elements in class 1
threshold <- seq(0, 1, length.out = 1000)
accuracy <- c()
for (t in threshold){
  tot <- 0
  for (i in 1:(length(preds))){
    if ((groups[i] == "Bone" && preds[i] < (1 - t)) || (groups[i] == "Tooth" && preds[i] > (t))){
      tot <- tot + 1
    }
  }
  prop <- tot/length(preds)
  accuracy <- c(accuracy, prop)
}  
BP11_sample_type <- accuracy
```

# 15bp

Read BP data into R:

```
BP15_mash <- read.csv("kmer_size_comparison/BP_15bp.csv")
# Add row names
rownames(BP15_mash) <- names(BP15_mash)
# Transform to matrix object for plotting
BP15_mash_mat <- as.matrix(BP15_mash)
```

BP dataset MDS

```
library(ggfortify)
BP15_pca_mat <- prcomp(BP15_mash_mat, scale. = TRUE)
```

Plot: (color according to geography) green = Brazil blue = Polynesia (RapaNui)

```
BP15_col_vec <- c("#33a02c", "#33a02c", "#33a02c", "#33a02c", "#33a02c", "#33a02c", "#33a02c", "#33a02c", "#33a02c", "#33a02c", "#33a02c", "#33a02c", "#33a02c", "#33a02c", "#33a02c", "#33a02c", "#33a02c", "#33a02c", "#33a02c", "#33a02c", "#33a02c", "#33a02c", "#33a02c", "#1f78b4", "#1f78b4", "#1f78b4", "#1f78b4", "#1f78b4", "#1f78b4", "#1f78b4", "#1f78b4", "#1f78b4", "#1f78b4", "#1f78b4", "#1f78b4", "#1f78b4", "#1f78b4", "#1f78b4")

autoplot(BP15_pca_mat, x = 1, y = 2, pch = 16, label = F, size = 4, label.size = 3, alpha = 0.7, label.colour = NULL, label.hjust=0.0001, label.vjust=-0.5,
          colour = BP15_col_vec) +
     theme_bw()
```

```
autoplot(BP15_pca_mat, x = 1, y = 3, pch = 16, label = F, size = 4, label.size = 3, alpha = 0.7, label.colour = NULL, label.hjust=0.0001, label.vjust=-0.5,
          colour = BP15_col_vec) +
     theme_bw()
```

# Model training and accuracy evalutation B-P dataset:

Select type of regularization (0 = ridge, 1 = lasso, 0<a<1 = elastic net):

```
library(glmnet)
a = 1
```

Get training dataset:

```
# get the PCs dataframe
BP15_train <- as.data.frame(BP15_pca_mat$x)
# remove the last PC which explain 0% of the variance
BP15_train <- BP15_train[,1:37]
```

Geography variable:

```
groups<- as.factor(c("Brazil", "Brazil", "Brazil", "Brazil", "Brazil", "Brazil", "Brazil", "Brazil", "Brazil", "Brazil", "Brazil", "Brazil", "Brazil", "Brazil", "Brazil", "Brazil", "Brazil", "Brazil", "Brazil", "Brazil", "Brazil", "Brazil", "Brazil", "RapaNui", "RapaNui", "RapaNui", "RapaNui", "RapaNui", "RapaNui", "RapaNui", "RapaNui", "RapaNui", "RapaNui", "RapaNui", "RapaNui", "RapaNui", "RapaNui", "RapaNui"))

n <- nrow(BP15_train)
# placeholder for storing the i-th prediction
preds <- rep(NA, n)
for(i in 1:n) {
    dataf.BP15_train <- BP15_train[-i, ]
    dataf.test <- BP15_train[i, ]
    groups_tmp <- groups[-i]
    set.seed(123) 
    cv.lasso <- cv.glmnet(as.matrix(dataf.BP15_train), groups_tmp, alpha = a, family = "binomial")
    glmnet.fit <- glmnet(as.matrix(dataf.BP15_train), groups_tmp, alpha = a, family = "binomial", lambda = cv.lasso$lambda.min)
    preds[i] <- predict(glmnet.fit, as.matrix(dataf.test), type="response")
}

# define the number of elements in class 1
threshold <- seq(0, 1, length.out = 1000)
accuracy <- c()
for (t in threshold){
  tot <- 0
  for (i in 1:(length(preds))){
    if ((groups[i] == "Brazil" && preds[i] < (1 - t)) || (groups[i] == "RapaNui" && preds[i] > (t))){
      tot <- tot + 1
    }
  }
  prop <- tot/length(preds)
  accuracy <- c(accuracy, prop)
}  
BP15_geo <- accuracy
```

Extraction protocol variable:

```
groups<- as.factor(c("Allentoft", "Allentoft", "Allentoft", "Allentoft", "Allentoft", "Allentoft", "Allentoft", "Allentoft", "Allentoft", "Allentoft", "Allentoft", "Allentoft", "Allentoft", "Allentoft", "Allentoft", "Allentoft", "Allentoft", "Allentoft", "Allentoft", "Allentoft", "Allentoft", "Allentoft", "Allentoft", "Allentoft", "Allentoft", "Allentoft", "Allentoft", "Damgaard", "Damgaard", "Damgaard", "Damgaard", "Damgaard", "Damgaard", "Damgaard", "Damgaard", "Damgaard", "Damgaard", "Damgaard"))

n <- nrow(BP15_train)
# placeholder for storing the i-th prediction
preds <- rep(NA, n)
for(i in 1:n) {
    dataf.BP15_train <- BP15_train[-i, ]
    dataf.test <- BP15_train[i, ]
    groups_tmp <- groups[-i]
    set.seed(123) 
    cv.lasso <- cv.glmnet(as.matrix(dataf.BP15_train), groups_tmp, alpha = a, family = "binomial")
    glmnet.fit <- glmnet(as.matrix(dataf.BP15_train), groups_tmp, alpha = a, family = "binomial", lambda = cv.lasso$lambda.min)
    preds[i] <- predict(glmnet.fit, as.matrix(dataf.test), type="response")
}
```

```
## Warning in lognet(xd, is.sparse, ix, jx, y, weights, offset, alpha, nobs, : one
## multinomial or binomial class has fewer than 8 observations; dangerous ground

## Warning in lognet(xd, is.sparse, ix, jx, y, weights, offset, alpha, nobs, : one
## multinomial or binomial class has fewer than 8 observations; dangerous ground

## Warning in lognet(xd, is.sparse, ix, jx, y, weights, offset, alpha, nobs, : one
## multinomial or binomial class has fewer than 8 observations; dangerous ground

## Warning in lognet(xd, is.sparse, ix, jx, y, weights, offset, alpha, nobs, : one
## multinomial or binomial class has fewer than 8 observations; dangerous ground

## Warning in lognet(xd, is.sparse, ix, jx, y, weights, offset, alpha, nobs, : one
## multinomial or binomial class has fewer than 8 observations; dangerous ground

## Warning in lognet(xd, is.sparse, ix, jx, y, weights, offset, alpha, nobs, : one
## multinomial or binomial class has fewer than 8 observations; dangerous ground

## Warning in lognet(xd, is.sparse, ix, jx, y, weights, offset, alpha, nobs, : one
## multinomial or binomial class has fewer than 8 observations; dangerous ground

## Warning in lognet(xd, is.sparse, ix, jx, y, weights, offset, alpha, nobs, : one
## multinomial or binomial class has fewer than 8 observations; dangerous ground

## Warning in lognet(xd, is.sparse, ix, jx, y, weights, offset, alpha, nobs, : one
## multinomial or binomial class has fewer than 8 observations; dangerous ground

## Warning in lognet(xd, is.sparse, ix, jx, y, weights, offset, alpha, nobs, : one
## multinomial or binomial class has fewer than 8 observations; dangerous ground

## Warning in lognet(xd, is.sparse, ix, jx, y, weights, offset, alpha, nobs, : one
## multinomial or binomial class has fewer than 8 observations; dangerous ground
```

```
# define the number of elements in class 1
threshold <- seq(0, 1, length.out = 1000)
accuracy <- c()
for (t in threshold){
  tot <- 0
  for (i in 1:(length(preds))){
    if ((groups[i] == "Allentoft" && preds[i] < (1 - t)) || (groups[i] == "Damgaard" && preds[i] > (t))){
      tot <- tot + 1
    }
  }
  prop <- tot/length(preds)
  accuracy <- c(accuracy, prop)
}  
BP15_extraction_protocol <- accuracy
```

Sample type variable:

```
groups<- as.factor(c("Tooth", "Tooth", "Tooth", "Bone", "Tooth", "Tooth", "Tooth", "Tooth", "Tooth", "Tooth", "Tooth", "Tooth", "Tooth", "Tooth", "Tooth", "Tooth", "Tooth", "Tooth", "Tooth", "Tooth", "Tooth", "Tooth", "Tooth", "Tooth", "Tooth", "Tooth", "Tooth", "Bone", "Bone", "Bone", "Bone", "Bone", "Bone", "Bone", "Bone", "Bone", "Bone", "Bone"))

n <- nrow(BP15_train)
# placeholder for storing the i-th prediction
preds <- rep(NA, n)
for(i in 1:n) {
    dataf.BP15_train <- BP15_train[-i, ]
    dataf.test <- BP15_train[i, ]
    groups_tmp <- groups[-i]
    set.seed(123) 
    cv.lasso <- cv.glmnet(as.matrix(dataf.BP15_train), groups_tmp, alpha = a, family = "binomial")
    glmnet.fit <- glmnet(as.matrix(dataf.BP15_train), groups_tmp, alpha = a, family = "binomial", lambda = cv.lasso$lambda.min)
    preds[i] <- predict(glmnet.fit, as.matrix(dataf.test), type="response")
}

# define the number of elements in class 1
threshold <- seq(0, 1, length.out = 1000)
accuracy <- c()
for (t in threshold){
  tot <- 0
  for (i in 1:(length(preds))){
    if ((groups[i] == "Bone" && preds[i] < (1 - t)) || (groups[i] == "Tooth" && preds[i] > (t))){
      tot <- tot + 1
    }
  }
  prop <- tot/length(preds)
  accuracy <- c(accuracy, prop)
}  
BP15_sample_type <- accuracy
```

# 25bp

Read BP data into R:

```
BP25_mash <- read.csv("kmer_size_comparison/BP_25bp.csv")
# Add row names
rownames(BP25_mash) <- names(BP25_mash)
# Transform to matrix object for plotting
BP25_mash_mat <- as.matrix(BP25_mash)
```

BP dataset MDS

```
library(ggfortify)
BP25_pca_mat <- prcomp(BP25_mash_mat, scale. = TRUE)
```

Plot: (color according to geography) green = Brazil blue = Polynesia (RapaNui)

```
BP25_col_vec <- c("#33a02c", "#33a02c", "#33a02c", "#33a02c", "#33a02c", "#33a02c", "#33a02c", "#33a02c", "#33a02c", "#33a02c", "#33a02c", "#33a02c", "#33a02c", "#33a02c", "#33a02c", "#33a02c", "#33a02c", "#33a02c", "#33a02c", "#33a02c", "#33a02c", "#33a02c", "#33a02c", "#1f78b4", "#1f78b4", "#1f78b4", "#1f78b4", "#1f78b4", "#1f78b4", "#1f78b4", "#1f78b4", "#1f78b4", "#1f78b4", "#1f78b4", "#1f78b4", "#1f78b4", "#1f78b4", "#1f78b4")

autoplot(BP25_pca_mat, x = 1, y = 2, pch = 16, label = F, size = 4, label.size = 3, alpha = 0.7, label.colour = NULL, label.hjust=0.0001, label.vjust=-0.5,
          colour = BP25_col_vec) +
     theme_bw()
```

```
autoplot(BP25_pca_mat, x = 1, y = 3, pch = 16, label = F, size = 4, label.size = 3, alpha = 0.7, label.colour = NULL, label.hjust=0.0001, label.vjust=-0.5,
          colour = BP25_col_vec) +
     theme_bw()
```

# Model training and accuracy evalutation B-P dataset:

Select type of regularization (0 = ridge, 1 = lasso, 0<a<1 = elastic net):

```
library(glmnet)
a = 1
```

Get training dataset:

```
# get the PCs dataframe
BP25_train <- as.data.frame(BP25_pca_mat$x)
# remove the last PC which explain 0% of the variance
BP25_train <- BP25_train[,1:37]
```

Geography variable:

```
groups<- as.factor(c("Brazil", "Brazil", "Brazil", "Brazil", "Brazil", "Brazil", "Brazil", "Brazil", "Brazil", "Brazil", "Brazil", "Brazil", "Brazil", "Brazil", "Brazil", "Brazil", "Brazil", "Brazil", "Brazil", "Brazil", "Brazil", "Brazil", "Brazil", "RapaNui", "RapaNui", "RapaNui", "RapaNui", "RapaNui", "RapaNui", "RapaNui", "RapaNui", "RapaNui", "RapaNui", "RapaNui", "RapaNui", "RapaNui", "RapaNui", "RapaNui"))

n <- nrow(BP25_train)
# placeholder for storing the i-th prediction
preds <- rep(NA, n)
for(i in 1:n) {
    dataf.BP25_train <- BP25_train[-i, ]
    dataf.test <- BP25_train[i, ]
    groups_tmp <- groups[-i]
    set.seed(123) 
    cv.lasso <- cv.glmnet(as.matrix(dataf.BP25_train), groups_tmp, alpha = a, family = "binomial")
    glmnet.fit <- glmnet(as.matrix(dataf.BP25_train), groups_tmp, alpha = a, family = "binomial", lambda = cv.lasso$lambda.min)
    preds[i] <- predict(glmnet.fit, as.matrix(dataf.test), type="response")
}

# define the number of elements in class 1
threshold <- seq(0, 1, length.out = 1000)
accuracy <- c()
for (t in threshold){
  tot <- 0
  for (i in 1:(length(preds))){
    if ((groups[i] == "Brazil" && preds[i] < (1 - t)) || (groups[i] == "RapaNui" && preds[i] > (t))){
      tot <- tot + 1
    }
  }
  prop <- tot/length(preds)
  accuracy <- c(accuracy, prop)
}  
BP25_geo <- accuracy
```

Extraction protocol variable:

```
groups<- as.factor(c("Allentoft", "Allentoft", "Allentoft", "Allentoft", "Allentoft", "Allentoft", "Allentoft", "Allentoft", "Allentoft", "Allentoft", "Allentoft", "Allentoft", "Allentoft", "Allentoft", "Allentoft", "Allentoft", "Allentoft", "Allentoft", "Allentoft", "Allentoft", "Allentoft", "Allentoft", "Allentoft", "Allentoft", "Allentoft", "Allentoft", "Allentoft", "Damgaard", "Damgaard", "Damgaard", "Damgaard", "Damgaard", "Damgaard", "Damgaard", "Damgaard", "Damgaard", "Damgaard", "Damgaard"))

n <- nrow(BP25_train)
# placeholder for storing the i-th prediction
preds <- rep(NA, n)
for(i in 1:n) {
    dataf.BP25_train <- BP25_train[-i, ]
    dataf.test <- BP25_train[i, ]
    groups_tmp <- groups[-i]
    set.seed(123) 
    cv.lasso <- cv.glmnet(as.matrix(dataf.BP25_train), groups_tmp, alpha = a, family = "binomial")
    glmnet.fit <- glmnet(as.matrix(dataf.BP25_train), groups_tmp, alpha = a, family = "binomial", lambda = cv.lasso$lambda.min)
    preds[i] <- predict(glmnet.fit, as.matrix(dataf.test), type="response")
}
```

```
## Warning in lognet(xd, is.sparse, ix, jx, y, weights, offset, alpha, nobs, : one
## multinomial or binomial class has fewer than 8 observations; dangerous ground

## Warning in lognet(xd, is.sparse, ix, jx, y, weights, offset, alpha, nobs, : one
## multinomial or binomial class has fewer than 8 observations; dangerous ground

## Warning in lognet(xd, is.sparse, ix, jx, y, weights, offset, alpha, nobs, : one
## multinomial or binomial class has fewer than 8 observations; dangerous ground

## Warning in lognet(xd, is.sparse, ix, jx, y, weights, offset, alpha, nobs, : one
## multinomial or binomial class has fewer than 8 observations; dangerous ground

## Warning in lognet(xd, is.sparse, ix, jx, y, weights, offset, alpha, nobs, : one
## multinomial or binomial class has fewer than 8 observations; dangerous ground

## Warning in lognet(xd, is.sparse, ix, jx, y, weights, offset, alpha, nobs, : one
## multinomial or binomial class has fewer than 8 observations; dangerous ground

## Warning in lognet(xd, is.sparse, ix, jx, y, weights, offset, alpha, nobs, : one
## multinomial or binomial class has fewer than 8 observations; dangerous ground

## Warning in lognet(xd, is.sparse, ix, jx, y, weights, offset, alpha, nobs, : one
## multinomial or binomial class has fewer than 8 observations; dangerous ground

## Warning in lognet(xd, is.sparse, ix, jx, y, weights, offset, alpha, nobs, : one
## multinomial or binomial class has fewer than 8 observations; dangerous ground

## Warning in lognet(xd, is.sparse, ix, jx, y, weights, offset, alpha, nobs, : one
## multinomial or binomial class has fewer than 8 observations; dangerous ground

## Warning in lognet(xd, is.sparse, ix, jx, y, weights, offset, alpha, nobs, : one
## multinomial or binomial class has fewer than 8 observations; dangerous ground
```

```
# define the number of elements in class 1
threshold <- seq(0, 1, length.out = 1000)
accuracy <- c()
for (t in threshold){
  tot <- 0
  for (i in 1:(length(preds))){
    if ((groups[i] == "Allentoft" && preds[i] < (1 - t)) || (groups[i] == "Damgaard" && preds[i] > (t))){
      tot <- tot + 1
    }
  }
  prop <- tot/length(preds)
  accuracy <- c(accuracy, prop)
}  
BP25_extraction_protocol <- accuracy
```

Sample type variable:

```
groups<- as.factor(c("Tooth", "Tooth", "Tooth", "Bone", "Tooth", "Tooth", "Tooth", "Tooth", "Tooth", "Tooth", "Tooth", "Tooth", "Tooth", "Tooth", "Tooth", "Tooth", "Tooth", "Tooth", "Tooth", "Tooth", "Tooth", "Tooth", "Tooth", "Tooth", "Tooth", "Tooth", "Tooth", "Bone", "Bone", "Bone", "Bone", "Bone", "Bone", "Bone", "Bone", "Bone", "Bone", "Bone"))

n <- nrow(BP25_train)
# placeholder for storing the i-th prediction
preds <- rep(NA, n)
for(i in 1:n) {
    dataf.BP25_train <- BP25_train[-i, ]
    dataf.test <- BP25_train[i, ]
    groups_tmp <- groups[-i]
    set.seed(123) 
    cv.lasso <- cv.glmnet(as.matrix(dataf.BP25_train), groups_tmp, alpha = a, family = "binomial")
    glmnet.fit <- glmnet(as.matrix(dataf.BP25_train), groups_tmp, alpha = a, family = "binomial", lambda = cv.lasso$lambda.min)
    preds[i] <- predict(glmnet.fit, as.matrix(dataf.test), type="response")
}

# define the number of elements in class 1
threshold <- seq(0, 1, length.out = 1000)
accuracy <- c()
for (t in threshold){
  tot <- 0
  for (i in 1:(length(preds))){
    if ((groups[i] == "Bone" && preds[i] < (1 - t)) || (groups[i] == "Tooth" && preds[i] > (t))){
      tot <- tot + 1
    }
  }
  prop <- tot/length(preds)
  accuracy <- c(accuracy, prop)
}  
BP25_sample_type <- accuracy
```

# 31bp

Read BP data into R:

```
BP31_mash <- read.csv("kmer_size_comparison/BP_31bp.csv")
# Add row names
rownames(BP31_mash) <- names(BP31_mash)
# Transform to matrix object for plotting
BP31_mash_mat <- as.matrix(BP31_mash)
```

BP dataset MDS

```
library(ggfortify)
BP31_pca_mat <- prcomp(BP31_mash_mat, scale. = TRUE)
```

Plot: (color according to geography) green = Brazil blue = Polynesia (RapaNui)

```
BP31_col_vec <- c("#33a02c", "#33a02c", "#33a02c", "#33a02c", "#33a02c", "#33a02c", "#33a02c", "#33a02c", "#33a02c", "#33a02c", "#33a02c", "#33a02c", "#33a02c", "#33a02c", "#33a02c", "#33a02c", "#33a02c", "#33a02c", "#33a02c", "#33a02c", "#33a02c", "#33a02c", "#33a02c", "#1f78b4", "#1f78b4", "#1f78b4", "#1f78b4", "#1f78b4", "#1f78b4", "#1f78b4", "#1f78b4", "#1f78b4", "#1f78b4", "#1f78b4", "#1f78b4", "#1f78b4", "#1f78b4", "#1f78b4")

autoplot(BP31_pca_mat, x = 1, y = 2, pch = 16, label = F, size = 4, label.size = 3, alpha = 0.7, label.colour = NULL, label.hjust=0.0001, label.vjust=-0.5,
          colour = BP31_col_vec) +
     theme_bw()
```

```
autoplot(BP31_pca_mat, x = 1, y = 3, pch = 16, label = F, size = 4, label.size = 3, alpha = 0.7, label.colour = NULL, label.hjust=0.0001, label.vjust=-0.5,
          colour = BP31_col_vec) +
     theme_bw()
```

# Model training and accuracy evalutation B-P dataset:

Select type of regularization (0 = ridge, 1 = lasso, 0<a<1 = elastic net):

```
library(glmnet)
a = 1
```

Get training dataset:

```
# get the PCs dataframe
BP31_train <- as.data.frame(BP31_pca_mat$x)
# remove the last PC which explain 0% of the variance
BP31_train <- BP31_train[,1:37]
```

Geography variable:

```
groups<- as.factor(c("Brazil", "Brazil", "Brazil", "Brazil", "Brazil", "Brazil", "Brazil", "Brazil", "Brazil", "Brazil", "Brazil", "Brazil", "Brazil", "Brazil", "Brazil", "Brazil", "Brazil", "Brazil", "Brazil", "Brazil", "Brazil", "Brazil", "Brazil", "RapaNui", "RapaNui", "RapaNui", "RapaNui", "RapaNui", "RapaNui", "RapaNui", "RapaNui", "RapaNui", "RapaNui", "RapaNui", "RapaNui", "RapaNui", "RapaNui", "RapaNui"))

n <- nrow(BP31_train)
# placeholder for storing the i-th prediction
preds <- rep(NA, n)
for(i in 1:n) {
    dataf.BP31_train <- BP31_train[-i, ]
    dataf.test <- BP31_train[i, ]
    groups_tmp <- groups[-i]
    set.seed(123) 
    cv.lasso <- cv.glmnet(as.matrix(dataf.BP31_train), groups_tmp, alpha = a, family = "binomial")
    glmnet.fit <- glmnet(as.matrix(dataf.BP31_train), groups_tmp, alpha = a, family = "binomial", lambda = cv.lasso$lambda.min)
    preds[i] <- predict(glmnet.fit, as.matrix(dataf.test), type="response")
}

# define the number of elements in class 1
threshold <- seq(0, 1, length.out = 1000)
accuracy <- c()
for (t in threshold){
  tot <- 0
  for (i in 1:(length(preds))){
    if ((groups[i] == "Brazil" && preds[i] < (1 - t)) || (groups[i] == "RapaNui" && preds[i] > (t))){
      tot <- tot + 1
    }
  }
  prop <- tot/length(preds)
  accuracy <- c(accuracy, prop)
}  
BP31_geo <- accuracy
```

Extraction protocol variable:

```
groups<- as.factor(c("Allentoft", "Allentoft", "Allentoft", "Allentoft", "Allentoft", "Allentoft", "Allentoft", "Allentoft", "Allentoft", "Allentoft", "Allentoft", "Allentoft", "Allentoft", "Allentoft", "Allentoft", "Allentoft", "Allentoft", "Allentoft", "Allentoft", "Allentoft", "Allentoft", "Allentoft", "Allentoft", "Allentoft", "Allentoft", "Allentoft", "Allentoft", "Damgaard", "Damgaard", "Damgaard", "Damgaard", "Damgaard", "Damgaard", "Damgaard", "Damgaard", "Damgaard", "Damgaard", "Damgaard"))

n <- nrow(BP31_train)
# placeholder for storing the i-th prediction
preds <- rep(NA, n)
for(i in 1:n) {
    dataf.BP31_train <- BP31_train[-i, ]
    dataf.test <- BP31_train[i, ]
    groups_tmp <- groups[-i]
    set.seed(123) 
    cv.lasso <- cv.glmnet(as.matrix(dataf.BP31_train), groups_tmp, alpha = a, family = "binomial")
    glmnet.fit <- glmnet(as.matrix(dataf.BP31_train), groups_tmp, alpha = a, family = "binomial", lambda = cv.lasso$lambda.min)
    preds[i] <- predict(glmnet.fit, as.matrix(dataf.test), type="response")
}
```

```
## Warning in lognet(xd, is.sparse, ix, jx, y, weights, offset, alpha, nobs, : one
## multinomial or binomial class has fewer than 8 observations; dangerous ground

## Warning in lognet(xd, is.sparse, ix, jx, y, weights, offset, alpha, nobs, : one
## multinomial or binomial class has fewer than 8 observations; dangerous ground

## Warning in lognet(xd, is.sparse, ix, jx, y, weights, offset, alpha, nobs, : one
## multinomial or binomial class has fewer than 8 observations; dangerous ground

## Warning in lognet(xd, is.sparse, ix, jx, y, weights, offset, alpha, nobs, : one
## multinomial or binomial class has fewer than 8 observations; dangerous ground

## Warning in lognet(xd, is.sparse, ix, jx, y, weights, offset, alpha, nobs, : one
## multinomial or binomial class has fewer than 8 observations; dangerous ground

## Warning in lognet(xd, is.sparse, ix, jx, y, weights, offset, alpha, nobs, : one
## multinomial or binomial class has fewer than 8 observations; dangerous ground

## Warning in lognet(xd, is.sparse, ix, jx, y, weights, offset, alpha, nobs, : one
## multinomial or binomial class has fewer than 8 observations; dangerous ground

## Warning in lognet(xd, is.sparse, ix, jx, y, weights, offset, alpha, nobs, : one
## multinomial or binomial class has fewer than 8 observations; dangerous ground

## Warning in lognet(xd, is.sparse, ix, jx, y, weights, offset, alpha, nobs, : one
## multinomial or binomial class has fewer than 8 observations; dangerous ground

## Warning in lognet(xd, is.sparse, ix, jx, y, weights, offset, alpha, nobs, : one
## multinomial or binomial class has fewer than 8 observations; dangerous ground

## Warning in lognet(xd, is.sparse, ix, jx, y, weights, offset, alpha, nobs, : one
## multinomial or binomial class has fewer than 8 observations; dangerous ground
```

```
# define the number of elements in class 1
threshold <- seq(0, 1, length.out = 1000)
accuracy <- c()
for (t in threshold){
  tot <- 0
  for (i in 1:(length(preds))){
    if ((groups[i] == "Allentoft" && preds[i] < (1 - t)) || (groups[i] == "Damgaard" && preds[i] > (t))){
      tot <- tot + 1
    }
  }
  prop <- tot/length(preds)
  accuracy <- c(accuracy, prop)
}  
BP31_extraction_protocol <- accuracy
```

Sample type variable:

```
groups<- as.factor(c("Tooth", "Tooth", "Tooth", "Bone", "Tooth", "Tooth", "Tooth", "Tooth", "Tooth", "Tooth", "Tooth", "Tooth", "Tooth", "Tooth", "Tooth", "Tooth", "Tooth", "Tooth", "Tooth", "Tooth", "Tooth", "Tooth", "Tooth", "Tooth", "Tooth", "Tooth", "Tooth", "Bone", "Bone", "Bone", "Bone", "Bone", "Bone", "Bone", "Bone", "Bone", "Bone", "Bone"))

n <- nrow(BP31_train)
# placeholder for storing the i-th prediction
preds <- rep(NA, n)
for(i in 1:n) {
    dataf.BP31_train <- BP31_train[-i, ]
    dataf.test <- BP31_train[i, ]
    groups_tmp <- groups[-i]
    set.seed(123) 
    cv.lasso <- cv.glmnet(as.matrix(dataf.BP31_train), groups_tmp, alpha = a, family = "binomial")
    glmnet.fit <- glmnet(as.matrix(dataf.BP31_train), groups_tmp, alpha = a, family = "binomial", lambda = cv.lasso$lambda.min)
    preds[i] <- predict(glmnet.fit, as.matrix(dataf.test), type="response")
}

# define the number of elements in class 1
threshold <- seq(0, 1, length.out = 1000)
accuracy <- c()
for (t in threshold){
  tot <- 0
  for (i in 1:(length(preds))){
    if ((groups[i] == "Bone" && preds[i] < (1 - t)) || (groups[i] == "Tooth" && preds[i] > (t))){
      tot <- tot + 1
    }
  }
  prop <- tot/length(preds)
  accuracy <- c(accuracy, prop)
}  
BP31_sample_type <- accuracy
```

Plot accuracy (B-P dataset - geography):

```
ggplot() + 
  geom_line(aes(threshold, BP11_geo), color = "yellow1") +
  geom_line(aes(threshold, BP15_geo), color = "gold1") +
  geom_line(aes(threshold, BP_geo), color = "orange2") +
  geom_line(aes(threshold, BP25_geo), color = "red2") +
  geom_line(aes(threshold, BP31_geo), color = "red4") +
  theme_bw() +
  ylim(0, 1)
```

Plot accuracy (B-P dataset - extraction protocol):

```
ggplot() + 
  geom_line(aes(threshold, BP11_extraction_protocol), color = "yellow1") +
  geom_line(aes(threshold, BP15_extraction_protocol), color = "gold1") +
  geom_line(aes(threshold, BP_extraction_protocol), color = "orange2") +
  geom_line(aes(threshold, BP25_extraction_protocol), color = "red2") +
  geom_line(aes(threshold, BP31_extraction_protocol), color = "red4") +
  theme_bw() +
  ylim(0, 1)
```

Plot accuracy (B-P dataset - sample type):

```
ggplot() + 
  geom_line(aes(threshold, BP11_sample_type), color = "yellow1") +
  geom_line(aes(threshold, BP15_sample_type), color = "gold1") +
  geom_line(aes(threshold, BP_sample_type), color = "orange2") +
  geom_line(aes(threshold, BP25_sample_type), color = "red2") +
  geom_line(aes(threshold, BP31_sample_type), color = "red4") +
  theme_bw() +
  ylim(0, 1)
```

# DE dataset

# 21 bp

Read data into R: (all the samples)

```
# import data
DE_all_mash <- read.csv("kmers_similarity_matrices/DE_all_similarity_matrix.csv") 
# Add row names
rownames(DE_all_mash) <- names(DE_all_mash)
# Transform to matrix object for plotting
DE_all_mash_mat <- as.matrix(DE_all_mash)
```

DE dataset Plot MDS (color according to geography) - also low-depth samples orange = Denmark purple = England

```
library(ggfortify)
DE_all_pca_mat <- prcomp(DE_all_mash_mat, scale. = TRUE)
DE_all_col_vec <- c("#d95f02", "#d95f02", "#d95f02", "#d95f02", "#d95f02", "#d95f02", "#d95f02", "#d95f02", "#d95f02", "#d95f02", "#d95f02", "#d95f02", "#d95f02", "#d95f02", "#d95f02", "#d95f02", "#d95f02", "#d95f02", "#d95f02", "#d95f02", "#d95f02", "#d95f02", "#d95f02", "#d95f02", "#d95f02", "#d95f02", "#d95f02", "#d95f02", "#d95f02", "#7570b3", "#7570b3", "#7570b3", "#7570b3", "#7570b3", "#7570b3", "#7570b3", "#7570b3", "#7570b3", "#7570b3", "#7570b3", "#7570b3", "#7570b3", "#7570b3", "#7570b3", "#7570b3", "#7570b3", "#7570b3", "#7570b3", "#7570b3", "#7570b3", "#7570b3")

autoplot(DE_all_pca_mat, x = 1, y = 3, pch = 16, label.size = 2, size = 6, alpha = 0.7, label = FALSE, label.colour = NULL, label.hjust=0.0001, label.vjust=-0.5,
        colour = DE_all_col_vec) +
        theme_bw()
```

D-E dataset (all samples) geography variable:

```
DE_all_train <- as.data.frame(DE_all_pca_mat$x)

# geo:
groups <- factor(c("Denmark", "Denmark", "Denmark", "Denmark", "Denmark", "Denmark", "Denmark", "Denmark", "Denmark", "Denmark", "Denmark", "Denmark", "Denmark", "Denmark", "Denmark", "Denmark", "Denmark", "Denmark", "Denmark", "Denmark", "Denmark", "Denmark", "Denmark", "Denmark", "Denmark", "Denmark", "Denmark", "Denmark", "Denmark", "England", "England",  "England", "England", "England", "England",  "England", "England", "England", "England",  "England", "England", "England", "England",  "England", "England", "England", "England",  "England", "England", "England", "England"))

n <- nrow(DE_all_train)
# placeholder for storing the i-th prediction
preds <- rep(NA, n)
for(i in 1:n) {
    dataf.train <- DE_all_train[-i, ]
    dataf.test <- DE_all_train[i, ]
    groups_tmp <- groups[-i]
    set.seed(123) 
    cv.lasso <- cv.glmnet(as.matrix(dataf.train), groups_tmp, alpha = a, family = "binomial")
    glmnet.fit <- glmnet(as.matrix(dataf.train), groups_tmp, alpha = a, family = "binomial", lambda = cv.lasso$lambda.min)
    preds[i] <- predict(glmnet.fit, as.matrix(dataf.test), type="response")
}

# define the number of elements in class 1
threshold <- seq(0, 1, length.out = 1000)
accuracy <- c()
for (t in threshold){
  tot <- 0
  for (i in 1:(length(preds))){
    if ((groups[i] == "Denmark" && preds[i] < (1 - t)) || (groups[i] == "England" && preds[i] > (t))){
      tot <- tot + 1
    }
  }
  prop <- tot/length(preds)
  accuracy <- c(accuracy, prop)
}  
DE_all_geo <- accuracy
```

# DE 11bp

Read data into R: (all the samples)

```
# import data
DE11_all_mash <- read.csv("kmer_size_comparison/DE_11bp.csv") 
# Add row names
rownames(DE11_all_mash) <- names(DE11_all_mash)
# Transform to matrix object for plotting
DE11_all_mash_mat <- as.matrix(DE11_all_mash)
```

DE dataset Plot MDS (color according to geography) - also low-depth samples orange = Denmark purple = England

```
library(ggfortify)
DE11_all_pca_mat <- prcomp(DE11_all_mash_mat, scale. = TRUE)
DE11_all_col_vec <- c("#d95f02", "#d95f02", "#d95f02", "#d95f02", "#d95f02", "#d95f02", "#d95f02", "#d95f02", "#d95f02", "#d95f02", "#d95f02", "#d95f02", "#d95f02", "#d95f02", "#d95f02", "#d95f02", "#d95f02", "#d95f02", "#d95f02", "#d95f02", "#d95f02", "#d95f02", "#d95f02", "#d95f02", "#d95f02", "#d95f02", "#d95f02", "#d95f02", "#d95f02", "#7570b3", "#7570b3", "#7570b3", "#7570b3", "#7570b3", "#7570b3", "#7570b3", "#7570b3", "#7570b3", "#7570b3", "#7570b3", "#7570b3", "#7570b3", "#7570b3", "#7570b3", "#7570b3", "#7570b3", "#7570b3", "#7570b3", "#7570b3", "#7570b3", "#7570b3")

autoplot(DE11_all_pca_mat, x = 1, y = 3, pch = 16, label.size = 2, size = 6, alpha = 0.7, label = FALSE, label.colour = NULL, label.hjust=0.0001, label.vjust=-0.5,
        colour = DE11_all_col_vec) +
        theme_bw()
```

```
DE11_all_train <- as.data.frame(DE11_all_pca_mat$x)

# geo:
groups <- factor(c("Denmark", "Denmark", "Denmark", "Denmark", "Denmark", "Denmark", "Denmark", "Denmark", "Denmark", "Denmark", "Denmark", "Denmark", "Denmark", "Denmark", "Denmark", "Denmark", "Denmark", "Denmark", "Denmark", "Denmark", "Denmark", "Denmark", "Denmark", "Denmark", "Denmark", "Denmark", "Denmark", "Denmark", "Denmark", "England", "England",  "England", "England", "England", "England",  "England", "England", "England", "England",  "England", "England", "England", "England",  "England", "England", "England", "England",  "England", "England", "England", "England"))

n <- nrow(DE11_all_train)
# placeholder for storing the i-th prediction
preds <- rep(NA, n)
for(i in 1:n) {
    dataf.train <- DE11_all_train[-i, ]
    dataf.test <- DE11_all_train[i, ]
    groups_tmp <- groups[-i]
    set.seed(123) 
    cv.lasso <- cv.glmnet(as.matrix(dataf.train), groups_tmp, alpha = a, family = "binomial")
    glmnet.fit <- glmnet(as.matrix(dataf.train), groups_tmp, alpha = a, family = "binomial", lambda = cv.lasso$lambda.min)
    preds[i] <- predict(glmnet.fit, as.matrix(dataf.test), type="response")
}

# define the number of elements in class 1
threshold <- seq(0, 1, length.out = 1000)
accuracy <- c()
for (t in threshold){
  tot <- 0
  for (i in 1:(length(preds))){
    if ((groups[i] == "Denmark" && preds[i] < (1 - t)) || (groups[i] == "England" && preds[i] > (t))){
      tot <- tot + 1
    }
  }
  prop <- tot/length(preds)
  accuracy <- c(accuracy, prop)
}  
DE11_all_geo <- accuracy
```

# DE 15bp

Read data into R: (all the samples)

```
# import data
DE15_all_mash <- read.csv("kmer_size_comparison/DE_15bp.csv") 
# Add row names
rownames(DE15_all_mash) <- names(DE15_all_mash)
# Transform to matrix object for plotting
DE15_all_mash_mat <- as.matrix(DE15_all_mash)
```

DE dataset Plot MDS (color according to geography) - also low-depth samples orange = Denmark purple = England

```
library(ggfortify)
DE15_all_pca_mat <- prcomp(DE15_all_mash_mat, scale. = TRUE)
DE15_all_col_vec <- c("#d95f02", "#d95f02", "#d95f02", "#d95f02", "#d95f02", "#d95f02", "#d95f02", "#d95f02", "#d95f02", "#d95f02", "#d95f02", "#d95f02", "#d95f02", "#d95f02", "#d95f02", "#d95f02", "#d95f02", "#d95f02", "#d95f02", "#d95f02", "#d95f02", "#d95f02", "#d95f02", "#d95f02", "#d95f02", "#d95f02", "#d95f02", "#d95f02", "#d95f02", "#7570b3", "#7570b3", "#7570b3", "#7570b3", "#7570b3", "#7570b3", "#7570b3", "#7570b3", "#7570b3", "#7570b3", "#7570b3", "#7570b3", "#7570b3", "#7570b3", "#7570b3", "#7570b3", "#7570b3", "#7570b3", "#7570b3", "#7570b3", "#7570b3", "#7570b3")

autoplot(DE15_all_pca_mat, x = 1, y = 3, pch = 16, label.size = 2, size = 6, alpha = 0.7, label = FALSE, label.colour = NULL, label.hjust=0.0001, label.vjust=-0.5,
        colour = DE15_all_col_vec) +
        theme_bw()
```

```
DE15_all_train <- as.data.frame(DE15_all_pca_mat$x)

# geo:
groups <- factor(c("Denmark", "Denmark", "Denmark", "Denmark", "Denmark", "Denmark", "Denmark", "Denmark", "Denmark", "Denmark", "Denmark", "Denmark", "Denmark", "Denmark", "Denmark", "Denmark", "Denmark", "Denmark", "Denmark", "Denmark", "Denmark", "Denmark", "Denmark", "Denmark", "Denmark", "Denmark", "Denmark", "Denmark", "Denmark", "England", "England",  "England", "England", "England", "England",  "England", "England", "England", "England",  "England", "England", "England", "England",  "England", "England", "England", "England",  "England", "England", "England", "England"))

n <- nrow(DE15_all_train)
# placeholder for storing the i-th prediction
preds <- rep(NA, n)
for(i in 1:n) {
    dataf.train <- DE15_all_train[-i, ]
    dataf.test <- DE15_all_train[i, ]
    groups_tmp <- groups[-i]
    set.seed(123) 
    cv.lasso <- cv.glmnet(as.matrix(dataf.train), groups_tmp, alpha = a, family = "binomial")
    glmnet.fit <- glmnet(as.matrix(dataf.train), groups_tmp, alpha = a, family = "binomial", lambda = cv.lasso$lambda.min)
    preds[i] <- predict(glmnet.fit, as.matrix(dataf.test), type="response")
}

# define the number of elements in class 1
threshold <- seq(0, 1, length.out = 1000)
accuracy <- c()
for (t in threshold){
  tot <- 0
  for (i in 1:(length(preds))){
    if ((groups[i] == "Denmark" && preds[i] < (1 - t)) || (groups[i] == "England" && preds[i] > (t))){
      tot <- tot + 1
    }
  }
  prop <- tot/length(preds)
  accuracy <- c(accuracy, prop)
}  
DE15_all_geo <- accuracy
```

# DE 25bp

Read data into R: (all the samples)

```
# import data
DE25_all_mash <- read.csv("kmer_size_comparison/DE_25bp.csv") 
# Add row names
rownames(DE25_all_mash) <- names(DE25_all_mash)
# Transform to matrix object for plotting
DE25_all_mash_mat <- as.matrix(DE25_all_mash)
```

DE dataset Plot MDS (color according to geography) - also low-depth samples orange = Denmark purple = England

```
library(ggfortify)
DE25_all_pca_mat <- prcomp(DE25_all_mash_mat, scale. = TRUE)
DE25_all_col_vec <- c("#d95f02", "#d95f02", "#d95f02", "#d95f02", "#d95f02", "#d95f02", "#d95f02", "#d95f02", "#d95f02", "#d95f02", "#d95f02", "#d95f02", "#d95f02", "#d95f02", "#d95f02", "#d95f02", "#d95f02", "#d95f02", "#d95f02", "#d95f02", "#d95f02", "#d95f02", "#d95f02", "#d95f02", "#d95f02", "#d95f02", "#d95f02", "#d95f02", "#d95f02", "#7570b3", "#7570b3", "#7570b3", "#7570b3", "#7570b3", "#7570b3", "#7570b3", "#7570b3", "#7570b3", "#7570b3", "#7570b3", "#7570b3", "#7570b3", "#7570b3", "#7570b3", "#7570b3", "#7570b3", "#7570b3", "#7570b3", "#7570b3", "#7570b3", "#7570b3")

autoplot(DE25_all_pca_mat, x = 1, y = 3, pch = 16, label.size = 2, size = 6, alpha = 0.7, label = FALSE, label.colour = NULL, label.hjust=0.0001, label.vjust=-0.5,
        colour = DE25_all_col_vec) +
        theme_bw()
```

```
DE25_all_train <- as.data.frame(DE25_all_pca_mat$x)

# geo:
groups <- factor(c("Denmark", "Denmark", "Denmark", "Denmark", "Denmark", "Denmark", "Denmark", "Denmark", "Denmark", "Denmark", "Denmark", "Denmark", "Denmark", "Denmark", "Denmark", "Denmark", "Denmark", "Denmark", "Denmark", "Denmark", "Denmark", "Denmark", "Denmark", "Denmark", "Denmark", "Denmark", "Denmark", "Denmark", "Denmark", "England", "England",  "England", "England", "England", "England",  "England", "England", "England", "England",  "England", "England", "England", "England",  "England", "England", "England", "England",  "England", "England", "England", "England"))

n <- nrow(DE25_all_train)
# placeholder for storing the i-th prediction
preds <- rep(NA, n)
for(i in 1:n) {
    dataf.train <- DE25_all_train[-i, ]
    dataf.test <- DE25_all_train[i, ]
    groups_tmp <- groups[-i]
    set.seed(123) 
    cv.lasso <- cv.glmnet(as.matrix(dataf.train), groups_tmp, alpha = a, family = "binomial")
    glmnet.fit <- glmnet(as.matrix(dataf.train), groups_tmp, alpha = a, family = "binomial", lambda = cv.lasso$lambda.min)
    preds[i] <- predict(glmnet.fit, as.matrix(dataf.test), type="response")
}

# define the number of elements in class 1
threshold <- seq(0, 1, length.out = 1000)
accuracy <- c()
for (t in threshold){
  tot <- 0
  for (i in 1:(length(preds))){
    if ((groups[i] == "Denmark" && preds[i] < (1 - t)) || (groups[i] == "England" && preds[i] > (t))){
      tot <- tot + 1
    }
  }
  prop <- tot/length(preds)
  accuracy <- c(accuracy, prop)
}  
DE25_all_geo <- accuracy
```

# DE 31bp

Read data into R: (all the samples)

```
# import data
DE31_all_mash <- read.csv("kmer_size_comparison/DE_31bp.csv") 
# Add row names
rownames(DE31_all_mash) <- names(DE31_all_mash)
# Transform to matrix object for plotting
DE31_all_mash_mat <- as.matrix(DE31_all_mash)
```

DE dataset Plot MDS (color according to geography) - also low-depth samples orange = Denmark purple = England

```
library(ggfortify)
DE31_all_pca_mat <- prcomp(DE31_all_mash_mat, scale. = TRUE)
DE31_all_col_vec <- c("#d95f02", "#d95f02", "#d95f02", "#d95f02", "#d95f02", "#d95f02", "#d95f02", "#d95f02", "#d95f02", "#d95f02", "#d95f02", "#d95f02", "#d95f02", "#d95f02", "#d95f02", "#d95f02", "#d95f02", "#d95f02", "#d95f02", "#d95f02", "#d95f02", "#d95f02", "#d95f02", "#d95f02", "#d95f02", "#d95f02", "#d95f02", "#d95f02", "#d95f02", "#7570b3", "#7570b3", "#7570b3", "#7570b3", "#7570b3", "#7570b3", "#7570b3", "#7570b3", "#7570b3", "#7570b3", "#7570b3", "#7570b3", "#7570b3", "#7570b3", "#7570b3", "#7570b3", "#7570b3", "#7570b3", "#7570b3", "#7570b3", "#7570b3", "#7570b3")

autoplot(DE31_all_pca_mat, x = 1, y = 3, pch = 16, label.size = 2, size = 6, alpha = 0.7, label = FALSE, label.colour = NULL, label.hjust=0.0001, label.vjust=-0.5,
        colour = DE31_all_col_vec) +
        theme_bw()
```

```
DE31_all_train <- as.data.frame(DE31_all_pca_mat$x)

# geo:
groups <- factor(c("Denmark", "Denmark", "Denmark", "Denmark", "Denmark", "Denmark", "Denmark", "Denmark", "Denmark", "Denmark", "Denmark", "Denmark", "Denmark", "Denmark", "Denmark", "Denmark", "Denmark", "Denmark", "Denmark", "Denmark", "Denmark", "Denmark", "Denmark", "Denmark", "Denmark", "Denmark", "Denmark", "Denmark", "Denmark", "England", "England",  "England", "England", "England", "England",  "England", "England", "England", "England",  "England", "England", "England", "England",  "England", "England", "England", "England",  "England", "England", "England", "England"))

n <- nrow(DE31_all_train)
# placeholder for storing the i-th prediction
preds <- rep(NA, n)
for(i in 1:n) {
    dataf.train <- DE31_all_train[-i, ]
    dataf.test <- DE31_all_train[i, ]
    groups_tmp <- groups[-i]
    set.seed(123) 
    cv.lasso <- cv.glmnet(as.matrix(dataf.train), groups_tmp, alpha = a, family = "binomial")
    glmnet.fit <- glmnet(as.matrix(dataf.train), groups_tmp, alpha = a, family = "binomial", lambda = cv.lasso$lambda.min)
    preds[i] <- predict(glmnet.fit, as.matrix(dataf.test), type="response")
}

# define the number of elements in class 1
threshold <- seq(0, 1, length.out = 1000)
accuracy <- c()
for (t in threshold){
  tot <- 0
  for (i in 1:(length(preds))){
    if ((groups[i] == "Denmark" && preds[i] < (1 - t)) || (groups[i] == "England" && preds[i] > (t))){
      tot <- tot + 1
    }
  }
  prop <- tot/length(preds)
  accuracy <- c(accuracy, prop)
}  
DE31_all_geo <- accuracy
```

# geo accuracy D-E dataset:

```
ggplot() + 
  geom_line(aes(threshold, DE11_all_geo), color = "yellow1") +
  geom_line(aes(threshold, DE15_all_geo), color = "gold1") +
  geom_line(aes(threshold, DE_all_geo), color = "orange2") +
  geom_line(aes(threshold, DE25_all_geo), color = "red2") +
  geom_line(aes(threshold, DE31_all_geo), color = "red4") +
  theme_bw() +
  ylim(0, 1)
```

# only best samples:

# 21bp

Read DE data into R: (only samples with >1 million reads)

```
# import data
DE_best_mash <- read.csv("kmers_similarity_matrices/DE_similarity_matrix.csv")
# Add row names
rownames(DE_best_mash) <- names(DE_best_mash)
# Transform to matrix object for plotting
DE_best_mash_mat <- as.matrix(DE_best_mash)
```

DE dataset Plot MDS (color according to geography) - only high-depth samples orange = Denmark purple = England

```
library(ggfortify)
DE_best_pca_mat <- prcomp(DE_best_mash_mat, scale. = TRUE)
DE_best_col_vec <- c("#d95f02", "#d95f02", "#d95f02", "#d95f02", "#d95f02", "#d95f02", "#d95f02", "#d95f02", "#d95f02", "#d95f02", "#d95f02", "#d95f02", "#d95f02", "#d95f02", "#d95f02", "#d95f02", "#d95f02", "#d95f02", "#d95f02", "#d95f02", "#d95f02", "#d95f02", "#d95f02", "#d95f02", "#7570b3", "#7570b3", "#7570b3", "#7570b3", "#7570b3", "#7570b3", "#7570b3", "#7570b3", "#7570b3", "#7570b3", "#7570b3")

autoplot(DE_best_pca_mat, x = 1, y = 2, pch = 16, label.size = 2, size = 4, alpha = 0.7, label = F, label.colour = NULL, label.hjust=0.0001, label.vjust=-0.5,
         colour = DE_best_col_vec) +
  theme_bw()
```

```
autoplot(DE_best_pca_mat, x = 2, y = 3, pch = 16, label.size = 2, size = 4, alpha = 0.7, label = F, label.colour = NULL, label.hjust=0.0001, label.vjust=-0.5,
         colour = DE_best_col_vec) +
  theme_bw()
```

## Model training and accuracy evalutation D-E dataset:

D-E dataset (high-depth samples) geography variable:

```
DE_best_train <- as.data.frame(DE_best_pca_mat$x)
# extraction protocol:
groups<- as.factor(c("Denmark", "Denmark", "Denmark", "Denmark", "Denmark", "Denmark", "Denmark", "Denmark", "Denmark", "Denmark", "Denmark", "Denmark", "Denmark", "Denmark", "Denmark", "Denmark", "Denmark", "Denmark", "Denmark", "Denmark", "Denmark", "Denmark", "Denmark", "Denmark", "England", "England", "England", "England", "England", "England", "England", "England", "England", "England", "England"))

n <- nrow(DE_best_train)
# placeholder for storing the i-th prediction
preds <- rep(NA, n)
for(i in 1:n) {
    dataf.train <- DE_best_train[-i, ]
    dataf.test <- DE_best_train[i, ]
    groups_tmp <- groups[-i]
    set.seed(123) 
    cv.lasso <- cv.glmnet(as.matrix(dataf.train), groups_tmp, alpha = a, family = "binomial")
    glmnet.fit <- glmnet(as.matrix(dataf.train), groups_tmp, alpha = a, family = "binomial", lambda = cv.lasso$lambda.min)
    preds[i] <- predict(glmnet.fit, as.matrix(dataf.test), type="response")
}
```

```
## Warning in lognet(xd, is.sparse, ix, jx, y, weights, offset, alpha, nobs, : one
## multinomial or binomial class has fewer than 8 observations; dangerous ground

## Warning in lognet(xd, is.sparse, ix, jx, y, weights, offset, alpha, nobs, : one
## multinomial or binomial class has fewer than 8 observations; dangerous ground

## Warning in lognet(xd, is.sparse, ix, jx, y, weights, offset, alpha, nobs, : one
## multinomial or binomial class has fewer than 8 observations; dangerous ground

## Warning in lognet(xd, is.sparse, ix, jx, y, weights, offset, alpha, nobs, : one
## multinomial or binomial class has fewer than 8 observations; dangerous ground

## Warning in lognet(xd, is.sparse, ix, jx, y, weights, offset, alpha, nobs, : one
## multinomial or binomial class has fewer than 8 observations; dangerous ground

## Warning in lognet(xd, is.sparse, ix, jx, y, weights, offset, alpha, nobs, : one
## multinomial or binomial class has fewer than 8 observations; dangerous ground

## Warning in lognet(xd, is.sparse, ix, jx, y, weights, offset, alpha, nobs, : one
## multinomial or binomial class has fewer than 8 observations; dangerous ground

## Warning in lognet(xd, is.sparse, ix, jx, y, weights, offset, alpha, nobs, : one
## multinomial or binomial class has fewer than 8 observations; dangerous ground

## Warning in lognet(xd, is.sparse, ix, jx, y, weights, offset, alpha, nobs, : one
## multinomial or binomial class has fewer than 8 observations; dangerous ground

## Warning in lognet(xd, is.sparse, ix, jx, y, weights, offset, alpha, nobs, : one
## multinomial or binomial class has fewer than 8 observations; dangerous ground

## Warning in lognet(xd, is.sparse, ix, jx, y, weights, offset, alpha, nobs, : one
## multinomial or binomial class has fewer than 8 observations; dangerous ground
```

```
# define the number of elements in class 1
threshold <- seq(0, 1, length.out = 1000)
accuracy <- c()
for (t in threshold){
  tot <- 0
  for (i in 1:(length(preds))){
    if ((groups[i] == "Denmark" && preds[i] < (1 - t)) || (groups[i] == "England" && preds[i] > (t))){
      tot <- tot + 1
    }
  }
  prop <- tot/length(preds)
  accuracy <- c(accuracy, prop)
}  
DE_best_geo <- accuracy
```

# DE 11bp

Read data into R: (only best samples (reads >1million))

```
# import data
DE11_best_mash <- read.csv("kmer_size_comparison/DE_11bp_best_samples.csv") 
# Add row names
rownames(DE11_best_mash) <- names(DE11_best_mash)
# Transform to matrix object for plotting
DE11_best_mash_mat <- as.matrix(DE11_best_mash)
```

DE dataset Plot MDS (color according to geography) - only high-depth samples orange = Denmark purple = England

```
library(ggfortify)
DE11_best_pca_mat <- prcomp(DE11_best_mash_mat, scale. = TRUE)
DE11_best_col_vec <- c("#d95f02", "#d95f02", "#d95f02", "#d95f02", "#d95f02", "#d95f02", "#d95f02", "#d95f02", "#d95f02", "#d95f02", "#d95f02", "#d95f02", "#d95f02", "#d95f02", "#d95f02", "#d95f02", "#d95f02", "#d95f02", "#d95f02", "#d95f02", "#d95f02", "#d95f02", "#d95f02", "#d95f02", "#7570b3", "#7570b3", "#7570b3", "#7570b3", "#7570b3", "#7570b3", "#7570b3", "#7570b3", "#7570b3", "#7570b3", "#7570b3")

autoplot(DE11_best_pca_mat, x = 1, y = 2, pch = 16, label.size = 2, size = 4, alpha = 0.7, label = F, label.colour = NULL, label.hjust=0.0001, label.vjust=-0.5,
         colour = DE11_best_col_vec) +
  theme_bw()
```

```
autoplot(DE11_best_pca_mat, x = 2, y = 3, pch = 16, label.size = 2, size = 4, alpha = 0.7, label = F, label.colour = NULL, label.hjust=0.0001, label.vjust=-0.5,
         colour = DE11_best_col_vec) +
  theme_bw()
```

## Model training and accuracy evalutation D-E dataset:

D-E dataset (high-depth samples) geography variable:

```
DE11_best_train <- as.data.frame(DE11_best_pca_mat$x)
# extraction protocol:
groups<- as.factor(c("Denmark", "Denmark", "Denmark", "Denmark", "Denmark", "Denmark", "Denmark", "Denmark", "Denmark", "Denmark", "Denmark", "Denmark", "Denmark", "Denmark", "Denmark", "Denmark", "Denmark", "Denmark", "Denmark", "Denmark", "Denmark", "Denmark", "Denmark", "Denmark", "England", "England", "England", "England", "England", "England", "England", "England", "England", "England", "England"))

n <- nrow(DE11_best_train)
# placeholder for storing the i-th prediction
preds <- rep(NA, n)
for(i in 1:n) {
    dataf.train <- DE11_best_train[-i, ]
    dataf.test <- DE11_best_train[i, ]
    groups_tmp <- groups[-i]
    set.seed(123) 
    cv.lasso <- cv.glmnet(as.matrix(dataf.train), groups_tmp, alpha = a, family = "binomial")
    glmnet.fit <- glmnet(as.matrix(dataf.train), groups_tmp, alpha = a, family = "binomial", lambda = cv.lasso$lambda.min)
    preds[i] <- predict(glmnet.fit, as.matrix(dataf.test), type="response")
}
```

```
## Warning in lognet(xd, is.sparse, ix, jx, y, weights, offset, alpha, nobs, : one
## multinomial or binomial class has fewer than 8 observations; dangerous ground

## Warning in lognet(xd, is.sparse, ix, jx, y, weights, offset, alpha, nobs, : one
## multinomial or binomial class has fewer than 8 observations; dangerous ground

## Warning in lognet(xd, is.sparse, ix, jx, y, weights, offset, alpha, nobs, : one
## multinomial or binomial class has fewer than 8 observations; dangerous ground

## Warning in lognet(xd, is.sparse, ix, jx, y, weights, offset, alpha, nobs, : one
## multinomial or binomial class has fewer than 8 observations; dangerous ground

## Warning in lognet(xd, is.sparse, ix, jx, y, weights, offset, alpha, nobs, : one
## multinomial or binomial class has fewer than 8 observations; dangerous ground

## Warning in lognet(xd, is.sparse, ix, jx, y, weights, offset, alpha, nobs, : one
## multinomial or binomial class has fewer than 8 observations; dangerous ground

## Warning in lognet(xd, is.sparse, ix, jx, y, weights, offset, alpha, nobs, : one
## multinomial or binomial class has fewer than 8 observations; dangerous ground

## Warning in lognet(xd, is.sparse, ix, jx, y, weights, offset, alpha, nobs, : one
## multinomial or binomial class has fewer than 8 observations; dangerous ground

## Warning in lognet(xd, is.sparse, ix, jx, y, weights, offset, alpha, nobs, : one
## multinomial or binomial class has fewer than 8 observations; dangerous ground

## Warning in lognet(xd, is.sparse, ix, jx, y, weights, offset, alpha, nobs, : one
## multinomial or binomial class has fewer than 8 observations; dangerous ground

## Warning in lognet(xd, is.sparse, ix, jx, y, weights, offset, alpha, nobs, : one
## multinomial or binomial class has fewer than 8 observations; dangerous ground
```

```
# define the number of elements in class 1
threshold <- seq(0, 1, length.out = 1000)
accuracy <- c()
for (t in threshold){
  tot <- 0
  for (i in 1:(length(preds))){
    if ((groups[i] == "Denmark" && preds[i] < (1 - t)) || (groups[i] == "England" && preds[i] > (t))){
      tot <- tot + 1
    }
  }
  prop <- tot/length(preds)
  accuracy <- c(accuracy, prop)
}  
DE11_best_geo <- accuracy
```

# DE 15bp

Read data into R: (only best samples (reads >1million))

```
# import data
DE15_best_mash <- read.csv("kmer_size_comparison/DE_15bp_best_samples.csv") 
# Add row names
rownames(DE15_best_mash) <- names(DE15_best_mash)
# Transform to matrix object for plotting
DE15_best_mash_mat <- as.matrix(DE15_best_mash)
```

DE dataset Plot MDS (color according to geography) - only high-depth samples orange = Denmark purple = England

```
library(ggfortify)
DE15_best_pca_mat <- prcomp(DE15_best_mash_mat, scale. = TRUE)
DE15_best_col_vec <- c("#d95f02", "#d95f02", "#d95f02", "#d95f02", "#d95f02", "#d95f02", "#d95f02", "#d95f02", "#d95f02", "#d95f02", "#d95f02", "#d95f02", "#d95f02", "#d95f02", "#d95f02", "#d95f02", "#d95f02", "#d95f02", "#d95f02", "#d95f02", "#d95f02", "#d95f02", "#d95f02", "#d95f02", "#7570b3", "#7570b3", "#7570b3", "#7570b3", "#7570b3", "#7570b3", "#7570b3", "#7570b3", "#7570b3", "#7570b3", "#7570b3")

autoplot(DE15_best_pca_mat, x = 1, y = 2, pch = 16, label.size = 2, size = 4, alpha = 0.7, label = F, label.colour = NULL, label.hjust=0.0001, label.vjust=-0.5,
         colour = DE15_best_col_vec) +
  theme_bw()
```

```
autoplot(DE15_best_pca_mat, x = 2, y = 3, pch = 16, label.size = 2, size = 4, alpha = 0.7, label = F, label.colour = NULL, label.hjust=0.0001, label.vjust=-0.5,
         colour = DE15_best_col_vec) +
  theme_bw()
```

## Model training and accuracy evalutation D-E dataset:

D-E dataset (high-depth samples) geography variable:

```
DE15_best_train <- as.data.frame(DE15_best_pca_mat$x)
# extraction protocol:
groups<- as.factor(c("Denmark", "Denmark", "Denmark", "Denmark", "Denmark", "Denmark", "Denmark", "Denmark", "Denmark", "Denmark", "Denmark", "Denmark", "Denmark", "Denmark", "Denmark", "Denmark", "Denmark", "Denmark", "Denmark", "Denmark", "Denmark", "Denmark", "Denmark", "Denmark", "England", "England", "England", "England", "England", "England", "England", "England", "England", "England", "England"))

n <- nrow(DE15_best_train)
# placeholder for storing the i-th prediction
preds <- rep(NA, n)
for(i in 1:n) {
    dataf.train <- DE15_best_train[-i, ]
    dataf.test <- DE15_best_train[i, ]
    groups_tmp <- groups[-i]
    set.seed(123) 
    cv.lasso <- cv.glmnet(as.matrix(dataf.train), groups_tmp, alpha = a, family = "binomial")
    glmnet.fit <- glmnet(as.matrix(dataf.train), groups_tmp, alpha = a, family = "binomial", lambda = cv.lasso$lambda.min)
    preds[i] <- predict(glmnet.fit, as.matrix(dataf.test), type="response")
}
```

```
## Warning in lognet(xd, is.sparse, ix, jx, y, weights, offset, alpha, nobs, : one
## multinomial or binomial class has fewer than 8 observations; dangerous ground

## Warning in lognet(xd, is.sparse, ix, jx, y, weights, offset, alpha, nobs, : one
## multinomial or binomial class has fewer than 8 observations; dangerous ground

## Warning in lognet(xd, is.sparse, ix, jx, y, weights, offset, alpha, nobs, : one
## multinomial or binomial class has fewer than 8 observations; dangerous ground

## Warning in lognet(xd, is.sparse, ix, jx, y, weights, offset, alpha, nobs, : one
## multinomial or binomial class has fewer than 8 observations; dangerous ground

## Warning in lognet(xd, is.sparse, ix, jx, y, weights, offset, alpha, nobs, : one
## multinomial or binomial class has fewer than 8 observations; dangerous ground

## Warning in lognet(xd, is.sparse, ix, jx, y, weights, offset, alpha, nobs, : one
## multinomial or binomial class has fewer than 8 observations; dangerous ground

## Warning in lognet(xd, is.sparse, ix, jx, y, weights, offset, alpha, nobs, : one
## multinomial or binomial class has fewer than 8 observations; dangerous ground

## Warning in lognet(xd, is.sparse, ix, jx, y, weights, offset, alpha, nobs, : one
## multinomial or binomial class has fewer than 8 observations; dangerous ground

## Warning in lognet(xd, is.sparse, ix, jx, y, weights, offset, alpha, nobs, : one
## multinomial or binomial class has fewer than 8 observations; dangerous ground

## Warning in lognet(xd, is.sparse, ix, jx, y, weights, offset, alpha, nobs, : one
## multinomial or binomial class has fewer than 8 observations; dangerous ground

## Warning in lognet(xd, is.sparse, ix, jx, y, weights, offset, alpha, nobs, : one
## multinomial or binomial class has fewer than 8 observations; dangerous ground
```

```
# define the number of elements in class 1
threshold <- seq(0, 1, length.out = 1000)
accuracy <- c()
for (t in threshold){
  tot <- 0
  for (i in 1:(length(preds))){
    if ((groups[i] == "Denmark" && preds[i] < (1 - t)) || (groups[i] == "England" && preds[i] > (t))){
      tot <- tot + 1
    }
  }
  prop <- tot/length(preds)
  accuracy <- c(accuracy, prop)
}  
DE15_best_geo <- accuracy
```

# DE 25bp

Read data into R: (only best samples (reads >1million))

```
# import data
DE25_best_mash <- read.csv("kmer_size_comparison/DE_25bp_best_samples.csv") 
# Add row names
rownames(DE25_best_mash) <- names(DE25_best_mash)
# Transform to matrix object for plotting
DE25_best_mash_mat <- as.matrix(DE25_best_mash)
```

DE dataset Plot MDS (color according to geography) - only high-depth samples orange = Denmark purple = England

```
library(ggfortify)
DE25_best_pca_mat <- prcomp(DE25_best_mash_mat, scale. = TRUE)
DE25_best_col_vec <- c("#d95f02", "#d95f02", "#d95f02", "#d95f02", "#d95f02", "#d95f02", "#d95f02", "#d95f02", "#d95f02", "#d95f02", "#d95f02", "#d95f02", "#d95f02", "#d95f02", "#d95f02", "#d95f02", "#d95f02", "#d95f02", "#d95f02", "#d95f02", "#d95f02", "#d95f02", "#d95f02", "#d95f02", "#7570b3", "#7570b3", "#7570b3", "#7570b3", "#7570b3", "#7570b3", "#7570b3", "#7570b3", "#7570b3", "#7570b3", "#7570b3")

autoplot(DE25_best_pca_mat, x = 1, y = 2, pch = 16, label.size = 2, size = 4, alpha = 0.7, label = F, label.colour = NULL, label.hjust=0.0001, label.vjust=-0.5,
         colour = DE25_best_col_vec) +
  theme_bw()
```

```
autoplot(DE25_best_pca_mat, x = 2, y = 3, pch = 16, label.size = 2, size = 4, alpha = 0.7, label = F, label.colour = NULL, label.hjust=0.0001, label.vjust=-0.5,
         colour = DE25_best_col_vec) +
  theme_bw()
```

## Model training and accuracy evalutation D-E dataset:

D-E dataset (high-depth samples) geography variable:

```
DE25_best_train <- as.data.frame(DE25_best_pca_mat$x)
# extraction protocol:
groups<- as.factor(c("Denmark", "Denmark", "Denmark", "Denmark", "Denmark", "Denmark", "Denmark", "Denmark", "Denmark", "Denmark", "Denmark", "Denmark", "Denmark", "Denmark", "Denmark", "Denmark", "Denmark", "Denmark", "Denmark", "Denmark", "Denmark", "Denmark", "Denmark", "Denmark", "England", "England", "England", "England", "England", "England", "England", "England", "England", "England", "England"))

n <- nrow(DE25_best_train)
# placeholder for storing the i-th prediction
preds <- rep(NA, n)
for(i in 1:n) {
    dataf.train <- DE25_best_train[-i, ]
    dataf.test <- DE25_best_train[i, ]
    groups_tmp <- groups[-i]
    set.seed(123) 
    cv.lasso <- cv.glmnet(as.matrix(dataf.train), groups_tmp, alpha = a, family = "binomial")
    glmnet.fit <- glmnet(as.matrix(dataf.train), groups_tmp, alpha = a, family = "binomial", lambda = cv.lasso$lambda.min)
    preds[i] <- predict(glmnet.fit, as.matrix(dataf.test), type="response")
}
```

```
## Warning in lognet(xd, is.sparse, ix, jx, y, weights, offset, alpha, nobs, : one
## multinomial or binomial class has fewer than 8 observations; dangerous ground

## Warning in lognet(xd, is.sparse, ix, jx, y, weights, offset, alpha, nobs, : one
## multinomial or binomial class has fewer than 8 observations; dangerous ground

## Warning in lognet(xd, is.sparse, ix, jx, y, weights, offset, alpha, nobs, : one
## multinomial or binomial class has fewer than 8 observations; dangerous ground

## Warning in lognet(xd, is.sparse, ix, jx, y, weights, offset, alpha, nobs, : one
## multinomial or binomial class has fewer than 8 observations; dangerous ground

## Warning in lognet(xd, is.sparse, ix, jx, y, weights, offset, alpha, nobs, : one
## multinomial or binomial class has fewer than 8 observations; dangerous ground

## Warning in lognet(xd, is.sparse, ix, jx, y, weights, offset, alpha, nobs, : one
## multinomial or binomial class has fewer than 8 observations; dangerous ground

## Warning in lognet(xd, is.sparse, ix, jx, y, weights, offset, alpha, nobs, : one
## multinomial or binomial class has fewer than 8 observations; dangerous ground

## Warning in lognet(xd, is.sparse, ix, jx, y, weights, offset, alpha, nobs, : one
## multinomial or binomial class has fewer than 8 observations; dangerous ground

## Warning in lognet(xd, is.sparse, ix, jx, y, weights, offset, alpha, nobs, : one
## multinomial or binomial class has fewer than 8 observations; dangerous ground

## Warning in lognet(xd, is.sparse, ix, jx, y, weights, offset, alpha, nobs, : one
## multinomial or binomial class has fewer than 8 observations; dangerous ground

## Warning in lognet(xd, is.sparse, ix, jx, y, weights, offset, alpha, nobs, : one
## multinomial or binomial class has fewer than 8 observations; dangerous ground
```

```
# define the number of elements in class 1
threshold <- seq(0, 1, length.out = 1000)
accuracy <- c()
for (t in threshold){
  tot <- 0
  for (i in 1:(length(preds))){
    if ((groups[i] == "Denmark" && preds[i] < (1 - t)) || (groups[i] == "England" && preds[i] > (t))){
      tot <- tot + 1
    }
  }
  prop <- tot/length(preds)
  accuracy <- c(accuracy, prop)
}  
DE25_best_geo <- accuracy
```

# DE 31bp

Read data into R: (only best samples (reads >1million))

```
# import data
DE31_best_mash <- read.csv("kmer_size_comparison/DE_31bp_best_samples.csv") 
# Add row names
rownames(DE31_best_mash) <- names(DE31_best_mash)
# Transform to matrix object for plotting
DE31_best_mash_mat <- as.matrix(DE31_best_mash)
```

DE dataset Plot MDS (color according to geography) - only high-depth samples orange = Denmark purple = England

```
library(ggfortify)
DE31_best_pca_mat <- prcomp(DE31_best_mash_mat, scale. = TRUE)
DE31_best_col_vec <- c("#d95f02", "#d95f02", "#d95f02", "#d95f02", "#d95f02", "#d95f02", "#d95f02", "#d95f02", "#d95f02", "#d95f02", "#d95f02", "#d95f02", "#d95f02", "#d95f02", "#d95f02", "#d95f02", "#d95f02", "#d95f02", "#d95f02", "#d95f02", "#d95f02", "#d95f02", "#d95f02", "#d95f02", "#7570b3", "#7570b3", "#7570b3", "#7570b3", "#7570b3", "#7570b3", "#7570b3", "#7570b3", "#7570b3", "#7570b3", "#7570b3")

autoplot(DE31_best_pca_mat, x = 1, y = 2, pch = 16, label.size = 2, size = 4, alpha = 0.7, label = F, label.colour = NULL, label.hjust=0.0001, label.vjust=-0.5,
         colour = DE31_best_col_vec) +
  theme_bw()
```

```
autoplot(DE31_best_pca_mat, x = 2, y = 3, pch = 16, label.size = 2, size = 4, alpha = 0.7, label = F, label.colour = NULL, label.hjust=0.0001, label.vjust=-0.5,
         colour = DE31_best_col_vec) +
  theme_bw()
```

## Model training and accuracy evalutation D-E dataset:

D-E dataset (high-depth samples) geography variable:

```
DE31_best_train <- as.data.frame(DE31_best_pca_mat$x)
# extraction protocol:
groups<- as.factor(c("Denmark", "Denmark", "Denmark", "Denmark", "Denmark", "Denmark", "Denmark", "Denmark", "Denmark", "Denmark", "Denmark", "Denmark", "Denmark", "Denmark", "Denmark", "Denmark", "Denmark", "Denmark", "Denmark", "Denmark", "Denmark", "Denmark", "Denmark", "Denmark", "England", "England", "England", "England", "England", "England", "England", "England", "England", "England", "England"))

n <- nrow(DE31_best_train)
# placeholder for storing the i-th prediction
preds <- rep(NA, n)
for(i in 1:n) {
    dataf.train <- DE31_best_train[-i, ]
    dataf.test <- DE31_best_train[i, ]
    groups_tmp <- groups[-i]
    set.seed(123) 
    cv.lasso <- cv.glmnet(as.matrix(dataf.train), groups_tmp, alpha = a, family = "binomial")
    glmnet.fit <- glmnet(as.matrix(dataf.train), groups_tmp, alpha = a, family = "binomial", lambda = cv.lasso$lambda.min)
    preds[i] <- predict(glmnet.fit, as.matrix(dataf.test), type="response")
}
```

```
## Warning in lognet(xd, is.sparse, ix, jx, y, weights, offset, alpha, nobs, : one
## multinomial or binomial class has fewer than 8 observations; dangerous ground

## Warning in lognet(xd, is.sparse, ix, jx, y, weights, offset, alpha, nobs, : one
## multinomial or binomial class has fewer than 8 observations; dangerous ground

## Warning in lognet(xd, is.sparse, ix, jx, y, weights, offset, alpha, nobs, : one
## multinomial or binomial class has fewer than 8 observations; dangerous ground

## Warning in lognet(xd, is.sparse, ix, jx, y, weights, offset, alpha, nobs, : one
## multinomial or binomial class has fewer than 8 observations; dangerous ground

## Warning in lognet(xd, is.sparse, ix, jx, y, weights, offset, alpha, nobs, : one
## multinomial or binomial class has fewer than 8 observations; dangerous ground

## Warning in lognet(xd, is.sparse, ix, jx, y, weights, offset, alpha, nobs, : one
## multinomial or binomial class has fewer than 8 observations; dangerous ground

## Warning in lognet(xd, is.sparse, ix, jx, y, weights, offset, alpha, nobs, : one
## multinomial or binomial class has fewer than 8 observations; dangerous ground

## Warning in lognet(xd, is.sparse, ix, jx, y, weights, offset, alpha, nobs, : one
## multinomial or binomial class has fewer than 8 observations; dangerous ground

## Warning in lognet(xd, is.sparse, ix, jx, y, weights, offset, alpha, nobs, : one
## multinomial or binomial class has fewer than 8 observations; dangerous ground

## Warning in lognet(xd, is.sparse, ix, jx, y, weights, offset, alpha, nobs, : one
## multinomial or binomial class has fewer than 8 observations; dangerous ground

## Warning in lognet(xd, is.sparse, ix, jx, y, weights, offset, alpha, nobs, : one
## multinomial or binomial class has fewer than 8 observations; dangerous ground
```

```
# define the number of elements in class 1
threshold <- seq(0, 1, length.out = 1000)
accuracy <- c()
for (t in threshold){
  tot <- 0
  for (i in 1:(length(preds))){
    if ((groups[i] == "Denmark" && preds[i] < (1 - t)) || (groups[i] == "England" && preds[i] > (t))){
      tot <- tot + 1
    }
  }
  prop <- tot/length(preds)
  accuracy <- c(accuracy, prop)
}  
DE31_best_geo <- accuracy
```

# geo accuracy D-E dataset:

```
ggplot() + 
  geom_line(aes(threshold, DE11_best_geo), color = "yellow1") +
  geom_line(aes(threshold, DE15_best_geo), color = "gold1") +
  geom_line(aes(threshold, DE_best_geo), color = "orange2") +
  geom_line(aes(threshold, DE25_best_geo), color = "red2") +
  geom_line(aes(threshold, DE31_best_geo), color = "red4") +
  theme_bw() +
  ylim(0, 1)
```

# Random forest - Logit comparison

Load libraries:

```
library(glmnet)
#Select type of regularization (0 = ridge, 1 = lasso, 0<a<1 = elastic net):
a = 1
library(randomForest)
```

```
## Warning: package 'randomForest' was built under R version 4.1.2
```

```
## randomForest 4.7-1.1
```

```
## Type rfNews() to see new features/changes/bug fixes.
```

```
## 
## Attaching package: 'randomForest'
```

```
## The following object is masked from 'package:ggplot2':
## 
##     margin
```

```
## The following object is masked from 'package:dplyr':
## 
##     combine
```

Read BP data into R:

```
BP_mash <- read.csv("kmers_similarity_matrices/BP_similarity_matrix.csv")
# Add row names
rownames(BP_mash) <- names(BP_mash)
# Transform to matrix object for plotting
BP_mash_mat <- as.matrix(BP_mash)
# Keep only reference samples
BP_mash_mat <- BP_mash_mat[6:43, 6:43]
```

Read DE data into R: (only samples with >1 million reads)

```
# import data
DE_mash <- read.csv("kmers_similarity_matrices/DE_similarity_matrix.csv")
# Add row names
rownames(DE_mash) <- names(DE_mash)
# Transform to matrix object for plotting
DE_mash_mat <- as.matrix(DE_mash)
```

Read data into R: (all the samples)

```
# import data
DE_all_mash <- read.csv("kmers_similarity_matrices/DE_all_similarity_matrix.csv") 
# Add row names
rownames(DE_all_mash) <- names(DE_all_mash)
# Transform to matrix object for plotting
DE_all_mash_mat <- as.matrix(DE_all_mash)
```

BP dataset MDS

```
library(ggfortify)
BP_pca_mat <- prcomp(BP_mash_mat, scale. = TRUE)
```

DE - only high-depth samples

```
library(ggfortify)
DE_pca_mat <- prcomp(DE_mash_mat, scale. = TRUE)
```

DE - only high-depth samples

```
library(ggfortify)
DE_all_pca_mat <- prcomp(DE_all_mash_mat, scale. = TRUE)
```

Get training dataset:

B-P dataset:

```
# get the PCs dataframe
BP_train <- as.data.frame(BP_pca_mat$x)
# remove the last PC which explain 0% of the variance
BP_train <- BP_train[,1:37]
```

D-E dataset (high-depth samples):

```
DE_train <- as.data.frame(DE_pca_mat$x)
```

D-E dataset (all samples):

```
DE_all_train <- as.data.frame(DE_all_pca_mat$x)
```

# Logit model - BP dataset

Geography variable:

```
groups<- as.factor(c("Brazil", "Brazil", "Brazil", "Brazil", "Brazil", "Brazil", "Brazil", "Brazil", "Brazil", "Brazil", "Brazil", "Brazil", "Brazil", "Brazil", "Brazil", "Brazil", "Brazil", "Brazil", "Brazil", "Brazil", "Brazil", "Brazil", "Brazil", "RapaNui", "RapaNui", "RapaNui", "RapaNui", "RapaNui", "RapaNui", "RapaNui", "RapaNui", "RapaNui", "RapaNui", "RapaNui", "RapaNui", "RapaNui", "RapaNui", "RapaNui"))

n <- nrow(BP_train)
# placeholder for storing the i-th prediction
preds <- rep(NA, n)
for(i in 1:n) {
    dataf.BP_train <- BP_train[-i, ]
    dataf.test <- BP_train[i, ]
    groups_tmp <- groups[-i]
    set.seed(123) 
    cv.lasso <- cv.glmnet(as.matrix(dataf.BP_train), groups_tmp, alpha = a, family = "binomial")
    glmnet.fit <- glmnet(as.matrix(dataf.BP_train), groups_tmp, alpha = a, family = "binomial", lambda = cv.lasso$lambda.min)
    preds[i] <- predict(glmnet.fit, as.matrix(dataf.test), type="response")
}

threshold <- seq(0, 1, length.out = 1000)
accuracy <- c()
accuracyA <- c()
errorA <- c()
accuracyB  <- c()
errorB <- c()
for (t in threshold){
  tot <- 0
  totA <- 0
  totAf <- 0
  totB <- 0
  totBf <- 0
  for (i in 1:(length(preds))){
    if ((groups[i] == "Brazil" && preds[i] < (1 - t)) || (groups[i] == "RapaNui" && preds[i] > (t))){
      tot <- tot + 1
    }
    if ((groups[i] == "Brazil" && preds[i] < (1 - t))){
      totA <- totA + 1
    } else if ((groups[i] == "RapaNui" && preds[i] < (1 - t))){
      totAf <- totAf + 1
    }
    if ((groups[i] == "RapaNui" && preds[i] > (1 - t))){
      totB <- totB + 1
    } else if ((groups[i] == "Brazil" && preds[i] > (1 - t))){
      totBf <- totBf + 1
    }
  }
  prop <- tot/length(preds)
  propA <- totA/length(which((groups == "Brazil")))
  propAf <- totAf/length(which((groups == "RapaNui")))
  propB <- totB/length(which((groups == "RapaNui")))
  propBf <- totBf/length(which((groups == "Brazil")))
  accuracy <- c(accuracy, prop)
  accuracyA  <- c(accuracyA, propA)
  errorA <- c(errorA, propAf)
  accuracyB  <- c(accuracyB, propB)
  errorB <- c(errorB, propBf)
}  
BP_geo <- accuracy
B_geo <- accuracyA
B_geo_error <- errorA
P_geo <- accuracyB
P_geo_error <- errorB
```

Extraction protocol:

```
groups<- as.factor(c("Allentoft", "Allentoft", "Allentoft", "Allentoft", "Allentoft", "Allentoft", "Allentoft", "Allentoft", "Allentoft", "Allentoft", "Allentoft", "Allentoft", "Allentoft", "Allentoft", "Allentoft", "Allentoft", "Allentoft", "Allentoft", "Allentoft", "Allentoft", "Allentoft", "Allentoft", "Allentoft", "Allentoft", "Allentoft", "Allentoft", "Allentoft", "Damgaard", "Damgaard", "Damgaard", "Damgaard", "Damgaard", "Damgaard", "Damgaard", "Damgaard", "Damgaard", "Damgaard", "Damgaard"))

n <- nrow(BP_train)
# placeholder for storing the i-th prediction
preds <- rep(NA, n)
for(i in 1:n) {
    dataf.BP_train <- BP_train[-i, ]
    dataf.test <- BP_train[i, ]
    groups_tmp <- groups[-i]
    set.seed(123) 
    cv.lasso <- cv.glmnet(as.matrix(dataf.BP_train), groups_tmp, alpha = a, family = "binomial")
    glmnet.fit <- glmnet(as.matrix(dataf.BP_train), groups_tmp, alpha = a, family = "binomial", lambda = cv.lasso$lambda.min)
    preds[i] <- predict(glmnet.fit, as.matrix(dataf.test), type="response")
}
```

```
## Warning in lognet(xd, is.sparse, ix, jx, y, weights, offset, alpha, nobs, : one
## multinomial or binomial class has fewer than 8 observations; dangerous ground

## Warning in lognet(xd, is.sparse, ix, jx, y, weights, offset, alpha, nobs, : one
## multinomial or binomial class has fewer than 8 observations; dangerous ground

## Warning in lognet(xd, is.sparse, ix, jx, y, weights, offset, alpha, nobs, : one
## multinomial or binomial class has fewer than 8 observations; dangerous ground

## Warning in lognet(xd, is.sparse, ix, jx, y, weights, offset, alpha, nobs, : one
## multinomial or binomial class has fewer than 8 observations; dangerous ground

## Warning in lognet(xd, is.sparse, ix, jx, y, weights, offset, alpha, nobs, : one
## multinomial or binomial class has fewer than 8 observations; dangerous ground

## Warning in lognet(xd, is.sparse, ix, jx, y, weights, offset, alpha, nobs, : one
## multinomial or binomial class has fewer than 8 observations; dangerous ground

## Warning in lognet(xd, is.sparse, ix, jx, y, weights, offset, alpha, nobs, : one
## multinomial or binomial class has fewer than 8 observations; dangerous ground

## Warning in lognet(xd, is.sparse, ix, jx, y, weights, offset, alpha, nobs, : one
## multinomial or binomial class has fewer than 8 observations; dangerous ground

## Warning in lognet(xd, is.sparse, ix, jx, y, weights, offset, alpha, nobs, : one
## multinomial or binomial class has fewer than 8 observations; dangerous ground

## Warning in lognet(xd, is.sparse, ix, jx, y, weights, offset, alpha, nobs, : one
## multinomial or binomial class has fewer than 8 observations; dangerous ground

## Warning in lognet(xd, is.sparse, ix, jx, y, weights, offset, alpha, nobs, : one
## multinomial or binomial class has fewer than 8 observations; dangerous ground
```

```
threshold <- seq(0, 1, length.out = 1000)
accuracy <- c()
accuracyA <- c()
errorA <- c()
accuracyB  <- c()
errorB <- c()
for (t in threshold){
  tot <- 0
  totA <- 0
  totAf <- 0
  totB <- 0
  totBf <- 0
  for (i in 1:(length(preds))){
    if ((groups[i] == "Allentoft" && preds[i] < (1 - t)) || (groups[i] == "Damgaard" && preds[i] > (t))){
      tot <- tot + 1
    }
    if ((groups[i] == "Allentoft" && preds[i] < (1 - t))){
      totA <- totA + 1
    } else if ((groups[i] == "Damgaard" && preds[i] < (1 - t))){
      totAf <- totAf + 1
    }
    if ((groups[i] == "Damgaard" && preds[i] > (1 - t))){
      totB <- totB + 1
    } else if ((groups[i] == "Allentoft" && preds[i] > (1 - t))){
      totBf <- totBf + 1
    }
  }
  prop <- tot/length(preds)
  propA <- totA/length(which((groups == "Allentoft")))
  propAf <- totAf/length(which((groups == "Damgaard")))
  propB <- totB/length(which((groups == "Damgaard")))
  propBf <- totBf/length(which((groups == "Allentoft")))
  accuracy <- c(accuracy, prop)
  accuracyA  <- c(accuracyA, propA)
  errorA <- c(errorA, propAf)
  accuracyB  <- c(accuracyB, propB)
  errorB <- c(errorB, propBf)
}  
BP_extraction_protocol <- accuracy
B_extraction_protocol <- accuracyA
B_extraction_protocol_error <- errorA
P_extraction_protocol <- accuracyB
P_extraction_protocol_error <- errorB
```

Sample type:

```
groups<- as.factor(c("Tooth", "Tooth", "Tooth", "Bone", "Tooth", "Tooth", "Tooth", "Tooth", "Tooth", "Tooth", "Tooth", "Tooth", "Tooth", "Tooth", "Tooth", "Tooth", "Tooth", "Tooth", "Tooth", "Tooth", "Tooth", "Tooth", "Tooth", "Tooth", "Tooth", "Tooth", "Tooth", "Bone", "Bone", "Bone", "Bone", "Bone", "Bone", "Bone", "Bone", "Bone", "Bone", "Bone"))

n <- nrow(BP_train)
# placeholder for storing the i-th prediction
preds <- rep(NA, n)
for(i in 1:n) {
    dataf.BP_train <- BP_train[-i, ]
    dataf.test <- BP_train[i, ]
    groups_tmp <- groups[-i]
    set.seed(123) 
    cv.lasso <- cv.glmnet(as.matrix(dataf.BP_train), groups_tmp, alpha = a, family = "binomial")
    glmnet.fit <- glmnet(as.matrix(dataf.BP_train), groups_tmp, alpha = a, family = "binomial", lambda = cv.lasso$lambda.min)
    preds[i] <- predict(glmnet.fit, as.matrix(dataf.test), type="response")
}

threshold <- seq(0, 1, length.out = 1000)
accuracy <- c()
accuracyA <- c()
errorA <- c()
accuracyB  <- c()
errorB <- c()
for (t in threshold){
  tot <- 0
  totA <- 0
  totAf <- 0
  totB <- 0
  totBf <- 0
  for (i in 1:(length(preds))){
    if ((groups[i] == "Bone" && preds[i] < (1 - t)) || (groups[i] == "Tooth" && preds[i] > (t))){
      tot <- tot + 1
    }
    if ((groups[i] == "Bone" && preds[i] < (1 - t))){
      totA <- totA + 1
    } else if ((groups[i] == "Tooth" && preds[i] < (1 - t))){
      totAf <- totAf + 1
    }
    if ((groups[i] == "Tooth" && preds[i] > (1 - t))){
      totB <- totB + 1
    } else if ((groups[i] == "Bone" && preds[i] > (1 - t))){
      totBf <- totBf + 1
    }
  }
  prop <- tot/length(preds)
  propA <- totA/length(which((groups == "Bone")))
  propAf <- totAf/length(which((groups == "Tooth")))
  propB <- totB/length(which((groups == "Tooth")))
  propBf <- totBf/length(which((groups == "Bone")))
  accuracy <- c(accuracy, prop)
  accuracyA  <- c(accuracyA, propA)
  errorA <- c(errorA, propAf)
  accuracyB  <- c(accuracyB, propB)
  errorB <- c(errorB, propBf)
}  
BP_sample_type <- accuracy
B_sample_type <- accuracyA
B_sample_type_error <- errorA
P_sample_type <- accuracyB
P_sample_type_error <- errorB
```

# Random Forest model - BP dataset

Geography variable:

```
groups<- as.factor(c("Brazil", "Brazil", "Brazil", "Brazil", "Brazil", "Brazil", "Brazil", "Brazil", "Brazil", "Brazil", "Brazil", "Brazil", "Brazil", "Brazil", "Brazil", "Brazil", "Brazil", "Brazil", "Brazil", "Brazil", "Brazil", "Brazil", "Brazil", "RapaNui", "RapaNui", "RapaNui", "RapaNui", "RapaNui", "RapaNui", "RapaNui", "RapaNui", "RapaNui", "RapaNui", "RapaNui", "RapaNui", "RapaNui", "RapaNui", "RapaNui"))

n <- nrow(BP_train)
# placeholder for storing the i-th prediction
preds <- rep(NA, n)
for(i in 1:n) {
    dataf.BP_train <- BP_train[-i, ]
    dataf.test <- BP_train[i, ]
    groups_tmp <- groups[-i]
    set.seed(123) 
    rf.fit <- randomForest(formula = groups_tmp ~ ., data = as.matrix(dataf.BP_train))
    preds[i] <- as.numeric(predict(rf.fit, as.matrix(dataf.test), type = 'prob'))
}
```

```
## Warning in preds[i] <- as.numeric(predict(rf.fit, as.matrix(dataf.test), :
## number of items to replace is not a multiple of replacement length

## Warning in preds[i] <- as.numeric(predict(rf.fit, as.matrix(dataf.test), :
## number of items to replace is not a multiple of replacement length

## Warning in preds[i] <- as.numeric(predict(rf.fit, as.matrix(dataf.test), :
## number of items to replace is not a multiple of replacement length

## Warning in preds[i] <- as.numeric(predict(rf.fit, as.matrix(dataf.test), :
## number of items to replace is not a multiple of replacement length

## Warning in preds[i] <- as.numeric(predict(rf.fit, as.matrix(dataf.test), :
## number of items to replace is not a multiple of replacement length

## Warning in preds[i] <- as.numeric(predict(rf.fit, as.matrix(dataf.test), :
## number of items to replace is not a multiple of replacement length

## Warning in preds[i] <- as.numeric(predict(rf.fit, as.matrix(dataf.test), :
## number of items to replace is not a multiple of replacement length

## Warning in preds[i] <- as.numeric(predict(rf.fit, as.matrix(dataf.test), :
## number of items to replace is not a multiple of replacement length

## Warning in preds[i] <- as.numeric(predict(rf.fit, as.matrix(dataf.test), :
## number of items to replace is not a multiple of replacement length

## Warning in preds[i] <- as.numeric(predict(rf.fit, as.matrix(dataf.test), :
## number of items to replace is not a multiple of replacement length

## Warning in preds[i] <- as.numeric(predict(rf.fit, as.matrix(dataf.test), :
## number of items to replace is not a multiple of replacement length

## Warning in preds[i] <- as.numeric(predict(rf.fit, as.matrix(dataf.test), :
## number of items to replace is not a multiple of replacement length

## Warning in preds[i] <- as.numeric(predict(rf.fit, as.matrix(dataf.test), :
## number of items to replace is not a multiple of replacement length

## Warning in preds[i] <- as.numeric(predict(rf.fit, as.matrix(dataf.test), :
## number of items to replace is not a multiple of replacement length

## Warning in preds[i] <- as.numeric(predict(rf.fit, as.matrix(dataf.test), :
## number of items to replace is not a multiple of replacement length

## Warning in preds[i] <- as.numeric(predict(rf.fit, as.matrix(dataf.test), :
## number of items to replace is not a multiple of replacement length

## Warning in preds[i] <- as.numeric(predict(rf.fit, as.matrix(dataf.test), :
## number of items to replace is not a multiple of replacement length

## Warning in preds[i] <- as.numeric(predict(rf.fit, as.matrix(dataf.test), :
## number of items to replace is not a multiple of replacement length

## Warning in preds[i] <- as.numeric(predict(rf.fit, as.matrix(dataf.test), :
## number of items to replace is not a multiple of replacement length

## Warning in preds[i] <- as.numeric(predict(rf.fit, as.matrix(dataf.test), :
## number of items to replace is not a multiple of replacement length

## Warning in preds[i] <- as.numeric(predict(rf.fit, as.matrix(dataf.test), :
## number of items to replace is not a multiple of replacement length

## Warning in preds[i] <- as.numeric(predict(rf.fit, as.matrix(dataf.test), :
## number of items to replace is not a multiple of replacement length

## Warning in preds[i] <- as.numeric(predict(rf.fit, as.matrix(dataf.test), :
## number of items to replace is not a multiple of replacement length

## Warning in preds[i] <- as.numeric(predict(rf.fit, as.matrix(dataf.test), :
## number of items to replace is not a multiple of replacement length

## Warning in preds[i] <- as.numeric(predict(rf.fit, as.matrix(dataf.test), :
## number of items to replace is not a multiple of replacement length

## Warning in preds[i] <- as.numeric(predict(rf.fit, as.matrix(dataf.test), :
## number of items to replace is not a multiple of replacement length

## Warning in preds[i] <- as.numeric(predict(rf.fit, as.matrix(dataf.test), :
## number of items to replace is not a multiple of replacement length

## Warning in preds[i] <- as.numeric(predict(rf.fit, as.matrix(dataf.test), :
## number of items to replace is not a multiple of replacement length

## Warning in preds[i] <- as.numeric(predict(rf.fit, as.matrix(dataf.test), :
## number of items to replace is not a multiple of replacement length

## Warning in preds[i] <- as.numeric(predict(rf.fit, as.matrix(dataf.test), :
## number of items to replace is not a multiple of replacement length

## Warning in preds[i] <- as.numeric(predict(rf.fit, as.matrix(dataf.test), :
## number of items to replace is not a multiple of replacement length

## Warning in preds[i] <- as.numeric(predict(rf.fit, as.matrix(dataf.test), :
## number of items to replace is not a multiple of replacement length

## Warning in preds[i] <- as.numeric(predict(rf.fit, as.matrix(dataf.test), :
## number of items to replace is not a multiple of replacement length

## Warning in preds[i] <- as.numeric(predict(rf.fit, as.matrix(dataf.test), :
## number of items to replace is not a multiple of replacement length

## Warning in preds[i] <- as.numeric(predict(rf.fit, as.matrix(dataf.test), :
## number of items to replace is not a multiple of replacement length

## Warning in preds[i] <- as.numeric(predict(rf.fit, as.matrix(dataf.test), :
## number of items to replace is not a multiple of replacement length

## Warning in preds[i] <- as.numeric(predict(rf.fit, as.matrix(dataf.test), :
## number of items to replace is not a multiple of replacement length

## Warning in preds[i] <- as.numeric(predict(rf.fit, as.matrix(dataf.test), :
## number of items to replace is not a multiple of replacement length
```

```
threshold <- seq(0, 1, length.out = 1000)
accuracy <- c()
accuracyA <- c()
errorA <- c()
accuracyB  <- c()
errorB <- c()
for (t in threshold){
  tot <- 0
  totA <- 0
  totAf <- 0
  totB <- 0
  totBf <- 0
  for (i in 1:(length(preds))){
    if ((groups[i] == "Brazil" && preds[i] > (t)) || (groups[i] == "RapaNui" && preds[i] < (1 - t))){
      tot <- tot + 1
    }
    if ((groups[i] == "Brazil" && preds[i] > (t))){
      totA <- totA + 1
    } else if ((groups[i] == "RapaNui" && preds[i] > (t))){
      totAf <- totAf + 1
    }
    if ((groups[i] == "RapaNui" && preds[i] < (t))){
      totB <- totB + 1
    } else if ((groups[i] == "Brazil" && preds[i] < (t))){
      totBf <- totBf + 1
    }
  }
  prop <- tot/length(preds)
  propA <- totA/length(which((groups == "Brazil")))
  propAf <- totAf/length(which((groups == "RapaNui")))
  propB <- totB/length(which((groups == "RapaNui")))
  propBf <- totBf/length(which((groups == "Brazil")))
  accuracy <- c(accuracy, prop)
  accuracyA  <- c(accuracyA, propA)
  errorA <- c(errorA, propAf)
  accuracyB  <- c(accuracyB, propB)
  errorB <- c(errorB, propBf)
}  
BP_geo_rf <- accuracy
B_geo_rf <- accuracyA
B_geo_rf_error <- errorA
P_geo_rf <- accuracyB
P_geo_rf_error <- errorB
```

Extraction protocol:

```
groups<- as.factor(c("Allentoft", "Allentoft", "Allentoft", "Allentoft", "Allentoft", "Allentoft", "Allentoft", "Allentoft", "Allentoft", "Allentoft", "Allentoft", "Allentoft", "Allentoft", "Allentoft", "Allentoft", "Allentoft", "Allentoft", "Allentoft", "Allentoft", "Allentoft", "Allentoft", "Allentoft", "Allentoft", "Allentoft", "Allentoft", "Allentoft", "Allentoft", "Damgaard", "Damgaard", "Damgaard", "Damgaard", "Damgaard", "Damgaard", "Damgaard", "Damgaard", "Damgaard", "Damgaard", "Damgaard"))

n <- nrow(BP_train)
# placeholder for storing the i-th prediction
preds <- rep(NA, n)
for(i in 1:n) {
    dataf.BP_train <- BP_train[-i, ]
    dataf.test <- BP_train[i, ]
    groups_tmp <- groups[-i]
    set.seed(123) 
    rf.fit <- randomForest(formula = groups_tmp ~ ., data = as.matrix(dataf.BP_train))
    preds[i] <- as.numeric(predict(rf.fit, as.matrix(dataf.test), type = 'prob'))
}
```

```
## Warning in preds[i] <- as.numeric(predict(rf.fit, as.matrix(dataf.test), :
## number of items to replace is not a multiple of replacement length

## Warning in preds[i] <- as.numeric(predict(rf.fit, as.matrix(dataf.test), :
## number of items to replace is not a multiple of replacement length

## Warning in preds[i] <- as.numeric(predict(rf.fit, as.matrix(dataf.test), :
## number of items to replace is not a multiple of replacement length

## Warning in preds[i] <- as.numeric(predict(rf.fit, as.matrix(dataf.test), :
## number of items to replace is not a multiple of replacement length

## Warning in preds[i] <- as.numeric(predict(rf.fit, as.matrix(dataf.test), :
## number of items to replace is not a multiple of replacement length

## Warning in preds[i] <- as.numeric(predict(rf.fit, as.matrix(dataf.test), :
## number of items to replace is not a multiple of replacement length

## Warning in preds[i] <- as.numeric(predict(rf.fit, as.matrix(dataf.test), :
## number of items to replace is not a multiple of replacement length

## Warning in preds[i] <- as.numeric(predict(rf.fit, as.matrix(dataf.test), :
## number of items to replace is not a multiple of replacement length

## Warning in preds[i] <- as.numeric(predict(rf.fit, as.matrix(dataf.test), :
## number of items to replace is not a multiple of replacement length

## Warning in preds[i] <- as.numeric(predict(rf.fit, as.matrix(dataf.test), :
## number of items to replace is not a multiple of replacement length

## Warning in preds[i] <- as.numeric(predict(rf.fit, as.matrix(dataf.test), :
## number of items to replace is not a multiple of replacement length

## Warning in preds[i] <- as.numeric(predict(rf.fit, as.matrix(dataf.test), :
## number of items to replace is not a multiple of replacement length

## Warning in preds[i] <- as.numeric(predict(rf.fit, as.matrix(dataf.test), :
## number of items to replace is not a multiple of replacement length

## Warning in preds[i] <- as.numeric(predict(rf.fit, as.matrix(dataf.test), :
## number of items to replace is not a multiple of replacement length

## Warning in preds[i] <- as.numeric(predict(rf.fit, as.matrix(dataf.test), :
## number of items to replace is not a multiple of replacement length

## Warning in preds[i] <- as.numeric(predict(rf.fit, as.matrix(dataf.test), :
## number of items to replace is not a multiple of replacement length

## Warning in preds[i] <- as.numeric(predict(rf.fit, as.matrix(dataf.test), :
## number of items to replace is not a multiple of replacement length

## Warning in preds[i] <- as.numeric(predict(rf.fit, as.matrix(dataf.test), :
## number of items to replace is not a multiple of replacement length

## Warning in preds[i] <- as.numeric(predict(rf.fit, as.matrix(dataf.test), :
## number of items to replace is not a multiple of replacement length

## Warning in preds[i] <- as.numeric(predict(rf.fit, as.matrix(dataf.test), :
## number of items to replace is not a multiple of replacement length

## Warning in preds[i] <- as.numeric(predict(rf.fit, as.matrix(dataf.test), :
## number of items to replace is not a multiple of replacement length

## Warning in preds[i] <- as.numeric(predict(rf.fit, as.matrix(dataf.test), :
## number of items to replace is not a multiple of replacement length

## Warning in preds[i] <- as.numeric(predict(rf.fit, as.matrix(dataf.test), :
## number of items to replace is not a multiple of replacement length

## Warning in preds[i] <- as.numeric(predict(rf.fit, as.matrix(dataf.test), :
## number of items to replace is not a multiple of replacement length

## Warning in preds[i] <- as.numeric(predict(rf.fit, as.matrix(dataf.test), :
## number of items to replace is not a multiple of replacement length

## Warning in preds[i] <- as.numeric(predict(rf.fit, as.matrix(dataf.test), :
## number of items to replace is not a multiple of replacement length

## Warning in preds[i] <- as.numeric(predict(rf.fit, as.matrix(dataf.test), :
## number of items to replace is not a multiple of replacement length

## Warning in preds[i] <- as.numeric(predict(rf.fit, as.matrix(dataf.test), :
## number of items to replace is not a multiple of replacement length

## Warning in preds[i] <- as.numeric(predict(rf.fit, as.matrix(dataf.test), :
## number of items to replace is not a multiple of replacement length

## Warning in preds[i] <- as.numeric(predict(rf.fit, as.matrix(dataf.test), :
## number of items to replace is not a multiple of replacement length

## Warning in preds[i] <- as.numeric(predict(rf.fit, as.matrix(dataf.test), :
## number of items to replace is not a multiple of replacement length

## Warning in preds[i] <- as.numeric(predict(rf.fit, as.matrix(dataf.test), :
## number of items to replace is not a multiple of replacement length

## Warning in preds[i] <- as.numeric(predict(rf.fit, as.matrix(dataf.test), :
## number of items to replace is not a multiple of replacement length

## Warning in preds[i] <- as.numeric(predict(rf.fit, as.matrix(dataf.test), :
## number of items to replace is not a multiple of replacement length

## Warning in preds[i] <- as.numeric(predict(rf.fit, as.matrix(dataf.test), :
## number of items to replace is not a multiple of replacement length

## Warning in preds[i] <- as.numeric(predict(rf.fit, as.matrix(dataf.test), :
## number of items to replace is not a multiple of replacement length

## Warning in preds[i] <- as.numeric(predict(rf.fit, as.matrix(dataf.test), :
## number of items to replace is not a multiple of replacement length

## Warning in preds[i] <- as.numeric(predict(rf.fit, as.matrix(dataf.test), :
## number of items to replace is not a multiple of replacement length
```

```
threshold <- seq(0, 1, length.out = 1000)
accuracy <- c()
accuracyA <- c()
errorA <- c()
accuracyB  <- c()
errorB <- c()
for (t in threshold){
  tot <- 0
  totA <- 0
  totAf <- 0
  totB <- 0
  totBf <- 0
  for (i in 1:(length(preds))){
    if ((groups[i] == "Allentoft" && preds[i] > (t)) || (groups[i] == "Damgaard" && preds[i] < (1 - t))){
      tot <- tot + 1
    }
    if ((groups[i] == "Allentoft" && preds[i] > (t))){
      totA <- totA + 1
    } else if ((groups[i] == "Damgaard" && preds[i] > (t))){
      totAf <- totAf + 1
    }
    if ((groups[i] == "Damgaard" && preds[i] < (t))){
      totB <- totB + 1
    } else if ((groups[i] == "Allentoft" && preds[i] < (t))){
      totBf <- totBf + 1
    }
  }
  prop <- tot/length(preds)
  propA <- totA/length(which((groups == "Allentoft")))
  propAf <- totAf/length(which((groups == "Damgaard")))
  propB <- totB/length(which((groups == "Damgaard")))
  propBf <- totBf/length(which((groups == "Allentoft")))
  accuracy <- c(accuracy, prop)
  accuracyA  <- c(accuracyA, propA)
  errorA <- c(errorA, propAf)
  accuracyB  <- c(accuracyB, propB)
  errorB <- c(errorB, propBf)
}  
BP_extraction_protocol_rf <- accuracy
B_extraction_protocol_rf <- accuracyA
B_extraction_protocol_rf_error <- errorA
P_extraction_protocol_rf <- accuracyB
P_extraction_protocol_rf_error <- errorB
```

Sample Type:

```
groups<- as.factor(c("Tooth", "Tooth", "Tooth", "Bone", "Tooth", "Tooth", "Tooth", "Tooth", "Tooth", "Tooth", "Tooth", "Tooth", "Tooth", "Tooth", "Tooth", "Tooth", "Tooth", "Tooth", "Tooth", "Tooth", "Tooth", "Tooth", "Tooth", "Tooth", "Tooth", "Tooth", "Tooth", "Bone", "Bone", "Bone", "Bone", "Bone", "Bone", "Bone", "Bone", "Bone", "Bone", "Bone"))

n <- nrow(BP_train)
# placeholder for storing the i-th prediction
preds <- rep(NA, n)
for(i in 1:n) {
    dataf.BP_train <- BP_train[-i, ]
    dataf.test <- BP_train[i, ]
    groups_tmp <- groups[-i]
    set.seed(123) 
    rf.fit <- randomForest(formula = groups_tmp ~ ., data = as.matrix(dataf.BP_train))
    preds[i] <- as.numeric(predict(rf.fit, as.matrix(dataf.test), type = 'prob'))
}
```

```
## Warning in preds[i] <- as.numeric(predict(rf.fit, as.matrix(dataf.test), :
## number of items to replace is not a multiple of replacement length

## Warning in preds[i] <- as.numeric(predict(rf.fit, as.matrix(dataf.test), :
## number of items to replace is not a multiple of replacement length

## Warning in preds[i] <- as.numeric(predict(rf.fit, as.matrix(dataf.test), :
## number of items to replace is not a multiple of replacement length

## Warning in preds[i] <- as.numeric(predict(rf.fit, as.matrix(dataf.test), :
## number of items to replace is not a multiple of replacement length

## Warning in preds[i] <- as.numeric(predict(rf.fit, as.matrix(dataf.test), :
## number of items to replace is not a multiple of replacement length

## Warning in preds[i] <- as.numeric(predict(rf.fit, as.matrix(dataf.test), :
## number of items to replace is not a multiple of replacement length

## Warning in preds[i] <- as.numeric(predict(rf.fit, as.matrix(dataf.test), :
## number of items to replace is not a multiple of replacement length

## Warning in preds[i] <- as.numeric(predict(rf.fit, as.matrix(dataf.test), :
## number of items to replace is not a multiple of replacement length

## Warning in preds[i] <- as.numeric(predict(rf.fit, as.matrix(dataf.test), :
## number of items to replace is not a multiple of replacement length

## Warning in preds[i] <- as.numeric(predict(rf.fit, as.matrix(dataf.test), :
## number of items to replace is not a multiple of replacement length

## Warning in preds[i] <- as.numeric(predict(rf.fit, as.matrix(dataf.test), :
## number of items to replace is not a multiple of replacement length

## Warning in preds[i] <- as.numeric(predict(rf.fit, as.matrix(dataf.test), :
## number of items to replace is not a multiple of replacement length

## Warning in preds[i] <- as.numeric(predict(rf.fit, as.matrix(dataf.test), :
## number of items to replace is not a multiple of replacement length

## Warning in preds[i] <- as.numeric(predict(rf.fit, as.matrix(dataf.test), :
## number of items to replace is not a multiple of replacement length

## Warning in preds[i] <- as.numeric(predict(rf.fit, as.matrix(dataf.test), :
## number of items to replace is not a multiple of replacement length

## Warning in preds[i] <- as.numeric(predict(rf.fit, as.matrix(dataf.test), :
## number of items to replace is not a multiple of replacement length

## Warning in preds[i] <- as.numeric(predict(rf.fit, as.matrix(dataf.test), :
## number of items to replace is not a multiple of replacement length

## Warning in preds[i] <- as.numeric(predict(rf.fit, as.matrix(dataf.test), :
## number of items to replace is not a multiple of replacement length

## Warning in preds[i] <- as.numeric(predict(rf.fit, as.matrix(dataf.test), :
## number of items to replace is not a multiple of replacement length

## Warning in preds[i] <- as.numeric(predict(rf.fit, as.matrix(dataf.test), :
## number of items to replace is not a multiple of replacement length

## Warning in preds[i] <- as.numeric(predict(rf.fit, as.matrix(dataf.test), :
## number of items to replace is not a multiple of replacement length

## Warning in preds[i] <- as.numeric(predict(rf.fit, as.matrix(dataf.test), :
## number of items to replace is not a multiple of replacement length

## Warning in preds[i] <- as.numeric(predict(rf.fit, as.matrix(dataf.test), :
## number of items to replace is not a multiple of replacement length

## Warning in preds[i] <- as.numeric(predict(rf.fit, as.matrix(dataf.test), :
## number of items to replace is not a multiple of replacement length

## Warning in preds[i] <- as.numeric(predict(rf.fit, as.matrix(dataf.test), :
## number of items to replace is not a multiple of replacement length

## Warning in preds[i] <- as.numeric(predict(rf.fit, as.matrix(dataf.test), :
## number of items to replace is not a multiple of replacement length

## Warning in preds[i] <- as.numeric(predict(rf.fit, as.matrix(dataf.test), :
## number of items to replace is not a multiple of replacement length

## Warning in preds[i] <- as.numeric(predict(rf.fit, as.matrix(dataf.test), :
## number of items to replace is not a multiple of replacement length

## Warning in preds[i] <- as.numeric(predict(rf.fit, as.matrix(dataf.test), :
## number of items to replace is not a multiple of replacement length

## Warning in preds[i] <- as.numeric(predict(rf.fit, as.matrix(dataf.test), :
## number of items to replace is not a multiple of replacement length

## Warning in preds[i] <- as.numeric(predict(rf.fit, as.matrix(dataf.test), :
## number of items to replace is not a multiple of replacement length

## Warning in preds[i] <- as.numeric(predict(rf.fit, as.matrix(dataf.test), :
## number of items to replace is not a multiple of replacement length

## Warning in preds[i] <- as.numeric(predict(rf.fit, as.matrix(dataf.test), :
## number of items to replace is not a multiple of replacement length

## Warning in preds[i] <- as.numeric(predict(rf.fit, as.matrix(dataf.test), :
## number of items to replace is not a multiple of replacement length

## Warning in preds[i] <- as.numeric(predict(rf.fit, as.matrix(dataf.test), :
## number of items to replace is not a multiple of replacement length

## Warning in preds[i] <- as.numeric(predict(rf.fit, as.matrix(dataf.test), :
## number of items to replace is not a multiple of replacement length

## Warning in preds[i] <- as.numeric(predict(rf.fit, as.matrix(dataf.test), :
## number of items to replace is not a multiple of replacement length

## Warning in preds[i] <- as.numeric(predict(rf.fit, as.matrix(dataf.test), :
## number of items to replace is not a multiple of replacement length
```

```
threshold <- seq(0, 1, length.out = 1000)
accuracy <- c()
accuracyA <- c()
errorA <- c()
accuracyB  <- c()
errorB <- c()
for (t in threshold){
  tot <- 0
  totA <- 0
  totAf <- 0
  totB <- 0
  totBf <- 0
  for (i in 1:(length(preds))){
    if ((groups[i] == "Bone" && preds[i] > (t)) || (groups[i] == "Tooth" && preds[i] < (1 - t))){
      tot <- tot + 1
    }
    if ((groups[i] == "Bone" && preds[i] > (t))){
      totA <- totA + 1
    } else if ((groups[i] == "Tooth" && preds[i] > (t))){
      totAf <- totAf + 1
    }
    if ((groups[i] == "Tooth" && preds[i] < (t))){
      totB <- totB + 1
    } else if ((groups[i] == "Bone" && preds[i] < (t))){
      totBf <- totBf + 1
    }
  }
  prop <- tot/length(preds)
  propA <- totA/length(which((groups == "Bone")))
  propAf <- totAf/length(which((groups == "Tooth")))
  propB <- totB/length(which((groups == "Tooth")))
  propBf <- totBf/length(which((groups == "Bone")))
  accuracy <- c(accuracy, prop)
  accuracyA  <- c(accuracyA, propA)
  errorA <- c(errorA, propAf)
  accuracyB  <- c(accuracyB, propB)
  errorB <- c(errorB, propBf)
}  
BP_sample_type_rf <- accuracy
B_sample_type_rf <- accuracyA
B_sample_type_rf_error <- errorA
P_sample_type_rf <- accuracyB
P_sample_type_rf_error <- errorB
```

# Logit - DE dataset:

Geography variable - best samples:

```
groups<- as.factor(c("Denmark", "Denmark", "Denmark", "Denmark", "Denmark", "Denmark", "Denmark", "Denmark", "Denmark", "Denmark", "Denmark", "Denmark", "Denmark", "Denmark", "Denmark", "Denmark", "Denmark", "Denmark", "Denmark", "Denmark", "Denmark", "Denmark", "Denmark", "Denmark", "England", "England", "England", "England", "England", "England", "England", "England", "England", "England", "England"))

n <- nrow(DE_train)
# placeholder for storing the i-th prediction
preds <- rep(NA, n)
for(i in 1:n) {
    dataf.train <- DE_train[-i, ]
    dataf.test <- DE_train[i, ]
    groups_tmp <- groups[-i]
    set.seed(123) 
    cv.lasso <- cv.glmnet(as.matrix(dataf.train), groups_tmp, alpha = a, family = "binomial")
    glmnet.fit <- glmnet(as.matrix(dataf.train), groups_tmp, alpha = a, family = "binomial", lambda = cv.lasso$lambda.min)
    preds[i] <- predict(glmnet.fit, as.matrix(dataf.test), type="response")
}
```

```
## Warning in lognet(xd, is.sparse, ix, jx, y, weights, offset, alpha, nobs, : one
## multinomial or binomial class has fewer than 8 observations; dangerous ground

## Warning in lognet(xd, is.sparse, ix, jx, y, weights, offset, alpha, nobs, : one
## multinomial or binomial class has fewer than 8 observations; dangerous ground

## Warning in lognet(xd, is.sparse, ix, jx, y, weights, offset, alpha, nobs, : one
## multinomial or binomial class has fewer than 8 observations; dangerous ground

## Warning in lognet(xd, is.sparse, ix, jx, y, weights, offset, alpha, nobs, : one
## multinomial or binomial class has fewer than 8 observations; dangerous ground

## Warning in lognet(xd, is.sparse, ix, jx, y, weights, offset, alpha, nobs, : one
## multinomial or binomial class has fewer than 8 observations; dangerous ground

## Warning in lognet(xd, is.sparse, ix, jx, y, weights, offset, alpha, nobs, : one
## multinomial or binomial class has fewer than 8 observations; dangerous ground

## Warning in lognet(xd, is.sparse, ix, jx, y, weights, offset, alpha, nobs, : one
## multinomial or binomial class has fewer than 8 observations; dangerous ground

## Warning in lognet(xd, is.sparse, ix, jx, y, weights, offset, alpha, nobs, : one
## multinomial or binomial class has fewer than 8 observations; dangerous ground

## Warning in lognet(xd, is.sparse, ix, jx, y, weights, offset, alpha, nobs, : one
## multinomial or binomial class has fewer than 8 observations; dangerous ground

## Warning in lognet(xd, is.sparse, ix, jx, y, weights, offset, alpha, nobs, : one
## multinomial or binomial class has fewer than 8 observations; dangerous ground

## Warning in lognet(xd, is.sparse, ix, jx, y, weights, offset, alpha, nobs, : one
## multinomial or binomial class has fewer than 8 observations; dangerous ground
```

```
threshold <- seq(0, 1, length.out = 1000)
accuracy <- c()
accuracyA <- c()
errorA <- c()
accuracyB  <- c()
errorB <- c()
for (t in threshold){
  tot <- 0
  totA <- 0
  totAf <- 0
  totB <- 0
  totBf <- 0
  for (i in 1:(length(preds))){
    if ((groups[i] == "Denmark" && preds[i] < (1 - t)) || (groups[i] == "England" && preds[i] > (t))){
      tot <- tot + 1
    }
    if ((groups[i] == "Denmark" && preds[i] < (1 - t))){
      totA <- totA + 1
    } else if ((groups[i] == "England" && preds[i] < (1 - t))){
      totAf <- totAf + 1
    }
    if ((groups[i] == "England" && preds[i] > (1 - t))){
      totB <- totB + 1
    } else if ((groups[i] == "Denmark" && preds[i] > (1 - t))){
      totBf <- totBf + 1
    }
  }
  prop <- tot/length(preds)
  propA <- totA/length(which((groups == "Denmark")))
  propAf <- totAf/length(which((groups == "England")))
  propB <- totB/length(which((groups == "England")))
  propBf <- totBf/length(which((groups == "Denmark")))
  accuracy <- c(accuracy, prop)
  accuracyA  <- c(accuracyA, propA)
  errorA <- c(errorA, propAf)
  accuracyB  <- c(accuracyB, propB)
  errorB <- c(errorB, propBf)
}  
DE_best_geo <- accuracy
D_best_geo <- accuracyA
D_best_geo_error <- errorA
E_best_geo <- accuracyB
E_best_geo_error <- errorB
```

Geography variable - all samples:

```
groups <- factor(c("Denmark", "Denmark", "Denmark", "Denmark", "Denmark", "Denmark", "Denmark", "Denmark", "Denmark", "Denmark", "Denmark", "Denmark", "Denmark", "Denmark", "Denmark", "Denmark", "Denmark", "Denmark", "Denmark", "Denmark", "Denmark", "Denmark", "Denmark", "Denmark", "Denmark", "Denmark", "Denmark", "Denmark", "Denmark", "England", "England",  "England", "England", "England", "England",  "England", "England", "England", "England",  "England", "England", "England", "England",  "England", "England", "England", "England",  "England", "England", "England", "England"))

n <- nrow(DE_all_train)
# placeholder for storing the i-th prediction
preds <- rep(NA, n)
for(i in 1:n) {
    dataf.train <- DE_all_train[-i, ]
    dataf.test <- DE_all_train[i, ]
    groups_tmp <- groups[-i]
    set.seed(123) 
    cv.lasso <- cv.glmnet(as.matrix(dataf.train), groups_tmp, alpha = a, family = "binomial")
    glmnet.fit <- glmnet(as.matrix(dataf.train), groups_tmp, alpha = a, family = "binomial", lambda = cv.lasso$lambda.min)
    preds[i] <- predict(glmnet.fit, as.matrix(dataf.test), type="response")
}


threshold <- seq(0, 1, length.out = 1000)
accuracy <- c()
accuracyA <- c()
errorA <- c()
accuracyB  <- c()
errorB <- c()
for (t in threshold){
  tot <- 0
  totA <- 0
  totAf <- 0
  totB <- 0
  totBf <- 0
  for (i in 1:(length(preds))){
    if ((groups[i] == "Denmark" && preds[i] < (1 - t)) || (groups[i] == "England" && preds[i] > (t))){
      tot <- tot + 1
    }
    if ((groups[i] == "Denmark" && preds[i] < (1 - t))){
      totA <- totA + 1
    } else if ((groups[i] == "England" && preds[i] < (1 - t))){
      totAf <- totAf + 1
    }
    if ((groups[i] == "England" && preds[i] > (1 - t))){
      totB <- totB + 1
    } else if ((groups[i] == "Denmark" && preds[i] > (1 - t))){
      totBf <- totBf + 1
    }
  }
  prop <- tot/length(preds)
  propA <- totA/length(which((groups == "Denmark")))
  propAf <- totAf/length(which((groups == "England")))
  propB <- totB/length(which((groups == "England")))
  propBf <- totBf/length(which((groups == "Denmark")))
  accuracy <- c(accuracy, prop)
  accuracyA  <- c(accuracyA, propA)
  errorA <- c(errorA, propAf)
  accuracyB  <- c(accuracyB, propB)
  errorB <- c(errorB, propBf)
}  
DE_all_geo <- accuracy
D_all_geo <- accuracyA
D_all_geo_error <- errorA
E_all_geo <- accuracyB
E_all_geo_error <- errorB
```

# Random Forest - DE dataset:

Geography variable - best samples:

```
groups<- as.factor(c("Denmark", "Denmark", "Denmark", "Denmark", "Denmark", "Denmark", "Denmark", "Denmark", "Denmark", "Denmark", "Denmark", "Denmark", "Denmark", "Denmark", "Denmark", "Denmark", "Denmark", "Denmark", "Denmark", "Denmark", "Denmark", "Denmark", "Denmark", "Denmark", "England", "England", "England", "England", "England", "England", "England", "England", "England", "England", "England"))

n <- nrow(DE_train)
# placeholder for storing the i-th prediction
preds <- rep(NA, n)
for(i in 1:n) {
    dataf.train <- DE_train[-i, ]
    dataf.test <- DE_train[i, ]
    groups_tmp <- groups[-i]
    set.seed(123) 
    rf.fit <- randomForest(formula = groups_tmp ~ ., data = as.matrix(dataf.train))
    preds[i] <- as.numeric(predict(rf.fit, as.matrix(dataf.test), type = 'prob'))
}
```

```
## Warning in preds[i] <- as.numeric(predict(rf.fit, as.matrix(dataf.test), :
## number of items to replace is not a multiple of replacement length

## Warning in preds[i] <- as.numeric(predict(rf.fit, as.matrix(dataf.test), :
## number of items to replace is not a multiple of replacement length

## Warning in preds[i] <- as.numeric(predict(rf.fit, as.matrix(dataf.test), :
## number of items to replace is not a multiple of replacement length

## Warning in preds[i] <- as.numeric(predict(rf.fit, as.matrix(dataf.test), :
## number of items to replace is not a multiple of replacement length

## Warning in preds[i] <- as.numeric(predict(rf.fit, as.matrix(dataf.test), :
## number of items to replace is not a multiple of replacement length

## Warning in preds[i] <- as.numeric(predict(rf.fit, as.matrix(dataf.test), :
## number of items to replace is not a multiple of replacement length

## Warning in preds[i] <- as.numeric(predict(rf.fit, as.matrix(dataf.test), :
## number of items to replace is not a multiple of replacement length

## Warning in preds[i] <- as.numeric(predict(rf.fit, as.matrix(dataf.test), :
## number of items to replace is not a multiple of replacement length

## Warning in preds[i] <- as.numeric(predict(rf.fit, as.matrix(dataf.test), :
## number of items to replace is not a multiple of replacement length

## Warning in preds[i] <- as.numeric(predict(rf.fit, as.matrix(dataf.test), :
## number of items to replace is not a multiple of replacement length

## Warning in preds[i] <- as.numeric(predict(rf.fit, as.matrix(dataf.test), :
## number of items to replace is not a multiple of replacement length

## Warning in preds[i] <- as.numeric(predict(rf.fit, as.matrix(dataf.test), :
## number of items to replace is not a multiple of replacement length

## Warning in preds[i] <- as.numeric(predict(rf.fit, as.matrix(dataf.test), :
## number of items to replace is not a multiple of replacement length

## Warning in preds[i] <- as.numeric(predict(rf.fit, as.matrix(dataf.test), :
## number of items to replace is not a multiple of replacement length

## Warning in preds[i] <- as.numeric(predict(rf.fit, as.matrix(dataf.test), :
## number of items to replace is not a multiple of replacement length

## Warning in preds[i] <- as.numeric(predict(rf.fit, as.matrix(dataf.test), :
## number of items to replace is not a multiple of replacement length

## Warning in preds[i] <- as.numeric(predict(rf.fit, as.matrix(dataf.test), :
## number of items to replace is not a multiple of replacement length

## Warning in preds[i] <- as.numeric(predict(rf.fit, as.matrix(dataf.test), :
## number of items to replace is not a multiple of replacement length

## Warning in preds[i] <- as.numeric(predict(rf.fit, as.matrix(dataf.test), :
## number of items to replace is not a multiple of replacement length

## Warning in preds[i] <- as.numeric(predict(rf.fit, as.matrix(dataf.test), :
## number of items to replace is not a multiple of replacement length

## Warning in preds[i] <- as.numeric(predict(rf.fit, as.matrix(dataf.test), :
## number of items to replace is not a multiple of replacement length

## Warning in preds[i] <- as.numeric(predict(rf.fit, as.matrix(dataf.test), :
## number of items to replace is not a multiple of replacement length

## Warning in preds[i] <- as.numeric(predict(rf.fit, as.matrix(dataf.test), :
## number of items to replace is not a multiple of replacement length

## Warning in preds[i] <- as.numeric(predict(rf.fit, as.matrix(dataf.test), :
## number of items to replace is not a multiple of replacement length

## Warning in preds[i] <- as.numeric(predict(rf.fit, as.matrix(dataf.test), :
## number of items to replace is not a multiple of replacement length

## Warning in preds[i] <- as.numeric(predict(rf.fit, as.matrix(dataf.test), :
## number of items to replace is not a multiple of replacement length

## Warning in preds[i] <- as.numeric(predict(rf.fit, as.matrix(dataf.test), :
## number of items to replace is not a multiple of replacement length

## Warning in preds[i] <- as.numeric(predict(rf.fit, as.matrix(dataf.test), :
## number of items to replace is not a multiple of replacement length

## Warning in preds[i] <- as.numeric(predict(rf.fit, as.matrix(dataf.test), :
## number of items to replace is not a multiple of replacement length

## Warning in preds[i] <- as.numeric(predict(rf.fit, as.matrix(dataf.test), :
## number of items to replace is not a multiple of replacement length

## Warning in preds[i] <- as.numeric(predict(rf.fit, as.matrix(dataf.test), :
## number of items to replace is not a multiple of replacement length

## Warning in preds[i] <- as.numeric(predict(rf.fit, as.matrix(dataf.test), :
## number of items to replace is not a multiple of replacement length

## Warning in preds[i] <- as.numeric(predict(rf.fit, as.matrix(dataf.test), :
## number of items to replace is not a multiple of replacement length

## Warning in preds[i] <- as.numeric(predict(rf.fit, as.matrix(dataf.test), :
## number of items to replace is not a multiple of replacement length

## Warning in preds[i] <- as.numeric(predict(rf.fit, as.matrix(dataf.test), :
## number of items to replace is not a multiple of replacement length
```

```
threshold <- seq(0, 1, length.out = 1000)
accuracy <- c()
accuracyA <- c()
errorA <- c()
accuracyB  <- c()
errorB <- c()
for (t in threshold){
  tot <- 0
  totA <- 0
  totAf <- 0
  totB <- 0
  totBf <- 0
  for (i in 1:(length(preds))){
    if ((groups[i] == "Denmark" && preds[i] > (t)) || (groups[i] == "England" && preds[i] < (1 - t))){
      tot <- tot + 1
    }
    if ((groups[i] == "Denmark" && preds[i] > (t))){
      totA <- totA + 1
    } else if ((groups[i] == "England" && preds[i] > (t))){
      totAf <- totAf + 1
    }
    if ((groups[i] == "England" && preds[i] < (t))){
      totB <- totB + 1
    } else if ((groups[i] == "Denmark" && preds[i] < (t))){
      totBf <- totBf + 1
    }
  }
  prop <- tot/length(preds)
  propA <- totA/length(which((groups == "Denmark")))
  propAf <- totAf/length(which((groups == "England")))
  propB <- totB/length(which((groups == "England")))
  propBf <- totBf/length(which((groups == "Denmark")))
  accuracy <- c(accuracy, prop)
  accuracyA  <- c(accuracyA, propA)
  errorA <- c(errorA, propAf)
  accuracyB  <- c(accuracyB, propB)
  errorB <- c(errorB, propBf)
}  
DE_best_geo_rf <- accuracy
D_best_geo_rf <- accuracyA
D_best_geo_rf_error <- errorA
E_best_geo_rf <- accuracyB
E_best_geo_rf_error <- errorB
```

Geography variable - all samples:

```
groups <- factor(c("Denmark", "Denmark", "Denmark", "Denmark", "Denmark", "Denmark", "Denmark", "Denmark", "Denmark", "Denmark", "Denmark", "Denmark", "Denmark", "Denmark", "Denmark", "Denmark", "Denmark", "Denmark", "Denmark", "Denmark", "Denmark", "Denmark", "Denmark", "Denmark", "Denmark", "Denmark", "Denmark", "Denmark", "Denmark", "England", "England",  "England", "England", "England", "England",  "England", "England", "England", "England",  "England", "England", "England", "England",  "England", "England", "England", "England",  "England", "England", "England", "England"))

n <- nrow(DE_all_train)
# placeholder for storing the i-th prediction
preds <- rep(NA, n)
for(i in 1:n) {
    dataf.train <- DE_all_train[-i, ]
    dataf.test <- DE_all_train[i, ]
    groups_tmp <- groups[-i]
    set.seed(123) 
    rf.fit <- randomForest(formula = groups_tmp ~ ., data = as.matrix(dataf.train))
    preds[i] <- as.numeric(predict(rf.fit, as.matrix(dataf.test), type = 'prob'))
}
```

```
## Warning in preds[i] <- as.numeric(predict(rf.fit, as.matrix(dataf.test), :
## number of items to replace is not a multiple of replacement length

## Warning in preds[i] <- as.numeric(predict(rf.fit, as.matrix(dataf.test), :
## number of items to replace is not a multiple of replacement length

## Warning in preds[i] <- as.numeric(predict(rf.fit, as.matrix(dataf.test), :
## number of items to replace is not a multiple of replacement length

## Warning in preds[i] <- as.numeric(predict(rf.fit, as.matrix(dataf.test), :
## number of items to replace is not a multiple of replacement length

## Warning in preds[i] <- as.numeric(predict(rf.fit, as.matrix(dataf.test), :
## number of items to replace is not a multiple of replacement length

## Warning in preds[i] <- as.numeric(predict(rf.fit, as.matrix(dataf.test), :
## number of items to replace is not a multiple of replacement length

## Warning in preds[i] <- as.numeric(predict(rf.fit, as.matrix(dataf.test), :
## number of items to replace is not a multiple of replacement length

## Warning in preds[i] <- as.numeric(predict(rf.fit, as.matrix(dataf.test), :
## number of items to replace is not a multiple of replacement length

## Warning in preds[i] <- as.numeric(predict(rf.fit, as.matrix(dataf.test), :
## number of items to replace is not a multiple of replacement length

## Warning in preds[i] <- as.numeric(predict(rf.fit, as.matrix(dataf.test), :
## number of items to replace is not a multiple of replacement length

## Warning in preds[i] <- as.numeric(predict(rf.fit, as.matrix(dataf.test), :
## number of items to replace is not a multiple of replacement length

## Warning in preds[i] <- as.numeric(predict(rf.fit, as.matrix(dataf.test), :
## number of items to replace is not a multiple of replacement length

## Warning in preds[i] <- as.numeric(predict(rf.fit, as.matrix(dataf.test), :
## number of items to replace is not a multiple of replacement length

## Warning in preds[i] <- as.numeric(predict(rf.fit, as.matrix(dataf.test), :
## number of items to replace is not a multiple of replacement length

## Warning in preds[i] <- as.numeric(predict(rf.fit, as.matrix(dataf.test), :
## number of items to replace is not a multiple of replacement length

## Warning in preds[i] <- as.numeric(predict(rf.fit, as.matrix(dataf.test), :
## number of items to replace is not a multiple of replacement length

## Warning in preds[i] <- as.numeric(predict(rf.fit, as.matrix(dataf.test), :
## number of items to replace is not a multiple of replacement length

## Warning in preds[i] <- as.numeric(predict(rf.fit, as.matrix(dataf.test), :
## number of items to replace is not a multiple of replacement length

## Warning in preds[i] <- as.numeric(predict(rf.fit, as.matrix(dataf.test), :
## number of items to replace is not a multiple of replacement length

## Warning in preds[i] <- as.numeric(predict(rf.fit, as.matrix(dataf.test), :
## number of items to replace is not a multiple of replacement length

## Warning in preds[i] <- as.numeric(predict(rf.fit, as.matrix(dataf.test), :
## number of items to replace is not a multiple of replacement length

## Warning in preds[i] <- as.numeric(predict(rf.fit, as.matrix(dataf.test), :
## number of items to replace is not a multiple of replacement length

## Warning in preds[i] <- as.numeric(predict(rf.fit, as.matrix(dataf.test), :
## number of items to replace is not a multiple of replacement length

## Warning in preds[i] <- as.numeric(predict(rf.fit, as.matrix(dataf.test), :
## number of items to replace is not a multiple of replacement length

## Warning in preds[i] <- as.numeric(predict(rf.fit, as.matrix(dataf.test), :
## number of items to replace is not a multiple of replacement length

## Warning in preds[i] <- as.numeric(predict(rf.fit, as.matrix(dataf.test), :
## number of items to replace is not a multiple of replacement length

## Warning in preds[i] <- as.numeric(predict(rf.fit, as.matrix(dataf.test), :
## number of items to replace is not a multiple of replacement length

## Warning in preds[i] <- as.numeric(predict(rf.fit, as.matrix(dataf.test), :
## number of items to replace is not a multiple of replacement length

## Warning in preds[i] <- as.numeric(predict(rf.fit, as.matrix(dataf.test), :
## number of items to replace is not a multiple of replacement length

## Warning in preds[i] <- as.numeric(predict(rf.fit, as.matrix(dataf.test), :
## number of items to replace is not a multiple of replacement length

## Warning in preds[i] <- as.numeric(predict(rf.fit, as.matrix(dataf.test), :
## number of items to replace is not a multiple of replacement length

## Warning in preds[i] <- as.numeric(predict(rf.fit, as.matrix(dataf.test), :
## number of items to replace is not a multiple of replacement length

## Warning in preds[i] <- as.numeric(predict(rf.fit, as.matrix(dataf.test), :
## number of items to replace is not a multiple of replacement length

## Warning in preds[i] <- as.numeric(predict(rf.fit, as.matrix(dataf.test), :
## number of items to replace is not a multiple of replacement length

## Warning in preds[i] <- as.numeric(predict(rf.fit, as.matrix(dataf.test), :
## number of items to replace is not a multiple of replacement length

## Warning in preds[i] <- as.numeric(predict(rf.fit, as.matrix(dataf.test), :
## number of items to replace is not a multiple of replacement length

## Warning in preds[i] <- as.numeric(predict(rf.fit, as.matrix(dataf.test), :
## number of items to replace is not a multiple of replacement length

## Warning in preds[i] <- as.numeric(predict(rf.fit, as.matrix(dataf.test), :
## number of items to replace is not a multiple of replacement length

## Warning in preds[i] <- as.numeric(predict(rf.fit, as.matrix(dataf.test), :
## number of items to replace is not a multiple of replacement length

## Warning in preds[i] <- as.numeric(predict(rf.fit, as.matrix(dataf.test), :
## number of items to replace is not a multiple of replacement length

## Warning in preds[i] <- as.numeric(predict(rf.fit, as.matrix(dataf.test), :
## number of items to replace is not a multiple of replacement length

## Warning in preds[i] <- as.numeric(predict(rf.fit, as.matrix(dataf.test), :
## number of items to replace is not a multiple of replacement length

## Warning in preds[i] <- as.numeric(predict(rf.fit, as.matrix(dataf.test), :
## number of items to replace is not a multiple of replacement length

## Warning in preds[i] <- as.numeric(predict(rf.fit, as.matrix(dataf.test), :
## number of items to replace is not a multiple of replacement length

## Warning in preds[i] <- as.numeric(predict(rf.fit, as.matrix(dataf.test), :
## number of items to replace is not a multiple of replacement length

## Warning in preds[i] <- as.numeric(predict(rf.fit, as.matrix(dataf.test), :
## number of items to replace is not a multiple of replacement length

## Warning in preds[i] <- as.numeric(predict(rf.fit, as.matrix(dataf.test), :
## number of items to replace is not a multiple of replacement length

## Warning in preds[i] <- as.numeric(predict(rf.fit, as.matrix(dataf.test), :
## number of items to replace is not a multiple of replacement length

## Warning in preds[i] <- as.numeric(predict(rf.fit, as.matrix(dataf.test), :
## number of items to replace is not a multiple of replacement length

## Warning in preds[i] <- as.numeric(predict(rf.fit, as.matrix(dataf.test), :
## number of items to replace is not a multiple of replacement length

## Warning in preds[i] <- as.numeric(predict(rf.fit, as.matrix(dataf.test), :
## number of items to replace is not a multiple of replacement length
```

```
threshold <- seq(0, 1, length.out = 1000)
accuracy <- c()
accuracyA <- c()
errorA <- c()
accuracyB  <- c()
errorB <- c()
for (t in threshold){
  tot <- 0
  totA <- 0
  totAf <- 0
  totB <- 0
  totBf <- 0
  for (i in 1:(length(preds))){
    if ((groups[i] == "Denmark" && preds[i] > (t)) || (groups[i] == "England" && preds[i] < (1 - t))){
      tot <- tot + 1
    }
    if ((groups[i] == "Denmark" && preds[i] > (t))){
      totA <- totA + 1
    } else if ((groups[i] == "England" && preds[i] > (t))){
      totAf <- totAf + 1
    }
    if ((groups[i] == "England" && preds[i] < (t))){
      totB <- totB + 1
    } else if ((groups[i] == "Denmark" && preds[i] < (t))){
      totBf <- totBf + 1
    }
  }
  prop <- tot/length(preds)
  propA <- totA/length(which((groups == "Denmark")))
  propAf <- totAf/length(which((groups == "England")))
  propB <- totB/length(which((groups == "England")))
  propBf <- totBf/length(which((groups == "Denmark")))
  accuracy <- c(accuracy, prop)
  accuracyA  <- c(accuracyA, propA)
  errorA <- c(errorA, propAf)
  accuracyB  <- c(accuracyB, propB)
  errorB <- c(errorB, propBf)
}  
DE_all_geo_rf <- accuracy
D_all_geo_rf <- accuracyA
D_all_geo_rf_error <- errorA
E_all_geo_rf <- accuracyB
E_all_geo_rf_error <- errorB
```

# Streptomyces reads analysis - BP dataset:

Note: Keep only the samples with more then 100.000 reads

Read data into R:

```
strepto_mash <- read.csv("kmers_similarity_matrices/streptomyces_matrix.csv")
# Add row names
rownames(strepto_mash) <- names(strepto_mash)
```

Read data into R - number of reads assigned to streptomyces genus:

```
strepto_num_reads <- read.csv("kmers_similarity_matrices/Number_streptomyces_reads.csv", row.names = 1, header = T)
```

Filter out samples with less then 100.000 reads:

```
# initialize the filtered dataframes
strepto_filtered <- strepto_mash
strepto_num_reads_filtered <- strepto_num_reads
# select sample to remove
remove <- which(strepto_num_reads$Number_streptomyces_reads < 100000)
k <- remove[1]
count <- 0
for (i in remove){
  k <- i - count
  strepto_filtered <- strepto_filtered[-k,-k]
  strepto_num_reads_filtered <- strepto_num_reads_filtered[-k,]
  count <- count + 1
}
```

Keep only reference samples:

```
strepto_mash_mat <- strepto_filtered[4:35, 4:35]
strepto_num_reads_filtered <- strepto_num_reads_filtered[4:35,]
```

Create group info file:

```
groups <- strepto_num_reads_filtered$Location

strepto_col_list <- c()
strepto_col_list <- append(strepto_col_list, rep("#33a02c", length(which(strepto_num_reads_filtered$Location == "Brazil"))))
strepto_col_list <- append(strepto_col_list, rep("#1f78b4", length(which(strepto_num_reads_filtered$Location == "RapaNui"))))
```

MDS plot:

```
library(ggfortify)
strepto_pca_mat <- prcomp(strepto_mash_mat, scale. = TRUE)

autoplot(strepto_pca_mat, x = 1, y = 2, pch = 16, label = F, size = 6, label.size = 3, alpha = 0.7, label.colour = NULL, label.hjust=0.0001, label.vjust=-0.5,
          colour = strepto_col_list) +
     theme_bw()
```

Get training dataset:

```
# get the PCs dataframe
strepto_train <- as.data.frame(strepto_pca_mat$x)
# remove the last PC which explain 0% of the variance
strepto_train <- strepto_train[,1:31]
```

# Logit - BP dataset:

Geography variable - streptomyces:

```
groups <- strepto_num_reads_filtered$Location

n <- nrow(strepto_train)
# placeholder for storing the i-th prediction
preds <- rep(NA, n)
for(i in 1:n) {
    dataf.strepto_train <- strepto_train[-i, ]
    dataf.test <- strepto_train[i, ]
    groups_tmp <- groups[-i]
    set.seed(123) 
    cv.lasso <- cv.glmnet(as.matrix(dataf.strepto_train), groups_tmp, alpha = a, family = "binomial")
    glmnet.fit <- glmnet(as.matrix(dataf.strepto_train), groups_tmp, alpha = a, family = "binomial", lambda = cv.lasso$lambda.min)
    preds[i] <- predict(glmnet.fit, as.matrix(dataf.test), type="response")
}


threshold <- seq(0, 1, length.out = 1000)
accuracy <- c()
accuracyA <- c()
errorA <- c()
accuracyB  <- c()
errorB <- c()
for (t in threshold){
  tot <- 0
  totA <- 0
  totAf <- 0
  totB <- 0
  totBf <- 0
  for (i in 1:(length(preds))){
    if ((groups[i] == "Brazil" && preds[i] < (1 - t)) || (groups[i] == "RapaNui" && preds[i] > (t))){
      tot <- tot + 1
    }
    if ((groups[i] == "Brazil" && preds[i] < (1 - t))){
      totA <- totA + 1
    } else if ((groups[i] == "RapaNui" && preds[i] < (1 - t))){
      totAf <- totAf + 1
    }
    if ((groups[i] == "RapaNui" && preds[i] > (1 - t))){
      totB <- totB + 1
    } else if ((groups[i] == "Brazil" && preds[i] > (1 - t))){
      totBf <- totBf + 1
    }
  }
  prop <- tot/length(preds)
  propA <- totA/length(which((groups == "Brazil")))
  propAf <- totAf/length(which((groups == "RapaNui")))
  propB <- totB/length(which((groups == "RapaNui")))
  propBf <- totBf/length(which((groups == "Brazil")))
  accuracy <- c(accuracy, prop)
  accuracyA  <- c(accuracyA, propA)
  errorA <- c(errorA, propAf)
  accuracyB  <- c(accuracyB, propB)
  errorB <- c(errorB, propBf)
}  
strepto_geo <- accuracy
B_strepto_geo <- accuracyA
B_strepto_geo_error <- errorA
P_strepto_geo <- accuracyB
P_strepto_geo_error <- errorB
```

Extraction protocol - streptomyces:

```
groups <- strepto_num_reads_filtered$extraction_protocol

n <- nrow(strepto_train)
# placeholder for storing the i-th prediction
preds <- rep(NA, n)
for(i in 1:n) {
    dataf.strepto_train <- strepto_train[-i, ]
    dataf.test <- strepto_train[i, ]
    groups_tmp <- groups[-i]
    set.seed(123) 
    cv.lasso <- cv.glmnet(as.matrix(dataf.strepto_train), groups_tmp, alpha = a, family = "binomial")
    glmnet.fit <- glmnet(as.matrix(dataf.strepto_train), groups_tmp, alpha = a, family = "binomial", lambda = cv.lasso$lambda.min)
    preds[i] <- predict(glmnet.fit, as.matrix(dataf.test), type="response")
}


threshold <- seq(0, 1, length.out = 1000)
accuracy <- c()
accuracyA <- c()
errorA <- c()
accuracyB  <- c()
errorB <- c()
for (t in threshold){
  tot <- 0
  totA <- 0
  totAf <- 0
  totB <- 0
  totBf <- 0
  for (i in 1:(length(preds))){
    if ((groups[i] == "Allentoft" && preds[i] < (1 - t)) || (groups[i] == "Damgaard" && preds[i] > (t))){
      tot <- tot + 1
    }
    if ((groups[i] == "Allentoft" && preds[i] < (1 - t))){
      totA <- totA + 1
    } else if ((groups[i] == "Damgaard" && preds[i] < (1 - t))){
      totAf <- totAf + 1
    }
    if ((groups[i] == "Damgaard" && preds[i] > (1 - t))){
      totB <- totB + 1
    } else if ((groups[i] == "Allentoft" && preds[i] > (1 - t))){
      totBf <- totBf + 1
    }
  }
  prop <- tot/length(preds)
  propA <- totA/length(which((groups == "Allentoft")))
  propAf <- totAf/length(which((groups == "Damgaard")))
  propB <- totB/length(which((groups == "Damgaard")))
  propBf <- totBf/length(which((groups == "Allentoft")))
  accuracy <- c(accuracy, prop)
  accuracyA  <- c(accuracyA, propA)
  errorA <- c(errorA, propAf)
  accuracyB  <- c(accuracyB, propB)
  errorB <- c(errorB, propBf)
}  
strepto_extraction_protocol <- accuracy
B_strepto_extraction_protocol <- accuracyA
B_strepto_extraction_protocol_error <- errorA
P_strepto_extraction_protocol <- accuracyB
P_strepto_extraction_protocol_error <- errorB
```

Sample type - streptomyces:

```
groups <- strepto_num_reads_filtered$sample_type

n <- nrow(strepto_train)
# placeholder for storing the i-th prediction
preds <- rep(NA, n)
for(i in 1:n) {
    dataf.strepto_train <- strepto_train[-i, ]
    dataf.test <- strepto_train[i, ]
    groups_tmp <- groups[-i]
    set.seed(123) 
    cv.lasso <- cv.glmnet(as.matrix(dataf.strepto_train), groups_tmp, alpha = a, family = "binomial")
    glmnet.fit <- glmnet(as.matrix(dataf.strepto_train), groups_tmp, alpha = a, family = "binomial", lambda = cv.lasso$lambda.min)
    preds[i] <- predict(glmnet.fit, as.matrix(dataf.test), type="response")
}


threshold <- seq(0, 1, length.out = 1000)
accuracy <- c()
accuracyA <- c()
errorA <- c()
accuracyB  <- c()
errorB <- c()
for (t in threshold){
  tot <- 0
  totA <- 0
  totAf <- 0
  totB <- 0
  totBf <- 0
  for (i in 1:(length(preds))){
    if ((groups[i] == "Bone" && preds[i] < (1 - t)) || (groups[i] == "Tooth" && preds[i] > (t))){
      tot <- tot + 1
    }
    if ((groups[i] == "Bone" && preds[i] < (1 - t))){
      totA <- totA + 1
    } else if ((groups[i] == "Tooth" && preds[i] < (1 - t))){
      totAf <- totAf + 1
    }
    if ((groups[i] == "Tooth" && preds[i] > (1 - t))){
      totB <- totB + 1
    } else if ((groups[i] == "Bone" && preds[i] > (1 - t))){
      totBf <- totBf + 1
    }
  }
  prop <- tot/length(preds)
  propA <- totA/length(which((groups == "Bone")))
  propAf <- totAf/length(which((groups == "Tooth")))
  propB <- totB/length(which((groups == "Tooth")))
  propBf <- totBf/length(which((groups == "Bone")))
  accuracy <- c(accuracy, prop)
  accuracyA  <- c(accuracyA, propA)
  errorA <- c(errorA, propAf)
  accuracyB  <- c(accuracyB, propB)
  errorB <- c(errorB, propBf)
}  
strepto_sample_type <- accuracy
B_strepto_sample_type <- accuracyA
B_strepto_sample_type_error <- errorA
P_strepto_sample_type <- accuracyB
P_strepto_sample_type_error <- errorB
```

# Random Forest - BP dataset:

Geography variable - streptomyces:

```
groups <- as.factor(strepto_num_reads_filtered$Location)

n <- nrow(strepto_train)
# placeholder for storing the i-th prediction
preds <- rep(NA, n)
for(i in 1:n) {
    dataf.strepto_train <- strepto_train[-i, ]
    dataf.test <- strepto_train[i, ]
    groups_tmp <- groups[-i]
    set.seed(123) 
    rf.fit <- randomForest(formula = groups_tmp ~ ., data = as.matrix(dataf.strepto_train))
    preds[i] <- as.numeric(predict(rf.fit, as.matrix(dataf.test), type = 'prob'))
}
```

```
## Warning in preds[i] <- as.numeric(predict(rf.fit, as.matrix(dataf.test), :
## number of items to replace is not a multiple of replacement length

## Warning in preds[i] <- as.numeric(predict(rf.fit, as.matrix(dataf.test), :
## number of items to replace is not a multiple of replacement length

## Warning in preds[i] <- as.numeric(predict(rf.fit, as.matrix(dataf.test), :
## number of items to replace is not a multiple of replacement length

## Warning in preds[i] <- as.numeric(predict(rf.fit, as.matrix(dataf.test), :
## number of items to replace is not a multiple of replacement length

## Warning in preds[i] <- as.numeric(predict(rf.fit, as.matrix(dataf.test), :
## number of items to replace is not a multiple of replacement length

## Warning in preds[i] <- as.numeric(predict(rf.fit, as.matrix(dataf.test), :
## number of items to replace is not a multiple of replacement length

## Warning in preds[i] <- as.numeric(predict(rf.fit, as.matrix(dataf.test), :
## number of items to replace is not a multiple of replacement length

## Warning in preds[i] <- as.numeric(predict(rf.fit, as.matrix(dataf.test), :
## number of items to replace is not a multiple of replacement length

## Warning in preds[i] <- as.numeric(predict(rf.fit, as.matrix(dataf.test), :
## number of items to replace is not a multiple of replacement length

## Warning in preds[i] <- as.numeric(predict(rf.fit, as.matrix(dataf.test), :
## number of items to replace is not a multiple of replacement length

## Warning in preds[i] <- as.numeric(predict(rf.fit, as.matrix(dataf.test), :
## number of items to replace is not a multiple of replacement length

## Warning in preds[i] <- as.numeric(predict(rf.fit, as.matrix(dataf.test), :
## number of items to replace is not a multiple of replacement length

## Warning in preds[i] <- as.numeric(predict(rf.fit, as.matrix(dataf.test), :
## number of items to replace is not a multiple of replacement length

## Warning in preds[i] <- as.numeric(predict(rf.fit, as.matrix(dataf.test), :
## number of items to replace is not a multiple of replacement length

## Warning in preds[i] <- as.numeric(predict(rf.fit, as.matrix(dataf.test), :
## number of items to replace is not a multiple of replacement length

## Warning in preds[i] <- as.numeric(predict(rf.fit, as.matrix(dataf.test), :
## number of items to replace is not a multiple of replacement length

## Warning in preds[i] <- as.numeric(predict(rf.fit, as.matrix(dataf.test), :
## number of items to replace is not a multiple of replacement length

## Warning in preds[i] <- as.numeric(predict(rf.fit, as.matrix(dataf.test), :
## number of items to replace is not a multiple of replacement length

## Warning in preds[i] <- as.numeric(predict(rf.fit, as.matrix(dataf.test), :
## number of items to replace is not a multiple of replacement length

## Warning in preds[i] <- as.numeric(predict(rf.fit, as.matrix(dataf.test), :
## number of items to replace is not a multiple of replacement length

## Warning in preds[i] <- as.numeric(predict(rf.fit, as.matrix(dataf.test), :
## number of items to replace is not a multiple of replacement length

## Warning in preds[i] <- as.numeric(predict(rf.fit, as.matrix(dataf.test), :
## number of items to replace is not a multiple of replacement length

## Warning in preds[i] <- as.numeric(predict(rf.fit, as.matrix(dataf.test), :
## number of items to replace is not a multiple of replacement length

## Warning in preds[i] <- as.numeric(predict(rf.fit, as.matrix(dataf.test), :
## number of items to replace is not a multiple of replacement length

## Warning in preds[i] <- as.numeric(predict(rf.fit, as.matrix(dataf.test), :
## number of items to replace is not a multiple of replacement length

## Warning in preds[i] <- as.numeric(predict(rf.fit, as.matrix(dataf.test), :
## number of items to replace is not a multiple of replacement length

## Warning in preds[i] <- as.numeric(predict(rf.fit, as.matrix(dataf.test), :
## number of items to replace is not a multiple of replacement length

## Warning in preds[i] <- as.numeric(predict(rf.fit, as.matrix(dataf.test), :
## number of items to replace is not a multiple of replacement length

## Warning in preds[i] <- as.numeric(predict(rf.fit, as.matrix(dataf.test), :
## number of items to replace is not a multiple of replacement length

## Warning in preds[i] <- as.numeric(predict(rf.fit, as.matrix(dataf.test), :
## number of items to replace is not a multiple of replacement length

## Warning in preds[i] <- as.numeric(predict(rf.fit, as.matrix(dataf.test), :
## number of items to replace is not a multiple of replacement length

## Warning in preds[i] <- as.numeric(predict(rf.fit, as.matrix(dataf.test), :
## number of items to replace is not a multiple of replacement length
```

```
threshold <- seq(0, 1, length.out = 1000)
accuracy <- c()
accuracyA <- c()
errorA <- c()
accuracyB  <- c()
errorB <- c()
for (t in threshold){
  tot <- 0
  totA <- 0
  totAf <- 0
  totB <- 0
  totBf <- 0
  for (i in 1:(length(preds))){
    if ((groups[i] == "Brazil" && preds[i] > (t)) || (groups[i] == "RapaNui" && preds[i] < (1 - t))){
      tot <- tot + 1
    }
    if ((groups[i] == "Brazil" && preds[i] > (t))){
      totA <- totA + 1
    } else if ((groups[i] == "RapaNui" && preds[i] > (t))){
      totAf <- totAf + 1
    }
    if ((groups[i] == "RapaNui" && preds[i] < (t))){
      totB <- totB + 1
    } else if ((groups[i] == "Brazil" && preds[i] < (t))){
      totBf <- totBf + 1
    }
  }
  prop <- tot/length(preds)
  propA <- totA/length(which((groups == "Brazil")))
  propAf <- totAf/length(which((groups == "RapaNui")))
  propB <- totB/length(which((groups == "RapaNui")))
  propBf <- totBf/length(which((groups == "Brazil")))
  accuracy <- c(accuracy, prop)
  accuracyA  <- c(accuracyA, propA)
  errorA <- c(errorA, propAf)
  accuracyB  <- c(accuracyB, propB)
  errorB <- c(errorB, propBf)
}  
strepto_geo_rf <- accuracy
B_strepto_geo_rf <- accuracyA
B_strepto_geo_rf_error <- errorA
P_strepto_geo_rf <- accuracyB
P_strepto_geo_rf_error <- errorB
```

Extraction protocol - streptomyces:

```
groups <- as.factor(strepto_num_reads_filtered$extraction_protocol)

n <- nrow(strepto_train)
# placeholder for storing the i-th prediction
preds <- rep(NA, n)
for(i in 1:n) {
    dataf.strepto_train <- strepto_train[-i, ]
    dataf.test <- strepto_train[i, ]
    groups_tmp <- groups[-i]
    set.seed(123) 
    rf.fit <- randomForest(formula = groups_tmp ~ ., data = as.matrix(dataf.strepto_train))
    preds[i] <- as.numeric(predict(rf.fit, as.matrix(dataf.test), type = 'prob'))
}
```

```
## Warning in preds[i] <- as.numeric(predict(rf.fit, as.matrix(dataf.test), :
## number of items to replace is not a multiple of replacement length

## Warning in preds[i] <- as.numeric(predict(rf.fit, as.matrix(dataf.test), :
## number of items to replace is not a multiple of replacement length

## Warning in preds[i] <- as.numeric(predict(rf.fit, as.matrix(dataf.test), :
## number of items to replace is not a multiple of replacement length

## Warning in preds[i] <- as.numeric(predict(rf.fit, as.matrix(dataf.test), :
## number of items to replace is not a multiple of replacement length

## Warning in preds[i] <- as.numeric(predict(rf.fit, as.matrix(dataf.test), :
## number of items to replace is not a multiple of replacement length

## Warning in preds[i] <- as.numeric(predict(rf.fit, as.matrix(dataf.test), :
## number of items to replace is not a multiple of replacement length

## Warning in preds[i] <- as.numeric(predict(rf.fit, as.matrix(dataf.test), :
## number of items to replace is not a multiple of replacement length

## Warning in preds[i] <- as.numeric(predict(rf.fit, as.matrix(dataf.test), :
## number of items to replace is not a multiple of replacement length

## Warning in preds[i] <- as.numeric(predict(rf.fit, as.matrix(dataf.test), :
## number of items to replace is not a multiple of replacement length

## Warning in preds[i] <- as.numeric(predict(rf.fit, as.matrix(dataf.test), :
## number of items to replace is not a multiple of replacement length

## Warning in preds[i] <- as.numeric(predict(rf.fit, as.matrix(dataf.test), :
## number of items to replace is not a multiple of replacement length

## Warning in preds[i] <- as.numeric(predict(rf.fit, as.matrix(dataf.test), :
## number of items to replace is not a multiple of replacement length

## Warning in preds[i] <- as.numeric(predict(rf.fit, as.matrix(dataf.test), :
## number of items to replace is not a multiple of replacement length

## Warning in preds[i] <- as.numeric(predict(rf.fit, as.matrix(dataf.test), :
## number of items to replace is not a multiple of replacement length

## Warning in preds[i] <- as.numeric(predict(rf.fit, as.matrix(dataf.test), :
## number of items to replace is not a multiple of replacement length

## Warning in preds[i] <- as.numeric(predict(rf.fit, as.matrix(dataf.test), :
## number of items to replace is not a multiple of replacement length

## Warning in preds[i] <- as.numeric(predict(rf.fit, as.matrix(dataf.test), :
## number of items to replace is not a multiple of replacement length

## Warning in preds[i] <- as.numeric(predict(rf.fit, as.matrix(dataf.test), :
## number of items to replace is not a multiple of replacement length

## Warning in preds[i] <- as.numeric(predict(rf.fit, as.matrix(dataf.test), :
## number of items to replace is not a multiple of replacement length

## Warning in preds[i] <- as.numeric(predict(rf.fit, as.matrix(dataf.test), :
## number of items to replace is not a multiple of replacement length

## Warning in preds[i] <- as.numeric(predict(rf.fit, as.matrix(dataf.test), :
## number of items to replace is not a multiple of replacement length

## Warning in preds[i] <- as.numeric(predict(rf.fit, as.matrix(dataf.test), :
## number of items to replace is not a multiple of replacement length

## Warning in preds[i] <- as.numeric(predict(rf.fit, as.matrix(dataf.test), :
## number of items to replace is not a multiple of replacement length

## Warning in preds[i] <- as.numeric(predict(rf.fit, as.matrix(dataf.test), :
## number of items to replace is not a multiple of replacement length

## Warning in preds[i] <- as.numeric(predict(rf.fit, as.matrix(dataf.test), :
## number of items to replace is not a multiple of replacement length

## Warning in preds[i] <- as.numeric(predict(rf.fit, as.matrix(dataf.test), :
## number of items to replace is not a multiple of replacement length

## Warning in preds[i] <- as.numeric(predict(rf.fit, as.matrix(dataf.test), :
## number of items to replace is not a multiple of replacement length

## Warning in preds[i] <- as.numeric(predict(rf.fit, as.matrix(dataf.test), :
## number of items to replace is not a multiple of replacement length

## Warning in preds[i] <- as.numeric(predict(rf.fit, as.matrix(dataf.test), :
## number of items to replace is not a multiple of replacement length

## Warning in preds[i] <- as.numeric(predict(rf.fit, as.matrix(dataf.test), :
## number of items to replace is not a multiple of replacement length

## Warning in preds[i] <- as.numeric(predict(rf.fit, as.matrix(dataf.test), :
## number of items to replace is not a multiple of replacement length

## Warning in preds[i] <- as.numeric(predict(rf.fit, as.matrix(dataf.test), :
## number of items to replace is not a multiple of replacement length
```

```
threshold <- seq(0, 1, length.out = 1000)
accuracy <- c()
accuracyA <- c()
errorA <- c()
accuracyB  <- c()
errorB <- c()
for (t in threshold){
  tot <- 0
  totA <- 0
  totAf <- 0
  totB <- 0
  totBf <- 0
  for (i in 1:(length(preds))){
    if ((groups[i] == "Allentoft" && preds[i] > (t)) || (groups[i] == "Damgaard" && preds[i] < (1 - t))){
      tot <- tot + 1
    }
    if ((groups[i] == "Allentoft" && preds[i] > (t))){
      totA <- totA + 1
    } else if ((groups[i] == "Damgaard" && preds[i] > (t))){
      totAf <- totAf + 1
    }
    if ((groups[i] == "Damgaard" && preds[i] < (t))){
      totB <- totB + 1
    } else if ((groups[i] == "Allentoft" && preds[i] < (t))){
      totBf <- totBf + 1
    }
  }
  prop <- tot/length(preds)
  propA <- totA/length(which((groups == "Allentoft")))
  propAf <- totAf/length(which((groups == "Damgaard")))
  propB <- totB/length(which((groups == "Damgaard")))
  propBf <- totBf/length(which((groups == "Allentoft")))
  accuracy <- c(accuracy, prop)
  accuracyA  <- c(accuracyA, propA)
  errorA <- c(errorA, propAf)
  accuracyB  <- c(accuracyB, propB)
  errorB <- c(errorB, propBf)
}  
strepto_extraction_protocol_rf <- accuracy
B_strepto_extraction_protocol_rf <- accuracyA
B_strepto_extraction_protocol_rf_error <- errorA
P_strepto_extraction_protocol_rf <- accuracyB
P_strepto_extraction_protocol_rf_error <- errorB
```

Sample Type - streptomyces:

```
groups <- as.factor(strepto_num_reads_filtered$sample_type)

n <- nrow(strepto_train)
# placeholder for storing the i-th prediction
preds <- rep(NA, n)
for(i in 1:n) {
    dataf.strepto_train <- strepto_train[-i, ]
    dataf.test <- strepto_train[i, ]
    groups_tmp <- groups[-i]
    set.seed(123) 
    rf.fit <- randomForest(formula = groups_tmp ~ ., data = as.matrix(dataf.strepto_train))
    preds[i] <- as.numeric(predict(rf.fit, as.matrix(dataf.test), type = 'prob'))
}
```

```
## Warning in preds[i] <- as.numeric(predict(rf.fit, as.matrix(dataf.test), :
## number of items to replace is not a multiple of replacement length

## Warning in preds[i] <- as.numeric(predict(rf.fit, as.matrix(dataf.test), :
## number of items to replace is not a multiple of replacement length

## Warning in preds[i] <- as.numeric(predict(rf.fit, as.matrix(dataf.test), :
## number of items to replace is not a multiple of replacement length

## Warning in preds[i] <- as.numeric(predict(rf.fit, as.matrix(dataf.test), :
## number of items to replace is not a multiple of replacement length

## Warning in preds[i] <- as.numeric(predict(rf.fit, as.matrix(dataf.test), :
## number of items to replace is not a multiple of replacement length

## Warning in preds[i] <- as.numeric(predict(rf.fit, as.matrix(dataf.test), :
## number of items to replace is not a multiple of replacement length

## Warning in preds[i] <- as.numeric(predict(rf.fit, as.matrix(dataf.test), :
## number of items to replace is not a multiple of replacement length

## Warning in preds[i] <- as.numeric(predict(rf.fit, as.matrix(dataf.test), :
## number of items to replace is not a multiple of replacement length

## Warning in preds[i] <- as.numeric(predict(rf.fit, as.matrix(dataf.test), :
## number of items to replace is not a multiple of replacement length

## Warning in preds[i] <- as.numeric(predict(rf.fit, as.matrix(dataf.test), :
## number of items to replace is not a multiple of replacement length

## Warning in preds[i] <- as.numeric(predict(rf.fit, as.matrix(dataf.test), :
## number of items to replace is not a multiple of replacement length

## Warning in preds[i] <- as.numeric(predict(rf.fit, as.matrix(dataf.test), :
## number of items to replace is not a multiple of replacement length

## Warning in preds[i] <- as.numeric(predict(rf.fit, as.matrix(dataf.test), :
## number of items to replace is not a multiple of replacement length

## Warning in preds[i] <- as.numeric(predict(rf.fit, as.matrix(dataf.test), :
## number of items to replace is not a multiple of replacement length

## Warning in preds[i] <- as.numeric(predict(rf.fit, as.matrix(dataf.test), :
## number of items to replace is not a multiple of replacement length

## Warning in preds[i] <- as.numeric(predict(rf.fit, as.matrix(dataf.test), :
## number of items to replace is not a multiple of replacement length

## Warning in preds[i] <- as.numeric(predict(rf.fit, as.matrix(dataf.test), :
## number of items to replace is not a multiple of replacement length

## Warning in preds[i] <- as.numeric(predict(rf.fit, as.matrix(dataf.test), :
## number of items to replace is not a multiple of replacement length

## Warning in preds[i] <- as.numeric(predict(rf.fit, as.matrix(dataf.test), :
## number of items to replace is not a multiple of replacement length

## Warning in preds[i] <- as.numeric(predict(rf.fit, as.matrix(dataf.test), :
## number of items to replace is not a multiple of replacement length

## Warning in preds[i] <- as.numeric(predict(rf.fit, as.matrix(dataf.test), :
## number of items to replace is not a multiple of replacement length

## Warning in preds[i] <- as.numeric(predict(rf.fit, as.matrix(dataf.test), :
## number of items to replace is not a multiple of replacement length

## Warning in preds[i] <- as.numeric(predict(rf.fit, as.matrix(dataf.test), :
## number of items to replace is not a multiple of replacement length

## Warning in preds[i] <- as.numeric(predict(rf.fit, as.matrix(dataf.test), :
## number of items to replace is not a multiple of replacement length

## Warning in preds[i] <- as.numeric(predict(rf.fit, as.matrix(dataf.test), :
## number of items to replace is not a multiple of replacement length

## Warning in preds[i] <- as.numeric(predict(rf.fit, as.matrix(dataf.test), :
## number of items to replace is not a multiple of replacement length

## Warning in preds[i] <- as.numeric(predict(rf.fit, as.matrix(dataf.test), :
## number of items to replace is not a multiple of replacement length

## Warning in preds[i] <- as.numeric(predict(rf.fit, as.matrix(dataf.test), :
## number of items to replace is not a multiple of replacement length

## Warning in preds[i] <- as.numeric(predict(rf.fit, as.matrix(dataf.test), :
## number of items to replace is not a multiple of replacement length

## Warning in preds[i] <- as.numeric(predict(rf.fit, as.matrix(dataf.test), :
## number of items to replace is not a multiple of replacement length

## Warning in preds[i] <- as.numeric(predict(rf.fit, as.matrix(dataf.test), :
## number of items to replace is not a multiple of replacement length

## Warning in preds[i] <- as.numeric(predict(rf.fit, as.matrix(dataf.test), :
## number of items to replace is not a multiple of replacement length
```

```
threshold <- seq(0, 1, length.out = 1000)
accuracy <- c()
accuracyA <- c()
errorA <- c()
accuracyB  <- c()
errorB <- c()
for (t in threshold){
  tot <- 0
  totA <- 0
  totAf <- 0
  totB <- 0
  totBf <- 0
  for (i in 1:(length(preds))){
    if ((groups[i] == "Bone" && preds[i] > (t)) || (groups[i] == "Tooth" && preds[i] < (1 - t))){
      tot <- tot + 1
    }
    if ((groups[i] == "Bone" && preds[i] > (t))){
      totA <- totA + 1
    } else if ((groups[i] == "Tooth" && preds[i] > (t))){
      totAf <- totAf + 1
    }
    if ((groups[i] == "Tooth" && preds[i] < (t))){
      totB <- totB + 1
    } else if ((groups[i] == "Bone" && preds[i] < (t))){
      totBf <- totBf + 1
    }
  }
  prop <- tot/length(preds)
  propA <- totA/length(which((groups == "Bone")))
  propAf <- totAf/length(which((groups == "Tooth")))
  propB <- totB/length(which((groups == "Tooth")))
  propBf <- totBf/length(which((groups == "Bone")))
  accuracy <- c(accuracy, prop)
  accuracyA  <- c(accuracyA, propA)
  errorA <- c(errorA, propAf)
  accuracyB  <- c(accuracyB, propB)
  errorB <- c(errorB, propBf)
}  
strepto_sample_type_rf <- accuracy
B_strepto_sample_type_rf <- accuracyA
B_strepto_sample_type_rf_error <- errorA
P_strepto_sample_type_rf <- accuracyB
P_strepto_sample_type_rf_error <- errorB
```

Create datafreme for roc curve plotting (random forest (dashed) and logit (continuos)):

```
# create the dataframe
roc_curve <- as.data.frame(B_geo_rf)
roc_curve <- cbind(roc_curve, B_geo_rf_error, P_geo_rf, P_geo_rf_error, B_geo, B_geo_error, P_geo, P_geo_error, B_extraction_protocol, B_extraction_protocol_error, P_extraction_protocol, P_extraction_protocol_error, B_sample_type, B_sample_type_error, P_sample_type, P_sample_type_error, B_extraction_protocol_rf, B_extraction_protocol_rf_error, P_extraction_protocol_rf, P_extraction_protocol_rf_error, B_sample_type_rf, B_sample_type_rf_error, P_sample_type_rf, P_sample_type_rf_error, D_best_geo, D_best_geo_rf, D_best_geo_error, D_best_geo_rf_error, E_best_geo, E_best_geo_rf, E_best_geo_error, E_best_geo_rf_error, D_all_geo, D_all_geo_rf, D_all_geo_error, D_all_geo_rf_error, E_all_geo, E_all_geo_rf, E_all_geo_error, E_all_geo_rf_error, B_strepto_geo, B_strepto_geo_error, B_strepto_geo_rf, B_strepto_geo_rf_error, P_strepto_geo, P_strepto_geo_error, P_strepto_geo_rf, P_strepto_geo_rf_error, B_strepto_extraction_protocol, B_strepto_extraction_protocol_error, B_strepto_extraction_protocol_rf, B_strepto_extraction_protocol_rf_error, P_strepto_extraction_protocol, P_strepto_extraction_protocol_error, P_strepto_extraction_protocol_rf, P_strepto_extraction_protocol_rf_error, B_strepto_sample_type, B_strepto_sample_type_error, B_strepto_sample_type_rf, B_strepto_sample_type_rf_error, P_strepto_sample_type, P_strepto_sample_type_error, P_strepto_sample_type_rf, P_strepto_sample_type_rf_error, threshold)
# BP dataset
roc_curve$avg <- (roc_curve$B_geo + rev(roc_curve$P_geo))/2
roc_curve$avg_err <- (roc_curve$B_geo_error + rev(roc_curve$P_geo_error))/2
roc_curve$ex_avg <- (roc_curve$B_extraction_protocol + rev(roc_curve$P_extraction_protocol))/2
roc_curve$ex_avg_err <- (roc_curve$B_extraction_protocol_error + rev(roc_curve$P_extraction_protocol_error))/2
roc_curve$st_avg <- (roc_curve$B_sample_type + rev(roc_curve$P_sample_type))/2
roc_curve$st_avg_err <- (roc_curve$B_sample_type_error + rev(roc_curve$P_sample_type_error))/2
roc_curve$avg_rf <- (roc_curve$B_geo_rf + rev(roc_curve$P_geo_rf))/2
roc_curve$avg_err_rf <- (roc_curve$B_geo_rf_error + rev(roc_curve$P_geo_rf_error))/2
roc_curve$ex_avg_rf <- (roc_curve$B_extraction_protocol_rf + rev(roc_curve$P_extraction_protocol_rf))/2
roc_curve$ex_avg_err_rf <- (roc_curve$B_extraction_protocol_rf_error + rev(roc_curve$P_extraction_protocol_rf_error))/2
roc_curve$st_avg_rf <- (roc_curve$B_sample_type_rf + rev(roc_curve$P_sample_type_rf))/2
roc_curve$st_avg_err_rf <- (roc_curve$B_sample_type_rf_error + rev(roc_curve$P_sample_type_rf_error))/2
# DE dataset
roc_curve$de_best_avg <- (roc_curve$D_best_geo + rev(roc_curve$E_best_geo))/2
roc_curve$de_best_avg_err <- (roc_curve$D_best_geo_error + rev(roc_curve$E_best_geo_error))/2
roc_curve$de_all_avg <- (roc_curve$D_all_geo + rev(roc_curve$E_all_geo))/2
roc_curve$de_all_avg_err <- (roc_curve$D_all_geo_error + rev(roc_curve$E_all_geo_error))/2
roc_curve$de_best_avg_rf <- (roc_curve$D_best_geo_rf + rev(roc_curve$E_best_geo_rf))/2
roc_curve$de_best_avg_err_rf <- (roc_curve$D_best_geo_rf_error + rev(roc_curve$E_best_geo_rf_error))/2
roc_curve$de_all_avg_rf <- (roc_curve$D_all_geo_rf + rev(roc_curve$E_all_geo_rf))/2
roc_curve$de_all_avg_err_rf <- (roc_curve$D_all_geo_rf_error + rev(roc_curve$E_all_geo_rf_error))/2
# BP dataset - streptomyces
roc_curve$avg_strepto <- (roc_curve$B_strepto_geo + rev(roc_curve$P_strepto_geo))/2
roc_curve$avg_strepto_err <- (roc_curve$B_strepto_geo_error + rev(roc_curve$P_strepto_geo_error))/2
roc_curve$ex_avg_strepto <- (roc_curve$B_strepto_extraction_protocol + rev(roc_curve$P_strepto_extraction_protocol))/2
roc_curve$ex_avg_strepto_err <- (roc_curve$B_strepto_extraction_protocol_error + rev(roc_curve$P_strepto_extraction_protocol_error))/2
roc_curve$st_avg_strepto <- (roc_curve$B_strepto_sample_type + rev(roc_curve$P_strepto_sample_type))/2
roc_curve$st_avg_strepto_err <- (roc_curve$B_strepto_sample_type_error + rev(roc_curve$P_strepto_sample_type_error))/2
roc_curve$avg_strepto_rf <- (roc_curve$B_strepto_geo_rf + rev(roc_curve$P_strepto_geo_rf))/2
roc_curve$avg_strepto_err_rf <- (roc_curve$B_strepto_geo_rf_error + rev(roc_curve$P_strepto_geo_rf_error))/2
roc_curve$ex_avg_strepto_rf <- (roc_curve$B_strepto_extraction_protocol_rf + rev(roc_curve$P_strepto_extraction_protocol_rf))/2
roc_curve$ex_avg_strepto_err_rf <- (roc_curve$B_strepto_extraction_protocol_rf_error + rev(roc_curve$P_strepto_extraction_protocol_rf_error))/2
roc_curve$st_avg_strepto_rf <- (roc_curve$B_strepto_sample_type_rf + rev(roc_curve$P_strepto_sample_type_rf))/2
roc_curve$st_avg_strepto_err_rf <- (roc_curve$B_strepto_sample_type_rf_error + rev(roc_curve$P_strepto_sample_type_rf_error))/2
```

# ROC curve plotting

BP dataset - geography (logit vs randomforest):

```
ggplot(roc_curve) +
  geom_path(aes(x = B_geo_rf_error, y = B_geo_rf), color = "#33a02c", linetype = "dashed") +
  geom_path(aes(x = P_geo_rf_error, y = P_geo_rf), color = "#1f78b4", linetype = "dashed") +
  geom_path(aes(x = B_geo_error, y = B_geo), color = "#33a02c") +
  geom_path(aes(x = P_geo_error, y = P_geo), color = "#1f78b4") +
  geom_abline(intercept = 0, slope = 1, linetype = "dashed") +
  ylim(0, 1) +
  theme_bw()
```

BP dataset - geography (logit vs randomforest) - Brazil:

```
ggplot(roc_curve) +
  geom_path(aes(x = B_geo_rf_error, y = B_geo_rf), color = "#7fc97f", linetype = "dashed") +
  geom_path(aes(x = B_geo_error, y = B_geo), color = "#7fc97f") +
  geom_abline(intercept = 0, slope = 1, linetype = "dashed") +
  ylim(0, 1) +
  theme_bw()
```

BP dataset - geography (logit vs randomforest) - Polynesia:

```
ggplot(roc_curve) +
  geom_path(aes(x = P_geo_rf_error, y = P_geo_rf), color = "#1f78b4", linetype = "dashed") +
  geom_path(aes(x = P_geo_error, y = P_geo), color = "#1f78b4") +
  geom_abline(intercept = 0, slope = 1, linetype = "dashed") +
  ylim(0, 1) +
  theme_bw()
```

BP dataset - geography (logit vs randomforest) - average between Brazil and Polynesia:

```
ggplot(roc_curve) +
  geom_path(aes(x = avg_err, y = avg), color = "#7fc97f") +
  geom_path(aes(x = avg_err_rf, y = avg_rf), color = "#7fc97f", linetype = 2) +
  geom_abline(intercept = 0, slope = 1, linetype = "dashed") +
  ylim(0, 1) +
  theme_bw()
```

BP dataset (all variables, logit vs randomforest):

```
ggplot(roc_curve) +
  geom_path(aes(x = avg_err, y = avg), color = "#7fc97f") +
  geom_path(aes(x = ex_avg_err, y = ex_avg), color = "#beaed4") +
  geom_path(aes(x = st_avg_err, y = st_avg), color = "#fdc086") +
  geom_path(aes(x = avg_err_rf, y = avg_rf), color = "#7fc97f", linetype = 2) +
  geom_path(aes(x = ex_avg_err_rf, y = ex_avg_rf), color = "#beaed4", linetype = 2) +
  geom_path(aes(x = st_avg_err_rf, y = st_avg_rf), color = "#fdc086", linetype = 2) +
  geom_abline(intercept = 0, slope = 1, linetype = "dashed") +
  ylim(0, 1) +
  theme_bw()
```

DE dataset (best vs all samples logit vs randomforest):

```
ggplot(roc_curve) +
  geom_path(aes(x = de_best_avg_err, y = de_best_avg), color = "#386cb0") +
  geom_path(aes(x = de_all_avg_err, y = de_all_avg), color = "#f0027f") +
  geom_path(aes(x = de_best_avg_err_rf, y = de_best_avg_rf), color = "#386cb0", linetype = 2) +
  geom_path(aes(x = de_all_avg_err_rf, y = de_all_avg_rf), color = "#f0027f", linetype = 2) +
  geom_abline(intercept = 0, slope = 1, linetype = "dashed") +
  ylim(0, 1) +
  theme_bw()
```

DE dataset (best vs all samples - logit):

```
ggplot(roc_curve) +
  geom_path(aes(x = de_best_avg_err, y = de_best_avg), color = "#386cb0") +
  geom_path(aes(x = de_all_avg_err, y = de_all_avg), color = "#f0027f") +
  geom_abline(intercept = 0, slope = 1, linetype = "dashed") +
  ylim(0, 1) +
  theme_bw()
```

BP dataset - streptomyces (logit vs randomforest):

```
ggplot(roc_curve) +
  geom_path(aes(x = avg_strepto_err, y = avg_strepto), color = "forestgreen") +
  geom_path(aes(x = ex_avg_strepto_err, y = ex_avg_strepto), color = "darkorchid4") +
  geom_path(aes(x = st_avg_strepto_err, y = st_avg_strepto), color = "darkorange3") +
  geom_path(aes(x = avg_strepto_err_rf, y = avg_strepto_rf), color = "forestgreen", linetype = 2) +
  geom_path(aes(x = ex_avg_strepto_err_rf, y = ex_avg_strepto_rf), color = "darkorchid4", linetype = 2) +
  geom_path(aes(x = st_avg_strepto_err_rf, y = st_avg_strepto_rf), color = "darkorange3", linetype = 2) +
  geom_abline(intercept = 0, slope = 1, linetype = "dashed") +
  ylim(0, 1) +
  theme_bw()
```

BP dataset - streptomyces vs whole metagenomes (logit):

```
ggplot(roc_curve) +
  geom_path(aes(x = avg_strepto_err, y = avg_strepto), color = "forestgreen") +
  geom_path(aes(x = ex_avg_strepto_err, y = ex_avg_strepto), color = "darkorchid4") +
  geom_path(aes(x = st_avg_strepto_err, y = st_avg_strepto), color = "darkorange3") +
  geom_path(aes(x = avg_err, y = avg), color = "#7fc97f") +
  geom_path(aes(x = ex_avg_err, y = ex_avg), color = "#beaed4") +
  geom_path(aes(x = st_avg_err, y = st_avg), color = "#fdc086") +
  geom_abline(intercept = 0, slope = 1, linetype = "dashed") +
  ylim(0, 1) +
  theme_bw()
```

BP dataset - streptomyces vs whole metagenomes (rf):

```
ggplot(roc_curve) +
  geom_path(aes(x = avg_strepto_err_rf, y = avg_strepto_rf), color = "forestgreen", linetype = 2) +
  geom_path(aes(x = ex_avg_strepto_err_rf, y = ex_avg_strepto_rf), color = "darkorchid4", linetype = 2) +
  geom_path(aes(x = st_avg_strepto_err_rf, y = st_avg_strepto_rf), color = "darkorange3", linetype = 2) +
  geom_path(aes(x = avg_err_rf, y = avg_rf), color = "#7fc97f", linetype = 2) +
  geom_path(aes(x = ex_avg_err_rf, y = ex_avg_rf), color = "#beaed4", linetype = 2) +
  geom_path(aes(x = st_avg_err_rf, y = st_avg_rf), color = "#fdc086", linetype = 2) +
  geom_abline(intercept = 0, slope = 1, linetype = "dashed") +
  ylim(0, 1) +
  theme_bw()
```

# Accuracy plots

Plot accuracy curve for the B-P dataset (three variables) - random forest vs logit:

```
ggplot() + 
  geom_line(aes(threshold, BP_geo), color = "#7fc97f") +
  geom_line(aes(threshold, BP_extraction_protocol), color = "#beaed4") +
  geom_line(aes(threshold, BP_sample_type), color = "#fdc086") +
  theme_bw() +
  ylim(0, 1)
```

Plot accuracy curve for the B-P dataset (three variables) - random forest:

```
ggplot() + 
  geom_line(aes(threshold, BP_geo_rf), color = "#7fc97f", linetype = 2) +
  geom_line(aes(threshold, BP_extraction_protocol_rf), color = "#beaed4", linetype = 2) +
  geom_line(aes(threshold, BP_sample_type_rf), color = "#fdc086", linetype = 2) +
  theme_bw() +
  ylim(0, 1)
```

Plot accuracy curve for the B-P dataset (three variables) - random forest vs logit:

```
ggplot() + 
  geom_line(aes(threshold, BP_geo), color = "#7fc97f") +
  geom_line(aes(threshold, BP_geo_rf), color = "#7fc97f", linetype = 2) +
  geom_line(aes(threshold, BP_extraction_protocol), color = "#beaed4") +
  geom_line(aes(threshold, BP_extraction_protocol_rf), color = "#beaed4", linetype = 2) +
  geom_line(aes(threshold, BP_sample_type), color = "#fdc086") +
  geom_line(aes(threshold, BP_sample_type_rf), color = "#fdc086", linetype = 2) +
  theme_bw() +
  ylim(0, 1)
```

Plot accuracy curve for the B-P dataset (geography variables) - random forest vs logit:

```
ggplot() + 
  geom_line(aes(threshold, BP_geo), color = "#7fc97f") +
  geom_line(aes(threshold, BP_geo_rf), color = "#7fc97f", linetype = 2) +
  theme_bw() +
  ylim(0, 1)
```

Plot accuracy curve for the D-E dataset (geography variables) - logit:

```
ggplot() + 
  geom_line(aes(threshold, DE_best_geo), color = "#386cb0") +
  geom_line(aes(threshold, DE_all_geo), color = "#f0027f") +
  theme_bw() +
  ylim(0, 1)
```

Plot accuracy curve for the D-E dataset (geography variables) - random forest:

```
ggplot() + 
  geom_line(aes(threshold, DE_best_geo_rf), color = "#386cb0", linetype = 2) +
  geom_line(aes(threshold, DE_all_geo_rf), color = "#f0027f", linetype = 2) +
  theme_bw() +
  ylim(0, 1)
```

Plot accuracy curve for the D-E dataset (geography variables) - logit vs random forest (all vs best samples):

```
ggplot() + 
  geom_line(aes(threshold, DE_best_geo), color = "#386cb0") +
  geom_line(aes(threshold, DE_all_geo), color = "#f0027f") +
  geom_line(aes(threshold, DE_best_geo_rf), color = "#386cb0", linetype = 2) +
  geom_line(aes(threshold, DE_all_geo_rf), color = "#f0027f", linetype = 2) +
  theme_bw() +
  ylim(0, 1)
```

Plot accuracy curve for the B-P & D-E datasets (geography variables) - logit:

```
ggplot() + 
  geom_line(aes(threshold, BP_geo), color = "#7fc97f") +
  geom_line(aes(threshold, DE_best_geo), color = "#386cb0") +
  geom_line(aes(threshold, DE_all_geo), color = "#f0027f") +
  theme_bw() +
  ylim(0, 1)
```

Plot accuracy curve for the B-P & D-E datasets (geography variables) - random forest vs logit:

```
ggplot() + 
  geom_line(aes(threshold, BP_geo_rf), color = "#7fc97f", linetype = 2) +
  geom_line(aes(threshold, DE_best_geo_rf), color = "#386cb0", linetype = 2) +
  geom_line(aes(threshold, DE_all_geo_rf), color = "#f0027f", linetype = 2) +
  theme_bw() +
  ylim(0, 1)
```

Plot accuracy curve for the B-P & D-E datasets (geography variables) - random forest vs logit:

```
ggplot() + 
  geom_line(aes(threshold, BP_geo), color = "#7fc97f") +
  geom_line(aes(threshold, BP_geo_rf), color = "#7fc97f", linetype = 2) +
  geom_line(aes(threshold, DE_best_geo), color = "#386cb0") +
  geom_line(aes(threshold, DE_all_geo), color = "#f0027f") +
  geom_line(aes(threshold, DE_best_geo_rf), color = "#386cb0", linetype = 2) +
  geom_line(aes(threshold, DE_all_geo_rf), color = "#f0027f", linetype = 2) +
  theme_bw() +
  ylim(0, 1)
```

Plot accuracy curve for the B-P (all variables) streptomyces data - random forest vs logit:

```
ggplot() + 
  geom_line(aes(threshold, strepto_geo), color = "#7fc97f") +
  geom_line(aes(threshold, strepto_geo_rf), color = "#7fc97f", linetype = 2) +
  geom_line(aes(threshold, strepto_extraction_protocol), color = "#beaed4") +
  geom_line(aes(threshold, strepto_extraction_protocol_rf), color = "#beaed4", linetype = 2) +
  geom_line(aes(threshold, strepto_sample_type), color = "#fdc086") +
  geom_line(aes(threshold, strepto_sample_type_rf), color = "#fdc086", linetype = 2) +
  theme_bw() +
  ylim(0, 1)
```

Plot accuracy curve for the B-P (geography variables) streptomyces data - random forest vs logit:

```
ggplot() + 
  geom_line(aes(threshold, strepto_geo), color = "#7fc97f") +
  geom_line(aes(threshold, strepto_geo_rf), color = "#7fc97f", linetype = 2) +
  theme_bw() +
  ylim(0, 1)
```

Plot accuracy curve for the B-P (geography variables) streptomyces data vs whole metagenomes (logit):

```
ggplot() + 
  geom_line(aes(threshold, strepto_geo), color = "forestgreen") +
  geom_line(aes(threshold, BP_geo), color = "#7fc97f") +
  theme_bw() +
  ylim(0, 1)
```

Plot accuracy curve for the B-P (geography variables) streptomyces data vs whole metagenomes (randomforest):

```
ggplot() + 
  geom_line(aes(threshold, strepto_geo_rf), color = "forestgreen", linetype = 2) +
  geom_line(aes(threshold, BP_geo_rf), color = "#7fc97f", linetype = 2) +
  theme_bw() +
  ylim(0, 1)
```

Plot accuracy curve for the B-P (geography variables) streptomyces data vs whole metagenomes (logit vs randomforest):

```
ggplot() + 
  geom_line(aes(threshold, strepto_geo), color = "forestgreen") +
  geom_line(aes(threshold, strepto_geo_rf), color = "forestgreen", linetype = 2) +
  geom_line(aes(threshold, BP_geo), color = "#7fc97f") +
  geom_line(aes(threshold, BP_geo_rf), color = "#7fc97f", linetype = 2) +
  theme_bw() +
  ylim(0, 1)
```

Plot accuracy curve for the B-P (extraction protocol variable) streptomyces data vs whole metagenomes (logit):

```
ggplot() + 
  geom_line(aes(threshold, strepto_extraction_protocol), color = "darkorchid4") +
  geom_line(aes(threshold, BP_extraction_protocol), color = "#beaed4") +
  theme_bw() +
  ylim(0, 1)
```

Plot accuracy curve for the B-P (sample type variable) streptomyces data vs whole metagenomes (logit):

```
ggplot() + 
  geom_line(aes(threshold, strepto_sample_type), color = "darkorange3") +
  geom_line(aes(threshold, BP_sample_type), color = "#fdc086") +
  theme_bw() +
  ylim(0, 1)
```
